# 118th Annual Meeting, Medical Library Association, Inc., Atlanta, GA, May 18–23, 2018

**DOI:** 10.5195/jmla.2019.672

**Published:** 2019-04-01

**Authors:** JJ Pionke, Ellen Aaronson

**Affiliations:** Proceedings Coeditor and Applied Health Sciences Librarian, University Library, University of Illinois at Urbana-Champaign, 1408 W. Gregory, Urbana IL 61801, pionke@illinois.edu; Proceedings Coeditor and Librarian, Libraries, Mayo Clinic, 200 First Street SW, Rochester, MN 55905, aaronson.ellen@mayo.edu

## INTRODUCTION

The Medical Library Association (MLA) held its 118th annual meeting in Atlanta, Georgia, May 18–23, 2018, at the Hyatt Regency Atlanta. The meeting theme was “Adapting, Transforming, Leading.” Total attendance for the meeting was 1,670, with 236 participating in continuing education courses. Additional meeting content—including the meeting program and various electronic presentations from the business meeting, plenary sessions, poster sessions, and program sessions—can be accessed by all meeting registrants via the MLA ’18 website.

## WELCOME TO MLA ’18

### Sunday, May 20, 2018

Executive Director Kevin Baliozian welcomed attendees and gave a pre-session announcement about a suicide in the hotel in a public area the night before. He discussed the steps MLA was taking to reach out to members who had been present as well as counseling services provided by the hotel. He then introduced MLA President Barbara A. Epstein AHIP, FMLA.

MLA President Barbara A. Epstein, AHIP, FMLA, welcomed attendees to the 2018 annual meeting. She updated the audience on a change to the meeting format in regard to how awards would be given out over a series of sessions, rather than all at once, and encouraged everyone to attend the plenary sessions and visit with vendors throughout the meeting. President Epstein then introduced Connie K. Machado, AHIP, who welcomed attendees on behalf of the Southern Chapter.

Connie K. Machado, AHIP: Good morning and welcome. The Southern Chapter and all its members extend a warm welcome to you in beautiful downtown Atlanta on behalf of MLA for this 118th annual meeting. Many of our local Southern Chapter members have worked with MLA to plan this exciting meeting and venue for all of you in attendance. We hope that you are able to adapt to our humidity and temperatures while you’re here in the South.

Founded in 1951, the Southern Regional Group later transformed to the Southern Chapter and has hosted three MLA meetings in this region. It has been seventeen years since the last meeting, which was held in Orlando, and we welcome you back with our warm Southern charm, great food, and hospitality. The Southern Chapter has a reputation for having fun and hosting great social events, so we hope you take some of our spirit of Southern hospitality home with you.

Throughout the Southern Chapter’s rich history, many of our founding librarians have served as MLA president and in other positions as leaders. Names like Eileen Cunningham, Mildred Crow Langner, and Mary Louise Marshall, as well as William (Bill) Postel, Alfred N. Brandon, Mildred Jordan, T. Mark Hodges, and a host of others. And I’m sure that there will be more leaders to come in the future.

Most of you have passed through Atlanta when traveling, at least at the airport. But there is much more to see and absorb here in the city’s rich history. Southern Chapter members are pleased to welcome you to Atlanta, home to the Centers for Disease Control and Prevention (CDC), Coca-Cola, and CNN, and plenty of dining experiences and fun places to visit during your stay. They also have seventy-five streets with the name “Peach” in it, so navigate carefully. So, again, I welcome you on behalf of the Southern Chapter members and MLA.

Now, please join me in welcoming these truly creative and dedicated members who have spent more than three years planning this Atlanta meeting: the 2018 National Program Committee (NPC) Cochair David A. Nolfi, AHIP; 2018 NPC Cochair Debra Berlanstein, AHIP; 2018 Local Assistance Committee Chair Sandra G. Franklin, AHIP, FMLA; and 2018 Local Assistance Committee Cochair Joe Swanson Jr. Let’s give them a big hand.

David A. Nolfi, AHIP: Thank you, Connie. On behalf of the MLA 2018 National Program Committee and the Local Assistance Committee, we officially welcome you to Atlanta, the first time for the MLA annual meeting. Our theme, “ATL: Adapting, Transforming, Leading,” reflects both this vibrant city and the spirit of our membership. The rising phoenix in our logo reflects the rise of Atlanta to become the dynamic leader in commerce, health care, research, education, and entertainment that it is today.

Debra Berlanstein, AHIP: As medical librarians, we too, are continually evolving and searching for innovative ways to grow, change, reimagine, and most of all, make a difference. We know you will be inspired by the programming and the many opportunities to connect and share with colleagues during your stay in this amazing city. We hope you will get out and about and explore the world outside the doors of the Hyatt Regency Atlanta.

David Nolfi: Planning this meeting over the last two years gave us the privilege of working with a great group of enthusiastic librarians whose hard work, energy, and commitment made cochairing the committee a pleasure. And so I ask now, would the members of the National Program Committee as well as the MLA staff who worked on the meeting please stand to be recognized. And now I ask you to please welcome Sandra Franklin, chair of the Local Assistance Committee, and Joe Swanson Jr., cochair.

Joe Swanson Jr.: Welcome to Atlanta. We’re so glad that you’re here and we hope you have an enjoyable time. We want you to enjoy the sessions and so forth. But in the meantime, we want you to have a little fun. We know that some of you have already been out and about, but you can visit places like the botanical gardens, the Jimmy Carter Library and Museum. You’ve already been around the attractions at the Centennial Olympic Park. And the Atlanta Symphony Orchestra will be performing *Candide*, by Leonard Bernstein.

So, as I said, we want you to enjoy the meeting, but in your spare time and for those who are going beyond the meeting, enjoy the city. And come back to see us! We have what we call Southern hospitality here. Thank you. [*Applause*.]

Sandra G. Franklin, AHIP, FMLA: Thanks, Joe. I’d also like to remind you that the service project for the Local Assistance Committee this year is called Street-to-Home. And in order to help with fundraising for that event, we have a silent auction that will be set up when you leave here and is in process now. We have some fun items, we have some nice items, we have a Kindle, we have a beautiful quilt that was made by Jan LaBeause, who is a member of the Southern Chapter and is a quilter, and it’s hanging in the backdrop of the silent auction. So I encourage you to come and participate so that we can leave a good showing for our local assistance project, Street-to-Home, here in Atlanta.

In addition to that, I’d like to thank personally everyone who has worked on this committee, but especially our Local Assistance Committee. They each had their tasks, they did their tasks and just did a fantastic job, and you’re seeing the results of that. So I’d like all of them to stand. Please stand [and be recognized] for this 2018 meeting. [*Applause*.] They’re scattered hither and yon, but just thank them if they’re near you.

Executive Director Baliozian returned to the podium to recognize and thank meeting planners and all the vendors who generously contributed to the meeting’s success. Each Gold sponsor gave a short one-minute speech. Gold sponsors were ClinicalKey, EBSCO Health, McGraw-Hill Education, and Wolters Kluwer.

Executive Director Balozian then welcomed back President Epstein who introduced the In Memoriam video. President Epstein then acknowledged distinguished MLA members and introduced Cynthia Beeler, AHIP, to discuss the Academy of Health Information Professionals (AHIP) and recognize new members.

### Recognitions

President Epstein returned to the stage and introduced Teresa L. Knott, AHIP, who joined President Epstein on the stage to acknowledge three new MLA fellows (FMLA):

- Patricia E. Gallagher, AHIP, FMLA, National Library of Medicine, Bethesda, MD
- Elaine Russo Martin, FMLA, Library Services, Countway Library, Harvard Medical School, Boston, MA
- Gerald J. Perry, AHIP, FMLA, Libraries, University of Arizona–Tucson

President Epstein then awarded the 2018 Presidential Award to the Task Force to Review MLA’s Competencies for Lifelong Learning and Professional Success:

- Gale G. Hannigan, AHIP, 2016/17 Task Force Chair, Health Sciences Library and Informatics Center, University of New Mexico–Albuquerque
- Paula Raimondo, AHIP, FMLA, 2015/16 Task Force Chair, Retired, Health Sciences & Human Services Library, University of Maryland–Baltimore
- Christopher Childs, Hardin Library for the Health Sciences, University of Iowa–Iowa City
- Martha Earl, AHIP, Preston Medical Library, University of Tennessee–Knoxville
- Kate Kelly, AHIP, Library, Royal College of Surgeons in Ireland, Dublin, Ireland
- Elizabeth Laera, AHIP, McMahon-Sibley Medical Library, Princeton Baptist Medical Center, Birmingham, AL
- Susan Lessick, AHIP, FMLA, Retired, University of California–Irvine
- Terri Ottosen, AHIP, Health Sciences Library, University of North Carolina–Chapel Hill
- Jodi L. Philbrick, AHIP, Department of Information Science, University of North Texas–Denton
- Caitlin Ann Pike, AHIP, IUPUI University Library, Indiana University–Purdue University–Indianapolis

President Epstein then introduced Jean Shipman, AHIP, FMLA, the 2017 Marcia C. Noyes Award recipient, who then awarded Ana D. Cleveland, AHIP, FMLA, Health Informatics Program, College of Information, University of North Texas–Denton, with 2018’s Noyes Award. Then President Epstein gave the presidential address.

## MLA PRESIDENTIAL ADDRESS: BARBARA A. EPSTEIN, AHIP, FMLA (PLENARY SESSION 1)

Barbara A. Epstein, AHIP, FMLA: At this point, it’s time for me to talk to you about my presidential year, and I’ve titled my talk, “Engaging the Future.” And in my talk today, I’m going to cover three things: what I did this year as president, and more importantly, what MLA did this year, and some things that you can do before and after you go home.

First, last June, I represented MLA at the Joint Meeting of the European Association of Health Information and Libraries, and the International Congress of Medical Librarianship in Dublin. I presented a poster on leadership training in US library associations, including MLA’s programs. I met with the presidents of other national and multinational health sciences libraries associations—including, from left to right on the bottom picture, Taiwan, Canada, Europe, Australia, and Africa—for a fascinating discussion of common trends and differences in our associations, and all expressed appreciation for the leadership of MLA and our National Library of Medicine (NLM) on their programs.

I also found time to socialize with other attendees whom you may recognize, including many from MLA, and I did kiss the Blarney stone, but there’s not a picture of that that I’m sharing.

Then I had the opportunity to participate in four chapter meetings: the South Central Chapter in Albuquerque, New Mexico, and you can see their lovely armadillo and several familiar faces; then on to the North Atlantic Health Sciences Libraries in Waltham, Massachusetts; my own Mid-Atlantic Chapter in Stanton, Virginia; and the Upstate New York and Ontario Chapter in Syracuse, New York.

We had an MLA board meeting in Chicago, and then it was on to Arizona in January—not too shabby for a girl from the north—and there were two chapter meetings, a joint meeting of two chapters, covering the northern and southern parts of California, Nevada, and Arizona. I enjoyed the chapters’ wonderful programming and the opportunity to provide updates of MLA’s activities. I gained an appreciation of the diverse activities and personalities of each of the chapters, and I thank the members for their hospitality.

Then, in April, I traveled to Washington, DC, for the annual Capitol Hill visits of the Joint MLA/AAHSL Legislative Task Force, AAHSL being the Association of Academic Health Sciences Libraries. I’ve been participating in these visits as a member for several years. If you have an opportunity to participate on this task force, I strongly urge you to say yes. It’s a chance to hear briefings from the leaders of NLM, the Association of American Medical Colleges (AAMC), and others, and to speak with congressional staffers, and sometimes even senators and representatives, about the issues that are important to our community: adequate funding for NLM and the National Institutes of Health (NIH) and support for legislation such as public access, copyright, and the Institute for Museum and Library Services (IMLS). It’s also just kind of fun to traipse around the Capitol for a day, and practice your lobbying skills, and see what’s going on.

But next, I want to talk about the many initiatives and other activities that are happening in our task forces and other committees. This has been a year of laying the groundwork for some major changes in the value of what we offer to members. But before I begin, I want to take a minute to remind you of what I said when I was a candidate for president in 2015.

I was asked about what changes in MLA I was most excited about and what kinds of experience I have with implementing change. I responded that MLA was at an important turning point in our organizational life, because our operations, in my opinion, had become stodgy and overly bureaucratic, and that I was excited by the top-to-bottom review of why we exist, how we function as an organization, and how we offer value to our members, and that it was my hope that this new process would lead to a more agile, flexible, and responsive association. And finally, I said that leading organizational change can be challenging, exciting, and even exhilarating. But however difficult and risky change may seem, it’s an even greater risk to stay in place.

Those of you who attended MLA updates at chapter meetings may remember this slide showing the varied communities and activities in which our members participate. You can see from this chart that we don’t currently have a great deal of cohesiveness and alignment between these various groups and activities. Some of our communities remain strong and active, but others face challenges in creating sustainable programs and recruiting leaders. There’s also a perception that our communities are often siloed in their activities and are fiscally and administratively challenging to support.

In the past five years, recommendations about the future of sections and special interest groups (SIGs) have been received by the MLA Futures Task Force, the Strategic Priorities Task Force, and the Rising Stars program. These recommendations suggested that MLA evolve our community structure to increase their effectiveness and empower members to participate at a more grassroots level, while retaining the important benefits of the membership structure that exist.

In response to recommendations from these two task forces, the Communities Strategic Goal Task Force—we’re big on task forces—was formed in May 2016, under the leadership of Rikke Sarah Ogawa, AHIP. Their charge was to strengthen our member communities by analyzing and recommending changes in community architecture and roles, leadership development, content, education, and communication.

In the past few years, the members of this task force have engaged in thoughtful consideration of their charge. They have spent many hours in discussion and met with the board, Section Council, and section and SIG leaders. They held an open meeting last year in Seattle and published blog posts and articles asking for feedback. They developed a set of guiding principles and then a draft proposal for a new organizational structure.

This member-driven proposal is included in your annual meeting app and online on the MLA website. The task force has already held a series of meetings with section and SIG leaders, as well as the board, and encouraged discussion at section and SIG and other meetings here in Atlanta.

They met with section leaders yesterday, and they have scheduled an open forum later this afternoon and an open meeting on Wednesday morning to gather feedback on their proposal. I strongly encourage each of you to attend one of these. Come with an open mind, ask questions, and offer your ideas to members. There are still a lot of details to be finalized based on your feedback. During the coming months, the task force will consider feedback, identify working groups to develop implementation details, and then will present their final proposal.

In the past year, we have also moved forward with our goal to advance our focus on education and professional development and to position MLA as the go-to education resource for health information professionals. We implemented a new committee structure to guide educational programming. Instead of one overworked Continuing Education Committee as in the past, we now have an integrated structure. The Education Steering Committee, led by Elizabeth Laera, AHIP, serves as the coordinating body for MLA education, and the Education Annual Programming Committee selects and plans the course roster for the annual meeting, along with webinars, symposia, and webcasts throughout the year.

Our goal was to appoint six curriculum planning committees, one for each of our six professional competencies. We began last year with leadership and management, and research and evidence-based practice, and newly appointed this spring are the other four: health information professionalism curriculum, information management curriculum, instruction and instruction design curriculum, and information services.

Another high priority of the Education Steering Committee, and one that is very important to me, is an early career boot camp to introduce new librarians to the basics of our six competency areas, and I encourage the steering committee to keep working on that.

Next, I’d like to turn to our new InSight Initiative. This started out last year as the corporate partners program, but we came up with a spiffier name. And it’s led by a small task force chaired by Gerald J. Perry, AHIP, FMLA. Their aim is to provide a forum for MLA experts, community partners, and industry partners to discuss issues of common interest in order to strengthen MLA’s position as a thought leader for our profession. We now have eleven supporting industry partners—these are listed on the slide—and AAHSL and Elsevier contributed sponsorship support.

The program format is to support two in-person summits each year for thirty to forty participants, equally divided between library experts and industry representatives. The first summit was held in Chicago last March, and discussion centered around user engagement. MLA participants were selected through an open application process.

Industry and MLA participants both rated the experience very highly and felt that it provided a setting for ongoing collaboration and possible new initiatives. Results of the discussion will be shared in four blog posts in the *MLA News*, and the first of these was posted by Gerry Perry on April 30. *Journal of the Medical Library Association (JMLA)* Editor Katherine G. Akers will publish a comprehensive report in the October issue of the *JMLA*, and a preprint is already linked on the Insight web page.

The second summit will be held in Chicago in September, so watch for more information and a call for applications in July.

You may be aware that IMLS awarded MLA a $240,000 grant to develop a research training institute. The immediate goal was to give our members an opportunity to immerse themselves in instruction and focused activities related to scholarly communication and publishing. The ultimate goal was to develop an online community of practice, where librarians are able to get research guidance from experts, make connections, and share their research experiences.

With this IMLS support, MLA will offer a weeklong workshop in research design for twenty participants. The first institute is scheduled for this coming July in Chicago. In addition to the instruction, participants will identify a research project and work with a mentor over the next year to complete the project and, ideally, to present and to publish the results. The institute is open to all MLA members. For the first round, there were forty-one applicants for twenty slots, but there will be another opportunity to participate because the workshop will be repeated in 2019.

And we certainly thank our other partners listed on this slide for their generous support.

One new initiative, and a very exciting initiative, at this annual meeting is a concurrent symposium for about 140 public librarians that will run all day Tuesday and Wednesday morning. The goal is to empower public librarians to address health-related questions from their patrons and to increase their awareness of authoritative consumer health information resources.

On Tuesday, some of the programming will run concurrently with other MLA programming and is open to our members, and some of the programs will be aimed more specifically at public librarians. Tuesday evening will be our combined Silver and Gold Networking Dinner, and the Wednesday morning speakers will be a joint session between the public librarian attendees and our MLA attendees.

This is an initiative of MLA and the Public Library Association, and it is funded by the National Network of Libraries of Medicine (NNLM) and the NIH All of Us research program. And this unique partnership allows us to leverage the expertise of our members, of you [*referring to the audience*], in building stronger relationships between these groups. And if you don’t know what the NIH All of Us research program is, plan to be here on Wednesday morning to hear Dr. Dara Richardson-Heron.

And just a few days ago, the board adopted a new strategic goal, which is to conduct an in-depth review of our annual meeting. The goal is to clarify our objectives in areas such as education, contributed content, association life, networking, and implementing best association management practices. The outcome will be aimed at enhancing the attendee and vendor experience and attracting a diverse community of our members, along with new attendees and vendors who may not currently view our meeting as the place to network or gain education.

We will also explore ways to improve our marketing and to align location and cost. Leading this three-year initiative will be an Annual Meeting Innovation Task Force—of course—that will be appointed very soon.

MLA needs your engagement, and here’s how you can participate in reinventing our association. First, you can go to one of the three open forums this afternoon at four-thirty. If you’re very energetic, you can go to more than one. If you’re really, really, really energetic, you can go to all of them. The Diversity and Inclusion Task Force asked you to visit the registration area before that and to register your ideas on several questions that are displayed. The group is interested in your thoughts about what diversity and inclusion—which I’ll abbreviate as D&I—means to you.

First, are you satisfied with the level of D&I in various MLA programs? What’s the best way to achieve D&I in MLA committee appointments? How can MLA gather D&I data? What are ways to make MLA scholarships more diverse and inclusive? And how does length of service at MLA relate to D&I initiatives? And if you cannot attend the committee’s open forum today, remember that you can attend the task force meeting on Wednesday morning to learn more.

And as my presidential year draws to a close, and my talk draws to a close, I have a lot of “thank-yous” to hand out. First, the library staff at University of Pittsburgh Health Sciences Library System (HSLS), especially our two associate directors, Fran Yarger and Renae Barger—Barger and Yarger; it’s like a law firm—along with management council, librarians, systems and operations staff, and our NNLM staff.

Shown here are just a sampling of the many smiling faces at HSLS. Everyone has been very patient with me over the past year when I was traveling or on a phone call or a video conference or just preoccupied or otherwise unavailable, and maybe they had a better time while I was going to those things. I appreciate their encouragement and their can-do attitude, and I thank them with all my heart.

Right here should have been a slide of my board members, of the Board of Directors, but it’s not here. But the board has been a wonderful group to work with, and I thank all of them. And I will miss the outgoing members, and I certainly welcome the incoming members.

I also want to say thank you to the staff at MLA headquarters. Many of you may not know each of these folks personally, but I can guarantee that they come to work every day dedicated to the success of our programs and our association.

And third, my family. We have two great daughters who married two great sons-in-law, who brought us four terrific grandchildren. And the littlest one is Hartley, who was born just last month, and she’s still tiny. She’s eating a lot, she’s more than eight pounds now, and we expect her to grow into her hair bows very soon. [*Laughter*.]

And last but not least, my husband, Arnold. Through the years, he has been my confidante, my sounding board, my gentle critic, my press agent, my legal advisor, my best friend, and my secret sauce. I have dragged him to a few MLA meetings, and I have to say he sometimes enjoys them way more than I do. Last night, he came back from the exhibit opening with just a whole bagful of stuff that he collected and a list of people he talked to. And I think marrying him was the best decision I ever made. [*Applause*.]

And thank you, the members of MLA, for the honor of serving as your president. My last act as president is to challenge you to stay engaged. Our profession has a long history, and our association has undergone changes that were unimaginable when it began in 1998. And together, we can overcome today’s challenges and create the future together.

So, I can’t really do a mic drop, but pretend I am. And this session is now concluded. We will reconvene at 10:30 a.m., in fifteen minutes, for the John P. McGovern Award Lecture that will be delivered by William Powers, author of the *New York Times’* best-seller with the intriguing title of *Hamlet’s Blackberry: Building a Good Life in the Digital Age*. So, please come back and join us for what promises to be a wonderfully stimulating discussion.

Thank you. [*Applause*.]

## OTHER PLENARY SESSIONS

### Plenary Session 2. Sunday, May 20: John P. McGovern Award Lecture

Introduction: Debra Berlanstein AHIP, cochair, 2018 National Program Committee, and associate director, Hirsh Health Sciences Library, Tufts University, Boston, MA

*Reviving the Human: Libraries in the Age of AI:* **William Powers**

### Plenary Session 3. Monday, May 21: The Janet Doe Lecture

Introduction: Julia F. Sollenberger, AHIP, FMLA, associate vice president and director, Medical Center Libraries and Technologies, and associate professor, Public Health Sciences, University of Rochester Medical Center, Rochester, NY

*Social Justice and the Medical Librarian*: **Elaine R. Martin**, director, Library Services, Countway Library, Harvard University Medical School, Boston, MA

### Plenary Session 4. Wednesday, May 23: Dara Richardson-Heron

Introduction: David A. Nolfi, AHIP, cochair, 2018 National Program Committee, and head, Research Engagement and Health Sciences/STEM Initiatives, and assessment coordinator, Gumberg Library, Duquesne University, Pittsburgh, PA

*An Overview of the All of Us Research Program:* **Dara Richardson-Heron, MD**, chief engagement officer, All of Us, National Institutes of Health (NIH)

### Plenary Session 5. Wednesday, May 23: David Satcher

Introduction: Joe Swanson Jr., cochair, 2018 Local Assistance Committee, and director, M. Delmar Edwards, M.D. Library, Morehouse School of Medicine, Atlanta, GA

**David Satcher, MD, PhD**, Satcher Health Leadership Institute, Morehouse School of Medicine, Atlanta, GA

## BUSINESS MEETING

The Business Meeting was held on Tuesday, May 22, 9:00 a.m.–10:25 a.m. President Barbara Epstein, AHIP, FMLA, welcomed everyone to the meeting. President Epstein then called to order the Business Meeting of the 2018 MLA annual meeting and asked if a quorum of 200 voting members, required for transaction of business, was present. Sergeant-at-Arms Linné Girouard, AHIP, confirmed the quorum, and President Epstein called on Secretary Lisa K. Traditi, AHIP, to move adoption of the Rules of Assembly. Secretary Traditi explained that the Rules of Assembly include information on addressing the chair, presenting motions, debating, and voting. At the direction of the Board of Directors, she moved that the Rules of Assembly as they appear on MLANET be adopted. Voting paddles were raised, and there being a majority in the affirmative, the rules were adopted. Secretary Traditi then announced that each meeting registrant had a printed copy of the *Official Program*, and that the agenda for the 2018 Business Meeting was on page 34. She moved that the agendas be adopted. The vote was affirmative, and the agendas were adopted.

President Epstein then asked Executive Director Kevin Baliozian to make introductions and announcements. Executive Director Baliozian presented the members of MLA’s 2017/18 Board of Directors: President Barbara A. Epstein, AHIP, FMLA; President-Elect Beverly Murphy, AHIP, FMLA; Immediate Past President Teresa L. Knott, AHIP; Treasurer Amy Blevins; Secretary Lisa K. Traditi, AHIP; Chapter Council Chair Melissa Ratajeski, AHIP; Section Council Chair Elizabeth R. Lorbeer, AHIP; and Directors Marie T. Asher, Stephanie Fulton, AHIP, Sandra Irene Martin, AHIP, and Melissa L. Rethlefsen, AHIP.

President Epstein then recognized and thanked retiring MLA Board Members and presented them with certificates as a token of respect and gratitude for work well done. She also expressed her gratitude to Teresa Knott, MLA president during the 2016/17 association year. Highlighting some of Knott’s initiatives, Past President Knott was presented a crystal gavel for a job well done.

President Epstein then called on Treasurer Amy Blevins to present the treasurer’s report.

Amy Blevins: Good morning, everyone. Can I get everyone to stand really quickly? Good. Okay, good. Just stretch right...left...right...left again. My report is only five minutes long, so you can stand and keep stretching the whole time if you’d like to.

So, unlike my poor dog, Cujo, who I thought would enjoy wearing a costume, I am going to try to make this as short and uncomfortable as—I mean, not uncomfortable—as possible. I know everyone loves math and numbers.

What I have here is a visual representation of our revenues and expenditures. And what you can see here is that a majority of our expenditures are coming from programming. That’s important to our members and adds value to our profession. And a majority of our revenue comes from the actual meeting that we’re at right now. It’s about 53% of our revenue that comes from the meeting. And 57% of that comes from our wonderful vendors.

I’m going to go through this a bit fast, but don’t worry, because there’s a report online that you can find, and there is also going to be an article in the *MLA News* that you can read that has all of the details about our budget.

We have been cutting costs like a machete cuts through a herd of zombies. Our operational cost reductions will yield a budget savings in 2018 and beyond of about $350,000. We have 2 fewer staff, we’ve done upgrades to our online applications, and we’ve transitioned the *Journal of the Medical Library Association (JMLA)* and *MLA News* from paper to online. But there is still an option for people who want the paper *JMLA* to get that. If the board and the staff had not made these decisions, our budget would be in a sad situation right now, so thank you, everyone.

At this point, the revenue for 2018 is about $3,000,000, so we get $180,000 in the annual meeting, with $80,000 coming from the new programming that’s devoted to joining our medical librarians, health sciences librarians, and the public librarians. We have an increase in membership—$53,000—so for the last 5 years, our membership had been decreasing by about 4.7% every year, and that’s due to changes in the profession. But we have stabilized with our institutional memberships.

And we now have an Institute of Museum and Library Services (IMLS) grant that is taking care of our research training initiative, and we have $112,000 related to 2 InSight Initiative Summits that are coming up this year, which is a wonderful partnership between our vendors and our MLA members.

In 2018 and beyond, we will be updating our professional competencies, expanding our education committee, and launching our MEDLIB-ED, which is already launched. So we’re making sure that all of our educational programming matches the needs of our membership, and we’re making sure that our educational programming is going to rise from the current 10% revenue to 20% revenue, which will be a cost-neutral strategy.

We also have our research training institute, our public librarians symposium, and our InSight Initiative, making sure we understand that it’s not a good idea for all of our revenue to come from our membership and the annual meeting, because if something happens to either of those two things, it puts MLA in a not-great situation.

If you want additional information, you can find our annual budget in the *MLA News*. Financial information is in the headquarters report. And I put this really simple breadcrumb trail for you to follow, so you can download that for yourselves. There is also a shortened uniform resource locator (URL), if you prefer to just go directly to the report for some reason. And then after June 1, the annual audit report can be found at the really short URL there, or the longer one, if you prefer. Any mistakes are my own and not Ray Naegele’s or Kevin’s. Thank you so much for all of your support in putting this together.

If you have any questions, you’re welcome to email Cujo, my dog, and he will respond to you, or you can email me or Ray, whatever you prefer. Thank you for all of your time. [*Applause*.]

Next, President Epstein called on Executive Director Kevin Baliozian to give the executive director’s report.

Kevin Baliozian: So, right before coming here—this is my family, and I went to a graduation in New York and made it to Atlanta just in time for the board meeting. And what I’ve decided to share with you is a few numbers, because I know that many of you have quoted these numbers out in your own meetings, so probably, that’s something you find interesting.

This is about membership. You heard from Amy that our membership had stabilized, and you can actually see that. One of the reasons is that we’ve got a lot of new members coming in, which is great, because there are a number of you who have been retiring—probably not in the room here. But we’ve stopped the drop in individual memberships, which is great.

“C” revenue, so continuing education (CE) revenue: You can see in the last column, which is the 2018 budget, that it significantly increased. The light blue is the IMLS grant, which is over several years, so that is going to continue in 2019 and part of 2020. The ambition here is for MLA to become THE place for education for our profession, also for those who aren’t members, to come and get education. And you can see that that number grows significantly in terms of the offerings and, therefore, in terms of revenue above half a million and more. So that’s an area that’s a long-term, five-year strategic plan, and you can see some of that happening nicely over the last two or three years.

If you look at MEDLIB-ED statistics, which was launched a year ago, you can see that two-thirds of the people who take a course in MEDLIB- ED are members, but that means one-third are not, which is really interesting. We awarded over 21,000 CE hours in a 1-year period. You can see here the number of learners. You can see that over 700 people have completed the self-assessment of the competencies on MEDLIB-ED. It’s free. If you haven’t done it, I invite you to do it. That has been helpful for you. It has also been helpful for the Education Steering Committee, because it provides a trove of evidence-based data, along with other surveys and other types of information to figure out what the right curriculums are.

This is usage of our catalogs. You can see a steady increase, one around our own offerings; the other one, you can find about a hundred courses from NLM that are listed on our catalog, and you can see that each quarter—each of these little bars is a quarter—that actually has gone up, which means that a lot of people are going to MEDLIB-ED to find courses.

Credentialing: You heard this on Sunday. It’s increasing every year. It’s impressive. It’s about a 16% increase over the last 2 years. So if you put that in the context of 2,700 or so members, not all Academy of Health Information Professionals (AHIP) members are members, but the percentage is significant and growing.

The annual meeting: There are a thousand of you who are registrants, health sciences librarians. That’s 35% of membership. That’s great. It’s also 65% of members who don’t attend the meeting, which we have to be very aware of as well. In addition, we have 145 public librarians who are currently in session, so please do greet them and make them feel welcome. We have 100 vendors, and that’s about 450 individuals who have come to meet with you as well. So that’s a nice crowd of 1,600, 1,700 people who are all gathered around this meeting.

Committee appointments: That’s an impressive number. In 2 years, we have gone from 172 applications to 237 this year. Everyone who applied has gotten appointed, essentially, in the last 3 years, so if you feel that you cannot get on a committee, that’s probably because you haven’t applied. So I suggest that you apply, because so far, our track record is pretty good as to appointing everyone to different positions, because there’s a lot of work to be done. So don’t hesitate. If you’re not sure, come speak to people who you feel comfortable with, and they will help you figure that out.

And lastly, I just want to mention the amazing effort done in grants and scholarships, which I think is a really important strategic direction also from the association. Last year, we granted $99,000 in grants and scholarships to 80 individuals. Out of those, about $18,000 came from sections and were granted by sections. The balance came from all sorts of different funds managed by MLA.

That number has increased by an impressive $46,000 this year, affecting 150 individuals. [*Applause*.] Why has it increased? Because of new programming, like the InSight Initiative, sponsored by the participating organizations, that allowed us to have the librarian attendees attend for free and pay for travel expenses, which is great.

The same thing with the IMLS grant: In addition to section and chapter contributions, for which we’re very appreciative, it allowed the twenty people for this coming session to be covered, either fully or significantly, in terms of their attendance for this five-day event.

So, there are a lot of efforts. We want to increase the efforts in terms of continuing education in general and annual meeting support, travel to the annual meeting. That’s a five-year project, and we look forward to that being launched probably around September or October around a great fundraising campaign to significantly increase our endowment for giving out grants over the next few years. So, more to come on that.

And lastly, after this meeting, vacation and kind of a zen in Canada on Lake Huron. And so between the graduation and that, I’ve had the pleasure of being here and meeting all of you. Thank you. [*Applause*.]

President Epstein then moved on to the annual reports. In the interest of time, annual reports were received in a block. The informational reports of the appointed officials, the councils, committees, task forces, representatives, sections, and chapters are found in the *2017/18 Annual Report of the Medical Library Association*. These reports are available on MLANET and will remain there throughout the year. They are also available in paper copy from the executive director’s office by request. There being no corrections or objections from the members, the reports were filed as presented.

President Epstein reported on the 2018/19 elections and introduced the Board of Directors: President Beverly Murphy, AHIP, FMLA; President-Elect Julia Esparza, AHIP; Immediate Past President Barbara A. Epstein, AHIP, FMLA; Treasurer Amy Blevins; Treasurer-Elect Shannon D. Jones, AHIP; Secretary Gurpreet Kaur Rana; Chapter Council Chair Melissa Ratajeski, AHIP; Section Council Chair Elizabeth R. Lorbeer, AHIP; and Directors Marie T. Ascher; Keith W. Cogdill, AHIP; Stephanie Fulton, AHIP; and Sandra Irene Martin, AHIP.

President Beverly Murphy, AHIP, FMLA, then presented outgoing President Epstein with the Presidential Cup and congratulated her for a year when, under her leadership, MLA broadened its opportunities to build its future. There were no resolutions and no new business, so Secretary Rana was called to adjourn the business meeting portion of the session, which was followed by several volunteer, award, fellowship, and grant recognitions.

### Recognitions

Immediate Past President Epstein led general recognitions of appointed officials; chapter chairs; section chairs and SIG conveners; committee, jury, and task force chairs; MLA representatives to allied organizations; and volunteers.

#### Annual meeting grants

- Ysabel Bertolucci MLA Annual Meeting Grant: Alexandria C. Quesenberry, UT Health Sciences Library, University of Tennessee Health Science Center–Memphis
- EBSCO/MLA Annual Meeting Grants (sponsored by EBSCO Information Services): Sarah Clarke, AHIP, Medical Library, US Army Medical Research Institute of Infectious Diseases, DSFederal, New Oxford, PA; Kelsey Leonard Grabeel, AHIP, Preston Medical Library, University of Tennessee–Knoxville; Alice Jean Jaggers, UAMS Library, University of Arkansas for Medical Sciences–Little Rock; and Jessica A. Koos, AHIP, Health Sciences Library, Stony Brook University, Stony Brook, NY

#### Professional development grants

- Naomi C. Broering Hispanic Heritage Grant: Dede Rios, AHIP, Optometric and Clinical Library Services, Rosenberg School of Optometry Library, University of Incarnate Word, San Antonio, TX
- Continuing Education Grant: Natalie Clairoux, Bibliothèque de la santé, Université de Montréal, Montréal, QC, Canada
- Hospital Libraries Section/MLA Professional Development Grant: Carrie D. Adams, Medical Library, Baptist Health, Jacksonville, FL, and Celine Soudant, Seymour J. Phillips Health Sciences Library, Mount Sinai Beth Israel, New York, NY
- Medical Informatics Section/MLA Career Development Grant: Carl Leak, University Libraries, George Mason University, Fairfax, VA

#### Scholarships

- MLA Scholarship: Justin Fuhr, Neil John Maclean Health Sciences Library, University of Manitoba–Winnipeg, Canada
- MLA Scholarship for Minority Students: Donna Baluchi, Spencer S. Eccles Health Sciences Library, University of Utah–Salt Lake City

#### Rising Stars

- 2018/19: Alexandria Leigh Brackett, AHIP, Cushing/Whitney Medical Library, Yale University, New Haven, CT; Rachel Helbing, AHIP, Health Sciences Library, University of Houston, Houston, TX; Liz Tucker Medical Library, National Jewish Health, Denver, CO; and Terry Kit Selfe, AHIP, Health Science Center Libraries, University of Florida–Gainesville
- 2017/18: Laura Menard, Ruth Lilly Medical Library, Indiana University–Indianapolis; Hanna Lee Schmillen, AHIP, Alden Library, Ohio University–Athens; Rachel Keiko Stark, AHIP, University Library, California State University–Sacramento; and Nicole Theis-Mahon, AHIP, Health Sciences Libraries, University of Minnesota–Minneapolis

#### Written works

- Ida and George Eliot Prize: Whitney A. Townsend, Patricia F. Anderson, Emily C. Ginier, Mark P. MacEachern, Kate M. Saylor, Barbara Lowther Shipman, and Judith E. Smith, Taubman Health Sciences Library, University of Michigan–Ann Arbor
- Erich Meyerhoff Prize: Vicki F. Croft, AHIP, FMLA, professor emeritus, Animal Health Library, Washington State University–Pullman, and Susanne K. Whitaker, AHIP, retired, Flower-Sprecher Veterinary Library, College of Veterinary Medicine, Cornell University, Ithaca, NY
- Rittenhouse Award (sponsored by Rittenhouse): Kelsa Bartley, Louis Calder Memorial Library, University of Miami Miller School of Medicine, Miami, FL

#### International awards

- Cunningham Memorial International Fellowship: 2018 Biliamin Oladele Popoola, University Library, University of Medical Sciences, Ondo City, Nigeria, and 2017 Tam Ha, Vinmec Medical University Project, Vinmec International Hospital, Vingroup, Hanoi, Vietnam
- Librarians without Borders®/Elsevier Foundation/Research4Life Grants (sponsored by the Elsevier Foundation): Emily J. Glenn, McGoogan Library of Medicine, University of Nebraska Medical Center–Omaha; Xan Goodman, AHIP, Lied Library, University of Nevada–Las Vegas, and Jill Barr-Walker, ZSFG Library, University of California–San Francisco; Phuntsho Norbu, Faculty of Nursing and Public Health Library, Khesar Gyalpo University of Medical Sciences of Bhutan, Thimphu, Bhutan; and Mboni Amiri Ruzegea, Library Services, Muhimbili University of Health and Allied Sciences, Dar es Salaam, Tanzania
- Librarians without Borders® Ursula Poland International Scholarship: Kristine M. Alpi, AHIP, William Rand Kenan, Jr. Library of Veterinary Medicine, North Carolina State University–Raleigh

#### Achievement awards

- Virginia L. and William K. Beatty Volunteer Service Award: Molly Knapp, AHIP, NNLM Training Office, National Network of Libraries of Medicine, Houston, TX
- Estelle Brodman Award for the Academic Medical Librarian of the Year: Holly K. Grossetta Nardini, Cushing/Whitney Medical Library, Yale University, New Haven, CT
- Clarivate Analytics/Frank Bradway Rogers Information Advancement Award (sponsored by Clarivate Analytics): Michael R. Kronenfeld, AHIP, FMLA, A.T. Still Memorial Library, A.T. Still University of the Health Sciences (ATUS), Mesa, AZ
- Lois Ann Colaianni Award for Excellence and Achievement in Hospital Librarianship: Edward J. Poletti, AHIP, Health Sciences Library, Central Arkansas Veterans Healthcare System–Little Rock
- Consumer Health Librarian of the Year Award: Erica Lake, AHIP, Spencer S. Eccles Health Sciences Library, University of Utah–Salt Lake City
- Carla J. Funk Governmental Relations Award: Julie A. Schneider, Ebling Library, University of Wisconsin–Madison
- Lucretia W. McClure Excellence in Education Award: Mary E. Edwards, Health Science Center Libraries, University of Florida–Gainesville

#### Research

- Clarivate Analytics/MLA Doctoral Fellowship (sponsored by Clarivate Analytics): Megan Threats, School of Information and Library Science, University of North Carolina–Chapel Hill
- Research Advancement in Health Sciences Librarianship Awards: University of Illinois–Chicago and University of Saskatchewan–Saskatoon, Canada
- David A. Kronick Traveling Fellowship: Erica Lake, AHIP, Spencer S. Eccles Health Sciences Library, University of Utah–Salt Lake City
- Donald A. B. Lindberg Research Fellowship: Sue Yeon Syn, Library and Information Science, Catholic University of America, Washington, DC, and JungWon Yoon, School of Information, University of South Florida–Tampa
- MLA Research, Development, and Demonstration Project Grants: Emily Brennan, Library, Medical University of South Carolina–Charleston; and Nadine Dexter, AHIP; Shalu Gillum, AHIP; Deedra Walton, AHIP; Natasha Williams, AHIP; Pamela R. Herring, AHIP; and Terri Gotschall, Harriet F. Ginsburg Health Sciences Library, University of Central Florida–Orlando

President Epstein next introduced 2018/19 MLA President Beverly Murphy, AHIP, FMLA, who delivered her inaugural address.

### Inaugural Address

Beverly Murphy, AHIP, FMLA: Good morning! Thank you, Barbara, for that lovely introduction. And thank you Stevie Wonder for those lyrics. That song really encapsulates how I feel about all of you: “You are the sunshine of my life.” There’s a line in that song that says, “that’s why I’ll always be around,” but there’s a little disclaimer to that, that is until I retire. Then it’s smooches, I’m out. I’m only sixty-one, though, so that won’t be for a few years.

So, I’ve titled this time with you today: If Chef Ramsay was coming to your house, would you cook? [*Laughter*.] Now, I hope most of you know who Chef Ramsay [Gordon Ramsay of *Hell’s Kitchen*] is, because if you don’t, this won’t make any sense at all. And anyway, what does that have to do with libraries, you say? Well, nothing. But it does have an analogy to what I want to say.

This is not a singing-in-the-choir speech. I consider this to be a conversation, which is why I’m sitting. Because all of you in this room are experts in your own areas. So this will be a singing-to-the choir conversation. Just think of me as doing a solo part right now. And I hope all of you in the background are singing, “Stand by Me.”

So, I’m in the kitchen, but I’m not necessarily the expert. We, collectively, are the experts. And if Chef Ramsay was coming to my house, heck, no, I wouldn’t cook, because he scares me. But I’m not afraid of you, because you are all my colleagues, and I feel comfortable in saying this: In the 120th year of the association, I am proud and honored to be the first African American president of the Medical Library Association. [*Applause*.]

I want to thank and commend everyone in this room, and those members and affiliates who are not here, for giving me this honor and opportunity, and in doing so, taking a positive stance, considering the world we are living in today.

I hear us saying collectively that no matter what race we are, what color we are, what ethnicity we are, what gender we are, or what gender identity we have, or whether we have physical issues, we are all information professionals with a common goal, and that is to be in an association of the most visible, valued, and trusted health information experts. Our principles, our beliefs, our ideals, and our standards, we will not compromise.

We are changing. We are becoming a more diverse society. But our profession has not yet caught up to that. And I want to thank Elaine R. Martin, as she so beautifully articulated that yesterday in her Doe lecture, so thank you Elaine. You said everything I wanted to say and didn’t have time. [*Applause*.]

Diversity drives excellence and makes us smarter, especially when we welcome it into our lives, our libraries, and our profession. And we are smart. The diversity of our staff and our organization is important and it’s necessary to help us survive and thrive in this journey. The melding of many different minds and thoughts, activities, feelings, and interactions produces a plethora of healthy, productive experiences that we all can gain from if we remain open and flexible.

We must reach the youngest generation—to middle school, high school, and maybe even earlier—because they are the next potential pool and the next generation of librarians. And that will be different from what we are now.

Research suggests that diversity in an organization has an advantage if the conditions are right. During the next year, the Diversity and Inclusion Task Force will continue to tackle some of these issues in an effort to create an environment where we all can thrive. I hope you had a chance to attend the Diversity and Inclusion Task Force’s open forum or some of the other sessions and activities we had here at the meeting. If you didn’t, I’m sure there will be other opportunities for your voice to be heard.

One way that we can continue to spread the seeds of diversity and inclusion is to expand our connections. Mentoring has really been a big thing for and a big part of my personal goals. I love the mutual interaction of giving as well as receiving, because I’ve learned from my mentees, as well as my mentors. It’s a win-win. And we had a great conversation and discussion about that yesterday in the Diversity and Inclusion Task Force Fish Bowl.

So I hope we will be able to expand the utilization of our MLA programs like Colleague Connection and the mentoring expertise directory that’s available on MLANET. And I would encourage everyone to mentor at least one person. It doesn’t matter whether you’re beginning your career or at the end of your career, because we can all learn from one another. Mentoring is important for recruitment and retention, as well, as we try to increase the diversity of the workforce that we’ve chosen to be a part of.

Another connection we have in MLA is our communities, and specifically right now, I’m referring to our sections and our special interest groups (SIGs), which leverage their skills in a very unique way. But MLA communities are changing, and that is requiring us to take a really good look at what we’re doing to see that we’re meeting the needs of members. The result of this exploration and discussion may require changes.

And that’s challenging, because everyone has a voice in our organization, but sometimes, in a personal way, we don’t always get what we want. So as Mick Jagger said, “You can’t always get what you want, but if you try sometimes, you might find you get what you need.” And at the end of the day, what’s best for our organization as a whole is what’s most important. So, the work that is being done by our Communities Strategic Goal Task Force is key to continuing interconnectivity, but hopefully in a more integrated way that expands across all the MLA activities.

So, over the next year, I want you to remain calm, I want you to remain open, I want you to remain flexible, and I want you to remain positive. [*Applause*.]

We are all teachers by nature, and that means all the staff, not just the librarians. Though we have different levels of how we do it, educational delivery is changing, and many people are not able to go to an MLA or chapter meeting or even get past their desks. Distance education is critical. It must honed so that it is seamless and convenient. So keep an eye out on our education committees and MEDLIB-ED, as they surround our competencies in an effort to stimulate growth of MLA as a learning destination for health professionals. That’s what we want to be: a learning destination for health professionals.

Lastly, I want to leave you with my personal message. I want you to meet my travel companion, Cardiana. She also answers to Cardiahna. Her personal pronouns are she, her, hers. And this message is open hearts and open minds. It’s open hearts because my heart is open to this profession. I have a love for librarianship and I have a love for my colleagues, which is all of you, as I celebrate our diversity and welcome the opportunity to know those of you I don’t already know. And it’s open mind, because where the heart goes, the mind follows.

And I’ve had to keep an open mind as I’ve seen libraries, librarianship, and librarians change and adapt over the thirty-eight-plus years that I’ve been in the profession. So I invite you to do the same, because guess what? There’s more to come, so, please feel free to share whatever you want. And you’ll notice on the back that Cardiana has a little zipper here. So if you have little cards or you have thoughts or ideas that you want to share with me, please feel free to give them to me. I’m going to put them in Cardiana, and she is going to travel with me. And there are already some things in there from some chapter meetings that I went to in the fall, and I want to thank everyone so far for doing that.

As we’re sitting in this room here in Atlanta—the home of Coke, fried foods, grits, and pie—we’re making history together. Thanks for taking this journey with me and helping to shape the framework for the next year and years to come. You will always be the sunshine in my life. Thank you. [*Applause*.]

I’d like to thank everyone. And please join us tonight for the Silver and Gold Networking Dinner, where we will present a few remaining awards and be entertained by our colleagues at MLA’s first-ever talent show. And get a sneak-peek at next year’s meeting, when the 2019 National Program Committee presents the MLA ’19 invitation. This session is now closed. [*Applause*.]

## SILVER AND GOLD NETWORKING DINNER, MLA ’19 INVITATION, AND RECOGNITIONS

The Silver and Gold Networking Dinner, sponsored by McGraw-Hill Education, was held on Tuesday, May 22, 2018, from 6:30 p.m.–10:00 p.m. The networking dinner was in a different format than in previous years and featured a talent show, which included a live band, singing, a sing-along, and poetry. The MLA ’19 Invitation to Chicago also took place and was hosted by James Dale Prince, AHIP, chair, 2019 National Program Committee, and executive director, Southeastern/Atlantic Region, National Network of Libraries of Medicine, University of Maryland–Baltimore; Mellanye J. Lackey, cochair, 2019 National Program Committee, and associate director, Education and Research, Spencer S. Eccles Health Sciences Library, University of Utah–Salt Lake City; Rosie Hanneke, AHIP, chair, 2019 Local Assistance Committee, and information services and liaison librarian, Library of the Health Sciences, University of Illinois–Chicago; and Debra Werner, cochair, 2019 Local Assistance Committee, and director, Library Research in Medical Education, John Crerar Library, University of Chicago, Chicago, IL. The Dental Section, recipient of the MLA Section Project of the Year Award, and the Mid-Atlantic Chapter of MLA, recipient of the Majors/MLA Chapter Project of the Year (sponsored by J.A. Majors), were recognized during the dinner.

## PROGRAM SESSIONS

Section programs were presented in 4 time slots: Sunday, May 20, 3:00 p.m.–4:25 p.m.; Monday, May 21, 10:30 a.m.–11:55 a.m., and 1:00 p.m.–2:25 p.m.; and Tuesday, May 22, 3:00 p.m.–4:25 p.m. Paper abstracts that were scheduled to be presented are available on the MLA ’18 website. The final version of the abstracts, reflecting only those presented at the meeting, is included as an online-only supplemental file to the April 2019 issue of the *Journal of the Medical Library Association*.

## POSTER SESSIONS

Poster sessions were presented in 4 time slots: Sunday, May 20, 2:00 p.m.–2:55 p.m.; Monday, May 21, 2:30 p.m.–3:25 p.m.; and Tuesday, May 22, 1:00 p.m.–1:55 p.m., and 2:00 p.m.–2:55 p.m. Poster abstracts that were scheduled to be presented are available on the MLA ’18 meeting website. The actual posters are available online in the MLA ’18 meeting website. The final version of the abstracts, reflecting only those posters presented at the meeting, is included as an online-only supplemental file to the April 2019 issue of the *Journal of the Medical Library Association*.

## OTHER MEETINGS AND EVENTS

### Pre-meeting activities

On Thursday, May 17, the MLA Board of Directors met. The MLA Board of Directors and Credentialing Committee met on Friday, May 18. On Saturday, May 19, the following groups met: 2019 National Program Committee; 2019 program planners; Chapter Council; Communities Strategic Goal Task Force; Joint Section Council/Chapter Council; National Network of Libraries of Medicine (NNLM) Steering Committee; Nominating Committee; and Section Council.

### Sunday, May 20

On Sunday, May 20, the following groups, sections, and SIGs met: AAHSL Future Leadership Committee; AAHSL Leadership Fellows Program; AAHSL New and Developing Libraries Committee; Ad Hoc Committee to Review Core Clinical Journals; African American Medical Library Alliance SIG Business Meeting; chapter treasurers orientation meeting; Collection Development Section Business Meeting; Consumer and Patient Health Information Section (CAPHIS) Business Meeting and Executive Committee Meeting; Data SIG Business Meeting; Education Steering Committee; Educational Media and Technologies Section (EMTS) Business Meeting #1; Fellows of MLA; Health Disparities SIG Lunch; International Cooperation Section Business Meeting; Interprofessional Education SIG Business Meeting; *JMLA* Editorial Board; Leadership Curriculum Committee; Metadata 2020: Join the Discussion to Help Improve the Quality of Metadata for Research; Midcontinental Chapter Meeting and Greet #1; Outreach and Marketing SIG Business Meeting; Research and Evidence-Based Practice Curriculum Committee; Research Section Research Award Judging; Research4Life Grants Workshop Planning Session; Resource Sharing SIG Business Meeting; and Systematic Reviews SIG Business Meeting. Exhibitor meetings included: Lunch & Learn: AAAS/Science: The Blurred Line between Fact and Fiction; Medical Librarian Lunch and Learn: EBSCO Health; Cell Press & *The Lancet* Lunch & Learn: Best Practices in Publishing and Reproducibility; Supporting Institutional Research and Raising the Profile of the Library; and The R2 Digital Library as a Health Sciences eBook Database.

### Monday, May 21

On Monday, May 21, the following groups, sections, and SIGs met: 2020 National Program Committee; AAHSL Research Services Committee; Around the World: Global Librarians’ Experiences; Awards Endowment Task Force Meeting; Awards Committee; Books Panel; Cancer Librarians Section Business Meeting; Clinical Librarians and Evidence-Based Healthcare SIG Business Meeting; Complementary and Alternative Medicine SIG Business Meeting; Data Catalog Collaboration Information Session; Dental Section Business Meeting; Department of Veterans Affairs Librarians SIG Business Meeting #2; EMTS Business Meeting #2; Gaming in Adult Learning SIG Business Meeting; Government Relations Committee; Health Information Professionalism Committee; History of the Health Sciences Section Business Meeting; Hospital Libraries Section (HLS) Executive Board Meeting and Business Meeting and Social; Information Services Curriculum Committee; Instruction and Instructional Design Committee; Latino SIG Business Meeting; LGBQT Health Sciences Librarians SIG Business Meeting; Leadership Curriculum Committee; Libraries in Curriculum SIG Business Meeting; Medical Humanities SIG Business Meeting; Medical Informatics Section Business Meeting; Medical Libraries: Starting from Scratch; MLA Research Training Institute; Molecular Biology and Genomics SIG Business Meeting; New York-New Jersey Chapter Board Meeting; Nursing and Allied Health Resources Section (NAHRS) Business Meeting and Executive Board; Osteopathic Libraries SIG Business Meeting; Pacific Northwest Chapter Business Meeting; Pediatric Librarians SIG Business Meeting; Pharmacy and Drug Information (PDI) Section Business Meeting; Professional Recruitment and Retention Committee; Public Health/Health Administration Section Business Meeting; Public Services Section Business Meeting; Scholarly Communications Committee; Solo Librarians SIG Business Meeting; Technical Services Section Business Meeting; Transitional Sciences Collaboration SIG Business Meeting; Veterinary Medical Libraries Section Business Meeting; and Vision Section SIG Business Meeting. Exhibitor meetings included: Lunch & Learn: How to Conduct Systematic Reviews the JBI Way; and How to Provide a World-Class Systematic Review Service Using Covidence.

### Tuesday, May 22

On Tuesday, May 22, the following groups, sections, and SIGs met: AAHSL Program and Education Committee; Bylaws Committee; Diversity and Inclusion Task Force; education committee chairs joint meeting; Federal Libraries Section Business Meeting; Health Association and Corporate Libraries Section (HACLS) Business Meeting; Information Literacy in Medical Education (ILME) SIG Business Meeting; Information Management Curriculum Committee; Institutional Animal Care and Use SIG Open Discussion; Joint 2018 and 2019 Contributed Content Work Group; Leadership and Management Section Business Meeting; Joseph Leiter NLM/MLA Lectureship Committee; Librarians without Borders® Committee; Medical Library Education Section (MLES) Business Meeting; Medical Library Group of Northern California and Nevada Business Meeting; Medical Library Group of Southern California and Arizona Business Meeting; Membership Committee; Midcontinental Chapter Meeting and Greet #2; MLA community managers and webmasters; MLA News Editorial Board; Research Section Business Meeting; Rising Stars presentations; Rising Stars program; section treasurers orientation; Social Justice Section Business Meeting; Southern Chapter Executive Committee; Systematic Reviews SIG Informal Meeting; and Technical Services Section Social. Exhibitor meetings included: Lunch & Learn: Elsevier Luncheon for Medical Librarians: Collaboration for Innovation.

### Wednesday, May 23

On Wednesday, May 23, the following groups, sections, and SIGs met: Community Strategic Goal Task Force Meeting 2; Education Annual Programming Committee (EAPC); Grants and Scholarships Committee; and Oral History Committee.

## OPEN FORUMS

Three open forums were held on Sunday, May 20, from 4:30 p.m.–5:25 p.m.:

- MLA Communities Strategic Goal
- MLA Diversity and Inclusion Strategic Goal
- MLA InSight Initiative Summit 1 Outcomes

## NATIONAL LIBRARY OF MEDICINE UPDATE

The National Library of Medicine (NLM) Update took place on Tuesday, May 22, 11:00 a.m.–11:55 a.m. Joyce E. B. Backus, associate director for library operations, began the session by introducing herself, Patricia F. Brennan, director of NLM, and Amanda J. Wilson, head, National Network Coordinating Office, National Network of Libraries of Medicine (NNLM), all of whom participated in the update.

Dr. Brennan presented an explanation of the NLM Strategic Plan, “A Platform for Biomedical Discovery and Data-Powered Health 2017–2027.” The three pillars (goals) were designed to target the future: (1) accelerate discovery and advance health through data-driven research; (2) reach more people through enhancement, dissemination, and engagement; and (3) build a workforce for data-driven research and health. In discussing the budget, Dr. Brennan said that NLM has received $22,000,000 for new investments in data science, accelerated training for librarians, and platform stabilization. She noted key accomplishments since February 2018.

Ms. Backus reported on strategic planning activities and outcomes. She discussed MEDLINE 2022, a 5-year plan to do a behind-the-scenes modernization of MEDLINE and more efficient delivery of information on TOXNET. She introduced PubMed Labs and PubOne that launched in October 2017 and is a test site to try new features for PubMed 2.0, which will be released in December 2018. The NLM website has been refreshed: the PubMed Health Portal will be going away, but PubMed and Bookshelf will still have systematic review and drug information. MedlinePlus now includes Lab Test information. Ms. Backus mentioned several other NLM activities including the DeBakey Fellowship, rare book loans, visiting library and information science students, and NLM associate fellows.

Ms. Wilson noted NNLM year three activities and illustrated an organizational chart of NNLM. She highlighted projects in health literacy, hurricane relief, funding for classes, and the Wikipedia edit-a-thon. She showed videos of three examples of NNLM activities: University of Arizona, St. Paul, MN, and University of Pittsburgh Medical Center (UPMC). The All of Us public library initiative was also mentioned. Ms. Wilson showed a slide of the *JAMA* article on the history of NNLM from 1985–2015.

The remainder of the session was devoted to questions and answers.

## LEGISLATIVE UPDATE

The Legislative Update was held on Tuesday, May 22, from 1:00 p.m.–1:55 p.m. Cristina Pope, AHIP, chair, MLA Governmental, Relations Committee, and director, Health Sciences Library, State University of New York (SUNY) Upstate Medical, University–Syracuse, moderated the session. Dina Paltoo, interim assistant director for policy development at NLM, presented information on the following: an overview of funding, functions of NLM, NLM’s most heavily used databases, statistics on PubMed Central, statistics on biomedical informatics and data science research, legislation and NLM, and bills and government activities that most affect NLM.

## OTHER SPECIAL EVENTS AND RECEPTIONS

### Saturday, May 19, 2018

- Welcome Reception and Opening of the Hall of Exhibits, 5:00 p.m.–7:30 p.m.

### Sunday, May 20, 2018

- MLA New Members/First-Time Attendees Program and Breakfast, 7:00 a.m.–8:55 a.m.
- Yoga Class, 7:30 a.m.–8:30 a.m.
- Library School Reunion Tea, 2:00 p.m.–2:55 p.m.
- Clinical Librarianship Happy Hour, 5:30 p.m.–7:00 p.m.
- International Visitors Reception, 6:00 p.m.–7:00 p.m.

### Monday, May 21, 2018

- Academy of Health Information Professionals Q&A Session, 2:30 p.m.–3:25 p.m.
- Diversity and Inclusion Task Force Fish Bowl, 5:00 p.m.–6:25 p.m.

### Tuesday, May 22, 2018

- Public Librarian Welcome Breakfast and Speed-Networking Event, 7:00 a.m.–8:55 a.m.
- Public Librarians Symposium, Overview and Sessions 1 & 2, 9:00 a.m.–10:25 a.m.
- Public Librarians Symposium, Morning Concurrent Sessions 1 & 2, 11:00 a.m.–11:55 a.m.
- Public Librarians Symposium, Afternoon Concurrent Sessions 1 & 2, 1:00 p.m.–1:55 p.m.
- MLA Book Authors and Prospective Authors Gathering, 2:00 p.m.–2:55 p.m.
- Public Librarians Symposium, Health Literacy Heroes Sessions 1 & 2, 4:30 p.m.–5:25 p.m.
- Silver and Gold Networking Dinner, 6:30 p.m.–10:00 p.m.

## SUNRISE SEMINARS

Exhibitors held Sunrise Seminars to provide information and to introduce new products and services. The following seminars were held:

### Sunday, May 20, 2018

- Artificial Intelligence (AI) in Health Care: From Adoption to Disruption
- Cochrane “Inside/Outside”: Update from Carol Lefebvre
- Embase Seminar: Librarian Roles in Teaching Evidence-Based Medicine
- The Power of Single Sign-On and the Benefits to All

### Monday, May 21, 2018

- American Psychological Association
- Springer Nature Competitive Intelligence: Research Trends in Health Care
- Three Trends That Eclipse Point-of-Care Tools

## TECHNOLOGY SHOWCASES

Ten Technology Showcases were held throughout Sunday, Monday, and Tuesday:

- Automating the Systematic Review Process: An Overview of DistillerSR’s New Productivity Enhancements
- Case Files: Teaching Case Collection
- Covering Your Nursing Bases: Research, Decision Support, and Competencies
- Supporting Your Interdisciplinary Care Dream Team
- Getting Beyond the Impact Factor: Using Bibliometrics for Research Evaluation at the NIH Library
- Taking Medical Research to the Next Level: 72,987,390 Searches Can’t Be Wrong
- The Ever-Evolving World of Medical E-Books
- From Data Standards to DermExpert: The Story of How an Image Collection Learned to Think
- Looking to the Future with EndNote
- Top Questions Medical Libraries Ask OpenAthens

## CONTINUING EDUCATION COURSES

The 2017/2018 Education Annual Programming Committee offered the following courses to 236 attendees on May 18 and 19, 2018.

### Friday, May 18, 2018

CE100 Advanced Searching Techniques and Advanced Strategy Design, **Instructors:** Julie Glanville, MCLIP, associate director, York Health Economics Consortium, University of York, York, United Kingdom, and Carol Lefebvre, HonFCLIP, independent information consultant, Lefebvre Associates, Oxford, United Kingdom

CE200 Dissemination in Action: Communicating Research in a Digital World, **Instructors:** Karen Gutzman, impact and evaluation librarian, and Patricia L. Smith, impact and dissemination librarian, Galter Health Sciences Library, Feinberg School of Medicine, Northwestern University, Chicago, IL

CE502 Statistics 101: A Primer in Statistical Methods for Health Sciences Librarians, **Instructors:** Sarah Young, senior librarian, Mellon Institute Library, Carnegie Mellon University, Pittsburgh, PA; Jin Wu, emerging technologies librarian, Norris Medical Library, University of Southern California–Los Angeles; and Ayaba Logan, research and education informationist and assistant professor, Medical Libraries, University of South Carolina–Charleston

CE600 Building Partnerships with Faculty, Clinicians, and Other Stakeholders, **Instructors:** Gwen Wilson, health sciences librarian, Mabee Library, Washburn University, Topeka, KS, and Kristen DeSanto, AHIP, clinical librarian, Health Sciences Library, Anschutz Medical Campus, University of Colorado–Aurora

### Saturday, May 19, 2018

CE101 Performing Systematic Reviews in Resource-Limited Settings, **Instructors:** Erin Eldermire, head, Flower-Sprecher Veterinary Library, Cornell University Library, Ithaca, NY; Sarah Young, senior librarian, Mellon Institute Library, Carnegie Mellon University, Pittsburgh, PA; and Lenny Rhine, FMLA, coordinator, E-Library Training Initiative, Librarians without Borders®/MLA, Gainesville, FL

CE102 Trials without Tribulations: Identifying Clinical Trials for Systematic Reviews and Other Clinical and Research Questions, **Instructors:** Julie Glanville, MCLIP, associate director, York Health Economics Consortium, University of York, York, United Kingdom, and Carol Lefebvre, HonFCLIP, independent information consultant, Lefebvre Associates, Oxford, United Kingdom

CE103 Effectiveness and Efficiency in Exhaustive Searches, **Instructors:** Wichor M. Bramer, biomedical information specialist, Medical Library, Erasmus University Medical Center, Rotterdam, the Netherlands, and Melissa L. Rethlefsen, AHIP, deputy director, Spencer S. Eccles Health Sciences Library, University of Utah–Salt Lake City

CE104 Upping Your Reference Game: Technologies and Strategies for Value-Added Reference Services, **Instructors:** Rachel Pinotti, AHIP, assistant library director, Education and Research Services, Levy Library, Icahn School of Medicine at Mount Sinai, New York, NY; Antonio P. DeRosa, AHIP, oncology consumer health librarian and assistant librarian faculty, Samuel J. Wood Library and C.V. Starr Biomedical Information Center, Weill Cornell Medicine, New York, NY; and Diana Delgado, AHIP, associate director, User Support, Research and Education, Samuel J. Wood Library and C.V. Starr Biomedical Information Center, Weill Cornell Medicine, New York, NY

CE105 Health Policy Research: Navigating Governmental and Legislative Sources, **Instructor:** Michele Malloy, research librarian, Congressional Research Service, Library of Congress, Washington, DC

CE300 Not Just Numbers: Teaching Students to Think Using Practical Curriculum Exercises, **Instructors:** Julia M. Esparza, AHIP, head, User Education and Outreach Services; Montie’ L. Dobbins, head, User Access Services/Circulation; and David C. Duggar, AHIP, head, Library Liaison Program; Health Sciences Library, Louisiana State University (LSU) Health–Shreveport; and Alexandria (Lexi) Brackett, AHIP, clinical support librarian, Harvey Cushing/John Hay Whitney Medical Library, Yale University, New Haven, CT

CE301 Innovations in Nursing Information Literacy: New Technologies, Approaches, and Ideas, **Instructor:** Jessica Sender, librarian, College of Nursing, Michigan State University–East Lansing

CE302 Peeking Under the Hood: Understanding, Assessing, and Improving Your Library’s Website and LibGuides, **Instructors:** Andy Hickner, web services librarian, Harvey Cushing/John Hay Whitney Medical Library, Yale University, New Haven, CT, and Susanna Galbraith, virtual services librarian, Health Sciences Library, McMaster University, Hamilton, ON, Canada

CE303 Evidence-Based Teaching: Finding and Applying the Best Evidence to Instruction, **Instructors:** Jamie Conklin, research and education librarian, and Megan von Isenburg, AHIP, associate dean, Library Services and Archives, Medical Center Library & Archives, Duke University, Durham, NC

CE400 Do You Want to Be a Library Director? Knowledge, Skills, and Career Paths for Library Leaders, **Instructors:** Heidi Heilemann, AHIP, associate dean and knowledge management director, Lane Medical Library and Knowledge Management Center, Stanford University, Stanford, CA; M.J. Tooey, AHIP, FMLA, associate vice president, Academic Affairs, and executive director, Health Sciences and Human Services Library, University of Maryland–Baltimore; and Gabriel Rios, director, Ruth Lilly Medical Library, Indiana University–Indianapolis

CE401 Digital Storytelling: Communication for Greater Impact, **Instructor:** Erinn Aspinall, AHIP, strategic initiatives librarian and communications coordinator, Bio-Medical Library, Health Sciences Libraries, University of Minnesota–Minneapolis

CE500 What Did You Hear? Qualitative Data Analysis, **Instructor:** Ayaba Logan, research and education informationist and assistant professor, Medical Libraries, Medical University of South Carolina–Charleston

CE501 Introduction to Visualization Principles, **Instructors:** Marci Brandenburg, bioinformationist, and Jean Song, assistant director, Academic and Clinical Engagement, Taubman Health Sciences Library, University of Michigan–Ann Arbor

CE503 Introduction to Data Analysis and Visualization with R, **Instructor:** Lisa Federer, AHIP, research data informationist, NIH Library, National Institutes of Health, Bethesda, MD

CE601 Learning to Liaise with Health Professions, **Instructors:** David A. Nolfi, AHIP, health sciences librarian and library assessment coordinator, Gumberg Library, Duquesne University, Pittsburgh, PA, and Carolyn Schubert, interim director, Research and Education Services, and health sciences and nursing librarian, Carrier Library, James Madison University, Harrisonburg, VA

## RESOURCES AND SERVICES

The online itinerary planner (sponsored by Wolters Kluwer) allowed attendees to peruse programs and events online. Complimentary WiFi was available throughout the Hyatt Regency Atlanta courtesy of The JAMA Network. Live streaming was available on Twitter using the hashtag #MLANET18, and volunteer bloggers, the Local Assistance Committee, and the 2018 National Program Committee contributed to the official meeting blog with meeting tips, announcements, and more. For those seeking new jobs and prospective employers, the Job Placement Center was open from Saturday through Tuesday, and the MLA Resume Clinic was available Saturday through Monday. The Hall of Exhibits was open Saturday evening through Monday.

## SUPPLEMENTAL FILES

Appendix MLA ’18 Program Session Abstracts

Appendix MLA ’18 Poster Session Abstracts

