# Supplementary material for: 118th Annual Meeting, Medical Library Association, Inc., Atlanta, GA, May 18–23, 2018
Source: J Med Libr Assoc. 2019 Apr 1;107(2):E1–E20. doi: 10.5195/jmla.2019.672 (PMC6466505; doi:10.5195/jmla.2019.672)
Supplement: Appendix [file jmla-107-E1-s001.pdf]

# Medical Library Association

# MLA '18 Program Session Abstracts

MLA'18

Adapting | Transforming | Leading

May 18–23 Atlanta, GA

www.mlanet.org | #mlanet18

Any unsolicited abstracts for the annual meeting undergo a process of blind peer review. Abstracts of papers intended for session programs are reviewed by members of a panel of reviewers sponsoring the programs. The final decision on program speakers rests with the program planners.

Sunday, May 20, 2018, 3:00 PM – 4:25 PM
Room: Embassy F (International Tower, Exhibit Level)

---

# Session: Instruction and Instructional Design 1 (ID-1)

## Moderator: Claire B. Joseph, AHIP

### Adaptations in Global Health Education: Reinventing a Course Collaboration through a Global Data Lens

**Gurpreet K. Rana**
Global Health Coordinator, Taubman Health Sciences Library, Ann Arbor, Michigan

**Laura S. Rozek**
Associate Professor, Environmental Health Sciences, School of Public Health, Ann Arbor, Michigan

**Objectives:** In fall 2017, Taubman Health Sciences Library's Global Health Coordinator and a School of Public Health faculty member at University of Michigan collaborated together to reinvent a global health course. The re-designed course focused on building students' skills in using open data to interpret challenges and solutions in global health. Students integrated data visualization tools, open data and infographics in case-based instruction.

**Methods:** Syllabus and course objectives for a long standing global public health course were revisited and redesigned in consultation with an instructional assessment expert. Strategies included increasing student interactivity, case-based instruction, and identifying focused specialists as guest lecturers. The instructors developed the course be more data-driven to illustrate the impact of data analysis in global public health, focusing on application of open global development data and open data visualization tools. Course highlights included use of data visualizations to identify health inequities, use of data sources to understand the Global Burden of Disease, and the impact of the Millennium Development Goals (MDGs) and Sustainable Development Goals (SDGs) in global development and investigation of infographics in public health promotion and communication. Students were required to develop global health case studies in an SDG framework as their final capstone projects.

**Keywords:** global health, instruction, public health, course-integration, data management, disparities, collaboration

## Adapting Best Practice for Education: Reinventing a Biomedical Informatics Course

**Kathy J. Davies**
Associate Director for Research, Robert B. Greenblatt MD Library, Augusta, Georgia

**Brenda Seago**
Professor and Director of Libraries, Augusta University, Augusta, Georgia

**Virginia (Ginny) Durham**
Project Manager, Augusta University, Reese Library, Augusta, Georgia

**Ashley Cullum**
Instructional Design Manager, Information Technology, Augusta, Georgia

**Objectives:** To create an innovative course to teach the principles of biomedical informatics to health professionals, educators, and librarians to build a cohort of change agents and leaders.

**Methods:** An academic medical center library developed a proposal for an intensive training in response to a National Library of Medicine (NLM) initiative to expand biomedical informatics instruction. The proposal recommended a hybrid approach to include both online course modules and face to face instruction. The course would be taught in a resort setting to facilitate participant interaction. A key component was integrating instructional technology including lecture capture, electronic white board, audience response system, and course software. The course was awarded funding and course planning finalized with NLM.

Course curriculum was a survey of key topics including genomics, imaging, NLM resources, public health, mathematical modeling, and data management. Information Technology and Continuing Education (CE) provided instructional technology and CE credit for the multidisciplinary audience. Faculty were evaluated on subject knowledge and teaching skills; feedback was incorporated into subsequent courses.

**Results:** Multiple course sessions were taught to 207 health professionals, librarians, or informationists selected from a nation-wide pool. The 77 librarian participants represented 28 states, District of Columbia and Puerto Rico. The university team was successful in creating a course that integrates health care leaders' expertise with online technology. The university team emphasized best practices for interactive education and incorporated a problem- based activity during their invited evening session. A library faculty member was invited to teach the NLM resources session and has partnered to teach a hands on sessions searching genomics and informatics databases.

**Conclusions:** Course participants experienced high career impact and expanded their biomedical informatics knowledge based on course evaluations, reusing course content, course webinars and informal feedback. Armed with the knowledge, tools, skills, and a supportive network, participants left the Biomedical Informatics Course ready to be successful agents of change within their institutions.

**Keywords:** Biomedical informatics, online education, instructional technology, course development

## Adapting a New Model for Library Orientation: The Clinical Case Presentation

**Nancy A. Bianchi**
Health Sciences Librarian, University of Vermont, Burlington, Vermont

**Gary S. Atwood**
Research and Instruction Librarian, University of Vermont, Dana Medical Library, Burlington, Vermont

**Objectives:** This presentation describes how a liaison librarian adapted the clinical case presentation format in medicine to a library orientation for new pediatric residents.

**Methods:** An introduction to the medical library has been a long-standing educational activity for new residents at the medical center. In the past, this orientation consisted primarily of a passive lecture and library tour. Currently, medical librarians are attempting to reduce the amount of lecture content in their own presentations in an effort to support an initiative by medical education to eliminate lectures from its curriculum by 2019. As a part of this effort, the library orientation for new pediatric residents underwent a complete redesign in 2016. The centerpiece of this redesign was the idea of adapting the familiar clinical case presentation model in medicine to a library orientation. This presentation describes an interactive case presentation about a pediatrics resident with a clinical information need, and the library resources and services relevant to that need.

**Results:** Nearly 40 pediatrics residents and medical students have participated in the transformed library orientation since 2016. In addition, the chief residents have played integral roles in promoting this library activity by serving as scribes for the case presentations and by continuing to incorporate discussion of library resources and services at educational conferences beyond the library orientation. Results from completed library evaluations in 2017 also spoke of the unexpected success of this adapted activity. 100% of the returned evaluations rated the library orientation as “Excellent” or “Very Good”. Comments included “Engaging example”, “Very Effective” and “Crucial talk especially for med students”.

**Conclusions:** The success of this adaptation should give librarians the motivation and encouragement to explore teaching formats in their liaison areas, and to adapt those models to library education and orientation activities, just as we did with the clinical case presentation format in a pediatrics residency program.

**Keywords:** Library Orientation; Clinical Case Presentation; Medical Education; Residents; Information Need

## Adapting to the Real World: Transforming Nursing Information Literacy Education in Four Intermountain West Universities

**Nena Schvaneveldt, AHIP**
Assistant Director of Library Services, Reference and Instruction Librarian, University Library, South Jordan, Utah

**Anne R. Diekema**
Assistant Professor / Librarian, Gerald R. Sherratt Library, Cedar City, Utah

**Elizabeth (Betsy) S. Hopkins**
Nursing and Communication Disorders Librarian, Harold B. Lee Library, Provo, Utah

**Brandon Patterson**
Technology Engagement Librarian, Spencer S. Eccles Health Sciences Library, Salt Lake City, Utah

**Objectives:** This study aimed to connect information literacy instruction more directly to nursing practice by surveying current information practices of professional nurses. While research examining the information practices of nurses exists, there is little connection to information literacy instruction. The researchers plan to modify existing nursing information literacy instruction based on study results.

**Methods:** We surveyed recent nursing alumni of four baccalaureate nursing education programs based in the Intermountain West. The questions of the detailed 59-item survey addressed five main areas: demographics

and current employment situation; information needs; information sources; Information environment at workplace; barriers to finding accessing, and evaluating information; and education & instruction. Within these five areas, survey questions covered a variety of information-seeking behaviors, including which resources are used, how often, and for what application in a clinical setting. Questions also determined nurses' comfort level and confidence with a variety of resources and information problem solving tasks. Finally, the survey asked nurses where they learned their information-seeking skills and dispositions, and how their nursing education could have better prepared them to find and evaluate information. The survey response rate was 21.0%.

**Results & Conclusions:** Results indicated that nurses sought information most often through conversations with nursing colleagues and physicians. Traditionally taught electronic databases were underutilized in the workplace, although nurses reported high confidence in their ability to find and evaluate information. Time and access were substantial barriers to seeking and evaluating information. Nurse respondents recommended to modify instruction by incorporating more case-based real-life scenarios to model information seeking within the existing workflows of the profession, including instruction on freely available online information resources; and introducing time constraints to searching conforming with reality. Additionally, nurses recommend integrating information literacy instruction into student clinical experiences.

**Keywords:** nurses
BSN
information literacy instruction
information seeking behavior
information practices

Sunday, May 20, 2018, 3:00 PM – 4:25 PM
Room: Embassy C (International Tower, Exhibit Level)

---

# Session: Information Services 1 (IS-1)

## Moderator: Joseph Harzbecker, AHIP

### Advancing, Transforming, and Leading Innovation through an Academic Health Sciences Library's Innovation Space

**M.J. Tooey, AHIP, FMLA**
Associate Vice President, Academic Affairs and Executive Director of Health Sciences and Human Services Library, Health Sciences/Human Services Library, U of MD, Baltimore, Baltimore, Maryland

**Objectives:** Makerspaces are quickly evolving to become essential components of the learning and research environments at universities. The neutrality and centrality of the library environment is a perfect place to locate a makerspace encouraging collaboration and multidisciplinary approaches to problem solving while utilizing new and emerging technologies such as 3D printing, data visualization, and training.

**Methods:** In 2014, a team was formed tasked with investigating the feasibility of developing a makerspace within the Library. Extensive research was done. Local makerspaces were visited. Meetings were held with technology innovators in the local area. An extensive white paper was written recommending the development of a library-based makerspace. Funding was found and a prominent location for the space was identified.

**Results:** The Innovation Space opened in April 2015. By October 2015 the size was doubled to meet growing demand. The Innovation Space (iSpace), contains 3D printers, digital scanners, learning tools, a button maker, Google Cardboards, and VR equipment. A robust array of classes and learning experiences have been developed. A newsletter focused on the creative and innovative use of technology is published monthly and a new Meet the Maker series has been instituted. Opportunities for creative collaborations have evolved. Assignments to use the space are being written into the curriculum and the library has been approached about inclusion in grants.

**Conclusions:** Plans are underway to add additional resources such as high performance computing, simulations, data visualization, and advanced graphics capabilities. The addition of the iSpace has been a boon to the programs and perception of the Library. Although only in existence for two and a half years, the space has elevated the visibility of the library and the appreciation for the expertise of the team supporting it.

**Keywords:** Technology, Innovation, Makerspaces, Collaboration, Education

### Bibliometric Analysis of Scholarly Journal Literature on the Zika Virus (ZIKV), 1952-2016

**Frances A. Delwiche**
Library Associate Professor, University of Vermont, Burlington, Vermont

**Objectives:** The primary objective of this study was to characterize the scholarly journal literature on the Zika Virus (ZIKV) on the basis of publication date, source journal, subject of source journal, country of first author, and publication type. A secondary objective was to develop a reproducible method of bibliometric analysis for this topic that would enable future updates to this study.

**Methods:** A search on the word Zika or ZIKV was conducted in PubMed, using both MeSH headings and keywords. The search was limited to articles published between 01/01/1952 and 12/31/2016. Results were imported into an EndNote library using a modified import filter, then exported to an Excel file using a customized output style. Exclusions included duplicates, off-topic articles, errata or corrections, and articles from popular magazines. Data were analyzed by publication date, in five-year increments. A list of journals in which the articles were published was generated, and frequency ranked. Frequency ranked lists were also obtained for the subjects of the journals and countries of first authors. Finally, a comparison of the number of opinion pieces versus research articles was conducted for six infectious diseases, including ZIKV.

**Results:** Approximately 93% of articles in PubMed on ZIKV were published in 2016, with the remaining 7% published between 1952 and 2015. Though nearly 600 journals contributed articles on the topic, one third of the articles were contributed by just 19 journals. Conversely, over 81% of journals contributed three articles or fewer, and 55% contributed only one article. Articles were contributed by authors working in over eighty countries scattered worldwide. The publication type comparison for six infectious diseases showed that ZIKV had both the highest percentage of opinion pieces and the lowest percentage of research articles.

**Conclusions:** This study provided a broad overview of the state of scholarly journal publishing on ZIKV as of the end of 2016. The analysis revealed a remarkably rapid response to the ZIKV epidemic of 2015-2016 by the medical, scientific, and publishing communities, drawing contributions from scholars worldwide, working across dozens of disciplines. By describing a reproducible methodology, the study provided a mechanism for conducting periodic updates that will track the evolution and maturation of the ZIKV literature well into the future.

**Keywords:** bibliometrics
journal productivity
scholarly journals
scientific communication
Zika Virus
ZIKV

## Creating New Research Services: Library Support for Electronic Lab Notebooks

**Carrie L. Iwema, AHIP**
Coordinator of Basic Science Services, Health Sciences Library System, Pittsburgh, Pennsylvania

**Melissa Ratajeski, AHIP**
Coordinator of Data Services; IACUC Liaison, Health Sciences Library System, Pittsburgh, Pennsylvania

**Objectives:** Lab notebooks are used to permanently record experimental research documentation such as hypotheses, methodology/instrumentation, workflows, results, and analyses. Researchers are transitioning from traditional paper to digital notebooks to facilitate searching of notebook entries, improve back-up capabilities, and enhance reproducibility through rigor and transparency. Our objective is to explore current and potential library-based research support roles for electronic lab notebooks (ELN).

**Methods:** Data Service librarians from the health sciences library and main library at a large research university collaborated to design an eighteen-question Qualtrics survey assessing ELN support practices at academic/health sciences libraries. The survey was available for one month and was distributed to relevant MLA, ALA, and SLA listservs. Questions covered whether the respondent's library currently provides, plans to, doesn't plan to, or previously provided ELN-related services including: instructional workshops, consultations, technical troubleshooting support, or guidance to researchers via Libguides. The survey also gathered names of any institutionally-licensed ELN platforms; library outreach activities; target audience; service utilization; librarian time commitment; additional institutional stakeholders; and experience commentary. We will use descriptive statistics and trend analysis to inform library-based ELN service models and influence the second stage of the project, interviews with researchers on their data collection practices and advantages/barriers to ELN usage.

**Keywords:** Data, research, eln, lab, survey, descriptive, service, experimental, support

## Developing a Program for Institutional Animal Care and Use Committee Compliance: Benefits for Researchers and Libraries

**Melissa Funaro**
Clinical Librarian, Cushing/Whitney Medical Library, New Haven, Connecticut

**Kate Nyhan**
Research and Education Librarian, Cushing/Whitney Medical Library, New Haven, Connecticut

**Alexandria L. Brackett, AHIP**
Clinical Librarian, Cushing/Whitney Medical library, New Haven, Connecticut

**Andy Hickner**
Web Services Librarian, Cushing/Whitney Medical Library, New Haven, Connecticut

**Rolando Garcia Milian**
Biomedical Research Support Librarian, Cushing/Whitney Medical Library, New Haven, Connecticut

**Objectives:** This paper describes a partnership between medical librarians and an Institutional Animal Care and Use Committee (IACUC) to assist basic science researchers and promote compliance. The IACUC ensures compliance with the federal requirement that comprehensive searches for alternatives to animal experimentation must be performed before protocols are approved.

**Methods:** Librarians partner with the IACUC, serving as committee members and consulting with researchers doing animal experiments at professional schools across the University.

**Results:** This comprehensive program includes ten librarians who peer-reviewed each other's IACUC searches, have created an online guide, and delivered and evaluated hands-on training workshops. The librarians also create search hedges for IACUC concepts, such as refinement through pain reduction and replacement with non-animal models. These hedges build capacity and improve the quality of the mediated searches. Additionally, librarians educate animal researchers and institutional stakeholders about the value of reporting guidelines - for the scientific goal of reproducibility, the ethical goal of eliminating unnecessary pain, and the librarians' goal of retrieving relevant information with greater specificity.

**Conclusions:** Thanks to librarians' new collaborative relationship with the IACUC, researchers now rely on librarians to meet their short-term needs (for mediated searches that achieve the committee's quality standards) and long-term needs (for search training and reporting guidelines). This program supports the institutional goal of compliance with federal regulations and, through hedge development and peer review of search strategies, promotes the team's professional development.

**Keywords:** Institutional Animal Care and Use Committee, IACUC, Animal Use Alternatives

## Leading Easy Access to Content: RA21 Pilots Transform Researcher Productivity and Privacy

**Michelle M. Volesko Brewer**
Librarian/Market Intelligence Manager, Wolters Kluwer, Lawrenceville, New Jersey

**Jean P. Shipman, AHIP, FMLA**
VP, Global Library Relations, Elsevier, Cottonwood Heights, Utah

**Objectives:** To improve the user experience when accessing scholarly content by offering seamless, non-traditional authentication means that are not dependent on workflow or location. Further, to offer an anonymous form of authentication that enhances security, permits customizations, and enables the collection of accurate usage analytics.

**Methods:** Building on CNI's Report on the Authentication and Authorization Survey conducted in 2016, the STM Association and NISO have been convening conversations focused on how to improve the user experience and provide a more seamless access methodology, while also providing greater control and analytics.

This session explores the issues of accessibility, privacy, and security addressed through multi-stakeholder pilot projects. Three pilots will be showcased including:

P3 WAYF Pilot: demonstrates how institutional login credentials can be leveraged through streamlined Where Are You From (WAYF) UI flows whilst preserving user privacy.

WAYF Cloud Pilot: validates the use of a cloud service to facilitate the exchange of information between publisher platforms for the purpose of Identity Provider discovery.

Corporate Pilot: demonstrates how the use of (Pharma-Documentation-Ring) P-D-R companies' corporate login credentials can be leveraged to provide seamless access to scholarly resources.

**Results:** Feedback from user surveys and early recommendations emerging from the three pilot results will be shared. Results are being collected now and focus on guidance around the user experience as well as best

practices for the security and privacy of user data. The final results of this project will be a set of best practice guidelines based on the real-world experienced developed through the pilots.

**Conclusions:** The pilots themselves may or may not be deployed further after the conclusion of the initial project depending upon the results obtained.

**Keywords:** Access, Privacy, Identity Management, Authentication, Security, Analytics

Sunday, May 20, 2018, 3:00 PM – 4:25 PM

Room: Embassy D (International Tower, Exhibit Level)

---

# Session: Leadership and Management 1 (LM-1)

## Moderator: Eugenia Liu

### Examining the State of Health Sciences Librarianship in Nigeria

**Biliamin Oladele Popoola**
Systems/Evidence-Based Medicine Librarian, University of Medical Sciences, Ondo City, Ondo State, Nigeria, Ondo City, Ondo, Nigeria

**Ngozi Celestina Uzoagba**
Senior Librarian, Medical Library,College of Medicine,University of Nigeria,Nsukka., Enugu, Enugu, Nigeria

**Nafisa Rabiu**
Assistant Lecturer, Department of Library and Information Science, University of Ilorin, Ilorin, Kwara State, Ilorin, Kwara, Nigeria

**Objectives:** For the past two decades or more, there has been a gap in literature concerning the state of health science librarianship in Nigeria. This study examined the various aspects of service delivery in Nigerian medical libraries. The aim of the study is to highlight the health science library services, challenges and prospects in the country.

**Methods:** The study adopted a descriptive survey design. Two research instruments were used for data collection. The first research instrument, a questionnaire, was designed using Google Form and was deployed through the Nigeria Library Association Listserv and the Nigerian Medical Library Association (MLA-NG) WhatsApp Group to capture responses from all health science librarians in the country. The second instrument was an interview conducted with selected Heads of medical libraries in the country to obtain qualitative data on the prospects and challenges of Nigeria’s health science librarianship. Using the questionnaire, the study covered all health science libraries in Nigeria, with responses from 58 librarians from the six Geo-political zones in the country. The interview was conducted with Heads of the medical libraries in the five first-generation universities in Nigeria, because of the vastness of the institutions’ resources and experience.

**Results:** Findings from the study showed the type of classification scheme being used in Nigerian health science libraries, librarians’ perception on the level of users’ understanding and patronage of the libraries, the use of social media for service delivery, the availability of specialized services for healthcare practitioners and training for users, librarians’ self-appraised knowledge of Evidence-Based Medicine and systematic reviewing, librarians’ access to specialized training in health science librarianship, and the prospects and challenges of practicing as health science librarians in the country.

**Keywords:** Health science librarianship, Nigeria, medical libraries, medical library services

## Forecasting Science: Identification of Research Needs through Collaboration

**Kathleen B. Oliver**
Management Consultant, National Institutes of Health Library, Chevy Chase, Maryland

**Keith Cogdill, AHIP**
Director, NIH Library, Bethesda, Maryland

**James King**
Branch Chief, NIH Library, Bethesda, Maryland

**Lynn Young**
Bioinformaticist, NIH Library, Bethesda, Maryland

**Objectives:** The library and other scientific resource divisions at a major biomedical research organization collaborated with its research scientists to anticipate changes in the practice of biomedical research and to adjust the portfolio of services provided in support of research.

**Methods:** Data collection included a combination of qualitative and quantitative methods. The target population included both early career and senior laboratory-based and clinical scientists in six emerging areas of research identified in the 2017 institutional long range research plan. Qualitative components included interviews of senior scientists and focus groups of early career scientists from each of the six research areas. Qualitative findings informed the development of a survey targeting a population of over 4,100 trainees, tenure-track and tenured laboratory-based and clinical research scientists. Participants provided feedback on library services, veterinary services, radiation safety, laboratory safety, scientific equipment and medical arts.

**Results:** Several themes emerged from qualitative phases: 1) the importance of facilitating collaboration as interdisciplinary nature of science grows; 2) a lack of service awareness; 3) the importance of current technology infrastructure for genomics; 4) a clear need for bioinformatics and computational support at several levels. Concern about an organizational structure and policy implementation that complicates and impedes research (5), and the need for capital investments for emerging research (6) were among other, broader themes emerging from the qualitative phases. Quantitative results are pending. An analysis of both qualitative and quantitative results will be described at the time of the presentation.

**Conclusions:** Conclusions will be described at the time of the presentation.

**Keywords:** Forecasting; Biomedical Research; Libraries, Medical; Radiation; Laboratories; Safety; Scientific Equipment.

## HINARI: The Ghana Grant

**Karin Saric**
Information Services Librarian, Norris Medical Library / Research & Instruction Services, Los Angeles, California

**Objectives:** To report outcomes from three Hinari workshops held in Ghana in March 2017 that support use of Hinari by allied health user groups and certified local trainers across 19 Ghanaian institutions. These workshops were funded by the MLA/HINARI/Research4Life (R4L) Activities Grant.

**Methods:** Hinari is a World Health Organization program that provides free or low cost access to peer-reviewed journals to institutions in developing countries. Elsevier recently expanded their MLA/LWB grant to provide

funding for librarian training activities. This paper discusses the outcomes of three workshops held in Ghana, funded by the Elsevier grant: 1) A pre-departure training/in-country support for occupational therapy externship students to prepare them to train local students/professionals; 2) in-country workshops for allied health faculty/students to introduce Evidence-based practice, database searching, and demonstrate the value of Hinari for institutions with limited funding; and 3) a 3-day Hinari regional training for health care professionals from eligible/registered institutions in Ghana. The author will discuss tailoring of instructional material for these specific audiences, sustainability for future workshops, as well as elements for writing a successful grant and tips for coordinating Hinari in-country workshops.

**Results:** 110 health professionals received training on how to use Hinari, and on how to train others within their respective institutions/disciplines. Participants included every type of Hinari user from those with linkages to developing countries, to in-country health professionals from nineteen of the country's health institutions. From conversations with the participants, it was evident that they were all leaders in their field. Per survey results, the workshops were highly relevant, well received, and there was clear acknowledgment of the value that the knowledge gained would bring to their work. Per 6-month follow-up participants have already held additional workshops at their institutions.

**Conclusions:** There is constant need to provide training to support use of Hinari resources. These workshops fulfilled the grant requirement of training specific groups of end-users and raising awareness across key institutions. Tailored material has been shared permanently online and with several universities. Outcomes were presented at the Occupational Therapy Africa Regional Group conference that was attended by therapists from many African countries. The online training material was shared with this group, and resulted in a request to conduct a tailored training at the University of Zimbabwe, further extending use of the new training material for allied health professionals

**Keywords:** Hinari, global health, database searching, EBM/EBP, training, grant, Ghana

## Information Science Solutions to Communicating Public Health Research Findings to the Public

**Caitlin Bakker, AHIP**
Research Services Librarian, Bio-Medical Library, Minneapolis, Minnesota

**Shanda Hunt**
Public Health Liaison & Data Curation Specialist, Health Sciences Library, Minneapolis, Minnesota

**Objectives:** We conducted a needs assessment of public health researchers as part of a multi-institutional study led by Ithaka S+R November 2016 - January 2017. The aims of the study were to capture the evolving needs, opportunities, and challenges of public health researchers in the current environment and provide actionable recommendations.

**Methods:** This study reports on the data collected at one site. Participants (N=24) were recruited through convenience sampling, and one-on-one interviews were audio recorded. Recordings were sent to a professional transcription service. Qualitative analyses were conducted using NVivo 11 Pro and were based on the principles of grounded theory. Both authors independently coded four interviews using line-by-line open coding, after which coding agreement was assessed and a coding scheme developed. Transcripts and field notes were independently coded by authors. The two NVivo databases were merged, which showed great accordance between coders. The authors pulled emergent themes from the data.

**Results:** The data revealed that researchers recognized the need to communicate the significance of public health research findings to the public, yet felt they lacked the skills and resources necessary. Many researchers questioned the value of making articles, research data, and other outputs openly available. They expressed their

frustration in trying to make complex data sets and research findings easily digestible by broad audiences. Finally, they did not make the connection between their professional marketing activities and dissemination.

**Conclusions:** Information professionals can assist public health researchers in modernizing and broadening their dissemination practices by considering alternative forums, such as repositories and open education resources, and by utilizing formats, such as data visualizations, that more effectively convey research findings. Libraries can assist in the creation of plain language summaries of research outputs and introduce researchers to the ways in which social media is being used to communicate to the public. These strategies could advance public health communication to the public, practitioners, and policymakers, as well as contribute to open science.

**Keywords:** public health
dissemination of research
academic research libraries

Sunday, May 20, 2018, 3:00 PM – 4:25 PM

Room: Embassy E (International Tower, Exhibit Level)

---

# Session: Lightning Talks 1

### Moderator: Keydi Boss O'Hagan, AHIP

## Transform Data into a Visual Story

**Terri Gotschall**
Scholarly Communications Librarian, University of Central Florida, Orlando, Florida

**Objectives :** To demonstrate how visual creation tools can transform data in to a useful more easily understandable visual. Data is needed to make informed decisions but numbers don't always tell the full story. The audience is left to figure out the connections between data points. You can help your audience recall important details by turning raw data in to a visual.

**Methods :** A live demonstration of visual creation tools such as Powerpoint's SmartArt, Google Spreadsheet, Excel's Quick Charts to help transform data into a visual aid quickly without the use of specialized software.

**Results :** Participants will know how to convert data to a visual quickly using tools they already have.

**Conclusion :** Libraries collect an enormous amount of data that can be used to help leaders make informed decisions. Visual aids present data in a format that makes it easier to understand trends and connections.

**Keywords :** visual aid, data, graphic, presentation

## Ready Reference Training for Public Access Services

**Tiffney A. Gipson**
Head of Collection Development, Kornhauser Health Sciences Library, Louisville, Kentucky

**Lauren E. Robinson**
Emerging Technologies Librarian, Kornhauser Health Sciences Library, Louisville, Kentucky

**Jessica Petrey**
Clinical Librarian, Kornhauser Health Sciences Library, Louisville, Kentucky

**Objectives:** Purpose: The paper examines the implementation of a ready reference training program for public service staff. The program was envisioned to educated staff in anticipation of dwindling traditional reference desk service hours. The hope of this project was to increase foundational knowledge of public services staff, improve point of need response, and decrease utilization of on-call librarians for basic questions.

**Methods:** Setting/Participants: The Kornhauser Health Sciences Library at the University of Louisville developed a training program to equip 10 public access staff with ready reference, website, and electronic resource skills.

Brief Description: The training was administered in two separate 90 minute sessions according to public access staff schedules. One collections librarian, one clinical services librarian, and one technology librarian gave instruction on utilizing the newly redesigned website, efficient use of the library catalog, and decision making between the most frequently utilized library resources. In addition, downloading mobile applications and troubleshooting access issues were discussed. This paper describes the planning process, development, and challenges encountered.

**Results:** Results: After the implementation of this program, the librarians chose to develop a private LibGuide for convenient access to materials addressed in the training sessions. This LibGuide has been made to automatically open on startup. Further training sessions will also be offered and expanded to student workers as well as new employees.

**Conclusions:** Conclusion: The public access staff have been very receptive to the training and have improved confidence in utilizing library resources. We hope to continue this program and report metrics at a future date.

**Keywords:** staff instruction, cross training, ready reference, circulation, public access

## Precision Medicine Tool Kit

**Marisa L. Conte**
Assistant Director, Research and Informatics, Taubman Health Sciences Library, Ann Arbor, Michigan

**Patricia F. Anderson**
Emerging Technologies Informationist, Taubman Health Sciences Library, Ann Arbor, Michigan

**Objectives:** Precision medicine is simultaneously compelling and challenging, requiring integration of millions of data points from disparate sources. Informationists at the University of Michigan's Taubman Health Sciences Library developed a toolkit tailored to data layers of a commonly accepted precision medicine model. This toolkit is both a resource for researchers engaged in precision medicine, and a tool for community engagement, facilitating the NIH vision of participating-driven research.

**Methods:** The National Institutes of Health’s Precision Medicine Initiative (PMI) defines precision medicine as, "an emerging approach for disease treatment and prevention that takes into account individual variability in genes, environment, and lifestyle for each person." Recently, the University of Michigan launched Precision Health, an interdisciplinary research initiative focused on discovery, development implementation of validated treatments and tools for prevention.

The toolkit was a multi-phase project. Initial steps included: reviewing and summarizing institutional, state, and national priorities for precision medicine initiatives, meeting with institutional stakeholders; and conducting a comprehensive literature review and environmental scan to identify both a thorough precision medicine model and relevant resources for specific "layers" of information. Additional project phases include promoting the toolkit and its resources through targeted outreach to both researchers and the community, and developing and presenting workshops or other instructional materials on the model and specific resources.

**Results:** If accepted, we will provide results in February 2018

**Conclusions:** If accepted, we will provide results in February 2018

**Keywords:** precision medicine, learning health systems, genomics, microbiome, personal health data

## Talking ORCID

**Merle Rosenzweig**
Informationist, Taubman Health Sciences Library, Ann Arbor, Michigan

**Objectives:** On October 16, 2012, the ORCID (Open Researcher and Contributor ID) initiative was officially launched and began to issue its unique idenfiers. In December of 2012, our library signed a "Basic" member license agreement with ORCID making us an earlier adaptor. This paper will cover our implantation of ORCID into our online systems and across our three campuses.

**Methods:** The "Basic" membership allowed us to create ORCID records for our faculty, librarians, and researchers;
deposit to existing ORCID records; and employ various APIs (Application Program Interfaces) along with the data that those APIs can access to perform tasks associated with an ORCID. In December of 2016 we renewed or ORCID contract through the Big Ten Academic Alliance (BTAA). The consortia contract gives us "Premium" level membership allowing us, among other things, to: integrating ORCIDs into multiple systems; use ORCIDs in vendor-hosted systems and separate internal systems and; run analytics of the interactions between our systems and ORCID.

**Results:** The implementation of ORCID began with assigning the ID to all two hundred of our librarians. Following that, our librarians worked with their liaison departments to have faculty and researchers register for an ORCID. We have set up an ORCID Research Identifier Management System for registering for an ID which than adds it to our campus wide directory. Our institutional repositories for documents and data have fields for ORCID for each deposit that is made. Both our doctoral and master students are required to register for an ORCID when they reach candidacy.

**Conclusions:** Institutions are at various stages of implementing the assignment of ORCIDs for their researchers. Funding agencies are using ORCID in the grant process and publishers have integrated it in their online manuscript submission so with an ID our researchers are able to easily manage these systems. By implementing ORCID at our institution our authors and researchers are able get credit for their research out put and distinguish themselves in the global research community.

**Keywords:** ORCID, Open Researcher and Contributor ID, Implementation

## Expanding Question Safari: Reaching New Frontiers

**Emily S. Lawson, AHIP**
Clinical Librarian, Children's Healthcare of Atlanta, Atlanta, Georgia

**Kate Daniels, AHIP**
Clinical Librarian, Fran Golding Medical Library, Children's Healthcare of Atlanta, Atlanta, Georgia

**Objectives:** To provide an update of a clinical librarian service, Question Safari, since its expansion to other campuses and nursing units. To re-measure its impact on nursing practice and the libraries' continued collaboration with the Nursing Research department.

**Methods:** In MLA '15 in Austin, a poster (#190) was presented on Question Safari (QS), a librarian rounding service that captures clinical queries in real-time from nurses and allied health professionals. Since then, the service was expanded to include another Children's hospital campus. To provide an update on the service, a 6-question user survey was sent out to gauge if QS was still considered a valuable service. Survey questions included how frequently they participated, did the information received impact their clinical practice, and did their question led to an EBP or research project of their own.

**Results:** A revised user survey was conducted to evaluate the service's continued usefulness and relevance (the last survey was conducted in 2015). It was sent to 94 QS participants with a 25% response rate. 91% of respondents found that having clinical questions collected at point of care (POC) was valuable. 91% found that the articles and/or literature links provided by the Clinical Librarian were useful in caring for their patients. 30% agreed that the information they received led them to pursue a Quality Improvement (QI), Evidence-Based Practice (EBP), or original research project.

**Conclusions:** Through the QS service, 240 questions were collected from nurses at POC from Aug 2014 - present. QS has proven to be a popular, low-cost outreach program, increasing awareness of library services, improving collaboration with the Nursing Research department, and providing the librarians with a deeper understanding of what their users need through context and increased communication. All information requests are collected and reviewed by librarians for trending topics and potential education needs. Through sharing findings via article publication (Lawson, 2016, MRSQ) and presentations at various professional association meetings, QS became a model for another hospital library to initiate their own research rounding service that resulted in further article publication (Thomas, 2017, MRSQ).

**Keywords:** Clinical librarian services; hospital libraries; nursing; patient care; nursing research, pediatrics

## An Experiment to Address Staffing Fluctuations by Insourcing Professional Librarians

**Anna Beth Morgan, AHIP**
Executive Director, Mayo Clinic Libraries and Historical Units, Rochester, Minnesota

**Objectives:** "Insourcing" is a term coined to explain Mayo Clinic Libraries' practice of hiring supplemental librarians to address periodic upswings in patron demands for literature searches and systematic reviews. Insourcing is also used to address the predicament of providing replacement professional librarians when full-time librarians are absent or away on leave. Although limited use of supplemental librarians has been a staffing practice at Mayo Clinic for several years, strategic insourcing has been a new staffing alternative since July 2017.

**Methods:** At the beginning of 2017, two supplemental librarians were already employed by Mayo Clinic, one in Arizona and one in Minnesota. During Spring 2017, two additional supplemental librarians were recruited when the practice of insourcing became a strategic initiative for addressing library staffing concerns. Now, a total of four experienced medical librarians are employed by Mayo Clinic as supplemental librarians. Of the four librarians, one is physically located in Phoenix, Arizona, two are located in Rochester, Minnesota, and one teleworker lives in California. The three supplemental librarians living in Arizona and Minnesota assist with literature searching services and onsite library staffing. The teleworker in California focuses on providing literature searching services. Like full-time librarians, each of the supplemental librarians serve the patrons of the Mayo Clinic Libraries which are located in four U.S. states (Arizona, Florida, Minnesota and Wisconsin).

**Results:** Insourcing is a viable option for providing additional staffing for libraries. The practice of insourcing professional medical librarians has proved to be a successful and cost-effective staffing technique. Insourcing

remedies the unpredictable need to provide additional professional labor upon demand and helps acquire additional known, oriented and skilled labor quickly.

**Conclusions:** While fiscal evaluations show insourcing to be a cost-effective and valued staffing alternative, insourcing has also eased staffing concerns and improved satisfaction among other library employees.

**Keywords:** Personnel Management, Literature Searching, Insourcing, Library Staffing, Cost Control, Staffing Enhancement

## Leveraging the Librarian in a Nurse Residency Program

**Olivia Glotfelty**
Librarian, The Brady Library of the Health Sciences- UPMC Mercy, Pittsburgh, Pennsylvania

**Objectives:** To demonstrate how the librarian can be utilized in, and contribute to, a Nurse Residency Program. To demonstrate how participation in a Nurse Residency Program can highlight library services and resources. To demonstrate how participation in a Nurse Residency Program can change participants' perceptions of the library.

**Methods:** The Nurse Residency Program will take place at a 495-bed tertiary acute-care teaching hospital. The program is a year-long, mandatory requirement for all newly-hired graduate nurses, which culminates in a completed evidence-based practice (EBP) project. The librarian will develop and teach several (2-3) librarian-led sessions throughout the program, covering topics such as EBP, library resources and services, literature search strategies and writing a literature review, and abstract and poster development. During these sessions, a pre and post-survey will be given out to measure improvement and changes in knowledge and attitudes of the participants. The data collected from the surveys will be entered into a program and analyzed. These steps will be repeated for each of the 4 cohorts completing the first implementation of the Nurse Residency Program.

**Results:** A pre and post-survey was used to evaluate and determine the impact of the sessions. After the librarian-led sessions, the mean knowledge of EBP increased, participants were able to more accurately identify EBP resources (Google vs PubMed), knowledge regarding the library location and resources improved, attitudes regarding EBP improved, and attitudes about the library improved. For example, in the pre-survey, a word commonly associated with the library was dusty. In the post-survey, the word was research. There has also been an increase in library usage from the nursing staff, including program facilitators, who are part of this program.

**Conclusions:** Medical librarians have valuable skills to contribute to a Nurse Residency Program, including an in-depth knowledge of clinical information resources, knowledge of research processes and methodologies, creative thinking, and teaching skills. The library is a place that can bridge the gap between research and practice, and librarians have been identified as effective partners in training healthcare personnel in evidence-based practice. By offering time and expertise, the librarian can highlight library services and resources, position the library as an authority and resource on evidence-based practice procedures, resources, and research skills, and change the way people view the library. Librarians should be included as stakeholders and participants in their institution's Nurse Residency Program.

**Keywords:** Residents, Nursing, Evidence-Based Practice, Librarian, Hospital

## Connecting the Dots between Evidence-Based Practice and Shared Decision Making: Growing Medical Students' Information Skills

**Nicole Capdarest-Arest, AHIP**
Head, Blaisdell Medical Library, University of California, Davis, Sacramento, California

**Henry C. Lee**
Associate Professor, Department of Pediatrics, Division of Neonatal & Developmental Medicine, Lane Medical Library, Stanford, California

**Objectives:** Though evidence-based practice (EBP) and shared decision-making (SDM) are taught in medical school curricula, their interdependence is not always made explicit and they are seldom taught in concurrence. Given students' expressed desire to share evidence found with patients, along with required Core Entrustable Professional Activities for Entering Residency, the authors desired to explicitly connect the two frameworks in instructional activities.

**Methods:** Students participate in four information fluency/EBP instructional sessions during the course of their medical school curriculum at Stanford University School of Medicine. Based on existing research, student feedback and continuous quality improvement of instructional activities, the authors revised the third and fourth of these sessions to introduce the interdependence of SDM with EBP. Sessions were adjusted to include components wherein sharing evidence found with patients was made explicit and practiced by the students in simulated scenarios. In the third session, students role-played evidence sharing as part of a case-based exercise, and in the fourth session, which occurs during clerkships, students practiced framing a SDM discussion around evidence found using the context of a real case they had encountered. Student work and comments on the sessions were reviewed as part of ongoing formative feedback.

**Results:** Students improved in comfort and skill level in framing EBP/SDM discussions. Discussions were more complex and reflected nuances regarding patient/family values and preferences. Opportunities to practice and discuss complexities of sharing evidence with patients and families was valued by most students.

**Conclusions:** Patients/families are increasingly information seeking and have opinions prior to doctor-patient discussions, so understanding where they are coming from can inform medical students' searches for evidence and information sharing. Students need continual practice with information seeking skills, so frequent reminders during various stages of their studies and ongoing is important. In particular, students need guidance on distinguishing good sources of information in terms of background, foreground, and patient information tools. Students progressed in comfort and skill level in information seeking and framing EBP/SDM discussions when having multiple opportunities to practice such skills across the course of their studies.

**Keywords:** Evidence-based practice; Shared decision-making; Medical education; Physician-patient relations; Information fluency

## Creating "Wizards" on the Library Website: Using a Decision Tree Model to Assist Virtual Users to Find Their Answers

**Berika Williams**
Emerging Technologies and Web Librarian, Hirsh Health Science Library, Boston, Massachusetts

**Debra R. Berlanstein, AHIP**
Associate Director, Hirsh Health Science Library, Boston, Massachusetts

**Objectives:** The library website is the virtual front door to the variety of services we offer. We found confusion arose focused on two particular areas: how to reserve rooms in the library and how to reuse images using best practices. Two interactive forms were created that would hide and reveal content based on choices and lead the patron to answers.

**Methods:** Brainstorming meetings were held to create a flow chart that identified the specific questions to be answered that would lead the user to a logical answer. Once the correct flow was created, we approached each of these challenges by creating a decision tree custom module using code through Drupal's Form API. Each is an interactive form, hiding and revealing content based on options the user chooses. For example, the room reservation wizard begins with a question asking who you are: student, faculty, or staff and moves forward based on your response. The image reuse wizard begins by asking if you already have an image to use or wish to find one online. By answering the series of questions the user is led to an actual answer. If a more individualized answer is required, a contact form is provided.

**Results:** The image reuse decision tree went live on the library website on February 17, 2017, and the room reservation wizard went live on August 27, 2017. In the short six weeks that the room reservation wizard has been available, it has been accessed 192 times and risen to #11 on the list of top accessed website pages. In the seven months that the image reuse wizard has been available it has been accessed 85 times.

**Conclusions:** The popularity of both the decision tree pages is exciting. We will monitor the statistics on the use of both pages, and their success will inform whether there are other common questions that would benefit from creating a similar decision tree form in the future. We will continue to look for creative ways to assist patrons that visit us virtually in getting the answers to their questions. We see this as a companion to our searchable knowledge base available with our Ask Us virtual reference service.

**Keywords:** decision tree, drupal, virtual, help, wizard

## Clinical Informationist Phoenix Rises to Deliver Quality Library Service for Virtua

**Helen-Ann Brown Epstein, AHIP, FMLA**
Informationist, Health Sciences Library, Mt Laurel, New Jersey

**Objectives:** After 22 years in an outstanding academic medical center, this Clinical Librarian rises and shines under the Virtua star to join the Department of Clinical Research on the Clinical Learning Team. A .5 FTE slot, and an unstructured intranet page became 1 FTE, a well organized page, with an ambitious rounding schedule and participation on many systemwide committees and councils.

**Methods:** This is a mixed method study of the Library's progress in the almost 2 years of the Informationist being on board. An online and paper version of the Library Use Survey was distributed. Statistics of searches run, ILLs requested and filled have been gathered. Testimonials from customers have been collected. Hours spent on Information HouseCalls, as an active Magnet Champion, a member of the Nursing Research Council, a member of the IRB, and an attendee at many important hospital gatherings dealing with evidence based practice, shared governance and relationship based care to get information requests or perceive information needs have been counted. Business has been brisk and very rewarding.

**Results:** 119 responded to the Library Use Survey. Nurses are the largest user group. 37% of the respondents use the library once a week and 30% use it at least once a month. 14% have not used the library. CINAHL, Lexicomp, and NRC+ are the most used resources. 27% of the respondents have placed an information request and 38% received an Information HouseCall. Primarily the information resources are used for patient care. The Informationist rounds with 32% of the respondents, who read abstracts or the papers pushed and have applied the information to their practice. Over 200 search requests and ILLs.

**Conclusions:** This experienced Clinical Librarian rises like a phoenix to be Virtua's Informationist. Her virtual library gives her the freedom to travel throughout the Virtua Health System and participate in cutting edge projects, be present to take information requests, listen carefully and perceive information needs of all of her customers in groups or one-on-one Information House Calls. By this outreach, she is improving the health of the

patients with quality consumer health information, further educating the staff and supporting significant clinical research.

**Keywords:** Informationist Virtual Library outreach

## Leading the Data Discovery Charge: A Cross-Institutional Collaboration to Index Research Data

**Kevin B. Read**
Knowledge Management Librarian, NYU Health Sciences Library, New York, New York

**Melissa Ratajeski, AHIP**
Coordinator of Data Services; IACUC Liaison, Health Sciences Library System, Pittsburgh, Pennsylvania

**Na Lin**
Head of Resource Development and Sharing, Resource Sharing, Baltimore, Maryland

**Nicole Contaxis**
Data Catalog Coordinator, NYU Health Sciences Library, New York, New York

**Ian Lamb**
Senior Developer / Solutions Management, NYU Health Sciences Library, New York, New York

**Alisa Surkis**
Assistant Director for Research Data and Metrics, NYU Health Sciences Library, New York, New York

**Meg Del Baglivo**
Metadata Librarian, Health Sciences and Human Services Library, Baltimore, Maryland

**Carrie L. Iwema, AHIP**
Coordinator of Basic Science Services, Health Sciences Library System, Pittsburgh, Pennsylvania

**Joel Marchewka**
Web Applications Programmer, Health Sciences Library System / Digital Library Services, Pittsburgh, Pennsylvania

**Jean-Paul Courneya**
Bioinformationist, Health Sciences and Human Services Library, Baltimore, Maryland

**Angela Zack**
Knowledge Integration Lead, Health Sciences Library System, Pittsburgh, Pennsylvania

**Patricia Hinegardner, AHIP**
Associate Director for Resources, Health Sciences and Human Services Library, Baltimore, Maryland

**Brad Gerhart**
Web Developer, Health Sciences and Human Services Library, Baltimore, Maryland

**Objectives:** To facilitate the discovery of biomedical research data that are otherwise not easily findable, three academic health sciences libraries implemented local instances of an open source data catalog (DC) as a means of indexing institutional research datasets. This collaboration brings a cross-institutional perspective to addressing issues of usability, data sharing workflows, metadata, and outreach for improving data discovery efforts.

**Methods:** Librarians and developers from each institution collaborate to develop strategies for (1) expanding outreach efforts to market the DC and establish internal/external partnerships, (2) improving DC usability based on researcher interactions and feedback, (3) addressing researchers' concerns about data sharing workflows, and (4) consulting with researchers from various disciplines to improve the DC metadata and ensure datasets are appropriately indexed. With each library focused on describing research from different disciplines and grappling with different institutional infrastructures, this collaboration serves to inform how an institutional DC can better serve the needs of a broad range of institutions. Routinely evaluating the collaboration's strengths, challenges, and common goals allows each library to successfully facilitate the discovery of biomedical research data at their institution. Future directions include adding new institutions to the existing collaboration, and aligning DC metadata with NIH data sharing initiatives.

**Keywords:** data discovery; data sharing; metadata; data collection; data access

## Transforming the Archives into a Celebration: Nurses' Week

**Mary Shah, AHIP**
Medical Librarian & Archivist, Horblit Health Sciences Library, Danbury, Connecticut

**Objectives:** The hospital archive's home is in the library and the archivist is also the nursing liaison librarian. Nurses are our biggest group of users, for which we enjoy finding ways to celebrate whenever possible. Nurses' Week is a perfect time to not only celebrate the past, but to join those on the floor today with those who served before them.

**Methods:** Through work on the Practice Council and Research Council, the library connects with the nurses in the units. Through the oral history project, the archive has made strong connections with the hospital school of nursing alumnae association. The celebration of Nurses' Week is a wonderful opportunity to transform the history of nursing into a vibrant display—not only celebrating our past, but advocating for the archive and the library as well as nursing.

With each reunion of the alumnae association, the archivist brings mementos to help the retirees recall and celebrate their past. Photos have been scanned and turned into a music video. This inspires the trust of the retirees and then they donate or loan their things related to their schooling and work at the hospital.

**Results:** Through having the displays during Nurses' week at their events and the main lobby of the hospital, there has been an increased awareness of the archive and the library. The nurses continue to be the biggest group of users at the library. There are now two large display cases in the library, containing the nurses' donations and more donations from other staff members have increased as well. The facilities department is now working with an architect to create a permanent display of the archive in one of the lobbies of the hospital.

**Conclusions:** In transforming the archive into a vibrant celebration, the partnership between the present staff and those who preceded them is even stronger. Future opportunities are in finding innovative ways to connect the past to technology, enhancing its attractiveness to a newer generation of users as well as increasing access to the materials for future research and celebrations.

**Keywords:** Archive, nursing, history, transforming, technology, display, oral history, partnership

Sunday, May 20, 2018, 3:00 PM – 4:25 PM
Room: Embassy B (International Tower, Exhibit Level)

---

# Special Content Session: Transforming Libraries Using Implicit Bias Training

### Moderators: Shannon D. Jones, AHIP, Kelsa Bartley

## Transforming Libraries Using Implicit Bias Training

**Kimberly L. Reynolds**
Pediatrician, Miller School of Medicine, University of Miami, Miami, Florida

**Shannon D. Jones, AHIP**
Director of Libraries, Medical University of South Carolina, Charleston, South Carolina

**Program Description:** This session will provide attendees with an overview of implicit bias, the impact it has in libraries and in health care, and more importantly how it stands in the way of diversity and inclusion. An implicit bias occurs when we have attitudes towards people or associate stereotypes with them without our conscious knowledge. The invited speaker for this session will define implicit bias, provide examples of what it looks like in the workplace, and offer strategies that librarians may use to identify and overcome their own biases.

**Keywords:** implicit bias, unconscious bias, diversity, inclusion

Sunday, May 20, 2018, 3:00 PM – 4:25 PM
Room: Embassy A (International Tower, Exhibit Level)

---

# Special Content Session: Research Roadmap: Understanding the Research Process

**Moderator: Kimberly R. Powell**

## Research Roadmap: Understanding the Research Process

**Kristine Alpi, AHIP**
Director, William Rand Kenan, Jr. Library of Veterinary Medicine, Raleigh, North Carolina

**Jonathan D. Eldredge, AHIP**
Associate Professor, Medical library, Albuquerque, New Mexico

**Heather N. Holmes, AHIP**
Associate Director of Libraries, Medical University of South Carolina, Charleston, South Carolina

**Katherine G. Akers**
Biomedical Research and Data Specialist, Shiffman Medical Library, Detroit, Michigan

**Program Description:** Research has grown into an integral activity for all types of institutions, and we as a profession increasingly recognize the role of librarians as researchers. While many librarians are interested in getting involved in research to better understand and address complex questions, knowing where and how to start is challenging. With or without institutional support, research can be a daunting prospect. Join this session to learn the basics of getting started and how to travel down the road to research success. Participants in this session will review the research process and plan for:

- Turning daily work into a research project
- Identifying and overcoming obstacles to complete your project
- Choosing the research method that best fits your project
- Using technology and data collection techniques that best fit your study design
- Disseminating your research as a poster, presentation, or paper
- Understanding the publishing process

**Keywords:** Research, Hospital librarians, New librarians, Mentorship, Collaboration, Publishing

Sunday, May 20, 2018, 3:00 PM – 4:25 PM
Room: International Ballroom South (International Tower, Ballroom Level)

---

# Special Content Session: Hospital Libraries: Looking Backward to Stimulate Forward Momentum

**Moderator: Alice Jaggers**

## Hospital Libraries: Looking Backward to Stimulate Forward Momentum

**Basia Delawska-Elliott, AHIP**
Medical Librarian, Providence St. Joseph Health, Portland, Oregon

**Sandra G. Franklin, AHIP, FMLA**
Director, Woodruff Health Sciences Center Library, Atlanta, Georgia

**CeCe Railey, AHIP**
Clinical Librarian, University of Louisville , Kornhauser Health Sciences Library, Louisville, Kentucky

**Diane G. Schwartz, FMLA**
Research Associate Professor, University at Buffalo, SUNY, Buffalo, New York

**Rebecca Morgan, AHIP**
Hospital Librarian, University of Louisville Hospital, Rowntree Medical Library, Louisville, Kentucky

**Vida M. Vaughn**
Assistant Director, Kornhauser Health Sciences Library, Louisville, Kentucky

**Ellen Aaronson, AHIP**
Librarian, Mayo Clinic Libraries, Rochester, Minnesota

**Kathy Zeblisky, AHIP**

**Program Description:** Despite our profession's constant evolution to address the changing needs of our users, many health sciences libraries, particularly hospital libraries, are under scrutiny by their institutions. Many of these libraries are facing closure. In fact, a recent study of health sciences library closings [Schwartz, Elkin 2017] found that from 1989-2006 approximately 23-34% of US health sciences libraries closed. Furthermore, between 2011 and 2015, 613 libraries closed, for an average of 115 closings per year [Thibodeau, Funk 2009]. But why? What was the rationale for these closings? Was the decision based solely on economic reasons, or were there other underlying motives? How will providers who lack library services fill their information needs? How can we prevent the closing of additional libraries? This Special Content Session will focus on the ongoing

challenges faced by health sciences libraries to remain relevant in an ever-changing world. A panel of speakers will share their experiences with library closures and strategies and best practices for remaining vigilant and promoting library staff, resources, and services effectively. The panel will be followed by an open forum discussion of health sciences library closings in general.

**Sponsors:** This session is co-sponsored by the Outreach & Marketing SIG, Hospital Libraries Section, and the Leadership & Management Section.

**Keywords:** hospital libraries, library closures, marketing, institutional change, strategic planning

*Kathy Zeblisky, AHIP will be presenting on behalf of Ellen Aaronson, AHIP.*

Sunday, May 20, 2018, 3:00 PM – 4:25 PM
Room: International Ballroom North (International Tower, Ballroom Level)

---

# Special Content Session: My Favorite Tool

**Moderators: Margaret A. Hoogland, AHIP, Erin D. Foster, Christine Andresen, Jennifer Herron**

## My Favorite Tool

**Margaret A. Hoogland, AHIP**
Clinical Medical Librarian, Mulford Health Sciences Library, The University of Toledo, Temperance, Michigan

**Erin D. Foster**
Data Services Librarian, Indiana University School of Medicine, Indianapolis, Indiana

**Christine Andresen**
Research and Education Informationist, Medical University of South Carolina, Charleston, South Carolina

**Molly Knapp, AHIP**
Training Development Specialist, NNLM Training Office, Houston, Texas

**Program Description:** Tools are a key component for how individuals, regardless of our role in an organization, accomplish tasks efficiently and effectively. What some consider a tool, however, others might consider a splurge or unnecessary expense.

**Recruitment/Publicity for the session**: EMTS will send out invitations to participate in “My Favorite Tool” Unconference Session. Invitations will include the questions, which each contestant should be able to answer in 3 minutes:

1) State your full name and then tell the audience and judges the name and purpose of the tool.

2) Tell the audience and judges how you used, encountered, or discovered the tool the first time.
3) Describe the best features of the tool (i.e compatibility, functionality)

4) Which features of the tool would you change (i.e. if I could change x feature, this is what I would do!)

5) You have no budget. Would you recommend using personal funds or writing a grant proposal to acquire this tool?

The first 20 contestants with unique tool(s) will be presenting during this session. Selection will take place in February and an option Orientation/Overview session will take place in April. Contestants are encouraged to email (mahoogla@gmail.com) with questions.

A judges panel and the audience will vote on the presentations by each of the 20 contestants.

3 Prizes ($50, $30, $20) will be awarded to the top 3 contestants courtesy of the Educational Media and Technology Section.

**General Session Outline:**

**Introduction:** (5 minutes - likely will mention EMTS events and Business Meeting, if applicable)

**Contestant Presentations:** 3 minutes to answer the 5 questions posted above. (~60 minutes)

**Voting:** Judges and Audience will have 1 minute to vote after the presentation concludes (~20 minutes)

**Awards:** Prizes are awarded and a picture is taken of the three winners and judges (~8-10 minutes)

Judges for this event will be Christine Andresen, Erin Foster, and Molly Knapp. Margaret Hoogland will serve as the facilitator and time keeper.

We are excited about this event and we hope you will consider attending!

Monday, May 21, 2018, 10:30 AM – 11:55 AM
Room: Embassy B (International Tower, Exhibit Level)

---

# Session: Clinical Support 1 (CS-1)

**Moderator: Edith Starbuck**

## "HEALWA": Increasing Awareness of a Statewide Health Information Resource for Health Professionals

**Kathryn Vela, AHIP**
Health Sciences Outreach Librarian, Spokane Academic Library, Spokane, Washington

**Christina Pryor**
Assistant Director and Community Health Education Coordinator, Health Sciences Library, Seattle, Washington

**Jonathan Potter**
Library Director, Spokane Academic Library, Spokane, Washington

**Objectives:** Providing outreach to busy nurses in rural areas can be challenging, due to their busy schedules and geographic isolation. To increase awareness of a statewide health information resource "HEALWA" among rural nurses, a monthly webinar series was developed to familiarize participants with the resource.

**Methods:** After attempts at frequent in-person workshops failed due to lack of attendance, an hour-long webinar was developed that would be delivered once a month. These webinars introduced participating nurses to HEALWA, including how to set up their access and how to navigate the resource. To accommodate the busy schedules and differing learning styles of nurses, the workshops occur both as a monthly webinar and in-person when available. Marketing of the webinars occurred via email, association websites and newsletters, social media, and word of mouth. In order to determine the success of these workshops, data was collected using registration forms and post-webinar evaluation surveys.

**Results:** The webinar series and occasional in-person workshops have been able to reach a combined 284 health professionals, primarily nurses, in the target region of the state over 12 months and has achieved largely positive results. In response to whether the workshop taught them a new skill that they plan to use in the future, 99% of respondents said yes. Additionally, 99% of respondents indicated that they plan to use at least one resource in HEALWA in the future.

**Conclusions:** The webinar series has been a successful outreach approach to nurses in rural portions of the state. To build on this success, there are plans to implement an additional series of workshops to further increase awareness of HEALWA and the resources available in the portal.

**Keywords:** Outreach, rural, health professionals, webinars, workshops, nurses

## Adapting Data Management Training to Support Clinical Research

**Kevin B. Read**
Knowledge Management Librarian, NYU Health Sciences Library, New York, New York

**Objectives:** Clinical researchers who do not participate in large research studies receive little to no training or support in data management. A librarian at an academic medical center worked to fill this gap by developing a clinical data management (CDM) curriculum to educate clinical investigators, assess the needs for advanced topics, and disseminate information about institutional CDM services.

**Methods:** Using skills developed from a CDM training course offered by the Society for Clinical Data Management, a librarian developed and taught CDM courses in collaboration with the institution's CDM support core who have significant expertise in this area. The curriculum included the following topics: building data dictionaries, using data standards, collecting clinical data, ensuring data quality/security, and developing research workflows. This curriculum also highlighted institutional CDM services offered by the library and the CDM core that were unfamiliar to researchers. The course was offered to junior faculty, residents conducting research, and faculty. Evaluation data will serve to elucidate additional topics of interest from researchers, and topics that require more advanced training courses. This data will be used by the librarian and CDM core to develop and co-teach new and advanced CDM topics in the library's summer 2018 data workshop series.

**Keywords:** clinical data management; clinical research; institutional collaboration; data management education

## Bringing an Academic Library to the Point of Care: The Reno Experience

**Alexander Lyubechansky**
Clinical Librarian, Assistant Professor, Savitt Medical Library, University of Nevada, Reno School of Medicine, Reno, Nevada

**Katie Jefferson**
Library Services Liaison, Savitt Medical Library, Reno, Nevada

**Michelle Henderson**
Administrative Director, Office of Research and Education, Reno, Nevada

**Mary Shultz**
Director, Savitt Medical Library, Reno, Nevada

**Objectives:** This paper presents a service model where an academic medical library was integrated into a community hospital.

**Methods:** Hospital libraries and hospital librarians have been found to be crucial in the provision of quality patient care. However, over time many hospital libraries have closed due to cost-saving measures and hospital mergers or acquisitions. The Savitt Medical Library (SML) serves the University of Nevada, Reno School of Medicine (UNR Med) which is a community-based medical school. As such, UNR Med does not own a teaching hospital. Instead it partners with community hospitals to provide the clinical settings for its learners (students, residents, and fellows). Renown Regional Medical Center (Renown) is the largest hospital in the state of Nevada and UNR Med's major hospital partner. In 2017, the Savitt Medical Library collaborated with Renown's Office of Clinical Research and Education to create a branch library at Renown's main hospital.

**Results:** The hospital provided the library space and SML provided staffing, technology, and print resources.

The Clinical Librarian and the Library Services Liaison offer a wide range of services at the point of care in support of clerkship and residency programs, faculty development, hospital clinicians, community faculty, and patient care. Due to licensing restrictions, SML promotes the use of many freely available quality resources to the hospital clinicians. At the same time, the creation of the branch library was a factor in Renown decision to expand its own collection of licensed resources to their clinicians who are unaffiliated with UNR Med.

**Conclusions:** Preliminary evaluation of this service model indicates that bringing library services to the point of care appears to be beneficial despite having its own challenges. The ultimate result has been that clinicians at Renown (UNR Med and non-UNR Med) now have access to professional library services and to resources. Although licensing of two sets of resources for two sets of users sharing a space provides challenges, the overall benefits have outweighed complications.

**Keywords:** Collaboration, Community Hospital, Partnership, Medical School, Medical Library, Clinicians

## Curating the Evidence: Medical Librarians Collaborate with Physicians to Create a Clinical Decision Support System

**Margaret (Margo) Coletti**
Knowledge Services Director, Beth Israel Deaconess Medical Center, Boston, Massachusetts

**Julia Whelan**
Information Specialist, Knowledge Services, Boston, Massachusetts

**David Osterbur**
Director of Public and Access Services, Countway Library of Medicine, Boston, Massachusetts

**Carol Mita**
Research & Education Librarian, Countway Library of Medicine, Boston, Massachusetts

**Jacqueline Cellini**
Reference and Education Services Librarian, Countway Library of Medicine, Boston, Massachusetts

**Paul Bain**
Reference and Education Services Librarian, Countway Library of Medicine, Boston, Massachusetts

**Meaghan Muir**
Manager, Library Services, Medical Library, Boston, Massachusetts

**Emily Schon, AHIP**
Clinical Librarian, Walter Reed National Military Medical Center, Bethesda, Maryland

**Jessica LaBrie**
Librarian, Librarian, Boston, Massachusetts

**Objectives:** The 2014 Protecting Access to Medicare Act (PAMA) requires integration of clinical decision support and appropriate use criteria into the EHR. To satisfy that rule, a small group of academic and hospital librarians, physicians, and programmers, have built the Harvard Library of Evidence (libraryofevidence.med.harvard.edu) to satisfy that requirement. This report is on the role librarians play and what the future holds.

**Methods:** Published guidelines, local best practices, and peer-reviewed literature are combed for pieces of clinical logic. Each piece of logic is broken down into multiple decision points ("rules") and assigned to two different Curators (medical librarians). Curators independently evaluate the strength of the evidence provided by the primary source or in studies cited by the primary source. Using ratings from the Oxford Centre for Evidence Based Medicine and the US Preventative Task Force (USPTF), the curators assign scores to the primary source or the studies cited by each rule. Physicians review Curators' ratings before the rules are finalized. The entire interprofessional group meets weekly to discuss rules, ratings and other concerns.

**Results:** The Library of Evidence will play a key role in integrating evidence ratings into mandated electronic health records, and in promoting appropriate use and cost-saving efforts. The Library will be free to all, and open source coding for the Library will be available to any EHR vendor or healthcare provider. In the future, the Library of Evidence will expand beyond the current scope and involve more medical librarians in the curation process.

**Conclusions:** This project affords participating medical librarians a literal seat at the table where they debate and defend their ratings among physicians and colleagues, and contribute to the design of the interface. The Curator role is an opportunity to utilize our skills in evaluating evidence-based medicine and to contribute directly to a project designed to benefit all patients and healthcare organizations. In the experience of the authors, the project has also served to broaden their base of knowledge in certain medical specialties (e.g., radiology) and in evidence-based medicine. Curators of medical evidence are leaders in a shift in our profession.

**Keywords:** Evidence Based Medicine,
EBM,
Librarian Roles,
Clinical Decision Support,
CDS, Harvard Library of Evidence

## Expanding Empathy, Discovering Diversity: A Librarian-Led Fiction Book Club for Health Care Employees

**Cait Kortuem**
Medical Librarian, Regions Hospital, St Paul, Minnesota

**Mary Wittenbreer**
Head Medical Librarian, Regions Hospital, St Paul, Minnesota

**Jennifer Feeken**
Medical Librarian, Regions Hospital, St Paul, Minnesota

**Objectives:** In light of local and national events, the medical library created an employee book club focused on highlighting diverse fiction and authors, increasing employees' empathy and wellbeing. The book club exposes healthcare employees to new stories, experiences, and authors, while creating a welcoming community for discussion and aligning with HealthPartners' system-wide goals of diversity, inclusion, equitable care, and employee wellbeing.

**Methods:** Based on recent research suggesting the benefits of reading, and the benefit of increased empathy from reading fiction in particular, we created a framework of one fiction book every two months, with options for online and in-person discussion to account for busy employee schedules. We selected platforms for our discussions (Goodreads, Global Meet), chose our first book (Americanah, by Chimamanda Ngozi Adichie) and pitched the project to organization stakeholders, including groups focused on diversity and employee resiliency. With stakeholder buy-in, we advertised through institution publications and a book giveaway. Librarians received

training on facilitating discussions from an internal Diversity and Inclusion Consultant, and facilitated three discussion sessions per book. When discussions were complete, employees were asked to fill out surveys and choose the next book from a short list compiled by the medical librarians.

**Results:** The employee book club is ongoing, but preliminary results are positive. Employees have read five different diverse fiction books, we have more than 100 members on Goodreads, attendance at our online and in-person discussions increases with each book, and we have received positive feedback through anonymous surveys and word of mouth.

**Conclusions:** The first year of the employee book club has been successful. We are continuing the program, with increased involvement from other HealthPartners groups and committees focused on diversity and inclusion.

**Keywords:** book club, empathy, diversity, fiction, reading

Monday, May 21, 2018, 10:30 AM – 11:55 AM
Room: Embassy E (International Tower, Exhibit Level)

---

# Session: Instruction and Instructional Design 2 (ID-2)

**Moderator: Merle Rosenzweig**

## Assessing Medical Students' Ability to Find and Evaluate Online Health Information Using the Research Readiness Self-Assessment (RRSA)

**LaVentra E. Danquah**
Librarian III, Service Coordinator for Library Instruction and Outreach, Vera P. Shiffman Medical Library, Wayne State University, Detroit, Michigan

**Katherine G. Akers**
Biomedical Research and Data Specialist, Shiffman Medical Library, Detroit, Michigan

**Ella Hu**
Biomedical Sciences Reference & Research Informationist, Shiffman Medical Library, Detroit, Michigan

**Sandra Martin, AHIP**
Director, Shiffman Medical Library, Detroit, Michigan

**Patricia Vinson**
PT Librarian 1, Shiffman Medical Library, Detroit, Michigan

**Wendy Wu**
Librarian IV, Shiffman Medical Library, Detroit, Michigan

**Objectives:** To provide the best patient care, physicians must be able to locate, critically evaluate, synthesize, and make clinical decisions based on medical information from multiple digital sources. Here, we describe our experience in administering the Research Readiness Self-Assessment (RRSA): Health Version to first-year medical students to evaluate their perceived and actual competencies in finding and evaluating online health information.

**Methods:** In collaboration with faculty in our School of Medicine’s Office of Learning and Teaching, our team of medical librarians assessed students’ perceived and actual competencies in finding and evaluating online health information using the RRSA: Health Version, a fee-based online assessment tool. First, we administered the RRSA to two cohorts of first-year medical students (class of 2020, n = 295; class of 2021, n = 287). We are now in the process of developing several online, self-paced, interaction learning modules aimed at improving students’ competencies in asking clinical questions, finding and using relevant information sources, and managing references. Later, we plan to re-administer the RRSA to the same two cohorts at the end of their

second year to determine whether our learning interventions enhanced students' ability to find and evaluate online health information.

**Results:** Initial RRSA results suggest that first-year medical students were better at evaluating online health information (% of points possible; class of 2020, 84%; class of 2021, 85%) than finding online health information (class of 2020, 70%; class of 2021, 79%). Students also showed a relative lack of previous research and library experience (class of 2020, 52%; class of 2021, 56%). To improve these competencies, we have so far developed two online learning modules: one on using Bloom's taxonomy to ask good clinical questions and the other on choosing the most appropriate type of information resource to answer particular health-related questions.

**Conclusions:** Our administration of the RRSA: Health Version to incoming medical students allowed us identify students' strengths and weaknesses in locating and appraising health information sources and has guided our development of new learning interventions to address specific gaps in their health information literacy. By supporting our School of Medicine's recent initiative to help medical students become life-long learners by incorporating self-directed learning experiences into the medical curriculum, this initiative has created more opportunities for contact between medical librarians and medical students and has strengthened the collaborative relationships between medical librarians and medical education faculty at our institution.

**Keywords:** health information literacy, medical education, clinical questions, library instruction

## Coverage of Core Competencies for Health Information Professionals in American Library Association Programs

**Kaitlin L. Costello**
Assistant Professor, Rutgers University, Department of Library and Information Science, New Brunswick, New Jersey

**Norris Brown**
Research Assistant, Department of Library and Information Science, New Brunswick, New Jersey

**Objectives:** What health-related courses are available to students in master's programs accredited by the American Library Association? Which of the core competencies necessary for work in health informatics, as defined by both the Medical Library Association and the American Medical Informatics Association, are covered in these classes? What curriculum gaps should instructors in these programs focus on for future course development?

**Methods:** A list of 60 accredited programs was obtained from the American Library Association directory in January 2017. During a six-month period from February 2017 through July 2017, the research team visited program websites and institutional course catalogs, and searched for course listings covering health-related topics between Fall 2014 and Fall 2017. Course descriptions and other related data (e.g., date of last offering, number of credits, whether the class was part of a larger concentration in health informatics or similar, last available syllabi) were collected when available. The Medical Library Association and American Medical Informatics Association competencies were discussed among the research team and six codes, each corresponding to one of the six objectives outlined by both frameworks, were created. Deductive quantitative content analysis was then applied to the available course descriptions to determine the main competency addressed in each class.

**Results:** Thirty-nine of the 60 schools offer at least one class focused on health. Nine programs offer a concentration in health informatics, while 18 of them offer one health class. Most (32) classes focus on health information services and health reference. Nineteen are related to the development of the health information professions; 16 cover leadership, management, and organizational communication; 12 address technical skills like data structures and programming; 8 are on instructional design and the design of sociotechnical systems;

and 5 focus on evidence-based medicine and fundamental theoretical frameworks. Programs with concentrations in health offer more comprehensive coverage of the competencies.

**Conclusions:** Students in these programs will often need to supplement the health-related course offerings with classes in programming, coding, instructional design, human-computer interaction, and theoretical frameworks in information science and health in order to gain the core competencies required for a career in health information. Instructors in these programs looking to develop classes in health information should focus on developing those that address competencies that are not currently addressed fully, including the design of instructional programs and of sociotechnical systems for health; theoretical frameworks in information science, evidence-based medicine, and health behavior; and on technical skills like structuring data and coding.

**Keywords:** health information professions; core competencies; curriculum development; health informatics

## Curricular Transformation Using an Instructional Design Model: A Medical Informatics Elective Course Case Study

**Emily M. Johnson, AHIP**
Regional Health Science Librarian & Assistant Professor, University of Illinois at Chicago, Peoria, Illinois

**Deborah L. Lauseng**
Regional Head Librarian & Assistant Professor, University of Illinois at Chicago, Library of the Health Sciences - Peoria, Peoria, Illinois

**Carmen Howard**
Regional Health Sciences Librarian & Instructor, Library of the Health Sciences - Peoria, Peoria, Illinois

**Objectives:** To address updated educational objectives, the librarian-taught “Survey of Medical Informatics” underwent a curricular transformation. Using an instructional design model, faculty librarians created and implemented a revision plan, which was based on best practices and focused on learner’s discipline-specific needs. This case study demonstrates the value of the model throughout the instructional design and implementation process.

**Methods:** The online credit-bearing elective within the College of Medicine has been offered since 2008. A new team of librarian-designers determined that a curricular transformation was necessary given multiple advancements in informatics and course design technology, as well as, student course feedback. To guide this process, the team utilized the Kemp Instructional Design Model. Kemp’s model is a learner-centered, non-linear, and iterative structure, with nine core instructional elements: learner characteristics, task analysis, objectives, sequencing, strategies, instructional delivery, message design, evaluation instruments, and instructional problems. The elements are interdependent, allowing a designer to start with any element and engage with multiple elements simultaneously to create instruction as an individual or a team. This model provided the team creativity and flexibility while utilizing a common structure and goal-driven plan to accomplish a new curriculum for this elective course within a one-year period.

**Results:** Using the Kemp model, the team successfully completed the curriculum transformation on schedule. Faculty revised five broad medical informatics topic areas and created twenty new lectures. Eleven new and six revised assessments target learners’ discipline-specific educational and professional needs. Significant changes included an expansion of big data, meaningful use, and eHealth topics, a reduction in coverage of database concepts, and the incorporation of personal reflection in the assessments. Additionally, all content was linked to competencies in informatics from professional organizations. The revised elective launched in June 2017 with no downtime, uninterrupted student enrollment, and continuation of the course evaluation requirement.

**Conclusions:** Kemp’s model helped the team, who had limited formal training in instructional design, manage a

major curricular transformation of the “Survey of Medical Informatics” elective for medical students. Throughout the design of the new curriculum, the team was able to use Kemp’s model to focus on the learner’s needs, preferences, and goals, while utilizing best instructional design practices. This case study shows that Kemp’s flexible instructional design model has application in other library instructional or curricular design projects, including informatics education in the health professions.

**Keywords:** instructional design models, informatics, curriculum, undergraduate medical education, learner centered

## Developing a Framework for Competency Integration across the Curriculum

**Amanda Chiplock, AHIP**
Senior Medical Librarian/Department Head, Wimberly Library / Medical and Health Sciences Collections & User Services Department, Boca Raton, Florida

**Elizabeth Gundersen**

**Jennifer Caceres**

**Mario Jacomino**

**Dawn Sherling**

Objective. Adapting to a new competency-based grading system requires developing a framework for competency integration within the current curricula. Integration of evidence-based medicine (EBM) with the basic sciences and clinical skills curricula allows students to learn and apply EBM skills with more relevancy to practice. Students will develop lifelong learning skills necessary to increase self-efficacy, decrease stress, and resist empathy decline.

Method. Using a triangulation approach (observation, review of student work, and lecture surveys), we identified the need for better integration of EBM in the first- and second-year, basic science and clinical skills courses to increase alignment with competencies. Through interdisciplinary collaboration and teamwork, we restructured EBM lectures to include online, self-directed learning modules to supplement face-to-face lectures and individual and team-based activities in the first-year clinical skills course. In the first-year basic science course, we integrated EBM skills with lifelong learning lectures to support case-based, small group sessions. For the second-year clinical skills course, we expanded a current, critical appraisal, practice-based assignment to include more reflection and consideration of patient values, concerns, and circumstances when making clinical decisions. This assignment serves as the course assessment for the Practice-Based Learning and Improvement competency and is a component of students’ longitudinal learning portfolios.

Results. Thus far, students' responses on the lecture surveys have been positive, overall, with scores for lecture content, delivery, and instructor improving from previous years’ EBM lectures (pre-integration). In addition, observation of the small group activities have been more positive than in previous years in regards to students’ abilities as a group to apply EBM skills to case-based activities with increased critical reflection. Overall, assessment scores indicate students learned and understood critical appraisal of a randomized controlled trial. An unanticipated outcome was that more Year-1 students sought EBM help than students in previous Year-1 classes.

Conclusion. We hope this method will serve as a framework for better integration of competencies across the curriculum. Based on observation, feedback on lecture surveys, and student work, this framework seems to work well for aligning EBM skills with the Practice-Based Learning and Improvement/Lifelong Learning and Self-Improvement competency. However, based on anecdotal evidence provided by the students in the second

semester of Year 1, there is a need to improve EBM integration throughout the basic sciences and clinical skills curricula.

Monday, May 21, 2018, 10:30 AM – 11:55 AM
Room: Embassy C (International Tower, Exhibit Level)

---

# Session: Information Management 1 (IM-1)

**Moderator: Alexandria L. Brackett, AHIP**

## Search Filter to Identify Reports of Randomized Controlled Trials in CINAHL

**Julie M. Glanville**
Associate Director, York Health Economics Consortium, York, England, United Kingdom

**Gordon Dooley**
Director, Metaxis, Curbridge, England, United Kingdom

**Susi Wisniewski**
Editorial Assistant, Cochrane Editorial Unit, London, England, United Kingdom

**Ruth Foxlee**
Information Specialist, Cochrane Editorial Unit, London, England, United Kingdom

**Anna Noel-Storr**
Information Specialist, Cochrane Dementia and Cognitive Improvement Group, Oxford, England, United Kingdom

**Objectives:** Published search filters to identify reports of randomized controlled trials (RCTs) on CINAHL Plus are not recent. Reports of RCTs in CINAHL may not be being identified efficiently and therefore searchers may be missing studies. This project was undertaken to develop, test and validate a search filter to identify reports of RCTs, quasi-RCTs and controlled clinical trials from CINAHL Plus.

**Methods:** Eleven sets of relevant and irrelevant records were identified. Nine sets were used to develop and test search filters iteratively. Two sets were used to validate the filter performance in terms of sensitivity and precision. The performance of two previously published filters and the filter built into EBSCOhost were also evaluated.

**Results:** Following a sequence of iterative development we have developed a filter which offers sensitivity of 0.88 (95% CI: 0.77 - 0.95) and precision of 0.36 (95% CI: 0.31 - 0.41). This is comparable to the sensitivity of the published filters, but represents a great improvement in terms of precision.

**Conclusions:** A sensitive and precise filter is available for use in identifying reports of RCTs, controlled clinical trials and quasi RCTs from the CINAHL Plus database via EBSCOHost. The strategy can be made more sensitive, but less precise, with the addition of other terms. The precision of the filter is such that it is likely to cut

the number of results that need to be scanned by researchers to a third of those retrieved by a subject search alone.

**Keywords:** Search filters, databases, randomized controlled trials, CINAHL, systematic reviews, searching

## Counting What Matters: Combining Robust Assessment Practices with Dynamic Visual Dashboards

**Jaime Blanck, AHIP**
Lead Informationist, William H. Welch Medical Library, Baltimore, Maryland

**Blair Anton, AHIP**
Associate Director, Informationist Services, William H. Welch Medical Library, Baltimore, Maryland

**Carrie Price**
Clinical Informationist, Welch Medical Library, Baltimore, Maryland

**Lori Rosman, AHIP**
Public Health Informationist, Johns Hopkins University, School of Medicine, Baltimore, Maryland

**Sue Woodson**
Associate Director, Collections, Johns Hopkins University, School of Medicine, Baltimore, Maryland

**Stella Seal**
Associate Director, Welch Services Center, Johns Hopkins University, Baltimore, Maryland

**Anne K. Seymour**
Director, Welch Medical Library, Baltimore, Maryland

**Objectives:** To replace cumbersome data collection practices and stagnate spreadsheets with a single, library-wide system that proactively collects meaningful, usable data that the Assessment Committee can use to demonstrate the library's contribution to broad institutional goals. A secondary objective was to support internal library management decision-making using data from this system to create customizable reports.

**Methods:** The Assessment Committee completed an inventory of current data collection practices and sources. The committee also reviewed ARL and AAHSL reporting metrics to insure that required basic data would continue being collected. The committee conferred with library leadership to identify frequently requested information from institutional stakeholders. Based on these investigations, the committee identified categories of "intended use" to track specific areas of services that impact clinical and academic work. The committee eliminated irrelevant and outdated data points that no longer reflected value to leadership. The frequency of data collection was also adjusted reduce burden on staff, where appropriate. Using a combination of Springshare tools, the committee created data collection forms, automated reports, and dynamic dashboards that allow for rapid analysis of services based on combinations of service type, service location, intended use, patron status, and patron affiliation among other variables.

**Keywords:** Assessment, dashboard

## Establishing a Secure Data Core for Medical Research and Data Curation

**Peter R. Oxley**
Associate Director of Research Services, Samuel J. Wood Library and C.V. Starr Biomedical Information Center, New York, New York

**Michael Bales**
Research Impact and Evaluation Informationist, Samuel J. Wood Library and C.V. Starr Biomedical Information Center, New York, New York

**John Ruffing**
Assistant Director, Samuel J. Wood Library and C.V. Starr Biomedical Information Center, New York, New York

**Terrie Wheeler**
Director, Samuel J. Wood Library and C.V. Starr Biomedical Information Center, New York, New York

**Objectives:** Research involving protected health information (PHI) must conform to strict regulatory requirements relating to data acquisition, usage and storage, and data management must adhere to FAIR principles (Findable, Accessible, Interoperable, and Reproducible). To satisfy these requirements, our library has partnered to establish a secure computing environment for PHI, that provides a responsive, client-focused service of data curation, and governance evaluation.

**Methods:** The library "Data Core" leverages the computing infrastructure and expertise of our ITS department, to provide a secure, virtual desktop computing environment for researchers' projects, students' masters theses, and student classrooms. The library staff ensure that the data access and usage within each project is in accordance with the project-specific data governance policies, and we have established a protocol for evaluating whether data are 'disclosure proofed' prior to export from the data core.
The data core has also created the opportunity to develop and test technologies that assist in data curation, for which we have already submitted an NIH grant.

**Results:** The data core was originally established as a pilot service for the department of Health Care Policy and Research. Since its inception, it has supported 55 research projects (with up to ten users per research project), two classrooms (with up to 46 students), and 10 masters projects. We have also processed 23 data export requests.
The Data Core also hosts four NYC Clinical Data Research Network collaborative projects, receiving and hosting data from six institutions.
The wide adoption of the service within the department has led to support to expand the service to the entire college by 2018.

**Conclusions:** The Data Core has successfully demonstrated the feasibility and value of integrating an ITS computing service with library expertise in data management and client-facing service. We look forward to being able to demonstrate the ability to leverage the Data Core for the development and testing of data curation services, by utilizing large datasets and an existing relationship with the researchers who would interface with the library services developed. The continued growth of the service reveals its perceived value to the users at the college, in its support of medical research.

**Keywords:** Data Core, curation, Protected Health Information

## Regarding Retractions: The Representation of Retracted Publications in Mental Health Literature

**Caitlin Bakker, AHIP**
Research Services Librarian, Bio-Medical Library, Minneapolis, Minnesota

**Amy L. Riegelman**
Social Sciences Librarian, Wilson Library, Minneapolis, Minnesota

**Objectives:** Retractions are a mechanism by which science corrects itself, withdrawing statements or claims that have proven to be erroneous. However, this requires that such corrections be clearly and consistently displayed. In the medical literature, inconsistency and obscurity have implications for research and patient care. This research considers how retracted publications in the mental health literature are represented across different databases.

**Methods:** Using Retraction Watch, we identified 144 retracted articles in the mental health field, ranging from papers in bench science to clinical research to social sciences. Between June and July 2016, we examined records across seven resources: PubMed, MEDLINE via Ovid, PsycINFO via Ovid, Web of Science, EBSCOhost, Scopus, and publisher websites. We determined the consistency and clarity of the retracted status of these publications in accordance with guidelines from the Committee on Publication Ethics (COPE) and the International Committee of Medical Journal Editors (ICMJE).

**Results:** Of the 812 records for retracted publications, 40.0% (n=325) did not indicate that the paper had been retracted. 26.3% of available PDFs (53/201) did not indicate that the paper had been retracted. Of the 144 articles studied, only 10 were represented as being retracted across all resources through which they were available.

**Conclusions:** Retracted publications are inconsistently represented across library resources. Our findings show that journal publishers were relatively consistent with regards to how frequently retractions were indicated while the platforms showed greater variability. While technical solutions, such as CrossMark by CrossRef, may bring help to mitigate these challenges, the inconsistent display of retracted publications has potential consequences for research and practice and raising awareness of this issue should be incorporated into the work of medical librarians.

**Keywords:** publishing, reproducibility, research ethics, data sharing, metadata, discovery

## Reproducibility Guidelines and Medical Librarians: Adapting Expertise, Transforming Services, Leading Change

**Franklin Sayre**
Pharmacy Liaison Librarian, Bio-Medical Library, Minneapolis, Minnesota

**Amy L. Riegelman**
Social Sciences Librarian, Wilson Library, Minneapolis, Minnesota

**Objectives:** The current reproducibility ‘crisis’ has consequences for the extent to which our students and faculty can trust experimental results when building on them for their own research or using them for patient care. Our objective is to investigate how academic library services and expertise can contribute to research reproducibility by analysing reproducibility guidelines from funders, publishers, and professional societies.

**Methods:** A narrative review of major guidelines from funders, publishers, and professional societies was

conducted. The health, social, behavioral, biological, and grey literatures were searched for guidelines on improving research reproducibility. Specific recommendations were then extracted from guidelines and a summary tables was created to compare recommendations common between guidelines. Academic library services and librarian expertise were linked with the recommendations in order to determine the potential roles for librarians in supporting research rigor and reproducibility. Services and expertise were then grouped by theme relating the known issues with research reproducibility.

**Results:** Many of the recommendations contained in major reproducibility guidelines are core areas of academic librarianship, including data management and sharing, scholarly communication and metrics, and methodological support for systematic reviews and data-intensive research, among other areas.

**Conclusions:** Librarians are well placed to play a major role in ameliorating the reproducibility crisis. Roles are varied enough that librarians supporting clinical medicine, nursing, pharmacy, dentistry, public health, and basic science research in both academic and hospital settings have significant roles to play.

**Keywords:** Reproducibility; medical libraries; research support, scholarly communication, data management

Monday, May 21, 2018, 10:30 AM – 11:55 AM
Room: Embassy D (International Tower, Exhibit Level)

---

# Session: Information Services 1 (IS-2)

**Moderator: Joy A. Russell, AHIP**

## Making Space for a Makerspace: 3D Printing and Scanning in a Health Sciences Library

**Natalie Logue**
Access Services Librarian, Robert B. Greenblatt, M.D. Library, Augusta, Georgia

**Gail Kouame**
Chair, Research & Education Services, Greenblatt Library, Augusta, Georgia

**Bettina Askew**
Medical Library Associate, Robert B. Greenblatt, M.D. Library, Augusta, Georgia

**Objectives:** To advance the clinical and educational objectives of the university and to foster innovation by implementing a Makerspace in the health sciences library.

**Methods:** A committee of three librarians secured funding through a Technology Improvement Award from the National Network of Libraries of Medicine to purchase a Stratasys Mojo 3D printer and wave wash system, as well as an EinScan-SE 3D scanner and additional equipment and material needed to establish a Makerspace in the health sciences library. The Mojo 3D printer uses both hard and soluble plastic. The soluble plastic will dissolve in the wave wash system and allows users to print very delicate and interlocking parts. A committee of two librarians and one staff member is now in place to implement the Makerspace, develop policies, and provide training. The committee will identify opportunities to weave 3D printing and scanning across the curriculum in science and health care. The Makerspace area is included in a remodeling project currently underway at the health sciences library.

**Results:** Within two weeks of getting the 3D printer and scanner set up for staff training, a faculty member requested us to perform a 3D scan so that she could collaborate with a medical illustrator on a project. The innovative nature of Makerspaces fosters interdisciplinary research and rapid prototyping, which will be encouraged. The results will discuss learning curves, software, resources, and academic uses of the Makerspace.

**Conclusions:** There is a learning curve associated with the maintenance of the Makerspace equipment and the potential uses for the available technology. However, the support of online communities, collaborative brain-storming with faculty and students, and partnerships with departments such as computer science and medical illustration has driven the Makerspace forward. Future goals for the Makerspace will include more resources on the LibGuide to assist users, scheduled events to encourage participation, and the incorporation of Makerspace

equipment into class projects.

**Keywords:** makerspace; 3D printing; 3D scanning; software; programming

## Medical Librarian Citation Manager Use and Instruction across the United States

**Emily C. Weyant, AHIP**
Clinical Reference Librarian, East Tennessee University Quillen College of Medicine Library, Mountain Home, Tennessee

**Nakia J. Woodward, AHIP**
Senior Clinical Reference Librarian, East Tennessee University Quillen College of Medicine Library, Mountain Home, Tennessee

**Elisabeth Wallace**
Library Assistant, East Tennessee University Quillen College of Medicine Library, Mountain Home, Tennessee

**Objectives:** This study is an examination of the state of citation manager use and instruction by medical librarians across the United States and US territories. It focuses on librarian preference for citation managers and related instruction. The purpose of this study is to reveal barriers to and preferences for citation managers and citation manager instruction in hospital and academic libraries.

**Methods:** A literature review performed prior to undertaking this project revealed minimal current literature on citation manager instruction in health sciences and medical libraries. Citation managers evolve quickly, negatively impacting the relevancy of older literature. In effort to capture current reflections on citation manager use and instruction in health science and medical libraries, a qualitative survey was devised and disseminated via medical library listservs in late summer 2017. Questions included in this survey as well as the survey platform and data collection procedures were approved by East Tennessee State University's Institutional Review Board. Questions discussed librarian citation manager use preferences, instruction styles, barriers to instruction, and perception of value. RedCap was utilized for survey dissemination and analysis. Survey recipients received two weeks to respond to survey questions after which data was compiled and analyzed by researchers to reveal trends.

**Results:** This survey garnered 238 responses, 61% from academic librarians, 27% from hospital librarians, and 12% from other librarians. Respondents identified Zotero as the most utilized free citation manager and EndNote as the most utilized paid citation manager. Lack of patron interest was the most significant barrier identified by hospital librarians while lack of citation manager awareness was the greatest barrier for academics. Although 97% of respondents either agreed or strongly agreed that citation manager use instruction falls within library instructional domains, 82% of librarians surveyed report that they did not receive citation manager instruction while pursuing their library degrees.

**Conclusions:** As librarians assume responsibility for citation manager instruction and use, time must be dedicated to training of librarians to utilize citation managers and effectively teach them to others. Whether this training should occur in school or on the job is debatable and subject to circumstance. Additional recommendations include increased promotion of citation manager availability, purpose, and instruction opportunities in institutions where this is feasible. Limitations of this study include a small sample size with a bias towards respondents familiar with citation managers working in institutions with citation manager subscriptions.

**Keywords:** citation manager, library instruction, library education, hospital and academic libraries

## Needs Assessment for Data Services and Training at a Research University

**Zoe Pettway Unno**
Head, Science and Engineering Library, Science and Engineering Library, Los Angeles, California

**Jennifer E. Dinalo**
Information Services Librarian, USC, Los Angeles, California

**Objectives:** Campus initiatives to promote transparency and reproducibility in research at a research university provide an opportunity for the university's libraries to develop support for data services. To best utilize limited library resources, a needs assessment survey will provide guidance in the development of data services.

**Methods:** A needs assessment survey will be developed using the survey tool Qualtrics. The survey will be designed to identify gaps in knowledge and services as it relates to data services including: writing data management plans, identifying data repositories, compliance with funding mandates and data curation. Survey will be distributed through email to the campus community. Participants will be entered in a drawing for Amazon gift cards as an incentive for participation. Data will be compiled and analyzed in Excel. The results will be used to inform training for librarians and development of services and workshops for the campus community. Successful implementation of services can establish the library as a key participant in the research enterprise.

**Results:** My study is not complete yet. If selected, I will come back into the system and enter my results

**Conclusions:** My study is not complete yet. If selected, I will come back into the system and enter my conclusions

**Keywords:** Data Services
Data Management
Needs Assessment
Research
Reproducibility
Survey

## Many Hands Make Light Work: Leveraging Campus Expertise to Provide Data Science Support

**Lisa M. Federer, AHIP**
Research Data Informationist, NIH Library, Bethesda, Maryland

**Objectives:** With increased interest in data science, many libraries would like to provide services and support for their users’ increasingly complex data needs. However, developing and sustainably implementing data services requires a considerable time investment from staff with significant technical expertise. This talk discusses how libraries can stretch limited resources by leveraging expertise from the community to provide data science support.

**Methods:** Even if library have experienced staff, demand for data science support often exceeds library capacity. Fortunately, volunteers are often eager to share their expertise if the library can provide the planning, administrative structure, and physical space, as described here. First, the library coordinated “R Office Hours” staffed by experienced volunteers, allowing newer users to drop in with questions. Library staff handle administration of a Data Science Mentoring program that pairs learners with more experienced data scientists who share their interests. The library also hosts volunteer-led classes, including Data and Software Carpentry workshops.

**Results:**
Volunteer-provided services have been popularly and widely utilized, allowing a greater number of attendees to benefit. Offering new services has also helped increase the awareness of the library as a place for assistance with data-related questions and has increased partnerships between the library and other units.

**Conclusions:**
Using the type of collaborative approaches described here allows the library to provide a range of data science support and benefit volunteers by giving them the opportunity to share their knowledge and gain teaching experience. These services also help develop community and promote networking by connecting individuals with a common interest in data science.

**Keywords:** data science, cooperative service models, instruction, data services

## Up All Night: The Accreditation-Driven Trend toward 24-Hour Access among Medical School Libraries (Survey and Self-Study)

**Electra Enslow**
Head of Library Research and Instruction, Spokane Academic Library/Washington State University, Spokane, Washington

**Objectives:** Driven by accreditation concerns and student requests, the Spokane Academic Library, in service to Washington State University's Elson S. Floyd College of Medicine, undertook to explore current trends and best practices for 24-hour access among medical school libraries and to determine a course of action for offering the service to medical students and others at a dual-institution health sciences campus.

**Methods:** In 2016, the authors conducted an online survey of members of the Association of Academic Health Sciences Libraries (AAHSL), the library counterpart of the Association of Academic Medical Colleges (AAMC), which oversees medical school accreditation through the Liaison Committee for Medical Education (LCME). The survey probed a variety of issues related to offering 24-hour library access, including whether it is offered in the first place and for how long, impediments to offering it, intentions of offering it in the future, staffing considerations, security considerations, sources of resistance, marketing and education, what has worked well, what would be done differently, and overall satisfaction with the service. Informed by the survey results, library staff and faculty began an internal conversation about offering 24-hour access, followed by a proposal to campus administration to do so, followed by implementation of the service.

**Results:** Of the 99 medical libraries that responded to the survey, 59% reported current 24-hour access for students. Of those libraries not currently providing 24-hour access, 78% are considering offering it in the future. 24-hour library access is clearly a trend among health sciences libraries and is strongly encouraged by the Liaison Committee for Medical Education (LCME) accreditation process. 61% of responding libraries offering 24-hour access have transitioned to it within the past 5 years; and the importance of offering 24-hour access in the context of LCME accreditation (related to student input) was frequently mentioned in survey comments.

**Conclusions:** After an in-depth review of the online survey the library decided it was appropriate to move forward with this service. Incorporating information from the survey participants, the library implemented a badge-access after-hours service. The library trained staff, increased security, made cleaning supplies available, installed video surveillance on entrance doors, and informed students of their responsibilities in accessing the library after-hours. With support from campus administrators, library staff, and security, this service has been implemented and has been well received by faculty, students, staff and the LCME board.

**Keywords:** library scheduling, 24-hour access, after-hours access, medical school libraries, accreditation

## Using an Adapted Tool to Transform Medical School Faculty Evaluation of Open Access Journals and Lead to Wider Campus Acceptance of Open Access Publishing

**Sheila W. Green**
Bryan Campus Librarian, Medical Sciences Library, Bryan, Texas

**Bruce E. Herbert**
Director, Office of Scholarly Communication, College Station, Texas

Objective: To share how an adaptation of the Association of American Medical Colleges (AAMC) Annotated bibliography of journals for educational scholarship is being transformed into a program to facilitate medical faculty assessment of open access (OA) journals, and is enabling librarians to lead open access evaluation for faculty on the main campus. Methods: A companion reference chart indicating MEDLINE indexing, journal impact quartile rankings, and open access and/or self-archiving policy was adapted from the Association of American Medical Colleges (AAMC) Annotated bibliography of journals for educational scholarship and made freely available in April 2017. This chart has been downloaded from the institutional repository over 800 times. Scholarly communications leadership saw this format as an opportunity to overcome “predatory journal” anxiety that many faculty face when considering open access journals for publication. A pilot project with a medical school research team was developed to identify and supply open access journal quality criteria for journals relevant to the team’s research and publishing goals. After an initial proof-of-concept demonstration to the lab director, criteria and subject areas were refined, and a new journal reference chart prepared. Faculty will have a tool for both subscription and open access journal selection that is not only relevant to their research area but also sends a positive message about the potential of OA publication. Results: Outreach efforts to labs, centers and departments are ongoing and impact is measured by changes in the number of applications to the institution’s fund to subsidize OA author fees. Main campus subject librarians have formed a working group to tailor the process and format for their client groups. Conclusion: While every discipline has unique norms of publication, all faculty want common assurance about the quality and impact likelihood of the venue in which they publish. Librarians can lead faculty to data-driven decision-making by adapting and consolidating existing tools into easy-to-use reference sheets and transforming the OA landscape from predatory to inviting.

Monday, May 21, 2018, 10:30 AM – 11:55 AM
Room: Embassy A (International Tower, Exhibit Level)

---

# Session: Lightning Talks 2

**Moderator: Rebecca Morgan, AHIP**

## Using Data to Transform and Lead Library Instruction: Hospital Librarians Utilize Learning Needs Assessment Survey Results to Adapt to the Learning Needs of Nursing Staff

**Diana Almader-Douglas**
Librarian, Mayo Clinic Arizona/Hospital Library, Phoenix, Arizona

**Lisa Marks, AHIP**
Director, Library Services, Staff Library, Scottsdale, Arizona

**Objectives:** Hospital library staff will apply Learning Needs Assessment (LNA) survey data to examine whether supporting learning style profiles of nursing staff when teaching library resource use or literature searching instruction contributes to satisfaction with library services, or confidence or skill with literature searching.

**Methods:** Hospital librarians joined the Department of Nursing to determine learning styles of nursing staff. Librarians performed literature searches to 1) establish variations of learning profiles of nurses and 2) identify a validated survey tool to determine preferred learning styles. Through an IRB approved research study, Felder-Solomon's Index of Learning styles was selected and administered to hospital nursing staff between 1/1/17 and 4/20/17. 2098 paper and electronic responses were collected at a 69% response rate. Analysis was completed using SAS Studio statistical software and RStudio. Based on results, librarians are revising library instruction to meet the dominant learning profiles of nursing staff. When able, feedback and post session evaluations will be collected to assess level of satisfaction with instruction, literature searching confidence, and searching skills.

**Results:** According to the LNA survey results, 61% of staff nurses have a baccalaureate degree and 48% of nurses fit into the millennial (Generation Y) generational category. Survey results show a higher number of active, sensing, visual and sequential learners, regardless of generational age, experience, or gender, so it makes sense to tailor library instruction to learners in their preferred learning styles by including active or 'hands-on' participation and small group activities. Three groups consisting of advanced practice nurses, nurse residents, and nurses enrolled in an introductory evidence based practice course have received tailored instruction. Post instruction feedback or responses have not yet been received, but positive outcomes have been verbalized by course instructors and participants.

**Conclusions:** In line with Mayo Clinic's primary core value that 'the needs of the patient come first', hospital library staff aims to deliver optimal library service at all times, including formal instruction. During this project, we believe 'the needs of our learners come first', and anticipate this will lead to better outcomes.

**Keywords:** Data, survey, nurses, learning styles, nursing education, Felder and Solomon

## Participatory Leadership in Library Strategic Planning

**Emily J. Glenn, AHIP**
Education and Research Services Librarian, McGoogan Library of Medicine, Omaha, Nebraska

**Emily McElroy**
Director & Assistant Vice Chancellor for Academic Affairs, McGoogan Library of Medicine/ University of Nebraska Medical Center, Omaha, Nebraska

**Alison Bobal**
Head, Collection Development and Metadata, University of Nebraska Medical Center, McGoogan Library of Medicine, Omaha, Nebraska

**Objectives:**The process of strategic planning moved from a top-down approach, usually led by the director and department heads, to one where five small teams focused on specific areas: collections, special collections and archives, outreach, education and research services, and space. This shift in process dovetailed with the director's emphasis on invigorating staff participation.

**Methods:** The director formed five teams, each led by a staff member whose regular job responsibility focused on that area. To launch planning, all staff participated in design thinking exercises, facilitated by outside experts. Strategic planning teams were encouraged to use design thinking techniques in the planning process. Over several months, teams met to identify the big challenges in their area and use them to strategic initiatives. Team leads then examined where initiatives overlapped to ensure that diversity, wellness, and technology were woven throughout the overall strategic plan. Team leaders and the director reflected on what went well and stumbling blocks, then tuned messaging and activities to maintain momentum. Staff were asked to review and provide feedback to the strategic plan at different times in the process.

**Results:** The planning team structure provided an opportunity to grow leadership skills of librarians who do not lead units. Design thinking strategies helped teams to envision how new successes looked, rather than how to stretch current tasks to fit. A well-timed feedback loop permitted communications to flow between team leads, library staff, library council, and supervisors. Team leads also gained practice in collaborative writing, negotiating priorities during uncertain times, and developing metrics that matter to the institution. Some staff exhibited seemingly newfound enthusiasm, positivity, and responsibility toward the strategic changes in direction.

**Conclusions:** The team-based approach extended the planning process timeline, but this was anticipated. Rather than rush to completion, we intended to iterate on broad strategic directions to create clear and achievable outcomes. Still, even when people were engaged, fatigue set in during the long, iterative process. Following waning levels of feedback and focus in the middle of the process, interest was reignited during task assignment, when staff could clearly identify their deliverables. The final three-year strategic plan clearly maps to the university's plan, clarifies the lifecycle of goals, and provides transparency in accountability of tasks.

**Keywords:** Leadership
Strategic planning
Design thinking
Alignment
Culture change
Participation

## Longitudinal Library Curricular Integration and the Association of College and Research Libraries Information Literacy Framework

**Jean Song**
Assistant Director, Academic and Clinical Engagement, Taubman Health Sciences Library, Ann Arbor, Michigan

**Judith E. Smith**
Informationist, Taubman Health Sciences Library, Ann Arbor, Michigan

**Jacqueline Freeman**
Informationist, Taubman Health Sciences Library, Ann Arbor, Michigan

**Objectives:** In order to provide a holistic view of longitudinal library learning, a team of librarians is mapping the Association for College & Research Libraries' (ACRL) Framework for Information Literacy for Higher Education to a health sciences library's curricular instructional session objectives and to the distinct health sciences schools' competencies at a large academic institution.

**Methods:** A team of librarians consisting of the primary liaison for each of the health sciences schools at a large academic institution and the librarian liaison to the institution's interprofessional education center created a single spreadsheet documenting the library's curricular instructional sessions and metadata about the sessions, including session objectives. Each primary liaison also identified that school's educational competency requirements. Individual librarians were assigned to map the ACRL information literacy framework to the school's competencies. Librarians teaching instructional sessions were also asked to map the session objectives to the information literacy framework. Discrepancies between individual mappings of schools' competencies and the framework were discussed by the individual librarians assigned for that mapping to arrive at a consensus mapping agreement. Consensus mapping was facilitated through a mapping glossary.

**Keywords:** information literacy, curriculum integration

## Benchmarking Participation of Veterinary Librarians in Systematic Reviews and Scoping Reviews

**Lorraine Toews**
Librarian, Veterinary Medicine and Bachelor of Health Sciences, University of Calgary Health Sciences Library, Calgary, Alberta, Canada

OBJECTIVES: Anecdotal evidence suggests that veterinary librarians do not participate in systematic and scoping reviews (SRs) to the same extent as medical librarians. This study describes the current state of veterinary librarians' participation in SRs, barriers to participation and level of training in SR methods. Study findings provide benchmarks for training and tracking future changes in veterinary librarians' participation in SRs. METHODS: An online survey of multiple-choice and open-ended questions was emailed to 60 librarians working in veterinary colleges and veterinary university libraries in Australia, Canada, England, Ireland Scotland and the United States. The survey asked veterinary librarians about specific roles they had played in SRs in the past three years, how they rated their level of training in SR methods, factors that had limited their involvement in SRs and their institution's policies for librarian involvement in SRs. RESULTS: The response rate was 30% (n=20). Respondents participated most frequently in the SR roles of question formulator, database selector and search strategy developer. Participation in other SR roles such as article selector, data extractor and article appraiser was significantly lower. 60% of respondents received few/no requests from veterinary faculty/students at their institution to participate in SRs, and 40% of respondents stated that veterinary faculty/students at their institution rarely conducted SRs. Barriers to participation in SRs included insufficient training, not enough time

and a lack of institutional policies on librarian roles in SRs. CONCLUSIONS: Study results suggest that current veterinary librarian participation in SRs is limited, both in terms of the numbers of librarians involved and the roles played by librarians on SR teams. Training in conducting SRs and the development of institutional policies for librarian roles in SRs could facilitate greater participation of veterinary librarians on SR teams and enable them to function in a greater range of roles including co-authorship. Further research is needed to understand why many veterinary faculty members are not conducting SRs so that academic library SR services can be customized and marketed more effectively to animal health researchers.

## The Personal Librarian Program: Transforming Our Relationship with Our Medical Students

**Shalu Gillum, AHIP**
Head of Public Services, Harriet F. Ginsburg Health Sciences Library, Orlando, Florida

**Natasha Williams, AHIP**
User Services Librarian, Harriet F. Ginsburg Health Sciences Library, Orlando, Florida

**Pamela R. Herring, AHIP**
Electronic Resources Librarian, Harriet F. Ginsburg Health Sciences Library, Orlando, Florida

**Deedra Walton, AHIP**
Head of Electronic Resources, Harriet F. Ginsburg Health Sciences Library, Orlando, Florida

**Nadine Dexter, AHIP**
Director Harriet F. Ginsburg Health Sciences Library, Harriet F. Ginsburg Health Sciences Library, Orlando, Florida

**Objectives:** To create a Personal Librarian Program (PLP) to (1) increase librarian engagement with first and second year medical students; and (2) promote awareness of library resources and services. For years the library struggled with finding ways to connect with medical students. We created the PLP to give students an individualized library experience and an easier way to interact with librarians.

**Methods:** Each class of students is divided equally amongst the current faculty librarians. Students receive information about the PLP and meet their Personal Librarian during orientation week. Throughout the year, the library sends monthly emails to students from their Personal Librarian about events happening at the library and services offered to help them with research projects and other assignments. Personal Librarians meet with their students one-on-one throughout the year to assist with literature searching, formatting references, and other library-related questions.

**Results:** As a result of personalized communication from their Personal Librarian, first and second year students increasingly seek assistance from librarians in-person and via email. Since we began the PLP four years ago, first and second year students who are now third and fourth students continue to engage with their Personal Librarian.

Through the success of the PLP, faculty librarians are now engaging with students through the curriculum. Personal Librarians are formally integrated into the Research and Practice of Medicine modules, reviewing students' research reports, and providing feedback on students' use of evidenced-based medicine resources when answering clinical questions with patients.

**Conclusions:** The PLP not only allows us to provide individualized attention to our students, but has increased library integration into modules, and has improved engagement with students. The library would like to increase

PLP participation in other modules and create more opportunities for librarian-student interaction.

**Keywords:** Personal Librarian Program, personal librarians, public services, curriculum integration

## Transforming Information Literacy Training by Adapting to Students' Clinical Placements: Bridging the Gap between Theory and Practice in Nursing Education with E-Learning

**Margrethe Søvik**
Senior research librarian, VID Specialized University, Fyllingsdalen, N/A, Norway

**Objectives :** This presentation focuses on the library's role in redesigning an assignment for undergraduate nursing students in clinical placements. Transforming the assignment has helped moving from one-shot library instructions to making it a part of an assignment where higher-order skills as evaluation, source criticism, synthesis and reflection are integrated with concrete and hands-on information literacy training.

**Methods :** Nursing students in their final year search for literature to make educational presentations in clinical placements in mental health and home care services. Steps in evidence-based practice are "translated" into the assignment. The library is responsible for an online module supporting the students in the process of working with their assignment, where the students apply what they learnt about information searching on clinical topics. One goal is that the students should experience more coherence between theory in college and practice in clinical placements. Students get feedback from the librarian on topic and preliminary search. The students submit a paper on how they searched for information, evaluate their own process and reflect upon how and where they search for information. Additionally, students give feedback through surveys after the clinical placements. The materials are continuously analyzed to evaluate and redesign the module.

**Results :** Students feel confident about: Reading and understanding research, searching for new information and presenting what they have learned (written and orally). Some students report to struggle with deciding upon a topic. We can also see that some have problems with formulating questions and «translating» topic to search terms (via PICO), and being consistent when doing this. Some also struggle with finding articles that answer their topic and writing about research. The students that use the resources have used them several times and say that they have been useful. Some students have not used any of the resources. Even though many search for reviews, some still mainly aim for that one research article

**Conclusion :** It has been positive that the library has been involved in the entire process of information seeking and there has been much student-library interaction. In the assignments students reveal that they had some «aha-moments» doing the assignment. A preliminary analysis of assignments indicate that they work with information seeking on all levels of Bloom's taxonomy. However, it is labor-intensive and scaling may be an issue if there are big student groups.

**Keywords :** information literacy, evidence-based practice, nursing education, undergraduate students, clinical placements

## Teaching PubMed: Staying in the Shallows or Diving Deep

**Erica R. Brody**
Research and Education Librarian, Tompkins-McCaw Library for the Health Sciences, VCU, Richmond, Virginia

**John W. Cyrus**
Research and Education Librarian, Virginia Commonwealth University, Richmond, Virginia

**Objectives:** To deliver evidence-based care, healthcare professionals and students must search the peer-reviewed literature efficiently. Complex bibliographic databases have many features for creating and honing search queries. Which of the many facets of searching do librarians teach? This study examines the content of librarian-led database searching instruction.

**Methods:** To ascertain current medical librarian practice for teaching literature searching, we surveyed librarians about the elements of searching that they teach. Participants were invited via MLA listservs. Quantitative analysis of survey data will be complemented by a content analysis of learning objectives and slides collected.

**Results:** The 95 five medical librarians respondents teach searching in the following disciplines: medicine (43%), nursing (20%), allied health (12%), public health (12%), dentistry (9%), and pharmacy (4%). Most respondents teach the following concepts: filters/limits; identifying key concepts; advantages of using keywords and controlled vocabulary, controlled vocabulary, and Boolean operators. The content of searching instruction varied by discipline and education level of learner.

**Conclusions:** Do librarians Dive Deep? Maybe. The elements of searching most commonly taught span the basics of searching (identify key concepts; filters/limits) to advanced techniques that require greater understanding of the way each database works (Boolean operators; controlled vocabulary). Next steps involve content analysis of the learning objectives and slides collected from respondents.

**Keywords:** literature search, PubMed, instruction, digital literacy, survey, content analysis

## Talking with Your Doctor: Using Information Resources to Improve Health Communication

**Elizabeth Irish, AHIP**
Assistant Professor, Schaffer Library of Health Sciences, Albany, New York

**Kara Burke**
Director for Community Engagement, Division of Community Outreach and Medical Education, Albany, New York

**Enid Geyer, AHIP**
Associate Dean for Information Resources and Technology, Schaffer Library of Health Sciences, Albany, New York

**Ingrid Allard**
Associate Dean for Community Outreach and Medical Education, Division of Community Outreach and Medical Education, Albany, New York

**Objectives:** Low health literacy is linked with poor health, poor health care utilization, increased barriers to care, and early death. Albany Medical College (AMC) collaborated with community partners on workshops to improve health literacy and patient empowerment by providing community members with tools to research health information online, prepare for medical appointments, and use this information to improve communication with their physician.

**Methods:** AMC's Schaffer Library of Health Sciences (SLHS) and Division of Community Outreach and Medical Education (DivCOME) collaborated to implement this project. Two community partners, the Center for Law and Justice and Community Caregivers, were identified through the DivCOME's existing relationships. AMC's Institutional Review Board approved this project.

During the planning period, AMC faculty collaborated with community partners on content, site selection, and promotion. Representatives from the community organizations attended the workshops. A graduate student intern assisted with workshop preparation and evaluation.

Eight interactive workshops with 6-15 participants were delivered at community sites in urban, rural, and suburban settings. Topics include patient empowerment, appointment preparation, health information research, and hands-on practice. Workshops were evaluated using paper pre-/post- questionnaires, which were entered and analyzed in Qualtrics.

**Results:** There were 54 total participants. We collected 38 pre-questionnaires (70% response rate) and 36 post-questionnaires (67% response rate). The percentage indicating that they are “Very Confident” or “Confident” in their ability to find quality health information on the internet went from 25% to 91%. 97% indicated that they intended to tell a family member/friend about the websites and they would change where they look for health information. 100% indicated that they will be more comfortable explaining things to their physician, and 97% felt that the workshop improved their ability to ask questions at their next appointment.

**Conclusions:** These workshops were effective in improving individuals’ confidence in their ability to prepare for medical appointments and research health information. There is potential to replicate these workshops in other settings. Year 2 NNLM/MAR funding supports collaboration with five public libraries to deliver the community workshops and provide MLA CE credits leading towards MLA's Consumer Health Information Specialization Level 1 for their librarians.

This project has been funded in whole or in part by the National Library of Medicine (NLM), National Institutes of Health (NIH) under cooperative agreement number UG4LM012342 with the University of Pittsburgh, Health Sciences Library System.

**Keywords:** Patient Communication; Community-Based Collaborations; Consumer Health; Patient Empowerment; Health Literacy

## Search Workbench: Building, Refining, and Comparing PubMed Searches Using Interactive Visualizations

**Edwin V. Sperr, Jr., AHIP**
Clinical Librarian, Graduate Medical Education, AU/UGA Medical Partnership, Athens, Georgia

**Objectives:** Search Workbench is designed to facilitate the process of developing complex PubMed searches by allowing the user to examine, edit and visualize searches from a single interface. Importantly, the system not only displays multiple visualizations on one screen, it also allows for direct comparisons of completed searches to one another, speeding the process of fine-tuning a search strategy.

**Methods:** The NCBI API (E-utilities) is used to directly query PubMed, and search results are retrieved, parsed and visualized inside a web browser using JavaScript/jQuery. Each search is simultaneously visualized in two ways: as a Venn diagram of constituent terms using venn.js and as a line chart of yearly proportions of results using Google Charts. The interface allows for hand-editing the Automatic Term Mapping translation of a search and the application of one of several pre-defined hedges. Past searches within a session are itemized and can be selected for comparison to one another. In addition, individual search sessions can be saved and later

shared as text files.

**Keywords:** PubMed, data visualization, bibliometrics, database interfaces

## Programming for Biology: Learning the Language to Meet the Needs of Biomedical Researchers

**Carrie L. Iwema, AHIP**
Coordinator of Basic Science Services, Health Sciences Library System, Pittsburgh, Pennsylvania

**Melissa Ratajeski, AHIP**
Coordinator of Data Services; IACUC Liaison, Health Sciences Library System, Pittsburgh, Pennsylvania

**Ansuman Chattopadhyay**
Assistant Director, Molecular Biology Information Service, Health Sciences Library System, Pittsburgh, Pennsylvania

**Objectives:** We are an academic health sciences library at a large research university that provides numerous resources for data analysis. However, researchers are beginning to request training in basic programming to tackle specific analytical questions using open source software. Thus, we embarked on a new initiative to address this latest trend in biomedical researcher needs.

**Methods:** Our university provides support for many programming languages, but there is a gap between what computer scientists think is “basic” and what a bench/clinical researcher thinks, i.e., how to even launch the command prompt. We believe that subject specialists in the library can bridge this gap via a twofold approach: (1) offer *very* basic as well as biomedical-specific programming workshops to researchers in collaboration with university computational experts and (2) become acquainted with programming languages ourselves to translate jargon, needs, and capabilities between the computational experts and researchers. One of our librarians attended a competitive 2+ week in-person training course on the Python programming language and how to apply it to biological problems. Using this knowledge we are planning classes with the computational experts, including Data Viz and Command Line Basics. This presentation details our new programming for biology initiative.

**Keywords:** research, programming, biomedical, data visualization, biology, data, initiative, workshops, computer

## Utilizing Virtual Reality as a Method to Facilitate Learning

**Janet L. Hobbs, AHIP**
Associate Director, Medical Library, Medical Library, Los Angeles, California

**Objectives:** How can virtual reality impact adult learners in an academic medical center setting?

The Medical Library acquired a Samsung Gear Virtaul Reality headset and goggles. The Library has publicized the item and circulates it to registered Library patrons. The Library is following the circulation statistics to learn how Housestaff urilize them in their academic and clinical activities.

**Methods:** A major academic Medical Library in Los Angeles California serves the informational needs of over 60 residency programs (Housestaff and Fellows). This medical center is a complex teaching hospital with over

900 beds. The Library will review circulation data for all users of the Library to determine usage patterns, Housestaff affiliation and teaching elements. The exposure to the data will provide insight as to early adopters of this technology and provide implications for future engagement in training and compentency based activities. The Library will survey users about their experiences and insight and ascertain effectiveness of virtual reality systems as a learning system.

**Results:** The author will describe the results are they become available.

**Conclusions:** The author will report conclusions after data has been collected and analyzed.

**Keywords:** Virtual Realtiy, Technology, Patient interventions, Simulation, User Computer Interface

## A Bibliometric Study of the Scholarly Communications of Senior and Distinguished Members of the Academy of Health Information Professionals

**Jodi L. Philbrick, AHIP**
Senior Lecturer, Department of Information Science, Denton, Texas

**Ana D. Cleveland, AHIP, FMLA**
Regents Professor, Sarah Law Kennerly Endowed Professor, and Director, Health Informatics Program, Department of Information Science, Denton, Texas

**Objectives:** The purpose of the study was to examine the publication record of senior and distinguished members of the Medical Library Association (MLA)'s Academy of Health Information Professionals (AHIP), specifically to identify the number of publications members have produced, rates of co-authorship, and the journals in which their publications appear.

**Methods:** 507 senior and distinguished members of the Academy of Health Information Professionals were identified using the MLA Membership Directory in November 2016. The members' names were searched using Web of Science Core Collection (1995-May 2017) to gather information about their publishing record, including number of publications, co-authorship, and publishing journals.

**Results:** Of the 189 senior and 318 distinguished AHIP members, 41% (78) of senior members and 58% (186) of distinguished members have authored or co-authored a publication indexed by Web of Science Core Collection. The 264 members have produced a total of 1099 publications for a mean number of 4 publications per member., 62% (676) of the publications have been co-authored. The top 3 journals where senior and distinguished AHIP members have published include: Journal of the Medical Library Association, Library Journal, and The Journal of Family Practice.

**Conclusions:** A greater percentage of distinguished members versus senior members of the MLA's AHIP have authored or co-authored a publication indexed by Web of Science Core Collection. The majority of the articles published are co-authored. Not surprisingly, the top two journals where members' articles appear are related to library and information sciences (LIS); however, the third journal indicates that members are publishing outside of LIS. This study allows for greater understanding of the way that health information professionals are contributing to the LIS and health sciences literature.

**Keywords:** Bibliometrics, health information professionals, scholarly communication

## Leveraging Our Strengths: Building Capacity for Sustainable Integration into Systematic Review Projects

**Whitney A. Townsend**
Informationist, Taubman Health Sciences Library, Ann Arbor, Michigan

**Kate M. Saylor**
Informationist, Taubman Health Sciences Library, Ann Arbor, Michigan

**Objectives:** To describe the development of an interdisciplinary Systematic Review and Expert Searching Special Interest Group (SIG) at a large academic institution.

**Methods:** Building capacity for sustainable integration into systematic review projects is a challenge for many organizations with a large pool of systematic review-producing faculty, students, and staff. In addition, more researchers from social sciences and other fields are conducting projects that require structured, systematic searching and data management, with their librarians looking to the health sciences for guidance. To address this need, a pair of two health sciences librarians convened a local SIG and planned a longitudinal curriculum of in-person discussion sessions, sponsored webinars, presentations, and the development of a group-sourced search strategy on a systematic review topic. The SIG leads used a liberating structures exercise to help identify the most pressing skill development needs and discussion topics from the SIG participants, then leveraged local expertise and Medical Library Association CE opportunities to build their curriculum.

**Results:** If accepted, results will be provided in February 2018

**Conclusions:** If accepted, results will be provided in February 2018

**Keywords:** systematic reviews, skill-building, competencies, continuing education

Monday, May 21, 2018, 10:30 AM – 11:55 AM
Room: International Ballroom South (International Tower, Ballroom Level)

---

# Special Content Session: From Abstract to Tangible: Shifting Health Sciences Instructional Mindsets using the ACRL Framework for Information Literacy

**Moderators: Stephanie J. Schulte, Heather Collins, AHIP, Lin Wu, AHIP**

## From Abstract to Tangible: Shifting Health Sciences Instructional Mindsets Using the Association of College and Research Libraries Framework for Information Literacy

**Stephanie J. Schulte**
Head, Research and Education Services, Health Sciences Library, The Ohio State University, Columbus, Ohio

**Heather Collins, AHIP**
Assistant Director, Research and Learning Department, A.R. Dykes Health Sciences Library, Kansas City, Kansas

**Lin Wu, AHIP**
Assistant Director/Associate Professor, Research & Learning Services, Health Sciences Library, Memphis, Tennessee

**Molly Knapp, AHIP**
Training Development Specialist, NNLM Training Office, Houston, Texas

**Emily Brennan**
Research and Education Informationist, Medical University of South Carolina (MUSC) Library, Charleston, South Carolina

**Elizabeth Moreton**

**Jamie Conklin**
Health Sciences Librarian and Liaison to Nursing, University of North Carolina at Chapel Hill Health Sciences Library, Chapel Hill, North Carolina

**Jessica Sender, AHIP**

**Brenda M. Linares, AHIP**
Health Sciences Librarian, School of Nursing, KU Medical Center, Olathe, Kansas

**Joey Nicholson**
Education and Curriculum Librarian, NYU Health Sciences Library, New York, New York

**Program Description:** This special content session will work to explore the abstract concepts of the ACRL Framework for Information Literacy to create tangible, practical ideas for modifying common instructional situations. Built upon key concepts recently presented in the ACRL Framework for Information Literacy Toolkit and modeling the Understanding by Design concepts, the session will inspire health sciences librarians to critically think about their teaching approaches and leave with fresh ideas to better engage their constituents.

Session Outline
A. Big Ideas: The enduring understandings we hope attendees will gain from the session. Slide presentation and participatory exercise. 30 minutes.
a. Introduction and review of the state of health sciences education
b. The ACRL Framework Basics
c. How health sciences librarian education and the Framework can work together (participatory exercise)
B. Addressing essential questions: What do we want students to learn after a session/course with librarians? How does the Framework align with health sciences teaching objectives? Panel of 3-4 librarian instructors presenting exemplars illustrating Framework-related teaching approach for common essential questions. 30 minutes
C. Backward Design: How can librarians modify their teaching approach to incorporate the ACRL Framework? Facilitated large group discussion with time for questions and answers centered on commonalities among exemplars, activities that might be used, and planning a session around these activities rather than slides/demonstration. Handouts for taking notes and working along with discussion will be provided to attendees. 30 minutes

**Keywords:** Information literacy, instruction, ACRL Framework, backward design, teaching, health sciences

Monday, May 21, 2018, 10:30 AM – 11:55 AM
Room: Embassy F (International Tower, Exhibit Level)

---

# Special Content Session: Second Career Librarians: Transforming Previous Career Experiences into Librarianship Gold! Tips from Firsthand and Leadership Perspectives

**Moderator: Jessica E. DeCaro**

### Second Career Librarians: Transforming Previous Career Experiences into Librarianship Gold! Tips from Firsthand and Leadership Perspectives

**Tamara M. Nelson**
Instructor/Reference Librarian, Rowland Medical Library, Jackson, Mississippi

**Shalu Gillum, AHIP**
Head of Public Services, Harriet F. Ginsburg Health Sciences Library, Orlando, Florida

**Sharon Bailey, AHIP**
Manager, Library Services and Archives, Toronto, Ontario, Canada

**Michael Lindsay, AHIP**
Serials/E Resources Librarian & Associate Professor, University of Tennessee Graduate School of Medicine, Knoxville, Tennessee

**Helen-Ann Brown Epstein, AHIP, FMLA**
Informationist, Health Sciences Library, Mt Laurel, New Jersey

**Lori E. Harris**
Associate Director for the Donald C. Harrison Health Sciences Library and the Henry R. Winkler Center for the History of the Health Professions, University of Cincinnati, Cincinnati, Ohio

**Program Description:** Attend this panel discussion to learn how to market yourself and demonstrate your value to a hiring committee. Panel participants include librarians who came to the field from non-library backgrounds, including law, publishing, and insurance sales. See how they marketed themselves, leveraged their backgrounds to support their new careers in medical librarianship, and the factors that impact their current librarianship practice. Also, hear from current and former library directors with experience reviewing resumes to gain perspectives on how a candidate with diverse work experience can positively influence a work

environment.

**Keywords:** second career librarians
transformation
adapting
marketing
transferable skills
employment
leadership

Monday, May 21, 2018, 10:30 AM – 11:55 AM
Room: International Ballroom North (International Tower, Ballroom Level)

---

# Special Content Session: Design, Play, Learn: A Special Content Session to Design a Game for Database Instruction

**Moderator: Eugenia Liu**

## Design, Play, Learn: A Special Content Session to Design a Game for Database Instruction

**Rachel Keiko Stark, AHIP**
Health Sciences Librarian, California State University, Sacramento University Library, Sacramento, California

**Eugenia Liu**
Health and Human Services Librarian, Dimond Library, Durham, New Hampshire

**Nicole Capdarest-Arest, AHIP**
Head, Blaisdell Medical Library, University of California, Davis, Sacramento, California

**Program Description:** This Special Content Session utilizes a flipped classroom approach to engage interested information professionals in the gamification process, and will provide an active learning opportunity for participants to collectively design a game for use in health sciences information instruction. Participants will be asked to work in small groups to design a game prototype, which will then be played during the session. A finalized version of the game will be agreed on by the group. After the session, participants will be provided with a copy of the game designed during the session along with instructions for use in the classroom. Participants will also be provided with a suggested measurement for assessing the effectiveness of the game when used in their instruction sessions.

**Keywords:** Instruction, databases, gamification, flipped classroom, active learning, game design

Monday, May 21, 2018, 1:00 PM – 2:25 PM
Room: Embassy B (International Tower, Exhibit Level)

---

# Session: Clinical Support 2 (CS-2)

**Moderator: Margaret (Margo) Coletti**

## Identifying the Role of the Medical Librarian in Clinical Competency Entrustment


**Nancy E. Adams**
Director of Foundational Medical Sciences, Penn State College of Medicine, Hershey, Pennsylvania

**Heather Collins, AHIP**
Assistant Director, Research and Learning Department, A.R. Dykes Health Sciences Library, Kansas City, Kansas

**Iris Kovar-Gough, AHIP**
Health Sciences Librarian, Michigan State University Libraries, East Lansing, Michigan

**Elizabeth R. Lorbeer, AHIP**
Chair, Department of the Medical Library, Western Michigan University Homer Stryker M.D. School of Medicine Library, Kalamazoo, Michigan

**Joey Nicholson**
Education and Curriculum Librarian, NYU Health Sciences Library, New York, New York

**Judy M. Spak**
Assistant Director, Research and Education Services, Cushing/Whitney Medical Library, New Haven, Connecticut

**Emily Brennan**
Research and Education Informationist, Medical University of South Carolina (MUSC) Library, Charleston, South Carolina

**Rikke Sarah Ogawa, AHIP**
Director, Louise M. Darling Biomedical Library and Science and Engineering Library, UCLA, Los Angeles, California

**Neil Rambo**
Director, NYU Health Sciences Library, New York, New York

**Kelly Thormodson**
Director, Library Resources, School of Medicine and Health Sciences University of North Dakota, Grand Forks, North Dakota

**Megan von Isenburg, AHIP**
Associate Dean, Medical Center Library, Durham, North Carolina

**Objectives:** The AAHSL Task Force on Competency-based Medical Education will present the conclusions from their survey of medical schools and the work of librarians involved in teaching the Core Entrustable professional activities in the curriculum.

**Methods:** In May 2014, the Association of American Medical Colleges (AAMC) published the Core EPAs for Entering Residency, a 5-year pilot program to assess medical students based on competencies. In the spring of 2016, AAHSL formed a task force to evaluate how the new Core EPAs could affect the engagement of librarians in medical school curriculum development, teaching, and assessment. The task force charge included identifying libraries participating in Core EPA activities and developing a methodology to characterize the nature of their participation. A survey was developed in Qualtrics and sent to medical schools via the AAHSL listserv in fall of 2016. Results were analyzed using SPSS and with the expertise of a statistician.

**Results:** The results are leading to follow up interviews with exemplars that will commence in the fall of 2017. Generally, librarians are involved in teaching and assessment of EPA 7 (forming a clinical questions and retrieving evidence) and to a lesser degree in EPAs 9 (interprofessional collaboration) and 6 (oral presentations), but overall involvement and awareness of EPA domains remains low. Participation varied greatly and libraries are assessing theses skills to a lesser extent than teaching them.

**Conclusions:** Trends identified in the data can help medical librarians plan how they could use EPAs, specifically EPA 7, to identify areas for librarians to strategically impact medical school curricula. As EPAs become a standard set of competencies for all medical schools, beyond the 10 pilot schools and early adopters, they can be a powerful tool to increase information literacy activities in a curriculum and lead to more teaching and assessment by librarians. Variability in responses notwithstanding, the data from the survey demonstrates that librarians are interested in how EPA’s will change their role in medical education.

**Keywords:** information literacy, medical, entrustable professional activities, competency-based education, AAHSL, AAMC

## Library Tools at the Nurses’ Station: Exploring Health Information-Seeking Behaviors and Needs of Nurses in a War Veterans Nursing Home

**Gail Kouame**
Chair, Research & Education Services, Greenblatt Library, Augusta, Georgia

**Steph Hendren**
Nursing Information Librarian, Greenblatt Library / Research and Education Services, Augusta, Georgia

**Frances Yang**
Assistant Professor, Department of Population Health Sciences, Augusta, Georgia

**Objectives:** Analyze the information seeking practices and identify the information and education needs of nurses in a war veterans nursing home affiliated with a nearby university. Develop an online toolkit for use at the nurses’ stations to meet nurses’ health information needs. Supply laptop computers to provide access to the online toolkit at each of the nurses’ stations.

**Methods:** Investigators met with nurse leaders and with administrators at the war veterans nursing home to establish the best approach to set up and administer the proposed study. Nurse leaders also suggested commonly-encountered clinical situations as well as information resources for inclusion in the study questionnaire. Investigators used a two-pronged approach to track nurses' information seeking behaviors and determine their information needs. IRB approval was obtained for human subjects research. Investigators administered an online consent form and questionnaire to nurses at the skilled nursing facility. In-person observations of nurses at each nurses' station and work shift (days, evenings, and nights) were also performed. Resulting data was compared to determine how nurses' self-reported data corresponded with investigator observations. A consulting gerontologist/epidemiologist was employed to analyze the data. Funding for this project was secured through an NN/LM Health Information Outreach Award.

**Results:** Nurses self-reported seeking information prior to: assessing patient clinical status (52%), consulting the nurse manager (29%), and calling for treatment orders (37%). The observational data recorded nurses seeking information for assessing patient clinical status (16%), consulting the nurse manager (83%), and calling for treatment orders (66%).
Nurses self-reported feeling most confident in assessing falls and pain, followed by cognitive ability and cardiovascular health, and medication adherence. Issues most frequently encountered during observations were falls, cognitive ability/dementia, and use of antibiotics. Nurses both reported and were observed to consult colleagues most frequently, followed by drug handbooks, and relying on nursing experience.

**Conclusions:** Nurses in skilled nursing facilities benefit from ready online access to current drug handbooks as well as information resources surrounding commonly encountered clinical issues and stated needs. An outcome of this project is an online toolkit using a LibGuide, created specifically for this purpose. Laptop computers were also purchased and installed at each of the four nurses' stations in the veterans' home. Due to the small sample size of this study, it can be used as a pilot project to inform possible future studies in other skilled nursing facilities.

**Keywords:** Nursing; Information Needs Assessment: Library Research; Skilled Nursing Facilities

## Mind the Gap: Identifying What Is Missed When Searching Only the Broad Scope with Clinical Queries

**Edwin V. Sperr, Jr., AHIP**
Clinical Librarian, Graduate Medical Education, AU/UGA Medical Partnership, Athens, Georgia

**Objectives:** PubMed Clinical Queries are subdivided into “broad” and “narrow” versions, tuned to maximize retrieval sensitivity and specificity respectively by using similar but different sets of terms. This study seeks to determine whether narrow results are always a subset of broad results, and if not, quantify what is missed using only the broad search scope.

**Methods:** For each of the five sets of PubMed Clinical Query filters, “broad” and “narrow” versions were searched against the PubMed database. Citations found using the narrow scope but not the broad were counted, and a degree of difference between the two sets was computed. As an assessment of whether these results might pertain in more typical use, this process was repeated using a set of clinically-relevant terms, ranging from fairly wide in scope, such as “heart disease”, to relatively narrow, such as “Vitamin D Deficiency Anemia”.

**Results:** Aside from “Therapy”, searches using each filter missed potentially relevant citations when searching Filter/Broad alone. The greatest difference between the sets Filter/Broad and Filter/Narrow NOT Filter/Broad were seen when searching “Prognosis”. The ratio of items uniquely retrieved by Prognosis/Narrow to those found by using Prognosis/Broad ranged from 0.073 in all PubMed to 0.102 when searching using clinical terms. The degree of difference was less dramatic for “Diagnosis” (all PubMed: 0.037, clinical term set: 0.008), “Clinical Prediction Guides” (all: 0.026, clinical: 0.014) and “Etiology” (all: 0.008, clinical: 0.008).

**Conclusions:** For “Prognosis”, “Diagnosis”, “Etiology” and “Clinical Prediction Guides”, these differences in retrieval between Filter/Broad and Filter/Narrow NOT Filter/Broad mean that there are many potentially relevant citations that are missed when searching the broad scope alone. Users interested in retrieving as many relevant citations as possible should likely consider ORing the broad and narrow scopes together.

**Keywords:** PubMed, bibliometrics, database searching

## Partnering with Performance Improvement to Transform Patient Care through Care Redesign

**Megan Van Noord**
Research & Education Librarian, Medical Center Library & Archives, Durham, North Carolina

**Jamie Conklin**
Health Sciences Librarian and Liaison to Nursing, University of North Carolina at Chapel Hill Health Sciences Library, Chapel Hill, North Carolina

**Leila Ledbetter**
Research & Education Librarian, Medical Center Library & Archives, Durham, North Carolina

**Beverly Murphy, AHIP, FMLA**
Assistant Director, Communications & Web Content, Medical Center Library & Archives, Durham, North Carolina

**Megan von Isenburg, AHIP**
Associate Dean, Medical Center Library, Durham, North Carolina

**Objectives:** This paper describes how librarians have been embedded in care redesign teams to ensure integration of the best available evidence into large-scale quality improvement projects. We will share how librarian involvement in these multidisciplinary teams has positively impacted both health care and library services.

**Methods:** Since 2014, librarians have participated in 19 care redesign teams that last approximately 16 weeks each and target specific procedures or conditions, including obstetric delivery, renal failure, pediatric cardiothoracic surgery, and operating room utilization. Librarians embed in teams, identify information needs, and help fill requests regarding leading evidence and best practices around the focus areas. Statistics have captured the number of requests and resulting deliverables (e.g. citations and full-text articles). This paper will discuss what care redesign is, workflow, challenges, and tips for getting involved in quality improvement efforts at other institutions.

**Results:** Librarians have filled more than 160 requests and hand-selected and delivered 1,246 articles and 1,463 citations to fulfill them. Librarians have spent more than 272 hours searching, reviewing citations, and summarizing answers to questions (when needed). On average, most requests took one hour to fill, though for some teams, this average was higher at up to 3.5 hours per request. Tips for getting involved in system-wide quality improvement include thinking beyond traditional patron groups, collecting in new areas, and being a proactive advocate for the best evidence.

**Conclusions:** Librarian participation in care redesign and quality improvement efforts ensures that clinicians see the best evidence for use in patient care. Librarians can leverage their expertise to identify information needs of hospital units, formulate questions, perform searches, and lead efforts in sharing and accessing information. For librarians, participation in Care Redesign led to higher satisfaction and commitment to the

health system mission.

**Keywords:** evidence-based practice, care redesign, quality improvement, hospital partnerships

## The Role of the Library in Promoting Changes in the Health Care Delivery System through Systematic Assessment

**Kelsey Leonard Grabeel, AHIP**
Assistant Director of the Health Information Center / Assistant Professor, Preston Medical Library / Health Information Center, Knoxville, Tennessee

**Emily Tester**
Graduate Research Assistant, Health Information Center / Preston Medical Library, Knoxville, Tennessee

**Jennifer Russomanno**
Continuing Education Coordinator, Continuing Ed. & Professional Dev., Knoxville, Tennessee

**Eric Robert Heidel**
Associate Professor, Department of Surgery, Division of Biostatistics, Knoxville, Tennessee

**Sandy Oelschlegel, AHIP**
Retired Director / Associate Professor, Health Information Center / Preston Medical Library, Knoxville, Tennessee

**Objectives:** To implement a targeted health literacy initiative led by medical librarians that would serve as a catalyst for promoting changes in the healthcare system at an academic medical center.

**Methods:** A rigorous approach was taken to the library led research project, beginning with applying for and receiving exemption from Institutional Review Board (IRB) and the participation of a statistician in the research design. Librarians chose the Health Literacy Environment of Hospitals and Health Centers (HLEHHC) for the assessment. Each of the five HLEHHC tools were applied sequentially to the environment of the medical center. Data for each assessment was collected over a six-month time period and then analyzed by the research team using statistical analysis and qualitative research methods. The scope of the project included the following: analyzing 150 print patient education documents, interviewing 298 patients, feedback from 7 navigators, measuring policies and protocols knowledge of 77 administrators, and assessing technology using an authoritative source.

**Results:** Overall, the medical center's aggregate score ranked in the highest category, with a score of 218.57, which translates to “continue to monitor and eliminate literacy-related barriers.” The individual category scores were as follows: Navigation Rating: 64.71, Print Communication Rating: 53.94, Oral Communication Rating: 16.91, Technology Rating: 47, Policies and Protocols Rating: 36.01.

**Conclusions:** Although three of the ratings scored in the highest category, these ratings were on the lower end of that range. In addition, two categories scored in the middle category indicating opportunity for improvement. Therefore, researchers reviewed each tool on a question by question basis to reveal more granular information on where there are opportunities to improve the health care environment for low health literate patients. Library leadership in this project has increased the visibility with medical center administration and created opportunities for library participation in improvement plans based on our results.

**Keywords:** health literacy, organizational health literacy, assessment, patient engagement, consumer health

Monday, May 21, 2018, 1:00 PM – 2:25 PM
Room: Embassy C (International Tower, Exhibit Level)

---

# Session: Evidence-Based Practice 1 (EBP-1)

**Moderator: Stevo Roksandic, AHIP**

## Best Practices in Teaching Evidence-Based Medicine: A Qualitative, Multi-Site Study

**Catherine Pepper**
Associate Professor and Library Field Services Coordinator, Texas A&M University, Austin, Texas

**Esther E. Carrigan, AHIP**
Associate Dean and Director, Medical Sciences Library, College Station, Texas

**Objectives:** Evidence-Based Medicine (EBM) is a common component of undergraduate medical education, and is a required competency for entering medical residencies. However, there is no standardized method of teaching EBM; therefore, it is currently unknown how well prepared medical students are for searching and critically appraising the research literature for clinical questions. This study investigated effective educational approaches in teaching EBM.

**Methods:** Using a grounded theory approach, semi-structured interviews were conducted with librarians and faculty involved in teaching EBM at schools of medicine and other health sciences. Seventeen institutions were initially targeted, using three criteria: (1) At least two NNLM regions; (2) Diversity in size, administrative structure, history in teaching EBM, and type/extent of librarian involvement; (3) Clustered in a geographic region to allow visits within a two-month timeframe. Librarian contacts were asked to help recruit interviewees. Questions included: Where/when in the curriculum are EBM topics introduced (e.g., PICO, literature searching, and critical appraisal of evidence)? How often are these concepts reinforced? With sophisticated tools such as UpToDate available now, is there still value in students learning EBM skills? What specific methods are used? How are students assessed on their learning of these concepts? How do clinical faculty receive training in EBM?

**Results:** Ninety-six interviews were conducted. An almost equal number of interviews were recorded from medical librarians and from faculty from medical, nursing, pharmacy, and allied health schools. Data collection concluded in October 2017. Major themes identified so far include assessment methods, strategies for increasing students' receptivity to EBM instruction, active learning implementations, and varying levels of librarian involvement in EBM instruction. Final data analysis will be completed by February 2018 and results will be reported at the meeting.

**Conclusions:** Teaching evidence-based medicine is a "hot topic" in health sciences education. Perspectives on teaching EBM--or evidence-based practice (EBP)--and methods for teaching it, vary among health sciences disciplines and between instruction librarians and health sciences faculties. Each perspective and method can be considered in crafting a "blueprint" or "template" for teaching this competency, which could be adapted for use in various contexts and environments. A final Conclusion, based on the final data analysis, will be presented

at the meeting.

**Keywords:** Evidence-Based Medicine; Evidence-Based Practice; EBM; EBP; Health Sciences Education; Librarians;

## Giving Us Our Due: Are Librarians Acknowledged for Their Work in Nursing Systematic Reviews?

**Michelle Lieggi, AHIP**
Clinical Research Librarian, Fishbon Library, San Francisco, California

**Objectives:** Organizations such as the Cochrane Collaboration and the Institute of Medicine recommend librarian participation in systematic reviews. The Joanna Briggs Institute (JBI), a nursing research organization producing systematic reviews, specifically instructs its reviewers to consult a librarian. This study examines if librarians are consistently acknowledged for their work and/or given authorship in systematic reviews in nursing.

**Methods:** This preliminary study is an analysis of acknowledgement and authorship rates for librarians in systematic reviews in nursing. The author searched PubMed for systematic reviews from the top 75 nursing journals ranked by impact value (as reported by Journal Citation Reports/Science Citation Index), and from JBI Database of Systematic Reviews and Implementation Reports, from January 2015 through August 2017. Quantitative or qualitative studies in English were considered if they included “systematic review” in the title and/or abstract. After filtering out irrelevant studies and other (not systematic) reviews, 383 systematic reviews were retrieved. These were examined as to whether they noted a librarian in the acknowledgements and/or were co-authored by a librarian.

**Results:** 45 of the 75 nursing journals searched included systematic reviews. 1/3 of the reviews retrieved came from JBI. For all reviews retrieved, about 16% acknowledged and/or had a librarian co-author. 7% were co-authored by a librarian. Authorship rates were the same for both the reviews from the nursing journals and from JBI (7%). But a higher percentage of the JBI reviews mentioned a librarian in the acknowledgements (21% versus 4% in the nursing journals).

**Conclusions:** This study is limited by a narrow search of nursing journals and one systematic review resource. The author assumes librarians assist with many more reviews than acknowledged and the results don’t suggest a lack of librarian participation. The results do suggest that librarians aren’t consistently acknowledged for their work on systematic reviews in nursing. Future study will examine a broader set of systematic reviews, as well as investigate why acknowledgement and authorship rates are so low. Understanding why librarians aren't getting their due could help transform their participation in all systematic reviews going forward.

**Keywords:** systematic reviews, nursing, authorship, acknowledgement, analysis

## Empowering Nurses and Mentors with an Innovative, Online Roadmap for Evidence-Based Practice

**Roy E. Brown, AHIP**
Research and Education Librarian / Nursing Liaison, Tompkins-McCaw Library for the Health Sciences, VCU Libraries, N. Chesterfield, Virginia

**Objectives:** A librarian and nursing colleagues at an academic medical center noted considerable variability in understanding of evidence-based practice (EBP) and quality of EBP projects. To address the issue, the Online

EBP Portfolio (OEBPP) Tool was created. This proposal aims to evaluate changes in nurses' attitudes, practice behaviors, and knowledge pertaining to EBP as a result of using the OEBPP Tool.

**Methods:** In order to increase use and to standardize EBP knowledge and practice across the institution, the OEBPP tool was integrated into all EBP instruction and the nurse residency program within an academic health system. The tool provides information at the point of need at each step in the EBP process. The impact of the tool was evaluated using Upton and Upton's EBP questionnaire. The questionnaire is a self-assessment that examines the attitudes, practice behaviors, and knowledge and skills associated with EBP. The questionnaire was given at three points in the EBP process: at the beginning of an EBP project before any action is taken, at the midpoint when recommendations for changes are made, and at the completion of an EBP project. Responses to the questionnaire were compared between each of the data collection points.

**Results:** Approximately 80 nurses took the initial survey and 60 took the second survey. At this point in the implementation of the tool, no one has responded to the third survey. When comparing the results of the initial survey results and those of the one given at the midpoint, a positive correlation was found between the use of the online tool and all three of the scales of the questionnaire. There is also anecdotal evidence that the overall quality of the completed EBP projects has improved by standardizing knowledge and practice at the institution.

**Conclusions:** The OEBPP Tool has been effective in helping increase the comfort and knowledge of nurses as it relates to EBP. The tool served as an effective mechanism to enable the librarian to integrate more fully into the EBP processes and to provide direct value to hospital administration. It could also serve as an effective tool for librarians working with nurse residency for the work librarians do with nurse residency programs where new nurses come from many different and there is a great deal of variance in the knowledge base of those in the program by providing a framework for instruction.

**Keywords:** Evidence-Based Practice, Nursing, Hospitals, Program Evaluation, Embedded Librarianship, Liaison

## Is This a Systematic Review? A Librarian Assessment of Adherence to PRISMA Guidelines in Published Systematic Reviews

**Jennifer E. Dinalo**
Information Services Librarian, USC, Los Angeles, California

**Lynn Kysh**
Clinical & Research Librarian, Norris Medical Library, Los Angeles, California

**Amy J. Chatfield**
Information Services Librarian, Norris Medical Library, Los Angeles, California

**Objectives:** 2009's Preferred Reporting Items for Systematic Reviews and Meta-Analyses (PRISMA) guideline provides 27 items to report in systematic review manuscripts and suggests appraisal methods for editors and reviewers of such manuscripts. What proportion of publications labeled as systematic reviews within pediatric oncology adhere to the PRISMA guideline? How are journals incorporating PRISMA in policies and instructions to authors and reviewers?

**Methods:** Eight items within PRISMA with relevance to librarian-provided services for systematic reviews were selected for analysis. Rubrics to measure the degree of adherence to each item were created; items could be coded as Met, Partially Met, or Not Met. Medline, Embase, and Cochrane were searched for articles that covered pediatric oncology topics, stated they were systematic reviews, and published after 2009. Each article was independently coded by two reviewers. Editorial policies relating to submission/acceptance and peer review of systematic review articles were obtained from journals that published articles used in the analysis. Two

reviewers independently examined and coded each editorial policy for adherence. Adherence to each item by both journals and published articles were examined and compared. It is anticipated that published articles will partially meet PRISMA guidelines, and journal instructions/policies will not include the items PRISMA considers important.

**Results:** Each item from PRISMA was examined thoroughly, and a detailed instrument to code the adherence to each criterion was developed. Recognizing that this instrument could be re-used by other investigators, a test set of systematic reviews on tuberculosis was gathered and the instrument was tested for both usability and inter-rater reliability. Upon examination of the volume of systematic reviews in pediatric oncology, criteria were refined to focus on leukemia in children ages 0-18. 753 articles were identified; title and abstract screening reduced this to 303 articles; full-text screening to develop the final set of articles to code is ongoing.

**Conclusions:** PRISMA guidelines provide guidance on the structure and content of a systematic review report. Published systematic reviews vary widely in the types and degrees of adherence to PRISMA, requiring a more-complex-than-anticipated instrument in order to capture what is being presented and what is being omitted.

**Keywords:** systematic reviews, study design, process improvement, evidence-based medicine, expert searching

Monday, May 21, 2018, 1:00 PM – 2:25 PM

Room: Embassy D (International Tower, Exhibit Level)

---

# Session: Information Management 2 (IM-2)

**Moderator: Sheila W. Green**

## A Pilot Project to Facilitate the Development of Data Services at Health Sciences Libraries

**Alisa Surkis**
Assistant Director for Research Data and Metrics, NYU Health Sciences Library, New York, New York

**Kevin B. Read**
Knowledge Management Librarian, NYU Health Sciences Library, New York, New York

**Objectives:** Data services development in health sciences libraries (HSL) has been hampered by a lack of focused training materials for HSLs, insufficient researcher buy-in, and lack of an easily adaptable curriculum. We sought to develop data services at six HSLs by building a knowledge base, facilitating connections with researchers, establishing effective outreach strategies, and providing well-tested, adaptable teaching materials.

**Methods:** We implemented a data services pilot program based on successful services developed in our academic HSL. The program components were: 1) eight online training modules about research data management (RDM) and the culture and practice of research; 2) a data interview template for interviewing researchers regarding their attitudes towards data and data management; 3) a teaching toolkit consisting of slides, script, and evaluation materials to be used for in-person teaching; and 4) strategies and promotional materials for developing a data class series as a collaboration between the library and others at the institution with data expertise. Regular meetings with pilot participants served to provide ongoing feedback and build community. The program was evaluated based on pre and post qualitative interviews with participating librarians, the number of data interviews conducted and data classes offered, and researcher evaluations of RDM classes.

**Results:** Each of the pilot libraries conducted data interviews, and completed between 6 and 15 interviews with researchers from a range of disciplines. Each library used the teaching toolkit to teach at least one introductory RDM class, with some participants teaching up to four RDM classes. Evaluation data was extremely positive across all of the classes taught. Two of the institutions offered data series during Love Data Week.

**Conclusions:**
The components of the pilot program successfully translated to use in other institutions. The pilot program proved to be a very effective means of jump starting the development of research data management services in health sciences libraries, and

**Keywords:**
research data management

data services

data education

community building

## Standardizing Documentation and Curation Practices to Improve Reuse of Data from Structured Searches and Systematic Reviews

**Whitney A. Townsend**
Informationist, Taubman Health Sciences Library, Ann Arbor, Michigan

**Mark P. MacEachern**
Informationist, Taubman Health Sciences Library, Ann Arbor, Michigan

**Marisa L. Conte**
Assistant Director, Research and Informatics, Taubman Health Sciences Library, Ann Arbor, Michigan

**Objectives:** Data sharing is becoming increasingly important to funding agencies, researchers, and publishers.To develop best practices for sharing data from structured or systematic literature searches via open repositories, including recommendations for documentation and a minimum set of standard metadata.

**Methods:** Librarians who contribute expert searches to research projects should ensure that detailed information about these searches, including results sets, are discoverable and reusable by others. While some journals publish search-related information as appendices or online supplements, this is generally limited to the search strategy and may not be available permanently or without a subscription. Although librarians and other researchers who conduct searches may keep detailed records, providing access to these locally stored files is difficult and likely impermanent.

To address these issues, [redacted] sought to develop standards for data description and deposit of bibliographic datasets. We identified datasets resulting from systematic reviews or other structured, expert searches, developed a template for descriptive documentation, and a standard minimum set of metadata, and applied both to these datasets. After curation, the datasets were archived in [redacted], an institutional data repository.

**Results:** If accepted, results will be provided in February 2018

**Conclusions:** If accepted, results will be provided in February 2018

**Keywords:** data, repository, metadata, repositories

## Standards for Literature Searching: Validation of a Proposed Model for Search Methods

**Brooke Ballantyne Scott**
Librarian, Royal Columbian Hospital, New Westminster, British Columbia, Canada

**Jackie Macdonald**
Privacy Officer, Nova Scotia Health, Bridgewater, Nova Scotia, Canada

**Susan Baer**
Director of Libraries & Archives, Health Sciences Library, Regina, Saskatchewan, Canada

**Ashley Farrell, AHIP**
Medical Librarian, Cancer Care Ontario, Toronto, Ontario, Canada

**Marcus Vaska**
Librarian, Alberta Health Services (AHS), Knowledge Resource Service, Calgary, Alberta, Canada

**Pat Lee**
Retired, None, Toronto, Ontario, Canada

**Danielle Rabb**
Information Specialist, CADTH, Ottawa, Ontario, Canada

**Objectives:** Through three years of iterative literature review, research and development, a pan-Canadian group of librarians have outlined model search methods for handling each of five progressive levels of search complexity, supported by a lexicon glossary for mediated literature searching collected from the literature. This online questionnaire seeks to compare what mediated searchers “should do” with what they actually do.

**Methods:** As Part 2 of a multi-methods research study, our online questionnaire asked mediated searchers (n=104) to choose one of 13 exemplary searches and describe how they handled a recent similar search. Responses were analyzed by search level in subgroups defined by mediated searchers’ years of experience searching, and number of searches completed weekly. Data were then represented graphically to illustrate congruency within and between groups, and between questionnaire respondents and the model. Incongruities were flagged and addressed through reflexivity and consultation.

**Results:** The results of our online questionnaire were analysed and any incongruities between our placement and inclusion of certain steps within the search stages and the questionnaire responses were debated as a group. Preliminary questionnaire analysis suggests that mediated searchers may not always tailor search methods to search complexity, possibly “over-searching” or “under-searching” as a result. Inconsistencies within and between subgroups as defined by years of experience suggest inconsistencies in search education/training. Some respondents indicated uncertainty with search terms.

**Conclusions:** This questionnaire study highlights inconsistencies in approaches used by mediated search professionals. Although client satisfaction with mediated search services has been reported in research literature, professional searchers have worked for over fifty years without either a consistent approach to executing different levels of search complexity, or a framework against which a search service performance may be measured. Within health services, librarians are one of the few professions that operate without practice standards. Our proposed model of search methods provides a foundation for further work required to more closely align health librarians with other health professionals who work to performance standards.

**Keywords:** Literature searching, mediated searching, practice standards, questionnaire study

## Tracking and Reporting Faculty Scholarly Productivity and Impact

**Roxann W. Mouratidis, AHIP**
Scholarly Communications Librarian, Maguire Medical Library, Tallahassee, Florida

**Susan Epstein, AHIP**
Systems Librarian, Maguire Medical Library, Tallahassee, Florida

**Robyn Rosasco, AHIP**
Public Services Librarian, Maguire Medical Library, Florida State University College of Medicine, Tallahassee, Florida

**Martin Wood, AHIP**
Director, Maguire Medical Library, 32306-4300, Florida

Objectives: The Quality Enhancement Review (QER) is a process by which academic programs recognize accomplishments related to faculty performance. The library's responsibilities for the Biomedical Sciences QER were:

1) To capture and record the number of articles, books, and citations attributed to faculty within a 5 year period.
2) To calculate the impact factor for each faculty member's lifetime publication record.

Methods: Using our university's CV database, we generated a list of faculty names and their publications produced during 2012-2015. This list was compared to a report of faculty research productivity provided by an analytics company called Academic Analytics. We corroborated this data by running searches in library-licensed and freely available publication databases.

Web of Science's collection of databases was the most comprehensive and efficient resource available to us as we determined the number of journal articles and citations attributed to each faculty member, and then obtained the corresponding lifetime H-index score.

Since many of our faculty had common names, we created search strategies involving elements of their name, affiliations, and cities of employment. We also determined the number of faculty-authored books reported in the CV database during 2006-2015, and compared this data with author searches using the British Library's online catalog.

Results: The number of journal articles, books, and citations did not vary substantially depending on the source; however, there were discrepancies. These discrepancies are likely the result of the variety of practices and standards that may be used by authors, publishers, and literature databases.

For example:
1) Authors may or may not be consistent in how they provide their names, affiliations, and other identifiers to publishers.
2) Publishers may misspell or be inconsistent in how they list author names/affiliations/identifiers on publications.
3) Databases index a subset of scholarly literature depending on their specialization and inclusion criteria.

Conclusions: Faculty involvement in maintaining a record of their publications can improve the accuracy and efficiency of tracking publications. Although many faculty had a profile established with a research identifier service, in many cases these profiles were not current, complete, or consistently utilized across the department.

Review processes like the QER often call for a specific date range for faculty accomplishments, which may be arbitrary and will not showcase the full body of work of individual faculty members. We expanded this time frame

to calculate each researcher's H-index over the lifetime of their careers to better showcase faculty accomplishments and expertise.

## Wolf in the Fold: Quality of Databases and Digital Repositories Post-Beall's List

**Catherine A. Smith**
Associate Professor, University of Wisconsin-Madison, Madison, Wisconsin

**Askar Safipour Afshar**
PhD Student, The Information School, Madison, Wisconsin

**Objectives:** In January 2017, academic librarian Jeffrey Beall's controversial and impactful List of "predatory" publishers and standalone journals went suddenly dark. The publishers and journals, however, remain. How many "predatory" journals in the health sciences fields have permeated the full-text databases and online repositories on which researchers and librarians depend?

**Methods:** In November 2016, the researchers investigated each of the 248 publishers named on Beall's List for a minimum of 3 consecutive years and found a total of 74 listing titles in the health sciences. Analysis proceeded in 2 phases: (1) Journal selection: Each publisher website was harvested, resulting in a set of 1397 health sciences journals. (2) Article selection: One article from each journal was identified and checked for its presence in 6 digital locations: PubMed; PubMed Central; Google Scholar; Web of Science; Scopus; and the Primo academic library discovery service licensed by the researchers' home institution. In addition, the researchers examined characteristics of these "predatory" journals: presence in Ulrich's Periodicals Directory; topic according to NLM classification; indexing and abstracting services (both advertised and actual); and articles with similar or identical content appearing in both "predatory" and non-"predatory" journals.

**Keywords:** Scholarly communication; open access publishing; health science databases; citation indexes

Monday, May 21, 2018, 1:00 PM – 2:25 PM
Room: Embassy A (International Tower, Exhibit Level)

---

# Session: Leadership and Management 2 (LM-2)

**Moderator: Norice Lee**

## Librarian-Faculty Collaboration as Reflected by Articles Published in the Journal of the Medical Library Association


**Katherine G. Akers**
Biomedical Research and Data Specialist, Shiffman Medical Library, Detroit, Michigan

**Molly Higgins**
Reference and Digital Services Librarian, Library of Congress, Washington DC, District of Columbia

**Jennifer A. DeVito**
Director of Access & User Services, Frank Melville Jr. Memorial Library, Stony Brook, New York

**Sally Stieglitz**
Digital Learning and Instruction Librarian, Adelphi University, Garden City, New York

**Robert Tolliver**
Head, Science and Engineering, Stony Brook University Libraries, Stony Brook, New York

**Clara Y. Tran**
Science Librarian, Frank Melville Jr. Memorial Library, Stony Brook, New York



**Objectives:** A recent examination of several health sciences and STEM librarianship and education journals revealed that the Journal of the Medical Library Association publishes the largest number of articles jointly co-authored by librarians and faculty members. Here, we examined these JMLA articles more closely to identify the most frequent areas of librarian-faculty collaboration and the most common disciplines of faculty collaborators.

**Methods:** We examined all research articles and case studies published in the Journal of the Medical Library Association between 2002 and 2017 and included articles that were authored by at least one practicing librarian or informationist and one faculty member from any discipline. The following data were extracted from each included article: title; publication year; number of librarian co-authors; number and disciplines of faculty co-authors; number and roles of other co-authors; and article topic (e.g., teaching evidence-based medicine, information-seeking by healthcare providers, information-seeking by healthcare consumers, medical database information retrieval, systematic review methodology). We will present descriptive statistics for extracted data and perform inferential statistical analysis to identify potential temporal trends.

**Results:** Analysis currently in progress.

**Conclusions:** This analysis will provide information on the most productive areas of collaboration between health sciences librarians and faculty members and the disciplines in which faculty members may be more likely to view librarians as potential collaborators. Our results are expected to underscore the role of the health sciences librarian as a fully-fledged partner in health sciences education, research, and information dissemination.

**Keywords:** collaboration, publishing

## MLA Competency 2 “Organizes Resources” Indicator: How Can Medical Librarians Level Up from Basic to Expert?

**Michelle B. Bass, AHIP**
Population Research Librarian, Lane Medical Library, Stanford, California

**Thea S. Allen**
Metadata Librarian Manager, Lane Medical Library, Stanford, California

**Ariel Vanderpool**
Resource Acquisitions Specialist, Lane Medical Library, Stanford, California

**Nicole Capdarest-Arest, AHIP**
Head, Blaisdell Medical Library, University of California, Davis, Sacramento, California

**Objectives:** Given revised MLA competencies, medical librarians should be able to develop skills in areas where they may want to “level up” in domain areas. For this project, the authors reviewed available curricula for 30 ALA-accredited programs, as well as professional development and informal opportunities for skill development around Competency 2 (Information Management) Performance Indicator on organizing resources.

**Methods:** Using an Excel database and descriptive statistics, library school and continuing education (CE) programming around classification/metadata was collected and examined. The authors reviewed the 30 ALA-accredited library programs with Health Sciences Librarianship/Health Informatics as areas of concentration/career pathways. Program websites were reviewed or personal contact was made with each school to evaluate what courses are available on metadata standards and development and which, if any, of such courses are required. The authors also reviewed CE course listings on the Web by MLA, ALA, ACRL, NASIG and AMIA to identify CE options on these topics. Courses offered in conjunction with conferences were not included for purposes of this project. Lastly, the authors surveyed academic health science library directors on metadata/cataloging librarian employment and identified opportunities to informally integrate professional learning options in the workplace.

**Results:** Twenty-seven out of 30 schools in our sample require a course with metadata content. Beyond required introductory courses, schools ranged widely in course offerings, offering as few as 2 and as many as 9 courses with metadata-related content. As far as CE, ALA offers many opportunities of varying levels, while other organizations offered fewer, if any, opportunities. Librarians can also leverage workplace opportunities to share expertise. For example, liaison librarians can partner with metadata librarians (either within the library or across units/institutions) to enhance discoverability of content via library websites, thus enhancing liaison understanding of metadata and cataloging.

**Conclusions:** The 2017 MLA Competencies for Lifelong Learning and Professional Success identify vital skills

for medical librarians and provide a foundation for identifying opportunities to grow skills and also for identifying areas for which more educational opportunities may be needed. This project suggests that opportunities for growing skills related to organizing resources (e.g., metadata, classification) are varied and lie with particular schools or other professional groups (e.g., ALA, NASIG). To increase proficiency in the performance indicator for organizing resources, additional MLA CE courses on this topic should be developed and librarians should also look for informal cross-training opportunities.

**Keywords:** Classification; competencies; continuing education; cross-training; metadata; organizing resources; performance indicators

## Path to Leadership: The Career Journey of Academic Health Sciences Library Directors

**Rick L. Fought, AHIP**
Director, University of Tennessee Health Science Center, Memphis, Tennessee

**Mitsunori Misawa**
Assistant Professor, Adult Learning and Adult Education, College of Education, Health, and Human Sciences, Knoxville, Tennessee

**Objectives:** As part of a larger study on leadership in academic health sciences libraries, the authors sought to examine the career journey of academic health sciences library directors to better understand what led them into leadership positions in libraries.

**Methods:** Qualitative phenomenological research was selected for the research design due to its focus on exploring and understanding the meaning individuals ascribe to a particular phenomenon or experience. A part of the interpretivist theoretical perspective, phenomenology searches for the essence of a phenomenon from people’s shared experience of it and works particularly well with phenomena that does not lend itself to easy quantification, like leadership.

The study used purposeful sampling and criterion-based sampling strategies to select its participants. Eleven library directors from academic health sciences libraries at public universities with a RU/VH Carnegie Classification agreed to participate in the study. They also met all other selection criteria for the study. Data were collected through two semi-structured interviews with each participant. The data were later transcribed and coded. Thematic analysis was used to analyze the data from which categories and themes emerged.

**Results:** A major theme that emerged from the data was the participants’ path to library leadership. Each participants’ path was unique, but there were common elements that emerged that are informative as to how they understood their experience as emerging leaders and what it takes to be a leader in libraries. Each participant had a wide range of library experience and/or education, but at some point, when they decided to enter into a leadership position, they became very focused in their preparation for leadership. The participants also shared how important leadership mentors were to them in their journey to becoming leaders.

**Conclusions:** Leadership development and preparedness have been found to be important contributors to leadership effectiveness in other studies. It was encouraging to witness and understand the amount of preparation the study participants did to get themselves ready for their roles as library directors. In particular, it was illuminating to hear how big of a role that mentors and mentoring played in the participants’ development as leaders and their desire to mentor the next generation of library leaders.

**Keywords:** leadership, management, directors, phenomenology, preparation, administration, librarianship

## PubMed Central International (PMCI): Is It Time for an Australasia Member?

**Lisa M. Kruesi**
PhD Candidate, Monash University, Melbourne, Australia, Brighton East, Victoria, Australia

Objectives: To describe the purpose, features and challenges of the PubMed Central (PMC) International members, Europe PMC and PMC Canada. The analysis of PMC International is to inform an investigation on the viability of an Australasia PMC. There is potential for medical and health librarians, to lead and transform scholarly publishing and achieve knowledge management benefits through collaboration with PMC International.

Methods: The research is the basis of a PhD study on the establishment of an open Australasia biomedical repository. Based on action research, the intervention is the Australasia PMC Working Committee, whose role is to provide strategic leadership and advice on the conceptual design, implementation and sustainability of an Australasia PMC; in addition to feedback on any proposed funding and establishment opportunities. The first action cycle identified significant interest in an investigation on an Australasia PMC. This paper reports on a case study that forms the second cycle of the PhD research. Following ethics approval, semi-structured, video interviews were held with principal team members from Europe PMC and PMC Canada. Two PhD supervisors and four PMC Working Committee members screened the interview questions, drafted by the author, an experienced health sciences librarian. The PMC International profile is derived from the interview transcripts.

Results: Set-up in 2007, Europe PMC has developed services to access articles, grants, search tools, an array of export formats, external linking of related resources and the management of ORCID profiles. The European Bioinformatics Institute provides technology infrastructure support for Europe PMC. In contrast, PMC Canada has concentrated its effort to offer bilingual systems to search the archive and to deposit author manuscripts funded by the Canadian Institutes of Health Research, all in support of the Canada Tri-Agency Open Access Policy on Publications. Several factors, including infrastructure and structural changes, have challenged PMC Canada since its launch in 2009.

Conclusions: With over a billion users of the NLM website spread throughout the world, coverage by PubMed and PMC is a powerful means to make research output discoverable and permanently available. With major funders requiring researchers to openly publish their findings, PMC has become a successful means to make the results of research widely accessible. PMC International contributes to the corpus of full-text research available in the NLM's PMC. An investigation of Europe PMC and Canada PMC has contributed greater understanding of the strengths, weaknesses, threats and opportunities for an Australasia PMC.

Monday, May 21, 2018, 1:00 PM – 2:25 PM

Room: Embassy E (International Tower, Exhibit Level)

---

# Session: Lightning Talks 3

**Moderator: Ramune K. Kubilius, AHIP**

## Critiquing and Communicating: Assessing the Impact of a Librarian/Physician-Led Evidence-Based Medicine Project on Medical Student Confidence around Critical Appraisal and Effective Patient Communication


**Sarah Jane Brown**
Clinical Information LIbrarian, University of Minnesota Bio-Medical Library, Minneapolis, Minnesota

**David Power**
Professor, Department of Family Medicine and Community Health, Minneapolis, Minnesota

**Caitlin Bakker, AHIP**
Research Services Librarian, Bio-Medical Library, Minneapolis, Minnesota

**David Frenz**
Clinical Faculty, Department of Family Medicine and Community Health, Minneapolis, Minnesota

**Jim Beattie**
Librarian Consultant, N/A, Minneapolis, Minnesota

**Jonathan Koffel**
Emerging Technology and Innovation Strategist, Bio-Medical Library, Minneapolis, Minnesota



**Objectives:** To assess the impact of a 4-week evidence-based medicine (EBM) and health literacy assignment on medical students' confidence to find, critically appraise and summarize literature and then appropriately communicate it to patients using plain language principles. The results will be used to evaluate and redesign the assignment, a 10+ year long collaboration between librarians and medical school faculty.

**Methods:** Third and fourth-year medical students completing a Family Medicine Clerkship were invited to complete an initial pre-test asking about their confidence applying EBM concepts and their ability to effectively communicate with patients using plain language concepts. The students then attended a small-group session on critical appraisal and health literacy concepts and completed an assignment to find the best evidence to answer a clinical question and write summaries for providers and patients. After completing the summaries, the students completed a post-test asking them to assess their confidence levels at the end of the course as well retrospectively assess their confidence at the beginning of the project. Demographic information, including

medical specialization, was also gathered. Descriptive and inferential statistics were used to assess the magnitude and significance of any change in confidence and potential moderators, and analysis of subgroups was performed.

**Results:** 109 participants responded to both the pre- and post-tests. The survey results showed that the course increased student confidence in both applying EBM concepts and in health literacy and patient communication. The largest gains in confidence in applying EBM concepts were seen in assigning a strength of recommendation (18.7% increase) and formulating an answerable question (17.5%). The largest gains in confidence in health literacy and patient communication were seen in defining health literacy (28.3%) and in finding appropriate consumer resources for further information (18.3%). Both pre- and post-intervention, all students believed physicians in their chosen specialties were equally as good or better communicators as compared to physicians overall.

**Conclusions:** The course was well-received and had a positive impact on student confidence in applying EBM concepts and in health literacy and patient communication. Further analysis of the data is needed to inform future changes to the course delivery and content.

**Keywords:** medical education; evidence-based medicine; patient communication; program evaluation

## Comics in the Medical School Classroom: Health Literacy, Empathy, and Reflective Practice

**Matthew N. Noe**
Collection Outreach Librarian, Harvard Medical School, Franicis A. Countway Library of Medicine, Boston, Massachusetts

**Len Levin, AHIP**
Associate Director, Collections & Knowledge Management, Franicis A. Countway Library of Medicine, Boston, Massachusetts

**Objectives:** Graphic Medicine denotes "the intersection between the medium of comics and the discourse of healthcare." In 2017, we launched a week-long, intensive course on graphic medicine and health literacy for 3rd year medical students. The course covers the history of comics in medicine, health literacy, and the role of storytelling in medicine.

**Methods:** The course focuses on the following learning objectives: ability to describe the comics medium and how it might bridge communication gaps, ability to define challenges in health literacy, and discuss the role that comics can play in building empathy. The course combines lecture, book discussions, and hands-on creative activity to achieve these learning objectives. Lecture portions of the course are kept to a minimum, but are deemed necessary to provide the historical and theoretical overview of graphic medicine and health literacy. Book discussions center on three, carefully chosen medical comics, each designed for a different purpose (memoir, fictional, informational) and explore how each comic might be integrated into practice. Hands-on activities center on exploring the arts in expressing medical experience and culminate in each student creating a comic.

**Keywords:** Graphic Medicine; Health Literacy; Empathy; Reflective Practice

## Improving Health Outcomes with Embedded Diagnostic Consultation Services

**Ansley Stuart, AHIP**
Allied Health Sciences Librarian, Robert B. Greenblatt, M.D. Library, Augusta, Georgia

**Brandy Gunsolus**

**Objectives:** To better inform physicians about how to find guidelines for diagnostic testing, and to provide clinical advice on which diagnostic tests should be ordered.

**Methods:** A clinical laboratory scientist joined the Patient Care Rounding Team (PCRT) to provide consultation regarding diagnostic testing as part of the first doctorate in Clinical Laboratory Science (CLS) program. Documentation and analysis of diagnostics-related questions accumulated during PCRT rounds demonstrated the need for immediate access to advice about diagnostic testing in clinician rounding; the PCRT did not have a defined logic model to order diagnostic tests, place the test orders, test methodology, and determine the clinical value (cost/benefit) of the tests. The CLS resident requested an embedded librarian provide evidence-based research to support clinical decisions to better inform the rounding teams. The librarian demonstrated how to systematically search for evidence to support the choice in diagnostic testing and how to interpret testing results.

**Results:** Within the first 15 days of implementation of CLS consultation on PCRT, there were 146 consultations that improved clinical outcomes and resulted in over $60,000 in cost savings by eliminating unnecessary testing and changed ordered tests to better serve diagnostic needs. The librarian providing evidence-based research consulted on lesser known connections between symptoms and conditions, improving diagnostic clinical decision-making. Both the CLS resident and librarian offered evidence and expertise that decreased inappropriate test utilization while potentially improving clinical outcomes.

**Conclusions:** Incorporating information, laboratory, pharmaceutical, and other scientists into the clinical care team improves stewardship and unnecessary financial spending..

**Keywords:** clinical laboratory sciences, diagnostic testing, diagnostic error, clinical outcomes

## It’s Crunch Time: Adapting and Transforming Hospital Libraries to Support Institutional Budget Goals

**Elizabeth Laera, AHIP**
Medical Librarian, McMahon-Sibley Medical Library, Birmingham, Alabama

**Emily Harris**
Medical Librarian, Kahn-Sibley Medical Library, Birmingham, Alabama

**Objectives:** Hospital librarians have long sought ways to keep their doors open by demonstrating the value of their services. However, in today’s climate of hospital mergers, library closures, and the perceived availability of medical literature, standard methods may not be enough. Hospital librarians need to adapt and find new ways of contributing to their institutions by helping them save money.

**Methods:**
When recently presented with the need to dramatically reduce their budgets, the authors began thinking of ways to do so without impacting services. The librarians were tasked with helping the medical education department of a multi-hospital system meet a budget goal significantly smaller than previous years. Cutting hours, staff, and resources were all considered.

The librarians will also seek the advice and experiences of colleagues facing similar circumstances through a web survey. The survey will be distributed via relevant listservs and will ask participants to share ways, effective or not, that have helped save their institutions money. Respondents will have the option to provide contact information for the authors to conduct follow up interviews.

**Results:** 103 surveys were returned. 86% work in some kind of academic setting; 58% in health systems. Community hospital/health system librarians are more likely to face budget cuts. 19% of respondents have cut more than $100,000 from their budgets in the past 5 years. Almost half felt library services had been negatively impacted by these cuts.

**Conclusions:** The goal of the survey was to find new and inventive ways to help hospital librarians contribute to their organizations in larger ways. Free text survey responses yielded some good ideas and showed that many hospital librarians are using creative thinking to deal with difficult situations. By collecting and sharing experiences from others in similar situations, the authors hope to help other librarians searching for ways to save money and continue assisting clinicians in saving lives.

**Keywords:** hospital libraries, budget, library closures, solo librarians, health systems

## Good Margin of Possibility: Adapting PubMed Search with a Cognitive Apprentice Teaching Strategy to Introduce Evidence-Based Dentistry Skills

**Peter Shipman**
Dental Medicine and Cancer Librarian, Robert B. Greenblatt, MD Library, Augusta, Georgia

**Tasha Wyatt**
Educational Researcher, Educational Innovation Institute, Augusta, Georgia

**Objectives:** To use Cognitive Apprenticeship model teaching strategies to introduce PubMed database searching with evidence-based dentistry (EBD) process skills to predoctoral first-year dental students.

**Methods:** Prior to class, students (n=96) view a recording of the dental librarian creating a PICO (Problem, Intervention, Comparison, Outcome) from a clinical question and searching concepts in three PubMed interfaces [flipped classroom technique]. The Successive Fractions search strategy in the basic interface is preferred. In class, the clinical question, PICO, and search interfaces are quickly reviewed. Each research group (n=13) receives a unique clinical question on a worksheet, then determine the PICO components and PubMed search terms. One person per group has to present how they executed the process. Due to time, four groups were selected to present their PICO and search strategy to the class. Cognitive Apprenticeship allows the librarian to “think out loud” and share expert thinking in the recorded content. In class, other Cognitive Apprenticeship methods allow students to “articulate” how they constructed their PICO and searched the clinical question. The live librarian feedback is “coaching,” and allows the students to “reflect” and compare their proficiency with the librarian’s performance.

**Results:** Students struggle converting the clinical question into PICO and searching for concepts. All four groups applied a single search of the clinical question in the Advanced Search Builder interface. Students used search terms exclusively from the clinical question. Librarian feedback focused on organizing the PICO, verifying term mapping in search details, and expanding search by choosing text word synonyms and word variants.

**Conclusions:** Students are inexperienced in the EBD process, PICO, and searching PubMed, which is expected at this stage of their professional development. Students managed to retrieve useful articles for their clinical questions. Cognitive Apprenticeship methods allow the librarian to learn how students think about

search, and students receive real-time feedback. Future plans include preparing fewer clinical questions, expanding instruction time with students, and preparing better feedback to students based on the student preference for the Advanced Search Builder interface.

**Keywords:** Cognitive apprenticeship
Evidence based dentistry
Database search strategy
Flipped classroom

## Need Stats Support? We've Got Students for That

**Tiffany J. Grant**
Research Informationist, Health Sciences Library, University of Cincinnati, Cincinnati, Ohio

**Objectives:** Data analysis is often a service that researchers outsource due to lack of time or lack of knowledge of tools available. We sought to develop a cost-effective, low maintenance statistical support service that would educate our researchers on statistical software and programming language tools and provide some minor data analyses for research projects.

**Methods:** We submitted a job description to find knowledgeable and motivated university graduate students to tutor their peers, faculty and staff on the programming languages R and Python and the statistical programs SAS and SPSS. The job description indicated that selected students would be tasked with designing and teaching new workshops targeted to the needs of researchers of the medical center. The interview process consisted of a formal interview, and selected candidates were asked to perform a mock tutoring session using R to assess their ability to engage with patrons, their tutoring style, and how well they were able to articulate the tool. Once hired, students underwent an orientation to acclimate them to the library culture, and a new tutoring and data analytics service was advertised to researchers in our medical center.

**Results:** Two graduate students were selected to perform the duties of the newly established program in our health sciences library. Both students had an extensive background in R, Python, SAS, and were able to begin tutoring and take on some minor data analytics projects immediately. The students were given two months to design 2, 2- hour workshops on R that would be taught monthly in the library. The workshops were entitled: Introduction to R and Statistical Analyses with R. The services and workshops were heavily marketed, and were very well received by the research community.

**Conclusions:** The tutoring, and analyses services were offered at no charge to the researchers and at mutual convenient times for the students and the researcher. The workshops were typically rated very well. Thus, graduate students were able to provide a user friend and convenient statistical support service to researchers in our medical center. The student model of support is one that could be considered as a cost effective means to expand the services offered by the library.

**Keywords:** Students, statistical support, cost-effective service

## Diversity Includes Disability: An Informationist's Role

**Anna E. Schnitzer**
Informationist--Diversity/Disability Issues, University of Michigan Taubman Health Sciences Library, Ann Arbor, Michigan

**Objectives:** The President of the University of Michigan launched a plan on the theme of Diversity, Equity and Inclusion. To become an integral and supportive part of this program, a health sciences informationist instrumental in coordinating disability selected the theme: Diversity Includes Disability and instituted a month-long series of events to confirm diversity, equity and inclusion in the world of disability.

**Methods:** Invitations were issued to speakers who were expert in various areas and aspects of disability. A number of panels were organized on the following topics: adaptive technology for individuals with disability, psychology of marginalized persons, graphic comic depictions of disability heroes, and personal narratives of mental health. A film was selected to be screened that focused on diversity and integration of persons with disability ("Including Samuel"). Extensive publicity was used on a consistent, repetitive basis to blanket the campus with the events' themes. This marketing was accomplished through a variety of media: flyers, television screens, and online bulletins and newsletters.

**Results:** A campus-wide audience was targeted through publicizing the October series of events with the theme of Diversity Includes Disability. Some people chose to attend the events in person, but through broad, persistent marketing of the theme, it was concluded that numerous other individuals at the University became aware of the concept of disability being an integral aspect of the diversity program, which was one of the primary desired outcomes of the activity.

**Conclusions:** A campus-wide audience was reached through the informationist's efforts in publicizing a series of events with the theme of Diversity Includes Disability. Some individuals chose to attend the events in person, but through broad, persistent marketing of the theme, it was concluded that numerous other individuals at the University for the first time became aware of the concept of disability being an integral aspect of the diversity program.

**Keywords:** diversity
inclusion
equity
social justice
disability
marketing
publicity
adaptive technology

## The 4Be's: A First-Year Medical Librarian's Strategy for Immersion in a Liaison Area

**Rachel A. Koenig**
Research & Education Librarian, Tompkins-McCaw Library for the Health Sciences, Midlothian, Virginia

**Objectives:** Beginning a new position as a health sciences liaison librarian can be stressful. It takes skill to navigate a new institution and transition from an outsider to a fully immersed colleague in a school, department, or center. This lighting talk describes the integration strategy implemented by a first-year liaison librarian to a School of Pharmacy within a larger comprehensive research university, highlighting the lessons learned in the first year.

**Methods:** To develop the integration strategy, the librarian first met with fellow liaison librarians to the Schools of Allied Health, Dentistry, Medicine, and Nursing. During the meetings, the librarian asked colleagues about the integration strategies they used when they began their liaison assignment as well as strategies they continue to implement or decided to abandon. The librarian tested the strategies in the School of Pharmacy and additionally looked to the literature to determine strategies liaison librarians implemented at other types of academic libraries. She compiled the information into a cohesive approach, which suggested that to become fully

integrated into a liaison area and viewed as a collaborative partner, one must Be Confident, Be Inquisitive, Be Proactive, and Be Consistent. The 4Be’s Approach was monitored throughout the 2017-2018 academic year. Upon assessment of the strategy, the librarian gathered a few lessons that may be used to revise or edit the 4Be's in the future.

**Results:** Each Be is a step along the trajectory toward final immersion into the culture of the liaison area. The librarian has already experienced success during the initial months of implementation; however, upon reflection a few lessons can be learned from her implementation of the strategy.

**Conclusions:** The 4Be’s Approach is applicable at any health sciences library and for any liaison area. Follow-up research may include focus groups with faculty, student, and staff stakeholders in the School of Pharmacy. During these meetings, the librarian will inquire as to how stakeholders began noticing the services of the liaison librarian and how they would best be served moving forward. The results of these meetings can be reported through an updated version of The 4Be’s Approach in the hopes of establishing best practice.

**Keywords:** liaison, pharmacy, organizational culture, collaboration

## The Naomi C. Broering Hispanic Heritage Grant in Action: An Awardee Revisits the Year

**Adela V. Justice, AHIP**
Senior Librarian, The Learning Center, Houston, Texas

**Objectives:** A mid-career librarian was awarded one of the Medical Library Association's professional development grants. The purpose of these grants are to support continuing education and professional activities in a chosen field of study over the course of one year. Highlights from the activities that occurred during the grant's award period will be reviewed and discussed.

**Methods:** The grantee spent the award year studying health informatics and consumer health informatics. These studies occurred in the form of online classes, webinars, and reading two textbooks written by experts in the field. The grant also provided for paid memberships to selected health informatics professional organizations.

**Results:** The librarian became versed in the areas of health informatics and some related areas of study. The librarian plans to continue their education in these areas as well as to encourage widespread interest and participation in the Medical Library Association's professional grants and scholarships.

**Keywords:** MLA Awards, health informatics, consumer health

## Transforming Health Sciences Education with Virtual Reality

**Tallie Casucci**
Assistant Librarian, J. Willard Marriott Library, Salt Lake City, Utah

**Donna Baluchi**
Supervisor - Public Services, Spencer S. Eccles Health Sciences Library, Salt Lake City, Utah

**Brandon Patterson**
Technology Engagement Librarian, Spencer S. Eccles Health Sciences Library, Salt Lake City, Utah

**Nancy Lombardo**
Associate Director for Digital Collections, Spencer S. Eccles Health Sciences Library, Salt Lake City, Utah

**Erin Wimmer**
Instructional Designer, n/a, Cottonwood Heights, Utah

**Bryan Hull, Program Manager, Digital Collections, at the Spencer S. Eccles Health Sciences Library**

**Objectives:** To establish a centralized virtual reality (VR) space within the library where the health sciences community can experiment with VR, discover its capabilities, use VR patient and student education modules, and learn to develop VR products.

**Methods:** As campus demand and interest in VR education expand, library personnel interviewed VR champions and potential users of a future library space dedicated to VR play and development. Funds for VR equipment were secured through the Technology Improvement Award from the National Network of Libraries of Medicine MidContinental Region (NNLM MCR). The new VR Studio was added to the library educational technology studio space. The VR Studio was promoted through open houses, educational programs, hands-on workshops, events, and on-demand learning modules.

**Results:** The VR equipment was purchased and is currently set-up in a computer lab. The unveiling of this new equipment occurred during our Open House on Friday, October 13, 2017. The Open House highlighted our new innovative technologies and exhibits. During the Open House an estimated 80 people stopped by and about 60 people interacted with the VR station. In spring 2018, a student intern was hired to help develop workshops, on-demand learning modules, and library created and curated content. The Library is currently working with the Department of Ophthalmology to develop a tour of the eye.

**Conclusions:** VR will revolutionize teaching, especially for hard-to-teach topics, interprofessional team-work, and action-based tasks. By collaborating with health sciences departments, the Library can provide new teaching and learning tools that help students visualize and become immersed.

**Keywords:** virtual reality, VR, education, technology, educational technology

## Data Papers: How Librarians Can Promote Data Sharing

**Sarah Meyer**
Assistant University Librarian, Health Science Center Libraries, Gainesville, Florida

**Objectives :** For data-driven researchers, collection and identification of high-quality data sets is a paramount process of the research cycle. To establish creditability, collected data must be discoverable and utilized by researchers. The facets of data collection and sharing must be addressed for the impact of data-driven research to be fully realized. One potential solution is the publication of data papers

**Methods :** A data paper describes a specific data set, is a searchable metadata document, published in a peer-reviewed scholarly journal. Data papers , unlike traditional scholarly articles, do not require any interpretation, or analysis, rather value is established by enhancing data sets with robust metadata to increase; discovery, usability and credibility. As the roles of librarians in data-driven research expands one method to increase data sharing of data sets is to acquire and share knowledge with researchers about publishing data papers as a viable translation method.

**Results :** The publication of data papers facilitates sharing and provides recognition to authors. Applying methods to increase the intersection between data collection and sharing enhances data-driven research. Librarians can be instrumental in promoting data sharing by disseminating knowledge about data paper publications to researchers. Additionally librarians can assist in the development of metadata to enhance the discoverability of data sets and preservation of data sets in repositories.

**Conclusion :** The value of data-driven research relies on the creation and identification of high-quality, accessible datasets. Data papers provide a tool to identify data sets, avoid duplication of efforts, enhance reuse, and reduces barriers of traditionally published scholarly articles. There are many opportunities for librarians to have participatory roles in the publication of data papers.

**Keywords :** data papers, data sharing, metadata, data reuse,

## Harvard's Legacy for Data Services

**Julie Goldman**
Research Data Services Librarian, Countway Library of Medicine, Boston, Massachusetts

**Objectives:** The Harvard Library has a decentralized organizational structure serving a wide breadth of disciplines. This model allows librarians to build close relationships with research communities, but it presents challenges to systematically connect researchers to expertise necessary to support their data needs. In order to formalize a more integrated strategy, Harvard Library is developing a strategic plan for providing data services.

**Methods:** The Harvard Library Research Data Management Program connects community members to services and resources spanning the research data lifecycle, to help ensure multi-disciplinary research data is findable, accessible, interoperable, and reusable. HL-supported data librarians embedded at the school or department level provide direct-to-patron services. The program also provides program-to-unit services which include a directory of services and community events. In addition, the Harvard Medical School Data Management Working Group includes a variety of representatives. The group endeavors to help create solutions, provide guidance, and develop standards and best practices to meet unmet needs and anticipate future needs of biomedical researchers. Coordination must happen in conjunction with the HMS DMWG, and be transparent to the entire Harvard Library system. It is the goal of a strategic plan to outline Countway Library data services in a systematic, rather than ad hoc, fashion.

**Results:** Four strategic priorities identified:

Service/Collections: support DMPTool; advocate appropriate repositories; support ORCID adoption; provide consultations; re-think library space to support research.

Outreach/Resources: website with resources and guidance; RDM lectures; provide software and tools necessary for biomedical research; collaborate with local and national partners to foster best practices; identify strategies for promotion of services.

Education/Training: collaborate with others to provide training; offer specialized bioinformatics training; equip librarians with the skills necessary to provide data services.

Assessment/Impact: tie in data services assessment to the library-wide program; explore user feedback methods; gather analytics and implement user feedback tools.

**Conclusions:** The ultimate goal is to improve communication between the disparate groups on campus, as well as promote the Library’s services as the same. Working with a variety of entities will help guide Harvard Library to foster strong collaborations with diverse partners. With guidance from this strategic plan, the library will look to emerging trends and determine tools and knowledge necessary to implement new services.

**Keywords:** research data services, research data management, Harvard Library, strategic plan

## Partnering to Improve Institutional Data Policy Transparency: Leading Researchers in the Right Direction

**Nicole Contaxis**
Data Catalog Coordinator, NYU Health Sciences Library, New York, New York

**Alisa Surkis**
Assistant Director for Research Data and Metrics, NYU Health Sciences Library, New York, New York

**Kevin B. Read**
Knowledge Management Librarian, NYU Health Sciences Library, New York, New York

**Objectives :** Librarians conducting data management consultations within an academic medical center identified researcher confusion concerning institutional data sharing policies related to government, funder, and publisher policies. To address these challenges, librarians partnered with General Counsel, the Office of Science and Research, Basic Science Operations, and Research Information Technology (IT) to develop resources for disseminating information about the institution's data sharing policies.

**Methods :** Librarians collected anonymized data during data management consultations to inform institutional stakeholders. The library met with the General Counsel, the Office of Science and Research, Basic Science Operations, and Research IT to discuss researcher needs in regards to the confusion around institutional data policies. This partnership developed strategies to disseminate information about institutional data policies through the following library-led initiatives: (1) a library class series on data; (2) data management consultations; (3) a librarian-led graduate level course; (4) REDCap training; and (5) an institutional data catalog. Pursuing those initiatives, the library subsequently addressed researcher confusion about data sharing policies by creating data policy presentation slides, developing an institutional data policy FAQ, and facilitating the use of manuscript proposal forms for research collaboration. The library continues to partner with key stakeholders to develop methods for improving awareness of data sharing policies.

**Results :**Continued collaboration with stakeholders has resulted in the creation of a FAQ to clarify institutional policies for data sharing. Librarians supplied questions from previous research consultations while the General Counsel provided guidance on answers. Research consultations have resulted in the creation of manuscript proposal forms to assist them in specifying conditions for data sharing and collaboration. The manuscript proposal form is now offered to all researchers who participate in the institutional data catalog, and it has been used by several researchers. To address institutional data sharing issues on a broader scale, library will collaborate with General Counsel to hold a workshop on institutional data sharing and transfer agreements. The library will continue meeting with key stakeholders as data sharing issues arise from future researcher consultations.

**Conclusion :**Addressing researcher confusion about institutional data sharing policies requires consistent communication with relevant stakeholders, including General Counsel, Basic Science Operations, the Office of Science and Research, and Research Information Technology (IT). Through this collaborative effort, librarians can play a role in improving resources for researchers about institutional data sharing policies.

**Keywords :** Data Sharing, Policy, Data Management, Researcher Needs

Monday, May 21, 2018, 1:00 PM – 2:25 PM

Room: Embassy F (International Tower, Exhibit Level)

---

# Session: Lightning Talks 4

**Moderator: Timothy R. Roberts**

## Building a Framework to Guide Residents through Scholarly Activities

**Lindsay Blake, AHIP**
Clinical Services Librarian, University of Arkansas for the Medical Sciences, Little Rock, Arkansas

**Jennie T. Kirby**
Community Based Residency Program Manager, Graduate Medical Education, Arkansas

**Jennifer White**
Program Manager, Graduate Medical Education, Arkansas

**Objectives :** The objective of this project is to build an online resource that will help guide residents at a large health sciences campus through their Accreditation Council for Graduate Medical Education(ACGME) scholarly activity requirement by providing guidelines, advice, and resources on publishing, presenting, and research.

**Methods :** The Clinical Services Librarian first met with the Graduate Medical Education(GME) office to determine the needs of the residencies and clarify current ACGME requirements. Meetings were then set with educational and research representatives from the largest residencies to discuss how the scholarly activity requirement was currently being met and collect resources. After meeting with a number of residencies the librarian began building an online resource in the LibGuides software. The guide, Research and Scholarly Activity Guide, combines practical advice for residents and new faculty, from setting up a poster or writing an abstract to where to print your poster or submit your abstract. Research resources help guide users through the institutional review board, finding a mentor, and where to find statistical help.

**Results :** The Research and Scholarly Activity Guide is still being built, but residents are piloting early versions to create posters and prepare for oral presentations. The guide was expanded after meeting with residency representatives since many work with both residents and novice faculty. The most difficult section of this project deals with completing the research portion of the guide. Guiding residents through all the complexities of research while at the same time making the path easier to understand and follow is challenging. Feedback and resources from residency representatives and the GME office have assisted in building a complete site.

**Conclusion :** The full Research and Scholarly Activity Guide will be available to the campus starting with the new class of residents in July 2018. The GME office is providing residency statistics on completion of projects and resident feedback. Evaluation will also come from guide use statistics. It is expected that the guide will bring a number of disparate resources together easing residents road to completion of this requirement.

**Keywords :** graduate medical education, clinical librarian, scholarly activities

## Development of a Mobile-Friendly Website to Strengthen Clinical Decision Making

**Donna O'Malley**
Interim Director, Dana Medical Library, Burlington, Vermont

**Alice Stokes**

**Gary S. Atwood**
Research and Instruction Librarian, University of Vermont, Dana Medical Library, Burlington, Vermont

**Lynda Howell**

**Objectives :** Librarians' primary objective was to develop a mobile-friendly website for a month-long problem-based learning (PBL) course for pre-clinical medical students. The website sought to improve the range and quality of sources used by students in the PBL course. A secondary objective was to offer a website to encourage the use of evidence-based medicine tools in clinical care.

**Methods :** Librarians used a design thinking approach to rapidly create and roll out the website. After meeting with the course director for the College of Medicine PBL course to discuss desired content and functions, the library IT Specialist developed a prototype. The IT specialist used off-the-shelf technology to create a device- and platform-agnostic mobile-friendly website (http://go.uvm.edu/ebmtree). Development of the website also required the creation and integration of two additional web pages within the library's central website. Librarians conducted usability testing with 3rd and 4th year medical students and used their feedback to create a workable version in December 2016. Librarians developed longitudinal instruction sessions to align with the use of the website in the problem-based course in January 2017. The results of that implementation then fed into the next iteration of the mobile website.

**Results :** Written student evaluations suggest that they found the website useful when searching for appropriate library resources. Feedback from clinicians was also positive. Both groups contributed suggestions for improvement, which were implemented for the 2018 instance of the course. Google analytics data demonstrates ongoing use of the website throughout the clerkship year. Though the librarians and the course instructor emphasized the mobile-friendly advantages of the website, most usage was through laptop or desktop computers.

**Conclusion :** Involving the target audience in website development allowed librarians to create a focused site with a clear purpose. An additional benefit of the project is that it fostered deeper conversations about evidence-based medicine in the medical curriculum, and library support of evidence-based medicine instruction.

**Keywords :** mobile, website, discovery, evidence-based medicine, problem-based learning, undergraduate medical education

## From Transactional Service Model to Programmatic Approach: The Design, Implementation, and Measurement of Engaging User Experiences in a Knowledge Center

**Melissa L. Mendelson**
Director, Programs, The Knowledge Center at The Augustus C. Long Health Sciences Library, Columbia University Irving Medical Center, New York, New York

**Objectives :** To design, implement and measure sustainable and engaging user experiences through programs that captivate, enable, challenge and connect constituents.

**Methods :** The need for capturing and sustaining users' attention was identified by observing and analyzing service transactions with library users over an extended time period. Transformation to a programmatic model was envisioned as mission-critical to engage library constituents with information on a scalable and sustainable level. Next, a novel role of a Programs Director (PD) in a health sciences library was conceptualized to lead the design and implementation of a programmatic approach in alignment with the library's mission. With the PD role filled and Programs team successively recruited, programs and services utilizing the programmatic approach have and continue to be instituted and subsequently evaluated via pre/post testing, semi-structured interviews, feedback surveys, etc. Expected results/outcomes: Utilization of the programmatic approach generates a multitude of successfully designed and implemented programs that are measurable, sustainable and engaging experiences for the community of library users.

**Results :** Results will be made available during the presentation at MLA '18.

**Conclusion :** Conclusion(s) will be made available during the presentation at MLA '18.

**Keywords :** SUSTAINABILITY; PROGRAMMATIC APPROACH; PROGRAM DESIGN/IMPLEMENTATION; PROGRAM MEASUREMENT; USER ENGAGEMENT

## Humanities Reading Selective for Medical Students: Reflecting on the Human Side of Medicine

**Lisa Marks, AHIP**
Director, Library Services, Staff Library, Scottsdale, Arizona

**Objectives :** This Lightning Talk will discuss an option our medical school students have during dedicated 1-2 week blocks of time, for a selective experience of their choosing that is self-directed and emphasizes personal responsibility for the learning experience. This particular selective involves reading titles related to the field of Humanities in Medicine.

**Methods :** Selective blocks during years 1/2 give students' time for "career exploration, enhancing clinical skills, achieving higher learning objectives through reflection", etc. The Humanities in Medicine reading selective gives students a chance to read titles on the human side of medicine that give insight into the human condition, suffering, resilience, or historical/personal perspectives on medical practice, etc. Students are asked to read 2-3 titles then write a personal reflective essay - not a book report - on what they've read. They are also asked to think about how their mind was changed by reading these books, or how the books opened their eyes to situations they've not thought about before, how they were made to think/feel differently about medicine, or career choice. The student and librarian meet to discuss the titles read as well as the essay that was written.

**Results :** The librarian has had 22 students (of a class of 50) complete the reading selective during their first year. Feedback from the students has been positive and most would repeat the selective if allowed to do so. Most students read at least 2 books including titles like: "When Breath Becomes Air", "The House of God", "Cutting for Stone" and "How Doctors Feel". The essays written have been both personal and reflective on what

was learned by reading the books chosen.

**Conclusion :** The reading selective has given the librarian one-on-one time with each student allowing her to get to know the students on a personal level. It has also allowed the students to be reflective and share some of their own personal stories in a safe space. Overall, the selective is a positive experience for both the students and the librarian.
**If Accepted, Prefer Monday, May 21, 1:00-2:25pm presentation time. I will be leaving ATL Tuesday noon-time for daughter's graduation later that week. Thank you for considering this**

**Keywords :** Humanities in Medicine
Medical Students

## Increasing the Visibility of a Health Sciences Library Book Collection: Influencing Use of Book Collection by Using “Choice Architecture”

**Ricardo Andrade, Jr.**
Assistant Director for Information Resources, Columbia University Augustus C Long Health Sciences Library, New York, New York

**Michael Koehn**
Director for Library Operations, Columbia University Augustus C Long Health Sciences Library, New York, New York

**Eric Dillalogue**
Access Services Manager, Columbia University Augustus C Long Health Sciences Library, New York, New York

**Objectives :** Using library space to increase visibility of the HSL collection by redesigning the physical collection.

**Methods :** Declining use of the print books and new additions of electronic book packages prompted the HSL library to reconsider how to house and organize its collections. The books were consolidated from three separate locations into one on the main level of the library to ensure maximum visibility with the readers. Only the highest use titles were kept onsite and a large percentage of the collection was moved to an offsite storage facility. The library also decided to integrate the non-circulating reference materials and circulating general collections and inter-shelved those. Signage was created to promote electronic versions of high use titles and advertise new ebook collections. A formal project management technique was used by the library staff to re-organize the print collection. A variety of data was used to support decision-making process, as well as to establish baseline for future decisions.

**Results :** The lightning talk will cover the changes made to the physical library space, signage and next steps.

**Conclusion :** Conclusions will be covered in the lightning talk.

**Keywords :** Collection Development, Information Resources, Marketing, Users

## International Collaboration with the Fulbright Specialist Program

**Kathy Kwan**
Health Information Specialist, Independent, POTOMAC, Maryland

**Objectives :** Fulbright Specialist Program is a unique opportunity for US professionals to engage in short-term projects at organizations overseas. This talk highlights the steps taken by an MLA member to develop such a project. The goal of the talk is to share with MLA members this viable path for international collaboration.

**Methods :** The author was accepted into the Fulbright Specialist Roster and became eligible to work with an overseas organization on a Fulbright Specialist project. During a networking opportunity at MLA 2016 annual meeting, the author initiated the discussion with the representatives from the National Taiwan University Medical Library (NTUML) and Taiwan Medical Library Association (TMLA) to collaborate on a project. Subsequent discussions led to the focus of promoting Open Science in the biomedical research community in Taiwan and explore the librarian's roles in it. Working with the NTUML staff, the project proposal "Change to open: Open Science and Medical Libraries" was developed and approved by the Fulbright Commission in Taiwan and the US Department of State. The final approval of the match between the project and the author was granted by the Fulbright Foreign Scholarship Board.

**Results :** The project was implemented between October to December, 2017. A range of activities were conducted, including interviews and talks, to gauge the awareness and acceptance of Open Science in biomedical research in Taiwan. The target audiences included researchers at NTU College of Medicine, Academia Sinica and members of TMLA. An online survey was also conducted to reach a wider audience. The result of the project is being analyzed and will be shared in the future.

**Conclusion :** The short-term and flexible nature of the Fulbright Specialist Program is a great way for librarians, especially those who may not be engaged in international activities in their work duties, to engage in international collaboration and capacity building of an organization overseas.

**Keywords :** Fulbright Specialist Program
International Collaboration
Capacity Building
Taiwan

## Leading Nurses Up the Clinical Ladder

**Emily Brennan**
Research and Education Informationist, Medical University of South Carolina (MUSC) Library, Charleston, South Carolina

**Amanda Davis**
Senior Evidence-Based Practice Analyst, Value Institute, Charleston, South Carolina

**Rebecca Harper**
Senior Decision Support Implementation Specialist, Value Institute, Charleston, South Carolina

**Objectives :** To describe the Value Institute's involvement in the hospital's RN III projects.

**Methods :** In order for nurses to obtain a level three Registered Nurse (RN III) certification, they must complete an evidence-based practice (EBP) project that seeks to improve patient outcomes on their unit and within the organization. A Shared Governance committee at Medical University of South Carolina (MUSC) Health, a Magnet hospital, asked the Value Institute to teach a course geared toward this specific cohort of nurses. The Value Institute EBP team is comprised of a librarian, Senior Evidence-Based Practice Analyst, and Senior

Decision Support Implementation Specialist. From February through May 2018, nine nurses completed the hybrid course, which consisted of two in-person sessions, seven online modules, and three required consultations. Course content included developing the clinical question, searching the literature, appraising the evidence, and planning for implementation.

**Results :** The Value Institute hopes that nurses who complete the EBP course will develop higher quality projects, and have a higher success rate of receiving their RN III certification. Participants are eligible to receive 15 CE credits for completing the course. Nurses would also learn evidence-based practice skills that they would be able to use throughout their career to impact patient care delivery and outcomes.

**Conclusion :** Forthcoming.

**Keywords :** evidence-based practice, RN III, hospital nurses, process improvement, clinical ladder

## Streamlining the Accessing Process of Library Resources to Participants in a Distributive Model of Medical Education

**Marcelle Savoy, AHIP**
Medical Librarian, Lincoln Memorial University|DeBusk College of Osteopathic Medicine, Harrogate, Tennessee

**Objectives :** The objective of this talk is to present a procedure developed by librarians and coordinators, which markedly facilitated the accessing of library resources by faculty and students of a medical school which utilizes the community-based distributive model of medical education.

**Methods :** In centralized medical school facilities, library access to faculty and students is generally a straight forward process; however, their counterparts in different community-based centers, which make up the foundation of the distributive model of medical education, typically must navigate through a maze of administrative layers to access library resources.
A total of six key personnel were identified in the library access process. These included: the medical librarian, the appointed adjunct faculty coordinator, the IT technician, the electronic resources librarian, a vendor representative, and the systems librarian. After the roles of these individuals were described, a workable procedure was developed. Once a request was submitted, the medical librarian determined which individual was best suited to assist with the query based on the flow process system.

**Results :** The flow process developed is illustrated and explained using a sample query from an appointed adjunct faculty member located in a community-based medical facility.

**Conclusion :** By identifying and forging interconnected relationships with all members dedicated to supporting the needs of faculty and students, problems with library access, which once took days to answer, can now be resolved in hours and in many instances in less than one hour.

**Keywords :** Distributive Model of Medical Education; Library Access

## Students Editing Wikipedia

**Emily Brennan**
Research and Education Informationist, Medical University of South Carolina (MUSC) Library, Charleston, South Carolina

**Angela Dempsey**
Association Dean / Associate Professor, Medical University of South Carolina (MUSC) College of Medicine

**Objectives :** To describe medical students' experience with editing health topics on Wikipedia.

**Methods :** Wikipedia is the world's most consulted source of medical information, used by patients and health professionals alike. Based on encouragement from an aspirational institution, the Medical University of South Carolina (MUSC) initiated an elective for forth year medical students, "WikiEd: Medical Writing for the Public". This 4-week elective was co-taught by a librarian and College of Medicine faculty member. During November 2017 through December 2018, 12 medical students completed the course, which consisted of an in-person orientation session, online coursework, weekly check-ins, and a final presentation. Each student chose a medical topic of interest to edit throughout the course. Students received instruction on editing Wikipedia, writing for the public, health literacy, and identifying sources and citing sources.

**Results :** In total, 12 students edited 12 articles, made 868 edits, and added 35,000 words. The students' articles were viewed 730,000 times by the public. Students improved scholarly skills such as differentiating between primary and secondary sources, performing literature reviews, and properly citing sources. Students also gained valuable clinical skills including medical writing for the public and peer review, and reported increased confidence in their ability to write a manuscript for publication. The WikiEd course offered students the flexibility to travel during interview season, while simultaneously gaining academic and clinical skills.

**Conclusion :** Forthcoming.

**Keywords :** Wikipedia, wikied, medical students, medical education, research elective

## Transforming Health Sciences/Medical Librarian Courses for Library and Information Science Students

**Rebecca O. Davis**
Senior Lecturer, Simmons College / School of Library and Information Science, Boston, Massachusetts

**Objectives:** To determine how health sciences and medical library courses can be transformed in Library and Information Science programs to better prepare LIS students to enter the health sciences and medical librarian fields. Determining what competencies LIS students need to get jobs as health sciences and medical librarians.

**Methods:** Examine the literature on health sciences and medical libraries courses that are taught in Library and Information Science programs in the United States. Examine course syllabi and job advertisements to learn about the content covered in health sciences and medical library sources and the skills needed to become a librarian in this field.

**Results:** No results as this is an examination of literature, course syllabi and job advertisements.

**Conclusion:** No conclusions yet.

**Keywords:** Health Sciences or Medical Library Courses
Competencies
LIS Programs

## Transforming the G-Index: From Confusion to Clarity

**Liz Kellermeyer**
Biomedical Research Librarian, Library & Knowledge Services, Denver, Colorado

**Objectives :** The g-index is a bibliometric based on the distribution of citations received by a researcher's publications, giving highly cited articles more influence on the overall score. We created a tool to automate the calculation of the g-index, and then examined the usefulness of integrating the index into citation reports librarians prepare for faculty members.

**Methods :** Bibliometric services, including individualized citation reports, are one of the library services offered to faculty at our outpatient research hospital. Our institution's Faculty Appointment, Promotion, and Periodic Evaluation Committee contacted the library for guidance on standardizing citation reports submitted by faculty under review for promotion. The standard citation report as generated through Web of Science contains a publication list, citation counts and averages, and an h-index. To add a more nuanced view of the publication data, the library suggested including the g-index. Library staff prepared instruction for the Committee so they could better understand and interpret the citation reports. Library staff now provide reports with both the h- and g- indices. The g-index can be cumbersome to tally by hand, so our librarians developed a simple calculator in Excel that automatically determines a g-index for a set of citation totals.

**Results :** The Faculty Appointment, Promotion, and Periodic Evaluation Committee responded favorably to the addition of the g-index and made it a required element of faculty review and promotion packets. While close peer review of all publication data is still part of the committee's process, the g-index serves as a beneficial metric to draw attention to highly-cited publications. The g-index calculator was uploaded to a LibGuide and is publically available for use.

**Conclusion :** Adding a g-index to a citation report creates more nuance, complementing the h-index to provide a more accurate evaluation of an author's impact. The new metric has been embraced by faculty members and administration, and has sparked an interest in continued bibliometric education

**Keywords :** Bibliometrics, Publishing, Academic Faculty Services, Citation Reports

## Using Social Media to Promote HIV/AIDS Awareness on Campus

**Hannah F. Norton, AHIP**
Reference & Liaison Librarian, Health Science Center Libraries, Gainesville, Florida

**Margaret Ansell, AHIP**
Nursing & Consumer Health Liaison Librarian, Health Science Center Libraries, Gainesville, Florida

**Ariel Pomputius**
Health Sciences Liaison Librarian, Health Science Center Libraries, Gainesville, Florida

**Mary E. Edwards**
Reference & Liaison Librarian, Health Science Center Libraries, Gainesville, Florida

**Matthew Daley**

**Susan Harnett**
Medical Information Services Librarian, Health Science Center Libraries-Borland Library, Jacksonville, Florida

**Objectives :** Librarians at our institution engaged in a year-long project to raise awareness about HIV/AIDS risks, prevention, and treatment among university students and improve their information-seeking behaviors related to this disease. One of the primary activities of the project was a social marketing campaign, using content from an accompanying graphic novel contest and other locally-relevant information.

**Methods :** To enhance students' access to high-quality HIV/AIDS information, the team developed a month-long social marketing campaign, with daily Facebook posts leading to World AIDS Day. The preceding graphic novel contest provided illustrations for core messages within the campaign. To broaden the campaign audience, twelve Facebook posts were boosted, displaying them to individuals based on age and location. Posts linked to relevant local groups such as the student health promotion service, student health center, and researchers focusing on HIV/AIDS.

**Results :** The social media campaign included 27 messages throughout the month of November. Overall, messages in this campaign were viewed 52,149 times, with 5,327 views resulting from "organic reach" (the library's existing followers on Facebook and their shares) and 46,822 resulting from paid reach (Facebook users who were shown the promoted posts due to their age and geographic proximity to the university campus). Beyond simple views of these messages, there was a high level of engagement, with 1,120 clicks, 430 likes, 29 shares, and 19 comments on messages in this campaign.

**Conclusion :** Using a social media campaign proved successful in engaging students on our campus on the topic of HIV/AIDS awareness. This experience taught the team that Facebook's advertising mechanism for "boosting posts" is useful in widening the audience for library-generated information. Developing social media campaigns and, to a more limited extent, paying to boost posts are strategies the library plans to use in the future to get the word out about important health topics, events, and services.

**Keywords :** Social marketing, information outreach, graphic medicine, HIV, AIDS

Monday, May 21, 2018, 1:00 PM – 2:25 PM

Room: International Ballroom North (International Tower, Ballroom Level)

---

# Special Content Session: Collecting Data in the Field: Overview and Stories from Investigations

**Moderator: Kristine Alpi, AHIP**

## Collecting Data in the Field: Overview and Stories from Investigations


**Kristine Alpi, AHIP**
Director, William Rand Kenan, Jr. Library of Veterinary Medicine, Raleigh, North Carolina

**Barbara Knust**
Epidemiologist, Viral Special Pathogens Branch, Centers for Disease Control and Prevention, Atlanta, Georgia

**Aaron Kofman**
Epidemic Intelligence Service Officer, Centers for Disease Control and Prevention, Atlanta, Georgia


**Program Description:** Public health workers have long engaged with communities through gathering focused epidemiological data on the incidence and distribution of diseases and associated control efforts. However, the degree to which communities actively engage as partners in the process of public health research varies. Approaches like participatory epidemiology aim to enhance community engagement in “identifying the causes of health problems and in finding strategies to address them” (PMC5301332). At a broad level, these efforts aim for “research...not done ‘on’ people as passive subjects providing ‘data,’ but ‘with’ them to provide relevant information for improving their lives” (http://www.icphr.org/uploads/2/0/3/9/20399575/what_is_the_icphr_-_short_description_-_version_2014_10_20.pdf).

After some background on participatory epidemiology, facilitators will guide participants through simulation using practices of participatory epidemiology, including interactive data collection and sharing back of that data ‘in real time’. Participants will provide and collect data on a topic to be determined by the facilitators; however, the topic of the session is less important than the process of experiencing and learning about community-based data collection through the simulation.

This program will demonstrate approaches to handling community data collection and engaging participation from stakeholders in communities. This session will also highlight best practices as well as challenges related to data collection and data sharing in this context. Finally, emphasis will be placed on methods of disseminating data back to community members and exploring how communities can benefit from this type of participation in research.


**Keywords:** data collection, mobile devices, simulation, participatory epidemiology, participatory health research,

citizen science

Monday, May 21, 2018, 1:00 PM – 2:25 PM
Room: International Ballroom South (International Tower, Ballroom Level)

---

# Special Content Session: Show Me a Story: Data Storytelling Using Familiar Tools

**Moderator: Merle Rosenzweig**

## Show Me a Story: Data Storytelling Using Familiar Tools

**Sally A. Gore**
Research Evaluation Analyst, University of Massachusetts Medical School, Worcester, Massachusetts

**Program Description:** This session will demonstrate best practices for designing charts and other visuals that enhance one's ability to clearly and effectively present the story behind the data. Tools and technologies that most librarians already have, including Excel and PowerPoint, will be covered, as well as menu-driven online tools like Tableau. Numerous examples and creative in-session activities will give attendees a jump-start for thinking about how they can add data storytelling to their skillset, making them better communicators to all of their different stakeholders.

**Keywords:** Data visualization,
Data storytelling,
Infographics,
Research Impact,
Advocacy,
Evaluation

Tuesday, May 22, 2018, 3:00 PM – 4:25 PM

Room: International Ballroom North (International Tower, Ballroom Level)

# Session: Consumer Health 1 (CH-1)

**Moderator: Margaret Peloquin, AHIP**

## Assessing Wellness Needs of Library Users at a Health Sciences Center

**Ariel Pomputius**
Health Sciences Liaison Librarian, Health Science Center Libraries, Gainesville, Florida

**Margaret Ansell, AHIP**
Nursing & Consumer Health Liaison Librarian, Health Science Center Libraries, Gainesville, Florida

**Jane Morgan-Daniel, AHIP**
Community Engagement and Health Literacy Librarian, Health Science Center Libraries, University of Florida, Gainesville, Florida

**Nina Stoyan-Rosenzweig**
Senior Associate in Libraries, Health Science Center Libraries, Gainesville, Florida

**Robert Lockwood**
Senior Library Technical Assistant, Health Science Center Libraries, Gainesville, Florida

**Terry K. Selfe, AHIP**
Translational Research and Impact Librarian, University of Florida, Gainesville, Florida

**Michele R. Tennant, AHIP**
Associate Director, Health Science Center Libraries, Gainesville, Florida

**Objectives:** In the interest of developing wellness programming at the library of a large academic health center, a Wellness Team was created. The first order of that Wellness Team was to create and administer a survey assessing the wellness needs of library users, and to use those results to develop programming that would support community health and well-being.

**Methods:** The survey was originally administered to frequent library users through a sealed suggestion box at the library entrance. However, a low response rate and increasing interest in reaching a broader audience made it necessary for the team to expand distribution to all six colleges of the health science center community via emails sent by the liaison librarians. The original paper survey was anonymous, and included four questions on perceptions of wellness, current participation in wellness programming, and suggestions for program content and scheduling. The version distributed via email added some basic demographic questions. Institutional

Review Board exemption was sought and received for both versions of the survey.

**Results:** A total of 125 surveys were received. Preliminary analysis of the survey results shows that library users were particularly interested in participating in: meditative exercises such as yoga (55); therapy animals (53); meditation (23), creative expression activities such as coloring or origami (23), exercise equipment (9) and free coffee break socials (8). An in-depth analysis of the survey responses revealed several themes related to the library's role in wellness: popular wellness services, contradictory expectations, barriers to participation, and new areas of exploration. Respondents were interested in attending wellness programs at the library at least once a week, preferably in the afternoon or evenings.

**Conclusions:** The Wellness Team used the survey data and conversations with student representatives to assess the health science center community's wellness activity preferences, and to develop a plan for instituting wellness activities and long-term programming. That the library has a role in promoting wellness in the community was evident from the assessment results, but what exactly that role is and how the library can best carry it out is still evolving. While wellness programming is a worthwhile endeavor for any library, efforts must be tailored to the specific needs and preferences of the library's unique audience.

**Keywords:** wellness, mindfulness, programs, assessment, survey, research

## Community Knowledge Assessment of HPV Vaccination in Males

**Julia M. Esparza, AHIP**
Head, User Education and Outreach Services, Medical Library, Shreveport, Louisiana

**Grace N. Dodd**
Student Researcher, LSUHSC Library, Shreveport, Louisiana

**Derrick Murcia**
Student Worker, Neurosurgery, Frierson, Louisiana

**Jessica D. Sims**
Student, Multicultural Affairs, Shreveport, Louisiana

**Gunjan Kahlon**
Chair of Hospital Medicine, Department of Internal Medicine, Philadelphia, Pennsylvania

**Meher Sindhoora**
Assistant Professor, Department of Medicine, Shreveport, Louisiana

**Eric Thomas**
Section Chief - Internal Medicine, Department of Medicine, Shreveport, Louisiana

**Afaf Abdulbaki**
Clinical pharmacist, Pharmacy, Shreveport, Louisiana

**Lois Anderson**
Director of Pharmacy, CHRISTUS Shreveport - Bossier Health System, Shreveport, Louisiana

**Objectives:** To ascertain community member perspectives regarding transmission of Human Papillomavirus infection, associated diseases and to identify barriers which prevent these groups from ensuring that males 9-26 receive the three-shot vaccine series to prevent HPV infection. Using this information develop and implement

health professional and patient education.

**Methods:** After Institutional Board Approval at an Academic Medical Center, a community survey using a convenience sample of visitors and patients to outpatient clinics was done. To establish a margin of error ±6%, 270 paper surveys were needed. The survey was 15 questions broken into a demographics section, HPV vaccination status of their children or self (if a young adult), their knowledge of transmission of HPV and if they would vaccinate their children or self based on the information provided in the survey. Participation was sought from visitors in the outpatient Internal Medicine and Pediatric clinics. Over 800 individuals were approached to complete the survey. Surveys were collected during June through July, 2016. The Medical Library, Internal Medicine, and Pediatric faculty with the Hospital and Outpatient Pharmacy spearheaded the project. The Medical Library faculty recruited three research apprentices to collect data.

**Results:** The survey was completed by 385 (95% confidence interval with a ±5% margin of error) participants. Over 80% of the participants were female (80%) with most being African American (65%) or Caucasian (29%). Results show that 50% of the survey participants never had a physician discuss HPV vaccination, 39% didn't know the diseases associated with persistent HPV infection and only 39% knew that sexual contact is the mode of transmission. "Other" comments demonstrate the information needs of the participant population with statements such as "heard the vaccine doesn't work" and "don't trust vaccines" still a part of the community knowledge.

**Conclusions:** To date this is the largest study United States face-to face (not telephone) study of community knowledge on male HPV vaccination, knowledge of the results of persistent infection, methods of transmission, and change in vaccination acceptance. The results provided crucial insights. Healthcare providers were not always having conversations about vaccination of males against HPV and that there was a gap in community knowledge on the cancers associated with persistent infection with HPV, and how HPV was transmitted. With this data a grant was obtained to create a comprehensive educational plan for healthcare providers and the community.

**Keywords:** survey research community knowledge assessment "human papillomavirus" literacy consumer children

## Healthy People 2020 Health Disparities Data Widget: Finding and Working with Data on Minority Health

**Faye D. Williams**
Knowledge Center Manager, Office of Minority Health Resource Center, Knowledge Center, Washington, District of Columbia

**Minh Wendt**
Data Policy Lead, Division of Policy and Data, Rockville, Maryland

**Yen Lin**
Public Health Advisor, Office of Disease Prevention and Health Promotion, Office of the Assistant Secretary for Health, Rockville, Maryland

**Objectives:** For health science librarians, it can be a challenge to find detailed health disparities data for many population groups. In this session, attendees will learn about Healthy People 2020 (HP2020), a 10-year set of national health promotion and disease prevention objectives, and the Health Disparities Data Tool and Widget. HP2020 contains more than 1,200 searchable objectives and 200 data sources.

**Methods:** This presentation will demonstrate the Healthy People 2020 Health Disparities Data Widget, a portable application that dynamically displays content from www.HealthyPeople.gov on other websites.

The widget introduces users to DATA2020 the system that tracks and measures HP2020 progress. We will review case studies, using scenarios from the audience, to demonstrate the widget's charting features. Participants can view what type of data, descriptive statistics and visual graphics can be drawn from the widget. Furthermore, we will demonstrate how the widget links back to the comprehensive DATA2020 system.
During the live demonstration presenters will explore the new category of the Social Determinants of Health in HP2020 and how they are measured. Librarians will learn about specific data sources and how to download and share data with their users. Presenters also will identify published fact sheets, stories from the field, and evidenced-based resources related to health disparities. The session will conclude with a description of supporting statistical and library resources that can be used to find minority health data and statistics..

**Keywords:** health disparities; data; statistics; minority health; research resources; data visualization; health surveys

## Overcoming Health Disparities by Engaging Patients with the Personal Health Record

**Stephen Kiyoi, AHIP**
Library Director, UCSF Library at Zuckerberg San Francisco General, San Francisco, California

**Lina Tieu**
PhD Student, Department of Health Policy and Management, Los Angeles, California

**Courtney Lyles**
Assistant Professor, School of Medicine, San Francisco, California

**Objectives:** With funding from the National Library of Medicine G08 Grant, we conducted a randomized pilot trial to determine the effectiveness of an online video-based training program to increase use of a portal website among patients in a safety net healthcare setting.

**Methods:** In close collaboration with our patient advisory board and project advisory board, we created an 11 part video based curriculum designed to introduce safety net patients to patient portals and their frequently used features, such as test results, password reset, and medication information. We recruited and randomized 93 patients from 2 primary care clinics to view the training via a) a session with an in-person research assistant b) a link to watch the videos on their own. We also measured patients' confidence filling out medical forms as a measure of health literacy. Using baseline and follow up data, we compared subsequent portal use, perceptions of the importance of the patient portal, experiences with their chronic illness healthcare, and digital health literacy ratings. We also measured the extent to which participants viewed training videos over the course of the study.

**Results:** 18 participants (21%) logged into the portal website during the 3-6 months post-training. These proportions did not differ by in-person vs. take-home training assignment ($p=0.41$), but they were substantially higher than the overall clinic rate of 9% of patients logging into portal in the same timeframe. Those with limited health literacy were significantly less likely to be portal users (35% vs. 7%, $p<0.01$). We also found significant improvements in self-reported skills in using the website ($p=0.03$), patient ratings of digital literacy ($p<0.01$) and medication adherence ($p=0.01$) over time.

**Conclusions:** Health sciences libraries can lead interdisciplinary patient portal engagement programs. Educational videos created through this grant have been integrated in practice, through the Library's patient educator program, the inpatient TV system, the patient portal help website, and with external community partners. With their central place within their institutions and community, health sciences libraries have the potential to serve as "hubs for patient facing IT" to more effectively reduce disparities for patients with limited digital and health literacy.

**Keywords:** patient portals, health literacy, digital literacy, safety net

## Using Secure Tablet Technology to Assess the Health Information Needs of Incarcerated and Justice Involved Persons

**Gail Kouame**
Chair, Research & Education Services, Greenblatt Library, Augusta, Georgia

**J. Aaron Johnson**
Interim Director, Institute for Public and Preventive Health, Augusta, Georgia

**David Young**
Community Health Resources Specialist, MSU Extension, Bozeman, Montana

**Madison Gates**
Assistant Professor, Institute for Public and Preventive Health, Augusta, Georgia

**Anna Fechtor**
Content Specialist, Education Department, Chicago, Illinois

**Objectives:** This presentation describes the first phase of a three-year study that aims to engage incarcerated individuals with health care information and education to enhance their knowledge and use of health resources and services. The first phase is a needs assessment to answer the research question: What are the health information needs of incarcerated persons preparing for re-entry into the community?

**Methods:** A health sciences librarian proposed applying for an NLM Information Resources Grant to Reduce Health Disparities to the Public Health Institute on her campus based on her expertise with a county jail pilot project. The team was awarded funding to collaborate with a mission driven organization providing quality education to incarcerated persons through secure tablet computers. The tablets are currently deployed in 37 jails and prisons in 17 states, and are designed for self-guided learning experiences for low literacy individuals. Project leaders have established agreements with five corrections facilities to use the tablets to conduct a health information needs assessment of incarcerated individuals preparing for re-entry into the community. The study population includes both males and females. The results of the needs assessment will be used to create health literacy training modules to be made available using the tablets.

**Results:** Preliminary data from the needs assessment will be presented

**Keywords:** Health Literacy; Justice-Involved; Tablet Computers; Incarcerated Populations; Partnerships

Tuesday, May 22, 2018, 3:00 PM – 4:25 PM

Room: Embassy C (International Tower, Exhibit Level)

---

# Session: Evidence-Based Practice 2 (EBP-2)

**Moderator: Mary A. Hyde, AHIP**

## Exploring the Role of DistillerSR in Successfully Completing Systematic Reviews

**Rose L. Turner**
Research and Instruction Librarian, University of Pittsburgh Health Sciences Library System, Pittsburgh, Pennsylvania

**Mary Lou Klem**
Research and Instruction Librarian, University of Pittsburgh Health Sciences Library System, Pittsburgh, Pennsylvania

**Objectives:** To assist systematic review teams in screening and abstracting data the library has licensed DistillerSR, a systematic review software tool. The purpose of this study is to explore the value of DistillerSR to the research teams in conducting and producing their systematic reviews. This information will inform further practice and provide evidence to other libraries considering investing in specialized software.

**Methods:** This research project will analyze DistillerSR usage data along with qualitative data collected from researchers who have requested DistillerSR accounts. Additional data will be collected on each systematic review's progress including what stage the systematic review is in, if it is completed, and whether or not it has been published. DistillerSR users will be asked to complete a brief Qualtrics web-based survey that will measure their perceptions on the use of the software. A random sample of participants that completed the survey will be asked to attend a focus group interview to solicit more detailed information on their use of DistillerSR. Interview data will be explored using content analysis to describe the experiences of the researchers.

**Results:** Survey respondents generally reported a positive experience (n=61). 52 out of 61 (85%) rated their experience from extremely positive to somewhat positive. 96% of 24 respondents reported that they found using Distiller improved their systematic review experience in comparison to your systematic review experience without Distiller and 47 of 68 respondents, nearly 70%, planned to use DSR in the future. Focus group discussions identified that participants found DistillerSR to be a valuable tool, although they did experience some frustration and requested additional training.

**Conclusions:** Users required additional support to learn and use Distiller and the library support model has been updated. Distiller provides value to researchers, but users unfamiliar with systematic review methodology may still struggle with the process.

**Keywords:** systematic review, software, DistillerSR, screening, data extraction, reporting, review management

## Common Barriers to Replicability and Retrieval in Systematic Review Search Strategies

**Whitney A. Townsend**
Informationist, Taubman Health Sciences Library, Ann Arbor, Michigan

**Patricia F. Anderson**
Emerging Technologies Informationist, Taubman Health Sciences Library, Ann Arbor, Michigan

**Emily C. Ginier**
Informationist, Taubman Health Sciences Library, Ann Arbor, Michigan

**Mark P. MacEachern**
Informationist, Taubman Health Sciences Library, Ann Arbor, Michigan

**Kate M. Saylor**
Informationist, Taubman Health Sciences Library, Ann Arbor, Michigan

**Judith E. Smith**
Informationist, Taubman Health Sciences Library, Ann Arbor, Michigan

**Objectives:** To identify the most common elements of systematic review search strategies that affect the replicability of the search and the comprehensiveness of the retrieval.

**Methods:** Participants from three cohorts of a systematic review-focused workshop were asked to draft a replicable search strategy based on a brief scenario and a research question from a published systematic review. Participants were provided with three studies that were included in the published systematic review, but not the original systematic review. The researchers in the scenario asked for three commonly-requested limits: date range, inclusion of specific outcome, and publication type. Participants were free to choose to apply these limits or none. Submitted strategies were evaluated by two blinded reviewers for replicability, use of search limits, retrieval of the three provided studies, and retrieval of all studies included in the published systematic review. This study received exemption status from the Institutional Review Board.

**Results:** Seventy-nine workshop participants consented to having their search drafts reviewed for this study. Overall, 76% of the search strategies met criteria for replicability. Searches were considered not replicable if reviewers were unable to reasonably interpret the strategy and retrieve the same number of results, taking into account database variations over time. Sixty-two percent of participants elected to limit their search to the most recent 10 years as requested by the scenario research team, while 66% limited their search to human studies using a variety of limiting techniques. Finally, 71% of participants included search concepts and terms related directly to the outcomes of interest to the team. Overall, 31% of participants with an executable search successfully retrieved all three of the provided sentinel articles, while 10% successfully retrieved all the ten studies included in the published systematic review that the scenario was based on. Second to date limitation, adequate search term generation was the primary barrier to full retrieval.

**Conclusions:** The review of draft search strategies can help instructors and librarians involved in systematic review searching identify some of the most common pitfalls to comprehensive and replicable searches. The results of this review highlight the importance of communicating the bias potential of date limits to research teams, and of appropriate term generation by the librarian.

**Keywords:** systematic reviews, expert searching, search strategies

## Librarians in Systematic Review Teams: Extracting Patterns of Roles and Tasks from the Published Literature

**Emily C. Ginier**
Informationist, Taubman Health Sciences Library, Ann Arbor, Michigan

**Patricia F. Anderson**
Emerging Technologies Informationist, Taubman Health Sciences Library, Ann Arbor, Michigan

**Objectives:** Systematic reviews standards and methodology articles often designate skills and competencies required for those responsible for various tasks in the process, however the competencies and training expected vary by article. The goal of this literature review was to extract the roles and tasks which have been identified as appropriate for librarians to perform as members of systematic review project teams.

**Methods:** A literature search was conducted to identify standards and articles discussing librarian roles in systematic reviews and meta analyses. Articles were also identified through citation mining of included articles. Inclusion and exclusion criteria were identified, and articles assessed by two reviewers. When there was a question regarding inclusion of a role or task, the reviewers followed a consensus building strategy, and documented the results. If the original source was unclear as to which team member performed a required task, the task description and context was assessed by the co-authors. For each identified role extracted from any article, data was captured regarding how many articles of the selected set included that role. The proposed framework went through preliminary external validation through a local systematic review special interest group comprised of librarians across a range of libraries and disciplines, including non-medical experts.

**Results:** Forty-eight articles were discovered through the iterative search process, and librarian roles and tasks extracted from these. Over sixty roles and tasks were identified for librarians in the systematic review process. These fall into the broad groups of project management, methodology, support and training, literature searching, bibliographic management, delivery of results, data management, the publication process, and the post-publication process. Results are described by frequency of specific roles and tasks appearing across the range of articles, and the density of roles and tasks described in specific articles. This data expands upon prior preliminary presentations.

**Conclusions:** Librarians bring a rich range of expertise and competencies to the systematic review process and can be deeply valuable resources and active members of systematic review teams. The full scope of systematic reviews and related methodologies comprises a tapestry of interconnected skills through which librarians form the weft, being connected in various ways at different levels throughout the entire process, with new roles and tasks emerging for the profession to explore.

**Keywords:** systematic reviews, librarian roles, literature review

## Literature Search Methods and Reporting in Highly Cited Systematic Reviews

**Rachael Posey**
Health & Life Sciences Research Librarian and Associate Director, Veterinary Medicine, William Rand Kenan, Jr. Library of Veterinary Medicine, Raleigh, North Carolina

**Objectives:** To examine the reported search strategies and methods in 100 new and 100 older highly cited systematic reviews (SRs) and compare them to the reporting standards from the PRISMA Statement, Cochrane Handbook, and AHRQ Methods Guide for Effectiveness and Comparative Effectiveness Reviews, in order to gain an understanding of the state literature search reporting in the most highly influential SRs.

**Methods:** We examined literature search methods and strategy reporting of the top 20 most highly cited SRs in Web of Science published each year for the last 5 years, as well as 100 highly cited SRs published from 1997 to 2002. Using the PRISMA Statement, Cochrane Handbook, and AHRQ Methods Guide as our guide, we scored each SR on the following criteria: at least one full search strategy reported, all search strategies reported, more than one database searched, search platform reported, number found in each database reported, reported strategies had no major flaws or errors. We then stratified our results by subject area, presence of a librarian as a co-author or in the acknowledgements, and funding source, where available.

**Results:** Of the 240 reviewed SRs, very few (14%) acknowledged a librarian or included a librarian as a co-author. Roughly half (48.3%) were unfunded, and roughly half (47.9%) were on medical or clinical topics. In the 20 year period covered, the median time from final search to publication shrank from 22 months to 15 months. Subject matter and funding source did not appear to impact search methods or reporting. For the overall summary measure encompassing search quality and reporting, there was a small improvement in all SRs in the 2012-2017 set versus the 1997-2002 set (mean difference 0.25 points), with librarian involvement increasing that improvement (mean difference 0.46) The greatest improvements due to librarian involvement were found in search quality (m.d. 0.26 within 2012-2017) and reporting at least 1 search strategy and number in each database (m.d. 0.15 and 0.16).

**Conclusions: Overall, SR search quality and reporting have improved slightly in the last 20 years, but the difference was not found to be significant. Librarian involvement improved most investigated measures; however, the difference there was also not signifiant. Given the low number of highly cited SRs that appear to work with a librarian, there is still a large space for librarian education and involvement in the realm of published SRs.**

**Keywords:** Systematic review methods
Literature searching
Expert searching
Reporting standards

Tuesday, May 22, 2018, 3:00 PM – 4:25 PM

Room: Embassy B (International Tower, Exhibit Level)

---

# Session: Health Information Professionalism/Everything Else (ETC)

**Moderator: Caitlin Bakker, AHIP**

## Measuring Impostor Phenomenon among Health Sciences Librarians

**Jill Barr-Walker**
Clinical Librarian, ZSFG Library, San Francisco, California

**Michelle B. Bass, AHIP**
Population Research Librarian, Lane Medical Library, Stanford, California

**Liz Kellermeyer**
Biomedical Research Librarian, Library & Knowledge Services, Denver, Colorado

**Debra Werner**
Director of Library Research in Medical Education, Crerar Library, Chicago, Illinois

**Objectives:** Impostor phenomenon is the inability to internalize accomplishments while experiencing the fear of being exposed as a fraud. Despite informal discussion and interest among the medical librarian community, no research has been conducted on this topic within our field. Our research objective is to measure impostor phenomenon among US health sciences librarians.

**Methods:** In this mixed methods study, a census of all eligible Medical Library Association members, excluding students, retired members, unemployed members, and international members, was taken from October to December 2017. The census population was 2125, and 728 participants (34%) completed the study. We administered an online survey using REDCap, featuring the Harvey Impostor Phenomenon scale, open-ended questions about coping strategies to address impostor phenomenon at work, and demographic information. Demographic variables included race, gender, age, type of library setting, years of experience, and educational background in the health sciences.

**Results:** Results are pending. We hypothesize that impostor phenomenon will be greater in librarians with less work experience, younger librarians, and those without a health sciences educational background. Descriptive statistics will be used to look for trends and bivariate analyses will be used to examine relationships between impostor phenomenon measurements and other variables (e.g. type of library setting). Thematic analysis will be used to discover themes among strategies for addressing impostor phenomenon.

**Conclusions:** Conclusions are pending; we will have conclusions to present by May 2018. Our conclusions will

help inform awareness-raising and advocacy efforts around this issue.

**Keywords:** Impostor Phenomenon, Professional Development, Health Sciences Librarianship, Identity

## Providing the Updated Evidence for Medical Cannabis Recommendations

**Andrew S. Crow**
Knowledge Consultant Librarian, Allina Health Library Services, Minneapolis, Minnesota

**James Bulger**
Manager, Library Services, Minneapolis, Minnesota

**Pamela Barnard**
Knowledge Consultant Librarian, Library Services, Minneapolis, Minnesota

**Anita Von Geldren**
Knowledge Consultant Librarian, Library Services, Minneapolis, Minnesota

Background
In 2015, Minnesota legalized medical cannabis for nine qualifying chronic conditions. In 2017, the number of qualifying conditions has expanded and Library Services was tasked by Allina Health's Clinical Practice Council (CPC) to provide an updated literature review to amend the evidence-based policy recommendation.
Methods
For the last three years Allina Health Library Services has been optimized within a systematic nine-step model known as the Allina Health Model for Evidence-Based Clinical Decision Making (EBDM). The EBDM supports the development of clinical algorithms, pathways, guidelines, and other policy recommendations. The decision sought by the CPC was to determine whether there is new evidence to support the qualifying conditions.
Results
One librarian conducted an exhaustive literature search in PubMed, Cochrane Library, Google Scholar, and Guidelines.gov from the last 12 years. Of the total 67 articles retrieved, 44 articles from PubMed, two articles from the Cochrane, and one eBook were selected for review. The CPC determined the evidence was weak and of poor quality; however, preliminary data suggests some benefits for four out of eleven certified conditions. One formerly recognized chronic condition turned up less convincing evidence. While there is progress, we still have a long way to go to achieve clarity on the efficacy of medical cannabis for various conditions.
Conclusions
Most of the supportive literature is in very early stages of research. The conclusions in many studies suggest further investigations are needed to better understand the risks and benefits for medical use. It was concluded that a clinical librarian was a key ingredient to the EBDM team and process for Allina Health's updated medical cannabis recommendation. Library Services fill an ongoing need in informed practice in light of the ever-changing medical evidence.

## What We Talk about When We Talk about Collaboration: A Scoping Review

**Timothy R. Roberts**
Associate Curator, Population Health Librarian, Health Sciences Library/NYU Langone Health, New York, New York

**Fred W.Z. LaPolla**
Knowledge Management Librarian, Health Sciences Library, New York, New York

**Gregg A. Stevens, AHIP**
Health Sciences Librarian, Health Sciences Library, Stony Brook, New York

**Objectives:** Librarians have long wrestled with contrasting stereotypes. They are seen as stern yet passive, rigid yet deferential. While collaboration with faculty is seen as a requirement for promotion and advancement, this stereotype can be an impediment to achieving parity within research teams. We are seeking to identify examples of research projects where librarians set boundaries or requirements around their participation.

**Methods:** The authors will conduct a comprehensive search of bibliographic databases that index the medical and library literature on Medical Librarianship to identify descriptions of Medical Librarians collaborating with clinicians or faculty members (e.g. research projects, educational programming, library outreach.) The authors will examine the publications to delineate what boundaries were set for participation by librarians and the other research team members, whether those expectations were met, and if they were not whether accommodations were made. Based on our findings we will attempt to categorize ways in which Health Sciences Librarians have set ground rules. We will also examine the strategies they invoked to ensure compliance, make accommodations, or decline participation. We will also examine whether the methods differ depending on the type of project.

**Results:** The search of multiple databases yielded 1116 unique citations. All three authors conducted the title/abstract review. 173 papers underwent full text review. In the included 45, there was no information on boundaries set by librarians. While all papers described the librarian’s role, more than 38% did not describe the non-library faculty/clinician’s role in the project. 87% of papers portrayed the collaboration as a success, possibly highlighting a positive publication bias. We looked for a specific assertion that the library benefited from the collaboration, which was only present in 49% of the articles. When non-librarians were the first author, they were more likely than librarians to describe mixed outcomes (20% vs. 8.75%) and more likely to describe their roles in projects (40% vs. 30%).

**Conclusions:** The fact that important elements of collaboration were not reported in the included papers does not necessarily mean that they were not present in the collaboration. However, having these elements reported could be useful for librarians trying to replicate projects or evaluate their own collaborations. Additionally, the field of Medical Librarianship could benefit from more reports on the boundaries or ground rules that are set around agreements to collaborate with other faculty and clinicians.

**Keywords:** Research, faculty collaborations, scholarship, workplace issues, self-efficacy, interpersonal relationships

## Transforming Health Information Literacy from Academic to Activist at a Health Zine Workshop

**Rachel Helbing, AHIP**
Director of Library Services for the Health Sciences, University of Houston, University Libraries, Houston, Texas

**Lisa Cruces**
Hispanic Collections Archivist, University Libraries, Houston, Texas

OBJECTIVE: Do-It-Yourself (DIY) Health Zine Saturday was designed to empower students with skills on how to find credible consumer health information and share it through self-published magazines (“zines”), raise awareness of zine collections available at our library, and the zine culture in our local community. This event provided an opportunity for collaboration between Special Collections and the Health Sciences Library.

METHODS: This event was proposed to and approved for funding by our library's Microgrant Committee, then planned by a cross-disciplinary team of librarians. It took place in UH Libraries Special Collections and included a panel presentation and hands-on workshop. Panelists included organizers of Zine Fest Houston and the Hispanic Collections Archivist. The workshop incorporated instruction from the Health Sciences Librarian on finding and evaluating health information, and guidance from local experts on making zines. A resource guide was created for the event which contains links to zine and consumer health resources. During the workshop, the ten participants created zines on healthcare topics of interest to them, including nutrition, reproductive health, and mental health. Several participants donated copies of their zines to be archived by Special Collections. Participants were encouraged to disseminate copies to relevant community members.

RESULTS: We asked participants to take a short survey prior to the workshop to assess their habits and levels of confidence when seeking consumer health information online and used this to inform our instruction approach during the workshop. We asked participants to take a similar survey at the conclusion of the workshop, as well as to reflect verbally on their experiences as a group. We found that their levels of confidence, quality of the resources they planned to use in the future, and understandings of the roles that the library can play in their research all improved. Additionally, all participants stated they were extremely or very likely to visit Special Collections again.

CONCLUSIONS: A panel and workshop focused on health zines was an effective way to connect a small group of participants to zine culture and health information literacy, empowering them to disseminate quality health information on topics they find personally relevant. It also allowed two areas of UH Libraries which rarely get an opportunity to work together, Special Collections and the Health Sciences Library, to collaborate on a project which had an impact outside of health sciences programs. We plan to continue and grow our collaborative efforts going forward.
Keywords: zines, outreach, collaboration, health information literacy, workshop

## What I Wish I Had Known When I Designed My First Clinical Trial and Now Share during Reference Interactions with Novice Researchers

**Terry K. Selfe, AHIP**
Translational Research and Impact Librarian, University of Florida, Gainesville, Florida

**Objectives:** To highlight library-related knowledge and resources I found to be of value to myself and others designing and conducting clinical trials. With a focus on content that librarians can easily integrate into their current instructional interactions with researchers, by the end of the presentation, attendees should be able to synthesize new, value-added instruction sessions for those engaged in clinical research.

**Methods:** When providing instruction to those engaged in, or learning to design and conduct, clinical research, I incorporate the following content as applicable. I advise researchers to locate and become familiar with reporting standards for their trial design (e.g., CONSORT for randomized trials) as early in the process as possible, so they can be sure to design accordingly. I encourage researchers to look for expert panel recommendations for conducting clinical trials in their population, and if they exist, to conform to them or be prepared to justify any deviations. I suggest locating the actual outcomes instrument(s) (e.g., in dissertations/theses databases) and validation studies in their population, and searching for publications reporting scoring instructions and/or normative values (e.g., instrument's user manual), and minimal clinically important differences/improvements (MCIDs/MCIIs) for their instrument so they can address clinical importance of findings, not just statistical significance.

**Results:** The content being delivered has been well received. Students and faculty have stated the information is useful and not covered elsewhere. I have been asked to deliver the content as a guest lecture multiple times. I have also adapted it to be included in one-on-one consultations with graduate students or faculty members conducting human subjects research. The UF Clinical and Translational Science Institute has encouraged

researchers to consult with me when designing their clinical trials, particularly those in the early or brainstorming stages of study development or those using questionnaires or similar instruments to measure outcomes.

**Conclusions:** Health sciences librarians can add value to their instructional interactions with those engaged in clinical research by incorporating into their presentations and consultations as appropriate, information regarding how to locate and utilize the following: 1) reporting standards for the study design of interest, 2) outcomes measurement instruments and supportive documentation, and 3) expert panel recommendations regarding the conduct and design of clinical trials in the study population. Given that these resources are available through long-established library resources, for which librarians already frequently provide training, health sciences librarians are perfectly positioned to successfully impart this useful knowledge to their research communities.

**Keywords:** Clinical trials design; Human subjects research; Library instruction; Reporting standards; Outcome measures; Instruments; Minimal clinically important difference (MCID); Clinical researchers

## Institutional Research Impact Assessment: How I Learned That Good Can Be Better than Perfect (and How I Discussed This with Faculty)

**Kate Nyhan**
Research and Education Librarian, Cushing/Whitney Medical Library, New Haven, Connecticut

**Objectives:** This paper describes how a librarian collaborated with faculty and staff on a practical, evidence-based approach to institutional research impact assessment at a school of public health. This project investigates the data quality and required resources for multiple approaches to institutional assessment of faculty publications -- allowing stakeholders to make informed decisions about a workflow for good-enough citation data.

**Methods:** Who are institutional research impact stakeholders? Administrative leaders discussed the intended application of institutional research impact data; researchers described their information behavior in generating required annual publication lists; Faculty Affairs staff explained their existing processes and output; librarians shared important lessons from vendor trials and from an earlier citation analysis project which achieved high-quality data through a resource-intensive process.
In this context, the author is investigating several methods of generating a comprehensive list of publications by authors affiliated with this school. These approaches use funding and affiliation data in Scopus, Web of Science, Ovid MEDLINE, PubMed, and a novel workflow combining PubMed, EDirect, and OpenRefine. By comparing these methods' results with an existing, faculty-generated database of publications, we can better understand the validity of each method (including the status quo) and compare costs (in time) and benefits (in data quality).

**Results:** This project is in progress and will be completed before MLA 2018.

**Conclusions:** As a result of this project, these outcomes are anticipated:
- better understanding of data quality and required resources for each approach to institutional research impact assessment
- better understanding of the information behavior of faculty and staff who use bibliographic databases, citation managers, word processors, ORCID, and other tools to manage lists of publications
- recommendations to the dean of the school on a preferred author affiliation and ORCID
- opportunities to engage with faculty about research impact as a concept that extends beyond counting citations

**Keywords:** research impact, institutional assessment, citation analysis, eDirect, e-Utilities, faculty outreach

Tuesday, May 22, 2018, 3:00 PM – 4:25 PM
Room: Embassy E (International Tower, Exhibit Level)

---

# Session: Instruction and Instructional Design 3 (ID-3)

**Moderator: Sally A. Gore**

## Identifying Research Data Management in Nursing Doctoral Programs


**Rebecca Raszewski, AHIP**
Associate Professor & Information Services & Liaison Librarian, University of Illinois at Chicago Library of the Health Sciences Chicago, Chicago, Illinois

**Abigail Goben**
Associate Professor & Information Services & Liaison Librarian, Library of the Health Sciences Chicago, Chicago, Illinois

**Martha Dewey Bergren**
Clinical Associate Professor, College of Nursing, Department of Health System Science, Chicago, Illinois

**Krista L. Jones**
Clinical Associate Professor and Campus Director, College of Nursing, Department of Health System Sciences, Champaign, Illinois

**Catherine J. Ryan**
Clinical Associate Professor, College of Nursing, Department of Biobehavioral Health Science, Chicago, Illinois

**Alana D. Steffen**
Research Assistant Professor, College of Nursing, Department of Health System Science, Chicago, Illinois

**Susan C. Vonderheid**
Clinical Assistant Professor, College of Nursing, Department of Women, Children and Family Health Science, Chicago, Illinois



**Objectives:** Research data management (RDM) strategies are increasingly important as nurses use larger and more complex electronic data sets. The status of RDM education within nursing doctoral programs is currently unknown. A standard resource that identifies current educational expectations of these programs is the nursing student handbook. The purpose of this study is to identify the inclusion of RDM within doctoral nursing student handbooks. Findings will be used to determine gaps to improve current RDM practices and foster collaborations among librarians and nursing faculty.

**Methods:** An interdisciplinary research team of library faculty with expertise in RDM and nursing faculty in doctoral programs was established to conduct this study and serve as content experts. A list of over 350 doctoral programs was obtained from the American Association of Colleges of Nursing. Program websites were searched to locate doctoral program handbooks and available handbooks were downloaded for analysis. A textual review of the handbooks was conducted to determine whether RDM was mentioned and, if so, whether it was located in course descriptions, project requirements, institutional policy, or resources. Descriptive statistics will be used to compare presence of RDM by program and institution type, Carnegie Classification Basic status, program's geographical location, and the extent to which RDM is represented within the handbooks.

**Results:** Preliminary results based on review of one-third of doctoral programs (n=120) suggests that overall there is little attention to RDM and related practices. Common terms and phrases used to identify RDM included data analysis, collection of original data, and data privacy concerns primarily related to HIPAA and IRB. RDM appears to be more prevalent in PhD (research focused programs) compared to Doctor of Nursing Practice (clinically focused programs) handbooks. RDM is most frequently mentioned within student competencies, course descriptions, and PhD/DNP proposal guidelines.

**Conclusions:** Interdisciplinary collaboration between library and nursing faculty is critical to develop a comprehensive approach to the implementation of RDM education in doctoral nursing programs. Our findings suggest limited education and inclusion of RDM content currently, especially within DNP programs. This might be related to the focus of PhD programs on the generation of original research. Further inquiry is needed to complete the review of the program handbooks and to determine the opportunities for collaborative instruction in PhD/DNP programs.

Research data management, data education, doctoral nursing programs, handbooks

## Leading the Way Using Virtual Reality in Medical Education to Teach Empathy

**Elizabeth Dyer, AHIP**
Associate Dean of Library Services/ Research and Teaching Librarian, University of New England, Portland, Maine

**Barbara Swartzlander**
Research and Teaching Librarian, Library Services, Biddeford, Maine

**Marilyn Gugliucci**
Professor & Director Geriatrics Education and Research, College of Osteopathic Medicine, Division of Geriatric Medicine, Biddeford, Maine

**Objectives:** To adopt technology that will teach medical students to be empathic with older adults through virtual reality (VR) software that allows them to embody a seventy-four-year old man with functional deficits, and to familiarize medical students with information resources related to the health of older adults.

**Methods:** At a small university with a medical school, the project uses new software developed by an emerging company that creates immersive VR experiences for health training. Users become Alfred, a seventy-four-year-old man who has macular degeneration and hearing loss, thus experiencing these conditions from the patient's perspective as he interacts with his family and doctor. Pre- and postassessment surveys measure the impact of the experience. Funded by an NN/LM technology award, librarians and faculty partner to integrate the experience into the curriculum using the Prepare, Embody, Reflect model. Geriatrics faculty introduce the

assignment to first-year medical students, including an overview of NLM resources related to older adult health. Students go to the library at their own convenience to access the equipment, take the preassessment survey, and embody Alfred. The postassessment survey allows students to reflect on the experience.

**Results:** The project successfully introduced an innovative new teaching modality to the medical curriculum. Medical students in the first cohort indicated that it enhanced their understanding of age-related health problems and increased their empathy for older adults with vision and hearing loss. In the second year of the project, results from the second cohort of medical students show statistically significant changes in understanding and empathy. The project was successfully extended to physician assistant students and also showed significant change. Experiences to date help determine best practices, and evaluation has led to planned changes in implementation and assessment for future groups.

**Conclusions:** Virtual reality immersion training is an effective teaching method to help medical and physician assistant students develop empathy, and is a budding area for library partnerships. Success of this project will ensure its continuation in the medical and physician assistant curricula, and it has already expanded to other health professions programs including nursing and physical therapy. New VR modules related to older adult health, involving Alzheimer disease and also end-of-life conversations and decision-making, will enhance training for health care professionals that supports the model of person-centered care that is comprehensive and empathic toward older adult patients.

**Keywords:** virtual reality, empathy, medical education, faculty-librarian collaboration, technology, older adults

## Nurturing Statistical Literacy in Medical Students

**Abraham Wheeler, AHIP**
Health Sciences Librarian, MSU Libraries, East Lansing, Michigan

**Objectives:** Statistical literacy is a foundational skill physicians need for evidence based medicine. Despite this, physicians repeatedly report feeling uncomfortable or ignorant about statistics reported in medical literature. To address this knowledge gap the librarian at a large medical school rebuilt the EBM curriculum to focus on increasing students statistical literacy through a range of practical patient scenarios and lectures.

**Methods:** Statistical literacy is the ability to read, work with, analyse and argue with data and statistics. In collaboration with medical school faculty, the librarian totally redesigned the EBM curriculum to focus on practical statistics. A combination of lecture, hands on practice,and patient encounter scenarios are utilized longitudinally across the curriculum to present statistics in clinically meaningful ways. For further value, the content was also mapped to statistical questions on the USMLE and COMLEX. The goal of the curriculum is not to turn students into statisticians, but rather to give them the tools they need to make sense of the numbers and charts being presented in the research literature, and then apply that knowledge to patient care.This paper will present examples of curriculum content, training materials, literature evaluation handouts and will demonstrate practical steps attendees can implement at their institution.

**Conclusions:** This new librarian led initiative has tripled the number of teaching sessions by the liaison librarian, as well as earning him a seat at curriculum design committees and faculty meetings. Through the hard work of mastering the skills and knowledge necessary to lead this revision, new and deep roles for the librarian with the medical school curriculum has developed. Statistical literacy shares a foundation with information literacy, and as a result provides a natural and professionally appropriate avenue for librarians to deepen their role in their medical school curriculum.

**Keywords:** Statistics EBM Evidence Base Medicine curriculum literacy education medical students

## Social Media and Online Professionalism in the Medical School Curriculum

**Enid Geyer, AHIP**
Associate Dean for Information Resources and Technology, Schaffer Library of Health Sciences, Albany, New York

**Elizabeth Irish, AHIP**
Assistant Professor, Schaffer Library of Health Sciences, Albany, New York

**Amanda Hagzan**
Assistant Director for Instructional Technologies, Schaffer Library of Health Sciences, Albany, New York

**Objectives:** Studies demonstrate that medical students use and contribute to social media but they don't always post responsibly. Recognizing the need for education, online medical professionalism was added to the Library's longitudinal informatics theme (LMI) in 2014. Learning modules were integrated into year 1 and 3. Objectives include identifying standards for behavior and implications of use on confidentiality and patient trust.

**Methods:** The content was first introduced into the year 3 OB/GYN clerkship in a 2-hour interactive class. This clerkship was selected because of the well-defined ACOG (American Congress of Obstetricians and Gynecologists) guidelines and the opportunity for integration. The curriculum was developed by library faculty in collaboration with the Clerkship Director using guidelines, news stories, case studies and self-auditing to teach acceptable social media behavior. Sessions are co-facilitated by library and clinical faculty.

Feedback from year 3 students in July prompted us to incorporate the topic into the new student technology orientation in August. It was also added as a required LMI year 1 self-directed online module in 2015. Content is reinforced in another longitudinal theme, Health Care in Society, in which library faculty co-facilitate some small group sessions.

**Results:** Year 3 OB/GYN clerkship evaluations showed that the majority of responding students in 2015-2016 (70%) and 2016-2017 (66%) ranked the clinical significance and integration in the clerkship as good, very good, or excellent and felt the time and effort required was appropriate. In-class polling supports these results, with over 76% of students responding they learned something new and their online behavior would change as a result. Early results from 2017-2018 (n=35 students) indicate 51% have self-audited since LMI year 1. Evaluation results for year 1 2015-2016 indicate that 85% of students ranked the module as good, very good, or excellent.

**Conclusions:** The technology orientation and self-directed learning module in year 1 and the discussion session in year 3 improve student awareness of their online presence and influence their online behaviors. As the 2017-2018 year three students are the first cohort to have had the year 1 online professionalism instruction, the curriculum has been adapted to determine if the year 1 objectives were adopted by the students. Future plans include possible integration in clinical skills and inter-professional education, as well as development of a social media instruction curriculum for residents and faculty.

**Keywords:** Social Media; Online Professionalism; Medical Education; Curriculum-Based Instruction

## Using Pre- and Post-Questions about Prior Information-Seeking Frustrations to Assess Student Learning

**Adelia Grabowsky**
Health Sciences Librarian, Auburn University LIbraries, Auburn, Alabama

**Objectives:** Library instruction has evolved from a focus on simply what is being taught to an emphasis on what students are learning and more specifically, are they able to apply what they have learned to their own experience. This study sought to determine if pre- and post-questions about a prior information seeking frustration could be helpful in assessing student learning.

**Methods:** Setting/Participants: Twenty-six students enrolled in a Masters of Speech Language Pathology program.
Methodology: Students were asked to reflect on the last time they worked on a paper or project and list the biggest problem/frustration they encountered. After library instruction, students were asked if they had learned anything that might help with their previous frustration. Students who answered yes or maybe were also asked to explain what they had learned that would help. Responses were coded and analyzed to determine if students were able to appropriately apply information from class to solve their previous problem.

**Results:** Finding full text was the most often mentioned frustration for students (n=18) with far fewer students reporting difficulty in finding quality sources (n=4), or problems with creating and/or refining a search (n=3). Post-instruction, 25 students indicated "yes" and one student indicated “maybe” they had learned something that would help with their previous problem. When responses were analyzed, 65% of students were able to articulate a workable solution to their previously recorded problem, 12% offered a solution appropriate for a different problem, and 19% of the responses were not specific enough to analyze. Only 4% (n=1) of students listed an inappropriate solution.

**Conclusions:** All students believed they had either definitely or maybe learned something that they could apply to become more effective information seekers, but more importantly, 77% were able to articulate how they could solve a problem they had previously encountered. Students being able to appropriately use information taught in class to solve previous problems is an indication that learning has occurred. An unexpected finding is that 69% of students listed finding full text as their biggest problem in information seeking. More research is needed to understand why students struggle with finding full text and how that process could be more intuitive.

**Keywords:** Information literacy
speech-language pathology students
information seeking
assessment
student learning

Tuesday, May 22, 2018, 3:00 PM – 4:25 PM
Room: Embassy A (International Tower, Exhibit Level)

---

# Session: Lightning Talks 5

### Moderator: Helen-Ann Brown Epstein, AHIP, FMLA

## Anatomy Touch Table Project

**Roger Russell, Jr.**
Associate Director, Laupus Library, Greenville, North Carolina

**Marlena Barber**
Assistant Director of Collections & Historical Services, Laupus Health Sciences Library, Greenville, North Carolina

**Objectives :** The library has seen great usage in its anatomical model collection but struggles to meet patron demand during peak times. The objective of this project was to improve patron experience and utilization of anatomical resources, to better anticipate and understand period of high patron demand for anatomical models, and to ensure maximum user availability of models.

**Methods :** Usage statistics were analyzed on anatomical models and web-based anatomical model resources. Anecdotal data from librarian and staff interaction with library patrons was also recorded. The library launched several new approaches to achieve the objective including purchasing a large Touch Table on which electronic anatomy resources can be used, working directly with faculty and students to better anticipate and understand periods of high demand, and ordering additional models.

**Results :** Successes have been increased use of the physical anatomical model collection and increase in the types of students who are using the collection. Challenges have been convincing students or teaching faculty to use the electronic anatomy resources on the Touch Table, despite sponsoring classes, working with the university's tutoring center, and trying various locations. Other challenges have been making the anatomical model collection available "just in time" during periods of high demand. A reservation system was piloted but quickly abandoned due to unanticipated problems using text messaging for alerts that a model was ready for use.

**Conclusion :** Anatomical models resources are in demand on most health sciences campuses. Physical anatomical models are still most preferred, even when good and more accessible electronic versions are made available. Transitioning from physical to electronic for anatomical model resources will require partnership and buy-in from teaching faculty resulting in gradual culture change.

**Keywords :** anatomical models, technology, touch table, access services, user services

## Developing a Library Exhibit on Local Public Health History

**Beth Auten, AHIP**
Health & Human Services Librarian, The University of North Carolina at Charlotte, Charlotte, North Carolina

Objectives: A health sciences librarian at a public research university partnered with a public health policy scholar, to research local public health history and develop an exhibit of materials held by the library during National Public Health Week in April 2018.

Methods: This project arose from a conversation between the dean of the health sciences college, the department chair of public health, and a faculty member who approached the library about developing an exhibit. Working with material from the library's special collections and archives, the health sciences librarian and public health scholar curated the exhibit. Challenges faced by the librarian included communication across library and academic departments and working around library renovations that impacted access to the collections and available work space.

Results: The exhibit was hosted on the first floor of the library during National Public Health Week in 2018. The health sciences librarian was also able to become more comfortable with material from the library's special collections.

Conclusion: The exhibit has provided a way for a librarian new to an institution to form relationships within and outside the library, and to learn more about a new subject area (public health.) Informal follow-up with public health administration and faculty and with the special collections department will inform future projects and hopefully allow for additional collaboration.

Keywords: public health, exhibits, special collections, collaboration, partnering, relationship building

## Hailing Your Champions: Interdisciplinary Reproducibility in Action

**Tisha Mentnech**
Research Librarian, University of Utah Spencer S. Eccles Health Science Library, Salt Lake City, Utah

**Melissa L. Rethlefsen, AHIP**
Interim Executive Director & Librarian, Spencer S. Eccles Health Sciences Library, Salt Lake City, Utah

**Mellanye Lackey**
Associate Director for Education and Research, Eccles Health Sciences Library, Salt Lake City, Utah

**Objectives:** By taking an institutional approach to tackle a controversial and significant issue we aim to inform multiple audiences at the University on a variety of research reproducibility issues through workshops, grand rounds, coalition building, and organizing in a day long conference.

**Methods:** A partnership formed between the Vice President for Research and the Deputy Director at the library in 2015 sprung from mutual interest on ways to get in front of the reproducibility crisis the 2016 research reproducibility conference began the institutional efforts. From the success and evaluations of the first conference the the 2017/2018 school year with a weekly Grand Rounds sessions on various topics about research reproducibility, plus quarterly Coalition meetings, to allow faculty and staff at the University to come together around reproducible research, finishing with another conference and short course. We are providing space and time to educate and discuss a myriad of issues from institutional questions to journal policies. The goal for this talk is to highlight the importance of working with and supporting institutional champions, and giving

them the platform to disseminate the importance of reproducibility.

**Results:** Our library's approach to research reproducibility has achieved institutional support through the Office of the Vice President for Research, we have received over $70,000 dollars in funding support for workshop and all day conference. Due to the success of the 2016 Research Reproducibility conference, the Center for Clinical and Translational Sciences made research reproducibility a major theme of the renewal grant, and our Grand Rounds series on YouTube has over 1,500 views on 21 videos. We will have more results after the conference and course in June 2018 that will drive our 2018/2019 research reproducibility efforts.

**Conclusion:** After the short course and conference are complete we will collect evaluations to implement any adaptations to our future reproducibility efforts. We plan to continue to lead the institutional efforts of mitigate the reproducibility crisis and offer a place of conversation on how to improve overall institutional reproducibility.

**Keywords:** Reproducibility, Interdisciplinary partnerships, Replicability, Collaborations

## Hands-On Search Activities for Live Online Instruction

**Hannah Schilperoort**
Information Services Librarian, University of Southern California, Los Angeles, California

**Objectives:** A liaison librarian for an online program presents ideas for hands-on literature searching activities for live online information literacy instruction via video conferencing.

**Methods:** Online information literacy can be just as engaging and interactive as in-person instruction. Video conferencing applications, such as Adobe Connect and Blackboard Collaborate, have functions and tools that librarians can utilize to develop engaging individual and small group hands-on search activities.

The speaker will share two examples of hands-on search activities for two different learning outcomes.
1. Learning outcome: students practice search skills and start searching for literature on individual topics
a. Tools: Google spreadsheet, screen sharing, and polls
2. Learning outcome: skills assessment for keyword and subject searching
b. Tools: Breakout rooms, screen sharing, and polls

**Keywords:** Online education, distance education, instruction, information literacy, database searching, assessment

## I'm a Real Boy Now: Moving from Excel to Factor Analysis for Analyzing Survey Data

**Ardis Hanson**
Assistant Director, Research and Education, Shimberg Health Sciences Library, Tampa, Florida

**Objectives:** The objective of this lightning talk is to describe the use of factor analysis as a way to better understand the relationships that can be deduced from survey data using this method rather than Excel and to substantiate the power of interdisciplinary teams (librarians and experimental psychologist) in the conduct of library research.

**Methods:** We conducted a mixed methods survey in Qualtrics® examining the time librarians required to accomplish the discrete tasks comprising a systematic review (Interview, Search Strategy, Translating Search, Documenting, Delivering, Writing, and Additional Tasks). We used SPSS to conduct an exploratory Factor Analysis, specifically a Principal Components Analysis (PCA) with a VARIMAX rotation. The PCA created a

parsimonious model relating the durations of various tasks to the librarian's experience level. The VARIMAX rotation maximized differences in the factor structure and clarified relationships among selected measures.

**Results:** The PCA showed heavy loadings for Search, Translation, and Writing (Component 1: Information Processing ) and for Interview and Instruction loading (Component 2: Interpersonal Instruction/Training). The VARIMAX rotation essentially duplicated the PCA. Analyses identified potential clusters of tasks and their relationship to the level of librarian experience. The experience level of the librarian was positively related to interview and instruction. Experience level was unrelated to the amount of time spent on search, translation, and writing. The latter tasks suggest a relatively immutable cluster unaffected by the experience level of the librarian, but determined perhaps by situational or project variables.

**Conclusion:** By adding an experimental psychologist to our working group and employing FA, we were able to observe in finer detail relationships that differed from those originally hypothesized in Excel. We contend factor analysis is particularly well suited for the examination of library survey data and meets our library's current focus on professional development and MLA's Competency 5: Evidence-Based Practice & Research, which encourages the use of other research methodologies for the interpretation and presentation of statistical and data analyses. Our findings appear in an upcoming article in the Journal of the Medical Library Association.

**Keywords:** systematic reviews; factor analysis, time-task studies, interdisciplinary teams; evidence-based practice;

## Impact of Social Media-Like Document Sharing and Networking Sites on Traditional Interlibrary Loan Service: Results of a Small Study

**Kevin O'Brien**
Head, Access to Resources Department, UIC Library of the Health Sciences, Chicago, Illinois

**Objectives:** Commercial social media-like document sharing and networking sites represent a new potential source of scholarly papers. The intent of this small study was to measure what impact, if any, a representative commercial document sharing and networking site may have on academic medical library user demand for published literature, traditionally satisfied by interlibrary loan service.

**Methods:** The citations of four weeks of filled interlibrary loan requests submitted by users of the UIC Library of the Health Sciences Chicago were examined against the contents of ResearchGate.net, a high profile commercial social media-like document sharing and networking site. Two criteria were searched for in the database: 1) the presence of the citation of the published work and 2) the availability of full text content.

**Results:** The total number of citations searched was 108. 100 of those citations were present in the database (93%). Of the total number searched, 16 (14%) citations contained the full text of the papers.

**Conclusion:** While not an insignificant number, the total percentage of full text papers available is not currently enough to substantially impact user demand for traditional interlibrary loan service. This low number may be a result of a recent action taken by publishers against document sharing in violation of copyright law. If more authors become familiar with the permissible sharing of pre-print manuscripts, the impact of commercial social media-like document sharing and networking sites like ResearchGate.net on user demand traditionally met by interlibrary loan service may increase.

**Keywords:** Resource Sharing, copyright, interlibrary loan, scholarly communication, disruption

## Improving the Accessibility of Instructional Videos...Not as Scary as You Think

**Charlotte Beyer, AHIP**
Instruction and Reference Librarian, Rosalind Franklin University of Medicine and Science, North Chicago, Illinois

**Objectives:** The purpose of this presentation is to outline tips, tricks, and lessons learned when creating videos with accessibility in mind. Special focus will be strategies for creating scripts and caption for users who cannot see audio or video.

**Methods:** In 2017, the instruction and reference librarian sought to improve the instructional materials' accessibility to users with all abilities. She noticed the default YouTube captions were often incorrect, and needed revision. Having a limited budget, paid captioning software was not an option. The librarian created a plan for creating accurate captions which included identifying the main objectives of the videos, and writing a script around those objectives with recording the video last. One of the struggles of speech to text software is it needs time to learn the recorder's voice which is why the default text does not always initially match. The librarian easily corrected the text by using blocks of the script created earlier. By the end of 2017, most of the videos had correct captions and a process had been set for future video creation.

**Results:** After the librarian began the project, the library was informed that one of the incoming students needed accommodations and student services was impressed the library had already taken steps to make content more accessible. Another benefit was that students remarked on how the content was more concise, and that reading captions reinforced what was happening on the screen as well. Having a script reinforced the importance of objectives , and discouraged rambling so concepts were clearer. So designing for all users improved the overall quality of the instruction.

**Conclusion:** Many times people think improving their materials' access for all users is difficult. However this presentation shows that if there is a process, designing for all actually saves time in the end, because it reaches the most users possible. Creating accurate captions is one small way to improve your materials accessibility, and helping all students get the most out of the information resources available to them.

**Keywords:** accessibility, instruction, video tutorials, instructional design, online learning

## Library as Tenant: A Born Digital Library Embedded in a New Medical School Building

**Nell Aronoff**
Senior Assistant Librarian, University at Buffalo, Buffalo, New York

**Objectives:** In January 2018, a new medical school building opened at the University at Buffalo. The building contains a small, centrally-located library. The goal of this presentation is to examine the challenges and opportunities faced by the library as it began operating in a space that it does not manage.

**Methods:** A library task force was charged with making recommendations about services, hours, staffing, technology, collections, and security. The group generated a report outlining their recommendations so that we could hit the ground running. The reality was that there were unforeseen challenges along the way.

**Results:** Communication issues and confusion about whether the library or the medical school was addressing certain recommendations meant that the library was not staffed when the building initially opened. It has been staffed since April 2018. Besides internal organizational and administrative changes, the library has had to adjust to being a tenant in the building. This often means that the library depends on the medical school to make or carry out decisions. As a digital library, image has also been a challenge. Greater visibility, proximity to

patrons in the school and on the medical campus, and involvement in the curricular redesign process are opportunities that we hope to capitalize on.

**Conclusion:** Although being a tenant in the new space has presented some challenges, we are working to overcome them. We are fortunate to have a place in the building and a growing role in the curriculum.

**Keywords:** born digital library, medical school library, library space

## Reflecting Art in Nursing Practice: Developing a Program to Support Mid-Career Nurses

**Lita Anglin**
Nursing Research and Education Librarian, NYU Health Sciences Library, New York, New York

**Objectives:** A workshop series was created in partnership with a metropolitan art museum, departments of nursing and an academic health sciences library. Nurses engaged in hands-on exercises and reflective discussions of images to enhance clinical practice, reflective capacity and narrative skills. Four one-hour workshops emphasized themes of description, observation, communication, empathy and the underlying concept of art for self-care.

**Methods:** Humanities and humanistic learning experiences are well described in medical education and have become a standard part of medical education curricula in order to orient clinicians in training to a holistic view of patient care. Though less frequently and extensively described, similar humanities education experiences are a part of nursing education programs both at the undergraduate and graduate levels. This program was developed to support clinical and personal capacity for mid-career nurse professionals at their hospital workplace over a series of 4 weekly one-hour workshops led by a professional museum educator and facilitated by a nursing librarian. Cohorts were held summer 2017, fall 2017 and spring 2018. We collected qualitative feedback from participants throughout the learning experience.

**Keywords:** nursing, humanities, art, nurses, embedded librarians, workforce, interdisciplinary, interprofessional

## Scholarly Connections: Assessing Interdisciplinary Collaboration through Citation Data Exploration

**Carl E. LEAK**
Systems Biology and Life Sciences Librarian, George Mason University, Lorton, Virginia

**Kimberly Hoffman**

**Vamsi Kunaparaju**

**Objectives:** The science team consisting of librarians, staff and a graduate assistant sought to analyze citation data as a response to a strategic goal of the university to encourage more interdisciplinary research. Participants will be made aware of the direct and indirect challenges and rewards of such a partnership and how the library will move forward after the first year.

**Methods:** To document all processes and procedures for the data analysis project using the Center for Open Science's OSFramework (OSF). Thompson Reuter's Web of Science was the tool used to download the bibliometric data. In addition to the citation data, biographical data of each researcher was also saved and

stored in the OSF. The institutes vary in scope and include focuses ranging from proteomics, bioinformatics, serious gaming, and neuroscience.

**Results:** This project is ongoing and the team has completed the citation analysis of three institutions. Following the data analysis stage, visual representations were produced to display the trends in research and the interdisciplinary collaborations that exist among faculty. Since the makeup of each institute is different, there are unique challenges that had to be addressed on a case-by-case basis. For example, the volume of research dictated how the data was assessed and presented.

**Conclusion:** The team has completed the analysis of three institutes. The project will continue because it allows the library to contribute to a specific strategic goal that has become a primary objective of the university. In addition to the partnership with the engineering school where the graduate assistants are enrolled, the library is able to generate a narrative of interdisciplinary research driven by data. Also, the library is able to use the data in a traditional sense which is to inform collection development, outreach initiatives, and programming. Graduate assistants also have the opportunity to use their skills in a practical manner.

**Keywords:** Interdisciplinary Research, Data Analytics, Graduate Assistants, Bibliometrics

## Writing Test Items for National Board of Medical Examiners Shelf Exams: Innovative Involvement in Medical Education Assessment

**Rebecca C. McCall, AHIP**
Clinical Librarian, University of North Carolina-Chapel Hill, Chapel Hill, North Carolina

**Objectives:** The American Medical Association's Accelerating Change in Medical Education Consortium is a group of medical schools committed to innovative improvement in the medical education system. These 32 medical schools are working together to transform future physician training. Part of these curricula improvements are the creation and testing of new assessments, including exams from the National Board of Medical Examiners.

**Methods:** In fall 2017, the AMA put out a call to member schools in the consortium for medical education experts to create additional test items for a new NBME shelf exam being trialed by some consortium medical schools. Due to the strong partnership at my institution between the medical school and health sciences library, our university sent a medical librarian as our representative to the AMA workshop on creating NBME multiple-choice questions and writing items for this shelf exam. The medical librarian was assigned the subject area of evidence-based practice within health systems science. This was a unique, innovative opportunity to participate in the national conversation on changing medical education, collaborate with physicians and others involved in medical education initiatives, and contribute to the assessment of EBP learning in undergraduate medical education.

**Results:** AMA hosted the test item writing workshop at their headquarters in Chicago in fall 2017 and all attendees discussed current issues in medical education assessment, research innovations by consortium members, and best practices for NBME test item writing. The attendees then worked in breakout groups by subject to create sample test items and then discussed them with the broader group. All attendees then returned to their institutions to create their lists of test items. The medical librarian wrote theirs after soliciting input on important question topics from colleagues and through examination of the evidence-based practice and health systems science literature.

**Conclusion:** This unique medical education project was a learning experience on undergraduate medical education assessment and a unique way to expand our institution's partnership between the medical school and the health sciences library. The lightning talk will focus on lessons learned and applications for other health sciences librarians.

**Keywords:** medical education, student assessment, interdisciplinary collaboration

## But What Do All These Metrics Mean? A Review of Citation Ontologies and Current Research on Sentiment Analysis of Citations and Altmetrics

**Virginia Pannabecker, AHIP**
Health Sciences Research Support Coordinator, University Libraries, Blacksburg, Virginia

**Objectives:** Citation metrics and altmetrics for a work or works are sometimes presented as simple numbers, possibly because conducting deep level analysis of such metrics to add qualitative description can be time consuming. This literature review study investigates: what systematic (including automated) approaches are being used to categorize, analyze, and summarize qualitative characteristics and implications of citations and altmetrics?

**Methods:** The author is conducting a literature search of several scholarly databases as well as web searches to identify existing classification ontologies for qualitative categorization of citations and altmetrics; and to identify research studies that focus on qualitative categorization, description, and/or analysis of citations or altmetrics for research and scholarship. Search terms will include: (citations OR altmetrics) AND ("sentiment analysis" OR "qualitative description" OR categorization OR classification). Full search strategies used for each database and web search, sources searched, result numbers, and a bibliography of selected results will be shared along with study results. Ontologies and studies selected for inclusion in the review will also be examined for their usefulness with Health Sciences fields including any particularly relevant aspects (ontology fields/categories) or use case examples (studies focused on health sciences / biomedical works or researchers).

**Results:** Highlighted summary review results will be presented at the lightning talk with a link to study documents for more information: literature review search terms and sources, a bibliography of relevant review results, a list of identified ontologies with a table showing similarities and differences and examples of use of such ontologies in health sciences or other research studies, and an annotated bibliography of identified research studies using either automated or systematic manual methods to categorize and analyze citations and altmetrics to describe the meaning of the impact of a given work or works. Health Sciences specific examples will be emphasized.

**Conclusion:** The completed study will provide a highlighted summary of methods and list of example studies or documents demonstrating current methods to categorize, analyze, and describe the qualitative characteristics and implications of citations and altmetrics for scholarly and research works. Health Sciences specific examples will be emphasized.

**Keywords:** citation analysis, altmetrics, research metrics, sentiment analysis, qualitative description

## The National Library of Medicine Biomedical and Health Research Data Management for Librarians Online Course: A Student's Perspective

**Sheila W. Green**
Bryan Campus Librarian, Medical Sciences Library, Bryan, Texas

**Objectives:** To share a "student" experience with the Biomedical and Health Research Data Management for Librarians training program developed by the NNLM Training Office, supported by the National Library of Medicine, and per Director Patricia Brennan, RN, PhD, designed to "…offer the kind of training that will develop librarians' skills and develop practical and actionable data services at their own institutions."

**Methods:** The 8-week course was administered via Moodle with weekly readings and videos, online discussion boards and an assignment led by course administrators and a co-teacher. Program topics included an overview of data management, choosing appropriate metadata descriptors or taxonomies for a dataset, addressing privacy and security issues with data, and creating data management plans. Each student, with the help of their data librarian mentor, worked on a capstone project relevant to their own institution, culminating in an in-person Summit at the National Institutes of Health, April 10-11, 2018.

**Results:** A network of over fifty participants - students, teachers, and mentors - from many different types of institutions across the country worked together to build skills and identify actionable services that could apply to each student's situation. A letter of support expressing both protected time and institutional commitment to the course was required at application. Both were needed to produce a successful capstone project. The student also needed time management, focus, and a willingness to be comfortable with ambiguity.

**Conclusion:** The course helped this librarian adapt her current skillset, transform her expectations about the delivery of data management support, and work with the library team to lead the delivery of a growing portfolio of researcher services.

**Keywords:** Research data management, skills development, data services, researcher services

Tuesday, May 22, 2018, 3:00 PM – 4:25 PM

Room: International Ballroom South (International Tower, Ballroom Level)

---

# Session: Transforming Together: Innovating Consumer Health Information Development for Public Librarians (PLP-1)

**Moderator: Catherine A. Smith**

## What the Health: Helping Public Librarians Adapt, Transform, and Lead in Consumer Health Information

**Jessica A. Koos, AHIP**
Health Sciences Librarian, Stony Brook University Health Sciences Library, Ronkonkoma, New York

**Jamie Saragossi**
Head, Health Sciences Library, Health Sciences Library, Stony Brook, New York

**Gregg A. Stevens, AHIP**
Health Sciences Librarian, Health Sciences Library, Stony Brook, New York

**Salvatore Filosa**
Marketing & Outreach Librarian, Adult Reference, Port Jefferson, New York

**Objectives:** To determine if attending workshops on the use of various consumer health resources provided by the National Library of Medicine (NLM) will improve public librarians' and library science students' ability to provide consumer health information services.

**Methods:** Three individual workshops were held to teach MedlinePlus, TOXNET, and various genetics resources including Genetics Home Reference (one topic per workshop). Participants were instructed on best practices to find basic consumer health information. Each workshop was taught by an academic Health Sciences Librarian. After the workshop, each participant provided feedback via a survey to measure the efficacy and impact of the content presented. This project was funded by the National Network of Libraries of Medicine (NNLM) Health Information Awareness Award.

**Results:** All of the participants reported that they learned about one new resource or skill during the workshops. The majority of participants reported that they planned on using at least one resource or skill in the future.

**Conclusions:** This type of project shows promising results in educating public librarians about various types of health resources.

**Keywords:** education, outreach, consumer health

## What Each of Us Needs to Know about All of Us: 1 Million+ People and the Precision Medicine Initiative

**Kelli Ham**
Community Engagement Librarian, NNLM Pacific Southwest Region, UCLA Biomedical Library, Newbury Park, California

**Objectives:** This talk will:
- convey the scope and purpose of the National Institutes of Health (NIH) All of Us Research Program to engage one million diverse participants;
- describe the NNLM partnership with All of Us and the goals and strategies to support community engagement through libraries;
- foster ideas for medical librarians to actively contribute in their own institutions and communities.

**Methods:** Setting/Participants/Resources:
The NIH All of Us Research Program has a goal of recruiting one million or more people from all walks of life to take part in a longitudinal study to discover how environment, lifestyle, and genetics can impact health. NIH along with the National Library of Medicine (NLM) and the National Network of Libraries of Medicine (NNLM) will leverage the NNLM network to cultivate partnerships and meaningful collaborations with libraries and community organizations to support participants in the program. The NIH/NLM/NNLM partnership began in September of 2017. The current status of the project and next steps will be described.

**Keywords:** precision medicine, personalized, genomics, genetics, longitudinal, diversity, health studies, NIH,

## Filmology: Norris Medical Library Medical Movie Nights: Creation of a Library Space for Community Outreach and Discussion

**Karin Saric**
Information Services Librarian, Norris Medical Library / Research & Instruction Services, Los Angeles, California

**Nancy Olmos**
Head, Metadata and Content Management, Norris Medical Library, Los Angeles, California

**Objectives:** To provide an overview on the coordination of a film screening, pre-film events, and post-film discussion for a health sciences campus. Authors will discuss how a film screening can be used as a tool to promote the flow of informal information and connect community members working on similar topics so that they can better align and coordinate their work efforts.

**Methods:** In 2014 Norris Library initiated Filmology - NML Medical Movie Nights to bring social context to the academic environment and promote multidisciplinary discussion of healthcare topics across the University of Southern California Health Sciences Campus. The event features a blockbuster film or documentary on an underserved topic. We offer food and host pre-film events and a post-film discussion featuring an interdisciplinary panel of faculty, students, and/or community voices. We have screened six films, and have successfully connected faculty, staff, students, and members of the community. This paper discusses the logistical coordination of the events, reflecting on the evolution from the first event to the streamlined model that is now in place. Content to be discussed includes: film selection, licensing, venue/panel selection, inter-departmental staffing, publicity, food-truck, technology, live stream to second venue, resource guide, event

survey results, and growth & sustainability.

**Results:** We have successfully screened six films, and have connected faculty, staff, students, and members of the larger community. Based on survey and verbal feedback, these events have transformed our campus by allowing for multidisciplinary discussion around underserved health/community topics i.e. homelessness, mental health, intersex, etc. Event participants have shared with us that they have become greatly connected with other community members and are using/sharing our online resource guides. Students have also reached out to us in order to invite our panelists to future events that they have hosted.

**Conclusions:** Libraries used to serve as a meeting place for interdisciplinary conversation amongst health professionals. Although electronic access has altered the demographics of foot traffic, libraries can still make use of other channels within the information infrastructure to foster community conversations. Movie nights have demonstrated themselves to be well received programming that helps connect the community and fosters the flow of informal information across the university campus. Most importantly, due to the library's autonomy to provide a neutral, censorship free environment, we have provided our community with the opportunity to ask the "hard to ask" questions surrounding sensitive/underserved topics.

**Keywords:** movie night, film screening, community engagement, informal information, discussions

## Providing Health Information to Patients: Development and Implementation of a Consumer Health Workshop for Librarians

**Dana L. Ladd**
Community Health Education Center Librarian, Tompkins-McCaw Library for the Health Sciences, Richmond, Virginia

**Emily J. Hurst, AHIP**
Deputy Director, Tompkins-McCaw Library for the Health Sciences, Richmond, Virginia

**Objectives:** Librarians in all settings must often assess patron health information needs and have confidence to direct patrons to reliable sources. Two medical librarians with extensive consumer health experience developed classes designed to increase consumer health knowledge for librarians. Project goals were to enhance consumer health provision at libraries and to promote a sense of community among area health information providers.

**Methods:** The librarians were awarded a Health Information Outreach Award from the National Network of Libraries of Medicine, Southeastern/Atlantic Region to develop and hold a two-day workshop aimed at local public, health sciences, and hospital librarians. Grant funding provided resources to develop the workshop and supported participant travel and overnight lodging. The first day session focused on mobile technology, specifically finding reliable health and wellness apps. The second day contained multiple sessions focusing on essential skills needed to provide consumer health information: reference interview techniques, ethics, website evaluation, and overviews of reliable websites for finding consumer health information. The sessions were interspersed with library tours, interactive activities, and practice questions. Attendees received up to ten hours of continuing education credit from the Medical Library Association.

**Results:** Seventeen librarians attended the sessions representing eleven public libraries, four health sciences libraries, one community college, and one other library type. The workshop received excellent feedback from attendees with 55% indicating that the apps class met most expectations and 45% reported that the class exceeded expectations. The second day 11% of consumer health provision class attendees reported the class meeting some expectations, 11% meeting most expectations, and 78% exceeding expectations. For both the health apps and providing consumer health information sessions attendees reported an increase in knowledge in the subject areas.

**Conclusions:** Providing patients with reliable health information is important. This project sought to provide area librarians the essential skills to ascertain information needs and fill those needs with credible information written in language patients can understand. Overall the workshop was successful with a large number of attendees from around the local area. The classes met or exceeded expectations and attendees reported an increase in expertise level. In addition, the workshop allowed us to promote our library services as a resource for area librarians for further information and assistance and promoted a sense of collaboration among area librarians.

**Keywords:** consumer health, outreach, public libraries, consumer health training

## On the Proliferation of Medical Mis/Dis-information Regarding the Etiology and Manifestation of Autism Spectrum Disorders

**Brady Lund**
Student/Library Employee, Emporia State University - School of Library and Information Management, Osage City, Kansas

**Objectives:** Based on the findings from the Chitika Advertising Network (2013) that 97.8% of searchers using online search engines will select one of the top 12 items, what can viewpoints expressed in these items tell us about what kinds of misinformation and disinformation is being consumed by individuals who use these platforms?

**Methods:** A list of questions regarding various aspects of Autism Spectrum Disorders was created. A content analysis, adapted from Dennis, Carspecken, and Carspecken (2013) and Kupferberg and Protus (2011) was used to identify whether the viewpoints expressed in the search items aligned with a medical consensus or did not. Then the top 12 returned items for each of five platforms - Google, Bing, Google Scholar, Facebook, and Twitter - were rated and a "percent accurate" was found. For example, if 10 of 12 items aligned with the medical consensus, then the platform was deemed to be 83% accurate at responding to that question on that particular occasion. This information was compiled into a list which displays the accuracy of each platform for each question, as well as total accuracy.

**Results:** Among all platforms, Google Scholar was the most accurate, with most of its top two items being consistent with the medical consensus. Beyond Google Scholar, the probability of selecting a reliable information source is 51%,
while the probability of selecting reliable information from the top two items is 58%. This means that there is a less than two-thirds chance of an individual selecting a reliable information source, and less than 34% chance of selecting a reliable information source in both of two consecutive searches.

**Conclusions:** Mainstream search engines and social media websites were generally poor at identifying an accurate response to an autism-related query. With less than two-thirds of items returned from a given search matching the professional consensus, it is statistically unlikely that an individual searching for multiple questions would receive accurate information for all. These results emphasize the need for informed medical professionals and knowledgeable information professionals to help families retrieve accurate information about the challenges they face when raising a child with autism.

**Keywords:** Searching, Autism, Vaccines, Symptomology, Treatments, Social Media, Search Engines

Tuesday, May 22, 2018, 3:00 PM – 4:25 PM
Room: Embassy F (International Tower, Exhibit Level)

---

# Special Content Session: Transforming Perceptions and Adapting Health Library Services for Incarcerated Americans

**Moderator: Becky Baltich Nelson**

## Transforming Perceptions and Adapting Health Library Services for Incarcerated Americans

**Henrie M. Treadwell**
Research Professor, Morehouse School of Medicine, atlanta, Georgia

**Cheryl Franklin**
Medical Director, Morehouse Healthcare, Atlanta, Georgia

**Sara Totonchi**
Executive Director, Southern Center for Human Rights, Atlanta, Georgia

**Paulette Tompkins**
Director of Library Services, Georgia Department of Corrections, Forsyth, Georgia

**Program Description:** Incarcerated people don't always have access to appropriate health care or to the health information that they need. Often, information that is appropriate for prisoners may not be available to them through "normal" channels because of the unique, restrictive environment imposed by the prison system.

According to a recent publication from the Bureau of Justice Statistics, there are approximately 2.2 million people incarcerated in the United States, and the state of Georgia is ranked fifth in its per capita incarceration rate. In addition to a lack of health information access, people who are incarcerated encounter a number of health disparities including those that are unique to their particular circumstances, such as an HIV infection rate that is five times greater than the general population.

Through an informative panel discussion with educators who teach best practices for health care in underserved communities, individuals with a strong interest in criminal justice issues, and government employees with experience in providing health services in prisons, this session will shed a light on this vulnerable population's health and how we, as health librarians, can help to improve their health outcomes and their ability to participate fully in healthcare choices.

**Keywords:** prison reform, incarceration, health disparities, social justice

Tuesday, May 22, 2018, 3:00 PM – 4:25 PM
Room: Embassy D (International Tower, Exhibit Level)

---

# Special Content Session: Transforming Libraries through Diversity and Inclusion: Leading the Way

**Moderator: Hannah Rutledge, AHIP**

## Transforming Libraries through Diversity and Inclusion: Leading the Way

**Shannon D. Jones, AHIP**
Director of Libraries, Medical University of South Carolina, Charleston, South Carolina

**Cynthia L. Henderson, AHIP**
Executive Director, Howard University, Washington

**Brenda M. Linares, AHIP**
Health Sciences Librarian, School of Nursing, KU Medical Center, Olathe, Kansas

**Stephen Kiyoi, AHIP**
Library Director, UCSF Library at Zuckerberg San Francisco General, San Francisco, California

**Program Description:** The words “diversity” and “inclusion” are now popular buzzwords in many workplaces, including universities and hospitals. However, many health sciences librarians may not be aware of what exactly these concepts mean or how they can be incorporated into basic library policy, procedure, and employment practice. Sometimes even people selected to serve on workplace diversity committees do not have a clear understanding of what it means, what it encompasses, or how it can best be implemented in their environments.

This panel discussion will feature four library leaders--three from university health science libraries and one from a hospital library. They will discuss how diversity initiatives have been implemented in their own libraries and within their larger institutions. By sharing their experiences and advice, it is hoped that attendees will gain an appreciation of diversity and inclusion, be inspired to join existing diversity efforts within their own institutions or perhaps even start their own diversity project.

At the conclusion of the panel discussion, facilitators will announce the creation of a book/journal club on diversity and inclusion. Interested individuals will be encouraged to sign up on a form provided. This reading club will be held online once a quarter, with an informal meeting at the MLA 2019 Conference.

**Keywords:** Diversity, inclusion, leadership, workplace issues
