# Supplementary material for: 118th Annual Meeting, Medical Library Association, Inc., Atlanta, GA, May 18–23, 2018
Source: J Med Libr Assoc. 2019 Apr 1;107(2):E1–E20. doi: 10.5195/jmla.2019.672 (PMC6466505; doi:10.5195/jmla.2019.672)
Supplement: Appendix [file jmla-107-E1-s002.pdf]

Medical Library Association

MLA '18 Poster Abstracts

MLA'18
Adapting | Transforming | Leading
May 18–23 Atlanta, GA
www.mlanet.org | #mlanet18

Abstracts for the poster sessions are reviewed by members of the Medical Library Association National Program Committee (NPC), and designated NPC members make the final selection of posters to be presented at the annual meeting.

Poster Number: 1

Time: Tuesday, May 22, 1:00 PM – 1:55 PM

Bringing Each Other into the FOLD: Shared Experiences in Start-up Osteopathic Medical School Libraries

Darell Schmick, AHIP, Director of Library Services, University of the Incarnate Word, School of Osteopathic Medicine Library, San Antonio, TX; **Elizabeth Wright**, Director of Library Services, Arkansas College of Osteopathic Medicine, Arkansas Colleges of Health Education, Library, Fort Smith, AR; **Erin Palazzolo**, Library Director and Professor of Medical Informatics, Burrell College of Osteopathic Medicine at New Mexico State University, BCOM Library, Las Cruces, NM; **Norice Lee**, Assoc. Library Director & Assoc. Prof. / Medical Informatics, Burrell College of Osteopathic Medicine, Burrell College of Osteopathic Medicine Health Sciences Library, Las Cruces, NM; **Molly Montgomery**, Director of Library Services, Proposed Idaho College of Osteopathic Medicine, Library, Meridian, ID; **Anna Yang, AHIP**, Health Sciences Librarian, California Health Sciences University, Library, Clovis, CA

Objectives: To establish a communication channel for founding library administrators of new medical schools.

Methods: Library directors in founding osteopathic medical schools are faced with a unique set of challenges in this role. Depending on the establishing medical school's structure, these can be librarians in a solo capacity. Librarians in this role share experiences and best practices over a monthly meeting for their inaugural and second academic school years, respectively.

Results: Meetings enjoyed robust discussion and comparison of resources. Directors met through the Zoom teleconferencing tool. Meetings were set up to last one hour, though frequently lasted longer and invited discussions outside of the monthly meetings. Best practices were discussed to most effectively serve the schools of osteopathic medicine. Already, FOLD meetings have directly influenced an increase of library staff or increase of specific budget items.

Conclusions: The meetings became a valuable resource for the directors of new and upcoming libraries for schools of osteopathic medicine. As directors seek to establish a shared standard in what can be a traditionally lonely role, these meetings have served to correct this issue through an effective communication medium among colleagues in similar roles.

Keywords: Standards, Osteopathic Medicine, Leadership, New Medical Schools

Poster Number: 2

Time: Tuesday, May 22, 2:00 PM – 2:55 PM

Designing the Future: Librarians as Curricula Developers and Faculty

Alexandra Gomes, AHIP, Associate Director for Education, Information, and Technology Services; **Tom Harrod**, Librarian; **Laura Abate**, Electronic Resources and Instructional Librarian; Himmelfarb Health Sciences Library, Himmelfarb Health Sciences Library, Washington, DC

Objectives: To describe the involvement of three librarians in conceptualizing, creating and teaching 3-credit electives in the School of Medicine and Health Sciences.

Methods: Seeking new ways to integrate informatics instruction in the last two years of medical school, the librarians were invited to submit a course elective proposal. Accepting this new challenge, the librarians wrote and presented a proposal for an Introduction to Systematic Reviews three-credit course to the Curriculum Committees, developed the syllabus and grading rubric, wrote lessons plans and assignments, and taught the class in Spring and Summer 2017. The class is scheduled to be taught again in Spring and Summer 2018.

Results: The systematic review course was well received by the enrolled students. The experience of developing and teaching the course expanded the knowledge and skills of the involved librarians in curricular planning and administration, as well as in the specific subject content of the course. The success of the first elective led to a second proposal, a three-credit elective on the use of media to promote health information which draws on expertise of library staff and faculty from across campus. It was approved by the Curriculum Committees and will be taught in Spring 2018.

Conclusions: Librarians are ideally positioned to take on the creation of electives. In addition to topic expertise, librarians are organized and can meet deadlines, can tap a network of faculty relationships for guest lectures, and can easily handle the administrative and teaching responsibilities associated with being a course director and instructor. In addition, taking on the role of curricula development strengthens ties with the School of Medicine's faculty and administration, and solidifies the library's role in the formal curriculum.

Keywords: curriculum development, instruction, systematic review, elective

Poster Number: 3

Time: Sunday, May 20, 2:00 PM – 2:55 PM

Collaborating to Sustain a Core Collection of Online Resources

Nancy A. Clark, Director, Library Network Office, Library Network Office, Veterans Health Administration, Dallas, TX

Objectives: In 2010, the Library Network Office began to develop a collection of online resources that would be available 24 hours a day to all VA staff. The decision was made in 2015 to establish an agency level multidisciplinary group tasked with reviewing and prioritizing the current resources while also taking into consideration requests for additional high value resources.

Methods: Key stakeholders were asked to nominate individuals to serve on the group to not only represent their program area, but who would be able to place the needs of the agency first. Each year the group is provided with foundational information and relevant metrics about the resources.
In the initial year the group established evaluation criteria to provide an informed and subjective framework for the members to review and prioritize the current core collection. The criteria were then weighted to distinguish the level of importance of each. Members then scored the individual resources per the criteria. The consolidated ranking was then reviewed by the membership, adjustments were made based on discussion, and final recommendations were presented.
Each subsequent year, the methodology is reviewed and adjusted based upon lessons learned, input from the group, available funding and any other changes.

Results: Each year the multidisciplinary group presents their recommendations for resources to renew or to discontinue. Ultimate purchase decisions lay with the Library Network Office. During the initial year, the group developed several overarching conclusions:
• The core collection is a highly valuable resource that is essential to performing the agency mission
• Additional funding from program offices and/or other sources needs to be provided to fully fund the core collection
• To maintain effective fiscal stewardship and relevancy of content, an ongoing advisory group should be established.

Conclusions: This review process provides a mechanism when the budget allocated does not allow funding to renew all current resources or allows for additional resources. Input from this multidisciplinary group has increased ownership of the resources, expanded awareness of the resources, and provided funding support beyond that of the library.
As recommended the initial year, the Library Network Office will continue to use this multidisciplinary advisory group to conduct yearly reviews of all core collection resources.

Keywords: Online resources, collaboration, collection development, selection criteria, budget/funding

Poster Number: 4

Time: Monday, May 21, 2:30 PM – 3:25 PM

Transformative and Translational: Supporting a New PhD program

Alexandra Gomes, AHIP, Associate Director for Education, Information, and Technology Services; **Tom Harrod**, Librarian; **Anne Linton, AHIP**, Director; Himmelfarb Health Sciences Library, Himmelfarb Health Sciences Library, Washington, DC

Objectives: To describe the participation of three librarians supporting a new distance education Translational Health Sciences PhD program in the first two years of the program.

Methods: The School of Medicine and Health Sciences launched a new Translational Health Sciences PhD program in Fall 2016. Prior to the first year of the program, the librarians were invited to join the faculty at the weekly meetings during the year-long program and course planning process. Three librarians were integrated into the program to support the first cohort of 16 students. During the first semester the librarians provided instruction on searching the literature for articles relevant to each stage of the translational process (T1-T4), advanced PubMed search skills, and developing search strategies and record keeping processes for scoping reviews. All sessions were hands-on and followed by individually-tailored assistance. Librarians also provided in-depth consultations with interested students, focused on their problem of interest.

Results: The librarians' instruction and support was well received by the students and the program faculty. As the second cohort began in Fall 2017, the librarians were again invited to teach and support the new cohort. They were also asked to develop an independent study seminar focused on helping students with chapters 1 and 2 of their dissertations. The librarians developed the syllabus, selected readings, and taught the sessions asynchronously, with occasional WebEx sessions. This endeavor strengthened ties between the library and the program faculty, as well as expanded the librarians' skill sets and roles in the larger university community.

Conclusions: Supporting a new program is both exciting and challenging, as it requires learning content and process. The librarians had to develop subject knowledge in the area of translational research as well as a familiarity with the dissertation writing process if they were going to succeed in this new endeavor. Navigating this new role has been a transformative experience, solidifying the library's role as a partner in curriculum development and delivery within the School of Medicine and Health Sciences.

Keywords: translational health sciences, PhD program support, librarian roles, curricular development

Poster Number: 5

Time: Tuesday, May 22, 1:00 PM – 1:55 PM

Library Services Fast Track Merger Planning: Resource, Process, and Financial Integration

Linda M. Matula Schwartz, AHIP, Director, Library Services and Patient Education, Lehigh Valley Health Network Library Services, Allentown, PA; **Kristine A. Petre, AHIP**, Senior Medical Librarian, Lehigh Valley Health Network, LVHN Library Services, Allentown, PA

Objectives: To develop processes and tracking tools to manage the integration of three merger sites into a cohesive library service through SWOT analysis, 30-60-90 day needs assessment and implementation plans, three-phase resource integration tracking, policy and procedure standardization and financial planning for budgeting and expenditure allocation.

Methods: Developing a cohesive library service across multiple merged hospitals requires massive discovery and planning efforts to ensure centralization, policy/procedure standardization and equal access to resources across all sites despite differences in infrastructure.

Merging three additional sites into a network required planning including literature search; foundational SWOT analysis; a three-phase resource integration plan; site-specific webpages; budgeting, expense allocations, and tracking templates. Tracking templates gathered needs assessment information, IT infrastructure, and service needs to ensure critical resource access and post-rollout awareness efforts.

Three-phase resource expansion began with meeting immediate needs, grandfathering sites into some existing licenses, and negotiating renewal contracts over an 18-month period.

Innovative solutions found to cope with a 50% mid-merger staff reduction and remote staff included a “virtual” teleconferenced librarian to meet reference needs, webinar staff meetings, and secure remote desktop. Chat and robots were also investigated.

Conclusions: Integration of library services across merged sites requires massive discovery of everything from IS infrastructure to intranet access to interfacing with EMRs. Having templates to both collect and compare capabilities are crucial to successful efforts, as are a clear mandate from senior management and development of stakeholder relationships. Templates will be shared with requestors.

Keywords: merger mergers integration implementation needs assessment standardization planning services policies

Poster Number: 6

Time: Tuesday, May 22, 2:00 PM – 2:55 PM

Applying Organizational Behavior Principles to Library Management: A Review

Emily J. Hurst, AHIP, Deputy Director, VCU Libraries, Tompkins-McCaw Library for the Health Sciences, Richmond, VA; **Stephanie J. Schulte**, Head, Research and Education Services, Health Sciences Library, The Ohio State University, Columbus, OH

Objectives: Library management literature often consists of descriptive case studies of changes to specific services. Literature covering common library management issues of motivation, decision making, and hiring, are harder to find, even though these areas are developed in organizational behavior research and business management coursework. This review seeks to explore and document the use of organizational behavior in library management literature.

Methods: This review will utilize a scoping review methodology using the Arskey and O'Malley framework, searching the following databases: Medline, CINAHL, Library Literature and Information Science, Library, Information Science and Technology Abstracts, Business Source Premier, and Scopus. A search of book literature will also supplement this review. Criteria for inclusion will be any study methodology including descriptive studies or case reports that discuss aspects of motivation, decision making, or hiring in library management. Systematic review software will be used for tracking and screening results.

Results: Final results will document how organizational behavior research has been used in library management literature and highlight areas for future research. Studies will be sorted by the three areas of motivation, decision making, and hiring, and also by study design. Organizational behavior research methodologies will be used for leveling of the evidence found in this review.

Keywords: library management, organizational behavior, motivation, decision making, hiring

Poster Number: 7

Time: Sunday, May 20, 2:00 PM – 2:55 PM

Collaborating to Transform an Introduction to Drug Information Course: One Librarian's Experience

Emily P. Jones, Research and Education Informationist, Medical University of South Carolina Libraies, Charleston, SC

Objectives: In the first four months of beginning a new librarian position, a recent MLIS graduate collaborated with a College of Pharmacy faculty member to revise curriculum and implement flipped instruction in the first year's Drug Information course.

Methods: The librarian developed curriculum-integrated instruction in the flipped classroom environment for four classes of the semester-long course. Over a four-week period, the librarian created pre-class videos, taught a live lecture incorporating active learning, and developed pre- and post-class quizzes. Course content will focus on the intersection of drug information and library resources.

Results: Student perceptions of the librarian were collected through an end-of-course survey distributed through E*value. Students were asked to rate the librarian on various traits like availability, preparedness, and effectiveness. Students were also asked open-ended questions about strengths and areas of improvement of the librarian's teaching. The librarian applied themes to these responses based on the student's answers.

Conclusions: The librarian gained invaluable experience and insight into the information needs of pharmacy students. This experience also allowed the librarian to establish a consistent presence with students early in the curriculum, hopefully encouraging them continue to reach out to the librarian for assistance. Student perceptions of teaching effectiveness and areas of strength/improvement will be used to inform the librarian's teaching in future iterations of the course. New videos and revised handouts will be created Spring-Summer 2018 in preparation for the course in Fall 2018.

Keywords: drug information, curriculum, flipped instruction, collaboration

Poster Number: 8

Time: Monday, May 21, 2:30 PM – 3:25 PM

Transforming Teamwork: Training Biomedical Researchers to Effectively Collaborate with Data Scientists

Alisa Surkis, Assistant Director for Research Data and Metrics, NYU Health Sciences Library, NYU Health Sciences Library, New York, NY; **Aisha Langford**, Assistant Professor, NYU School of Medicine, Department of Population Health, New York, NY; **Yindalon Aphinyanaphongs**, Assistant Professor, School of Medicine, Department of Population Health, New York, NY; **Nicole Contaxis**, Data Catalog Coordinator, NYU School of Medicine, NYU Health Sciences Library, New York, NY

Objectives: Many biomedical researchers lack awareness about opportunities afforded by data science and so they miss collaboration opportunities. Differences in perspectives and language between researchers and data scientists can result in failed collaborations. Librarians with experience working with researchers and data scientists sought to bridge that gap by developing a class to explain data science practices, opportunities, and limitations to researchers.

Methods: Through discussions with a population health researcher and a data scientist who had previously collaborated, as well as with other data scientists and biomedical researchers at an academic medical center, the challenges and opportunities of collaborations between biomedical researchers and data scientists were elucidated. Discussions informed the appropriate level of detail for a data science class aimed at biomedical researchers, types of case studies to discuss, and issues to highlight regarding negotiations between a domain expert and data scientist. Other curricular resources (e.g. online data science courses) also informed the development of a two-hour, case study-based class that included a hands-on exercise of filling out a detailed form to propose capstone projects for data science masters students.Class evaluation forms assessed students perceptions of course effectiveness, level, and length, comfort level with the material, and degree to which students felt more prepared to collaborate with a data scientist.

Results: The class was offered twice, to a total of 40 attendees, from a range of disciplines. In the evaluations, the majority of students reported feeling more comfortable with data science concepts and more prepared to pursue a collaboration with a data scientist (31% much more comfortable, 46% somewhat more comfortable, 11% a little bit more comfortable). The majority also reported feeling more prepared to pursue a collaboration with a data scientist following the class (37% definitely more comfortable, 49% probably more comfortable).

Conclusions: Data science offers immense opportunities to biomedical researchers, but differences in how data scientists and biomedical researchers formulate questions, understand their answers, design their experiments, implement their discoveries, and weigh their priorities offer significant challenges to pursuing those opportunities. By teaching biomedical researchers about the tools, techniques, and limitations of data science, we can work towards overcoming these challenges and establishing a robust collaborative environment. As a first step towards a more collaborative biomedical data science environment, the class is a low-cost, low-barrier-to-entry way to investigate the needs of the research community and begin building bridges across the institution.

Keywords:
data science
team science
data education
collaboration

Poster Number: 9

Time: Tuesday, May 22, 1:00 PM – 1:55 PM

Current Practices of Academic Health Sciences Libraries Serving New Medical Schools: A Mixed Methods Study

Joanne M. Muellenbach, AHIP, Director, Jay Sexter Library, Touro University Nevada, Henderson, NV

Objectives: To conduct a systematic assessment of academic health sciences libraries serving new, 21st century medical schools. To provide a description of core library resources and services, detect trends through comparisons across allopathic and osteopathic medical schools, justify the need for additional resources and services, and assist leadership within developing medical schools in planning for their future health sciences libraries.

Methods: Mixed methods study pursued through three phases. The first phase involved the identification of allopathic and osteopathic medical schools in the United States and Canada that were accredited since 2000, and the contact information for the library directors or leaders serving them, via publicly-facing websites. An assessment tool, developed by the Association of Academic Health Sciences Libraries (AAHSL) for creating peer groups, was used to compare the AAHSL-member libraries who have completed AAHSL surveys, in the areas of budget, collections, instruction, and personnel. Phase two will consist of a survey directed at the library leaders serving the new, 21st century medical schools. Additional questions to be answered by library leaders through in-depth interviews will comprise the third phase of the study.

Conclusions: It is hoped that this study would offer a systematic comparison of academic health sciences libraries serving new, 21st century medical schools in the U.S. and Canada. Library leaders, not only those affiliated with new medical schools, but also those affiliated with long-standing, and more traditional schools, would use this data to make a case for new resources, services, or staff. In addition, medical school administrators would find opportunities in this study’s results for more productive collaborations with the library, in areas such as curriculum-integrated instruction. Finally, leaders within developing schools would become better informed about current library services being provided, in order to plan for an appropriate budget and staffing for their future health sciences libraries.

Keywords: academic health sciences libraries; assessment; cross-sectional study.

Poster Number: 10

Time: Tuesday, May 22, 2:00 PM – 2:55 PM

Transforming Library Education Opportunities to Reach New Audiences on Third-Party Platforms

Derek Johnson, Health Professionals Outreach Specialist, National Network of Libraries of Medicine Greater Midwest Region, Iowa City, IA

Objectives: To demonstrate how a librarian was able to reach a broad set of public health professionals through the creation and promotion of an asynchronous, online learning module on national and regional public health learning platforms.

Methods: In an effort to broaden engagement and outreach with public health professionals, a unique on-campus partnership was formed between a librarian and a regional public health training institute to develop an asynchronous, online learning module. After initial kick-off and brainstorming sessions, the collaboration consisted mostly of interactions between the librarian and the public health training institute's instructional designer. This poster describes the planning process, delegation of tasks, and challenges encountered.

Results: Through multiple meetings, online exchanges, and content development sessions, a 2-hour course was developed using Articulate Storyline software and promoted online via the Public Health Foundation's national TRAIN learning network and a regional public health learning management system.

Conclusions: By forming partnerships and engaging with non-library learning management platforms, health sciences librarians can expand their training outreach to broader audiences.

Keywords: Online Learning
Partnerships
Content Development

Poster Number: 11

Time: Sunday, May 20, 2:00 PM – 2:55 PM

Communication, Collaboration, Consistency, and Campus Culture: How Librarians and Nurses Worked Together to Bridge the Information Literacy Gap

Amy Knehans, AHIP, Clinical Outreach, Instruction & Liaiosn Librarian, Penn State Univeristy College of Medicine, Harrell Health Sciences Library Research & Learning Commons, Hershey, PA; **Christina L. Wissinger**, Health Sciences Librarian, Penn State, Life Sciences Library, University Park, PA; **Valerie Lynn**, Head Librarian, Hazleton Campus Library, Hazleton Campus Library, Penn State Hazleton, Hazleton, PA; **Nancy E. Adams**, Director of Foundational Medical Sciences, George T. Harrell Health Sciences Library, Penn State College of Medicine, Hershey, PA;

Objectives: A large academic institution liaison librarians are scattered throughout a region. They strive to balance providing resources to their campuses while maintaining consistency in information instruction across the institution. To address these challenges, we created a one-day symposium to bring nursing liaisons across 13 campuses together. This event enhanced communication, collaborative decision-making and exposed the librarians to differences between campuses.

Methods: A planning committee of 4 librarians from 3 different campus locations organized the event. Feedback from the 13 nursing liaisons helped inform content for the symposium. After contacting each nursing liaison by phone to discuss session topics the following objectives were created: increase collaboration and partnerships among nursing librarians, obtain knowledge about resources and tools for evidence-based nursing practice, identify how working nurses' information seeking skills differ from the nursing students, and learn how EBP is applied in a nurse's work routine. The planning committee also sought to identify strategies to improve knowledge-based information services for nurses and nursing students across the 13 campus locations. As part of the event nurses from a variety of backgrounds were invited to share information about how they used information as they moved through their education and careers.

Results: During the event, ideas from small group and large group discussions were recorded on whiteboards throughout the room. The day after the event a 6 question survey was sent to the 13 liaison librarians who attended the program. This allowed time for reflection and more thoughtful comments. The survey questions matched the event objectives and included questions about the usefulness of the day and interest in offering the symposium as an annual event.

Conclusions: The event began with a panel discussion from a bedside nurse, nurse researchers, and nurse educators who work in different positions and provide unique perspectives. The second part of the day consisted of small group discussions and ended with a large group discussion. Each discussion helped shape and develop goals to improve library instruction for nursing students and increase meaningful collaboration between librarians and nursing faculty. Librarians valued hearing how nurses find and use information and how their librarian colleagues are supporting nursing courses. Ultimately, all attendees were able to establish new relationships that will enhance librarian nursing instructor collaboration.

Keywords: Nursing, Liaison, Collaboration, Information Literacy

Poster Number: 12

Time: Monday, May 21, 2:30 PM – 3:25 PM

Another Genome Project: Medical Library Services for Genetic Counselors and Genetic Counseling Students

Amy E. Donahue, AHIP, Genetic Counseling Intern, University of Wisconsin-Madison, Master of Genetic Counseling Studies Program, Milwaukee, WI; **Elizabeth Petty**, Senior Associate Dean, Academic Affairs, University of Wisconsin-Madison, School of Medicine & Public Health, Madison, WI; **April Hall**, Genetic Counselor, University of Wisconsin-Madison Waisman Center, Medical Genetics Clinic, Madison, WI

Objectives: To determine services and resources medical libraries currently provide to genetic counselors and genetic counseling students. The goal of this research is to explore how medical libraries meet the information needs of the genetic counseling profession and to begin to elucidate any gaps between needs and existing resources in the era of clinical genomic medicine and precision medicine.

Methods: Representatives of medical libraries were invited in fall 2017 via MLA & National Network of Libraries of Medicine listservs to take an online survey developed by the authors. The IRB exempt Qualtrics survey collected library demographic information (including region, setting, staffing size, etc.), qualitative and quantitative information about services provided to genetic counselors and/or genetic counseling students, and whether specific resources are made available through the library.

Results: There were 27 responses, representing libraries across the United States and beyond in academic medical centers, teaching hospitals, multi-hospital systems, and additional specialized academic and medical settings. Fifteen respondents indicated their setting employed genetic counselors; 7 indicated that their setting is affiliated with a genetic counseling (GC) training program (of these, three indicated both). Eight respondents, with either no or unknown GC program affiliation, had no employed genetic counselors. Of the 19 responses from settings employing genetic counselors and/or affiliated with a GC program, 9 respondents said that they do not provide genetic counseling-specific library services. When services were provided (n=10), the most common was literature searching (n=8), followed by specific resource purchases/subscriptions (n=7). Four respondents indicated a dedicated genetic counseling liaison librarian, and three had developed a genetic counseling subject guide or other resource list. While over 80% indicated providing access to both genetics resources such as ClinVar and Genetics Home Reference and general medical resources such as PubMed, UpToDate, and medical journals (e.g. NEJM), less than half of all 27 respondents indicated they provide access to the American Journal of Medical Genetics, the Journal of Genetic Counseling, or the books *Desk Reference: Clinical Genetics & Genomics* and *A Guide to Genetic Counseling*.

Conclusions: Genetic counseling is a specialized and rapidly growing medical profession: there are currently over 4,000 board certified genetic counselors and 39 training programs in the United States. The results of this survey, while representing a small number of medical libraries, demonstrate a substantial opportunity for library and information services targeted to both practicing genetic counselors and genetic counseling students.

Keywords: genetic counseling, genetics, library services, library resources

Poster Number: 13

Time: Tuesday, May 22, 1:00 PM – 1:55 PM

Adaptations in Kinesiology: Collaborations for Global Experiential Learning

Jacqueline Freeman, Informationist, University of Michigan, Taubman Health Sciences Library, Ann Arbor, MI; **Gurpreet K. Rana**, Global Health Coordinator, Taubman Health Sciences Library, Taubman Health Sciences Library, Ann Arbor, MI; **Vanessa Barton**, Global Engagement Coordinator , School of Kinesiology, University of Michigan - Ann Arbor, Ann Arbor, MI

Objectives: Increasingly students are engaged in experiential learning that takes them outside their classroom as universities graduate global citizens. This is occurring in all disciplines, including kinesiology, yet global information needs often get overlooked. This poster describes the collaboration to address these information gaps and build awareness of global information resources for kinesiology.

Methods: Health sciences informationists at the Taubman Health Sciences Library met with the Kinesiology Global Programs Coordinator in summer 2017 to discover the mission, scope, and dimensions of global engagement in kinesiology. Using successful global health interventions in other health sciences schools as a framework, informationists began to assess information needs, including faculty interest in research, and to identify opportunities for integration into student learning. Information resources for pre-departure orientation were developed as an initial step. Assessment of pre-departure training and development of future activities will be carried out.

Keywords: kinesiology, global, experiential learning

Poster Number: 14

Time: Tuesday, May 22, 2:00 PM – 2:55 PM

Library Space Assessment Using an Open-Source Toolkit: Pitfalls and Promises

Laura Menard, Assistant Director for Medical Education & Access Services, Indiana University School of Medicine, United States, IN

Objectives: The science library at a small university with a robust health sciences program is preparing to undergo a total renovation in 2018. The library redesign planning committee was interested in using qualitative data to inform their design plans, and gate counts were not granular enough. Enter SUMA, a mobile-based open-source toolkit for collecting customizable data about library space usage.

Methods: The planning committee reviewed SUMA for suitability and found that it met our needs. We then built and tested an instance of SUMA that would gather data about users' preferences when it came to interacting with the library space: what resources they were using in-house, what tech they were bringing with them or checking out, how they were interacting with each other, and what activities were going on in the library besides quiet study. We set up workflows and a training so that student workers at the library could use SUMA to carry out regular counts at designated times, and ran quality checks on their process. We spent a semester collecting data, and at the end of the project we synthesized and presented our findings to the planning committee to inform the design of the future space.

Results: Over the course of Spring 2017 semester, 44 distinct sets of usage data, comprising 1,790 individual data points, were gathered. Data gathering times were reasonably uniform, and, as quality control checks were run regularly the data was in good shape to be analyzed and to inform some design elements for the new space. These elements include but are not limited to design flow, technology capabilities, security measures, furniture choices, and individual/group study space allocation. Throughout the process we learned several lessons that will be applicable to future space assessment initiatives.

Conclusions: The information gathered will be helpful for the design team as we go forward with future meetings with the architects and facilities managers in charge of design aspects such as furniture and amenities for the new space. Because of observed use patterns, the new space should be very tech-friendly (multiple outlets at each table, strong internet connection, chargers and other accessories available for checkout, printing from laptop assistance, etc) and have a mix of both quiet and collaborative spaces.

Keywords: open source, space assessment, redesign, usage statistics, data

Poster Number: 15

Time: Sunday, May 20, 2:00 PM – 2:55 PM

Content and Design Features of Academic Health Sciences Libraries' Home Pages

Rozalynd P. McConnaughy, AHIP, Assistant Director for Education & Outreach, University of South Carolina School of Medicine Library, Columbia, SC; **Steve Wilson, AHIP**, Web and Outreach Librarian, University of South Carolina, School of Medicine Library/Center for Disability Resources Library, Columbia, SC; **Ruth Riley, AHIP**, Director, Library Services, School of Medicine Library, Columbia, SC

Objectives: The goal of this study was to perform a content analysis of academic health sciences libraries' home pages in order to determine what features and design elements are most commonly being used. The results of the study will provide a profile of best practices for libraries engaged in web site redesign projects.

Methods: Subjects: 135 academic health sciences library home pages Methods: Using a checklist of eighteen questions, the project team collected data from each academic health sciences library's home pages. Information obtained about the home pages included commonly used resources in search boxes, common topics used in consistent navigation bars, the availability of social media, common content categories used on the body of the home page, and more. The data was compiled in an Excel spreadsheet for analysis.

Results: The following components were present in fifty percent or more of academic health sciences libraries' home pages: a contact phone number, a contact email address, an "Ask a Librarian" feature, the physical address listed, a feedback/suggestions link, a chat option, subject guides, a discovery tool or database-specific search box, multimedia, social media, a site search option, a responsive web design, and a copyright year or update date. The most commonly used resources within a database-specific search box included journals, library catalog, PubMed, and books/ebooks. Facebook was the most common type of social media maintained by an academic health sciences library.

Conclusions: About, services, resources, research, and help were the most common labels used in navigation bars, as well as listing specific databases in the navigation bar. The majority of the academic health sciences libraries have the library name and logo top-left aligned on the page, include a standalone site search box in the upper right header of the pace, place a consistent navigation bar near the top of the page, use a tabbed design style for their discovery services or database-specific search box, and include the library phone number in the footer.

Keywords: web site design, web site trends, home pages

Poster Number: 16

Time: Monday, May 21, 2:30 PM – 3:25 PM

Teaching Graphic Medicine to Undergraduate Students

Ariel Pomputius, Health Sciences Liaison Librarian; **Nina Stoyan-Rosenzweig**, Senior Associate in Libraries; Health Science Center Libraries, Gainesville, FL

Objectives: Through a call for proposals- most of which are accepted- University of Florida's honors program offers a unique opportunity for faculty members to design and offer 1-credit courses for undergraduate students on various books. For the first time at UF, two health science library faculty offered a semester long course focused on graphic medicine, using "The Graphic Medicine Manifesto."

Methods: Librarians have played a key role in the developing field of graphic medicine, and are involved in creating their own novels and promoting use of the medium in education. The health science center library encouraged librarians to create a course introducing students to both primarily health-related graphic novels and the emerging field of graphic medicine. While classes were largely discussion-based, students were also required to present on self-selected comics in the graphic medicine genre and engage with the medium of comics through participation in creative challenges. At the end of the semester, students must complete a final project on insights gained from the course. Based on their final project proposals, half of the students are creating their own comics and half are doing in-depth research on current and future applications for graphic medicine.

Results: The eight undergraduate students in the class were highly engaged and interested in the topic. While most of the students were interested in pursuing a career in the health sciences, a few joined for an interest in comics or graphic novels. Many of these students spoke from personal experience of issues discussed in graphic medicine novels, and were very passionate about the applications of graphic medicine for highlighting health concerns, and healthcare training experiences.

Conclusions: Graphic medicine is a growing field in the medical humanities. This approach combines verbal and visual narrative and facilitates sharing unique perspectives of the healthcare system. Use of the medium thus can give a platform to marginalized or under-served populations, encourage better physician-patient communication, build empathy between healthcare professionals and their patients, enhance development of reflective practice among students, and express some of the complexities experienced in the medical field. By working with undergraduate health science students, this class encourages future health professionals to look at alternative ways to view their field, develop as healthcare providers, and respect unique perspectives.

Keywords: graphic medicine, graphic novels, teaching, undergraduate students, semester-long course

Poster Number: 17

Time: Tuesday, May 22, 1:00 PM – 1:55 PM

Adapting a Game for Teaching Research Methods

Donna Baluchi, Supervisor - Public Services, University of Utah, Spencer S. Eccles Health Sciences Library, Salt Lake City, UT; **Tallie Casucci**, Assistant Librarian, Spencer S. Eccles Health Sciences Library, J. Willard Marriott Library, Salt Lake City, UT; **Brandon Patterson**, Technology Engagement Librarian, University of Utah, Spencer S. Eccles Health Sciences Library, Salt Lake City, UT; **Erin Wimmer**, Instructional Designer, SoFi, n/a, Cottonwood Heights, UT

Objectives: To modify an entertainment board game into a serious game, which leads participants through the research process in an engaging, inquiry-based way.

Methods: The librarians at University of Tennessee at Chattanooga Library created an open access serious game about the academic research process. The game was based on “The Game of Life,” an entertainment board game. The Game of Research splits the research progression into four components: brainstorming, keywording, searching, and writing. We modified UTC’s version to make it more relevant and challenging for a health sciences campus. For example, the topic cards were created to include health sciences topics and research questions. After testing the game with a small, practice audience, modifications were made to the serious game to better fit the health sciences audience. Additionally, more hands-on practice was built into the game in order for participants to progress. For instance, when a player lands on a "topic bonus," they are required to complete a task before receiving the bonus money.

Results: The game was successfully used in orientations for Nursing, Dentistry, graduate serious games course for Entertainment Arts and Engineering, an undergraduate pre-health professionals class, and a workshop for early-career researchers. By using the Game of Research, questions concerning the research process, such as Boolean, types of scholarly articles, and copyright, emerge more organically.

Conclusions: The Game of Research is a valuable and engaging teaching tool. As a serious educational game, it provides an inquiry-based method for students to learn more about the research process.

Keywords: gamification education teaching research game

Poster Number: 18

Time: Tuesday, May 22, 2:00 PM – 2:55 PM

Disaster Information Needs, Credible Information Access, and Use of Social Media for Crisis Communication: A Survey Study of the Adult General Public in Eleven Counties during Two Disasters

Feili Tu-Keefner, Associate Professor, University of South Carolina, School of Library and Information Science, Columbia, SC; **Jingjing Liu**, Assistant Professor, University of South Carolina, School of Library and Information Science, Columbia, SC; **Darin Freeburg**, Assistant professor, University of South Carolina, School of Library and Information Science, Columbia, SC; **Denise R. Lyons**, Deputy Director, South Carolina State Library, South Carolina State Library, Columbia, SC; **Michael Corbo**, Office and Communication Coordinator, University of South Carolina, School of Library and Information Science, Columbia, SC

Objectives: Catastrophic disasters (e.g., hurricanes) occur in a particular Southern coastal state almost annually. The study examines which disaster information sources the community members used and their evaluation of the information's credibility during two disasters in 2015 and 2016. An investigation of how people shared information with others (for instance, social media such as Facebook) during disasters is also included.

Methods: The methodology is survey-based, with both qualitative and quantitative research used for data analysis. Printed questionnaires were distributed to public libraries and their branches in eleven counties in the state. All of these counties experienced hurricanes or flooding in 2015 and 2016, while one county suffered from flooding in both years. The population is defined as adult community members who are 18 years or older. Information regarding the study was also posted on the public libraries' home pages and social media sites, with links to the digital version of the questionnaires. The participants could choose to fill in the digital survey questionnaire anonymously on the Internet and submitted it online, or filled in the printed survey questionnaire anonymously and used U.S. mail to send it to the research team.

Results: The Internet was widely used during and after the disasters. Of the community members surveyed, the majority felt that it was very easy or easy to find information about the disaster. The South Carolina Department of Health and Environmental Control website was the most frequently used resource. Both CDC.gov and Ready.gov were also mentioned as sources of information. Most of the survey participants did not mention the use of MedlinePlus.gov, the most essential consumer health information source recommended by health sciences librarians. Most concerning was that many of the survey participants said that they did not use any resource to find disaster health information. Among the popular social media platforms, Facebook was the site most commonly used. However, several members also voiced distrust of the information distributed via social media platforms. Conclusions: The findings show a discrepancy between the reliable resources vital to consumers and the health information accessed by the community members. Even though most of the survey participants indicated that it was easy or very easy to find information, some of them did not use any credible information resource at all either during or after the disaster. Social media platforms, such as Facebook, were widely used by the community members to communicate with others.

Poster Number: 19

Time: Sunday, May 20, 2:00 PM – 2:55 PM

Cost, Quality, Access, and Policy: Creating a Portal for Health Services Researchers

Judith E. Smith, Informationist; **Carol Shannon**, Informationist; Taubman Health Sciences Library, Taubman Health Sciences Library, Ann Arbor, MI

Objectives
To meet the needs of health services researchers at the University of Michigan, Ann Arbor, through creating a research guide focused on cost, quality, access, disparities, and policy. In addition to providing streamlined access to needed resources, goals include helping to build partnerships with health services researchers, and serving as a teaching tool for informationists.

Methods
Health Services Research is a highly interdisciplinary field, requiring the examination of a large category of resources to investigate questions and analyze topics to understand what works in healthcare. While health services researchers at the Institute for Healthcare Policy and Innovation have access to an abundance of resources in the library and beyond, access is complicated because resources are indexed in multiple databases and grey literature. To meet this challenge, public health informationists developed an information portal tailored to the needs of health service researchers, organized by topical areas such as cost, quality, access, disparities, and policy. Feedback from health service researchers and staff, as well as other informationists and librarians across campus, informed the process of aggregating information and customizing access to resources to meet researchers' needs in this complex area.

Results
We considered different ways of organizing the guide: 1) more traditionally, dividing by type of resource, or 2) organizing by broad, health services research topics. Driven by our experience with this area of research and the questions we are typically asked by students, faculty, and researchers, we decided to group resources by topic area, so that users who were searching for information on cost or quality, for example, could go directly to the best resources of all kinds on that topic.

Conclusions
This is our first version of a guide to make research on health services research easier to find and use. As we begin to teach from the guide and to use it in consultations, we plan to systematically gather user feedback and adapt and change to make it more useful for our users.

Poster Number: 20

Time: Monday, May 21, 2:30 PM – 3:25 PM

Teaching Evidence-Based Practice: Leading Nurses in Transforming Care

Beverly Murphy, AHIP, FMLA, Assistant Director, Communications and Web Content Management; DUHS Hospital Nursing Liaison; Watts SON Liaison, Medical Center Library & Archives, Duke Medical Center Library & Archives, Durham, NC; Virginia Carden, AHIP, Administrative Research Librarian, Duke University, Medical Center Library & Archives, Durham, NC; Jamie Conklin, Health Sciences Librarian and Liaison to Nursing, Duke University, University of North Carolina at Chapel Hill Health Sciences Library, Chapel Hill, NC; Deborah H. Allen, Director of Nursing Research & EBP, Duke University Health System, Duke University Health System, Durham, NC

Objectives: This poster describes the creation and implementation of a program designed to introduce the foundations of evidence-based practice (EBP) to clinical nurses through intensive workshops incorporating didactic presentations, roundtable discussions, and hands-on searching and writing. This program involves the interprofessional partnership of medical librarians, nursing faculty, and clinical nursing leaders.

Methods: Previous research and EBP workshops, as well as results from a 2016 hospital-wide nursing needs assessment, revealed that nurses desired more hands-on experience and understanding of EBP. The evaluation indicated a need for programming that covers basic to advanced EBP, nursing research outcomes from our institution, and clarity of roles in the research process. To address these essentials, the Nursing Research and EBP Council developed “Mitigating the Madness,” a series of hands-on workshops. “Approaches to Clinical Inquiry” covers idea development, question formulation, differentiation of project type, transformation of the clinical question to a PICO format, and literature searching. “Approaches to Project Development and Implementation” includes literature appraisal, formalization of outcomes and measurements, project design and implementation, regulatory processes, and dissemination. Participants are paired with coaches - a nurse and a librarian - who assist with project formation. “Approaches to Dissemination” focuses on abstract writing, poster formation, presentation skills, and publications.

Results: Thirty nurses, ranging in age, educational degree, years of work experience, and representing a variety of inpatient-outpatient areas, participated in the first workshop. One hundred percent of the attendees indicated that their learning needs were met, and 90% are continuing to work on their projects. This has required some updated literature searching and assistance with summarizing and synthesizing the literature. The second and third workshop in the series, and a repeat of the first workshop, will take place before the MLA Annual Meeting. Results from these workshops can be shared during the poster session.

Conclusions: A series of workshops that pair practicing nurses with both nurse and librarian coaches can lead to an improved understanding of EBP and its translation into practice through real-world projects.

Keywords: Evidence-Based Practice, Nursing, Interprofessional Collaboration

Poster Number: 21

Time: Sunday, May 20, 2:00 PM – 2:55 PM

The Transforming Landscape of Cultural Diversity in the Biomedical Literature

Karen Gutzman, Impact and Evaluation Librarian, Galter Health Sciences Library, Galter Health Sciences Library, Chicago, IL; **Diana Almader-Douglas**, Librarian, Mayo Clinic, Mayo Clinic Arizona/Hospital Library, Phoenix, AZ; **Brenda M. Linares**, AHIP, Health Sciences Librarian, School of Nursing, University of North Carolina, KU Medical Center, Olathe, KS; **Annabelle Nunez**, Associate Director, University of Arizona Libraries, Health sciences Library, Phoenix, AZ; **Bredny Rodriguez**, AHIP, Health Sciences Librarian, Health Sciences Library, Health Sciences Library, Las Vegas, NV

Objectives: Analyzing topical trends in the biomedical literature can help identify influential research, characterize changes in research direction, or discover research areas that have been overlooked. This project analyzes the metadata from papers indexed with the “cultural diversity” MeSH term from 2003-2017, so that we can better understand and discuss the landscape of cultural diversity in the biomedical literature.

Methods: Metadata from papers indexed with the MeSH term “cultural diversity” published between 2003-2017 were extracted from the Scopus database. The title and abstract fields of each paper were analyzed using natural language process to identify terms that occur more than ten times in the entire corpus. These terms were visualized in a term co-occurrence map using VOSViewer (http://www.vosviewer.com/). The analysis involved identifying topical trends over time, topical density, and topical usage and citations based on average year of publication. In a term co-occurence map the circles sizes show the number of times words occur in the title or abstract of the publication. The distance between two terms indicates how often the terms co-occur in a title or abstract; the smaller the distance, the larger the number of co-occurrences and the stronger the terms are related to each other.

Results: Overall, the terms used most often in this dataset are related to education, evaluation, and research.The term most often found in current literature is “health equity”, in papers highlighting the problems of health disparities and the need to better understand social determinants of health. The second most found term is “clinical placement”, in papers reflecting the physical location of students in healthcare related fields and its effect on their learning. The term occurring often in earlier papers is “affirmative action”, used when discussing the need for diversification of education and training programs. The second most occurring term in earlier papers is “clinical encounter”, used when discussing cultural competence of clinicians and other healthcare providers when working with diverse patients.

Conclusions: Cultural diversity literature has evolved over time with language that demonstrates a trend towards increased efforts in diversity and inclusion and cultural responsiveness in health care. These efforts build a health workforce better equipped to improve health disparities. It is important for librarians to stay current in this topic and to guide conversations of inclusivity in research, healthcare, and medical education.

Keywords: Topical analysis, bibliometrics, alternative metrics, medical education

Poster Number: 22

Time: Monday, May 21, 2:30 PM – 3:25 PM

Identifying Emerging Technologies through Text-Mining of PubMed Search Results: New Potential for Extracting Meaning

Patricia F. Anderson, Emerging Technologies Informationist, Taubman Health Sciences Library, Taubman Health Sciences Library, Ann Arbor, MI; **Skye Bickett**, AHIP, Assistant Director, Library, GA-PCOM, Suwanee, GA; Joanne Doucette, Librarian, Library and Learning Resources, Library & Systems, Boston, MA; **Pamela R. Herring, AHIP**, Electronic Resources Librarian, Harriet F. Ginsburg Health Sciences Library, Harriet F. Ginsburg Health Sciences Library, Orlando, FL; **Carol Shannon**, Informationist, Taubman Health Sciences Library, Taubman Health Sciences Library, Ann Arbor, MI; **Lin Wu**, AHIP, Assistant Director/Associate Professor, Research & Learning Services, University of Tennessee Health Science Center, Health Sciences Library, Memphis, TN; **Andrea C. Kepsel**, AHIP, Health Sciences Educational Technology Librarian, Michigan State University Libraries, East Lansing, MI; **Tierney Lyons**

Objectives: The Emerging Technologies Team, part of the Medical Library Association (MLA) systematic review (SR) projects, used text mining to identify patterns, themes, and trends important to the practice of medical librarianship, based on the previously reported PubMed Search filter.

Methods: After reviewing and finalizing the emerging technologies PubMed search strategy created for the project, and exporting the data, a variety of open source or open access text mining and analysis tools were. Voyant was the tool selected to begin the basic analysis, making it possible to identify big concepts from 2016 dataset. OpenRefine was next, useful for expanding the text analysis, and exploring the context surrounding terms identified in the Voyant analysis. AntConc made possible a deep dive into the specific terms/phrases of interest to discover a more precise context and related sub-concepts. Moving forward, it may be possible to expand the number of years analysed by using resources such as Flux to work with larger datasets.

Results: Preliminary analysis shows the concept of “virtual” dominating by a wide margin. As an example of how AntConc and OpenRefine allowed us to be more precise in discovery of specific concepts, “virtual” became virtual reality, virtual worlds, virtual simulation/simulator, and virtual screening. Additional concept and terms clusters, such as “3D,” will be discussed, as well as contexts such as which medical specialties and research areas focus on which emerging technology clusters.

Conclusions: Once a dataset exists, the dataset can itself be useful to identify trends in specific areas, such as surgery or education, potentially in response to areas of interest within the library’s target audience. Text mining may be an additional tool for extracting useful information from the search results of complex searches performed by librarians, such as those for systematic reviews or other related search-intensive methodologies. This may provide an opportunity for positioning the work of librarians in the context of strategic relevance at the organizational level, with especial relevance to forecasting and trendspotting in specific focused topics or research domains.

Keywords: textmining, pubmed, systematic reviews, data, analysis, open source, emerging technologies,

Poster Number: 23

Time: Sunday, May 20, 2:00 PM – 2:55 PM

Covidence vs. Rayyan: A Comparison of Systematic Review Tools

Liz Kellermeyer, Biomedical Research Librarian, National Jewish Health, Library & Knowledge Services, Denver, CO; Ben D. Harnke, Education & Reference Librarian, University of Colorado Anschutz Medical Campus, Education and Reference, Aurora, CO; **Shandra Knight**, Director, National Jewish Health, Library & Knowledge Services, Denver, CO

Objectives: To compare and evaluate the features and performance of a subscription-based systematic review tool, Covidence, against a free competitor, Rayyan.

Methods: Health Science Librarians from two institutions conducted a mock review on a predetermined article set using Covidence and Rayyan. An assessment of the tools' abilities, strengths, and limitations were made by examining how each handles key functionalities, including importing and exporting citations and PDFs, identifying and removing duplicates, blinding reviewers, highlighting keywords and inclusion/exclusion criteria, assigning reviewers and workflows, recording reviewer audit trails, and final quantitative/qualitative data extraction.

Results: Covidence mirrors the multi-phase review process, including data extraction, directly in its design. Rayyan, on the other hand, is really only designed to aid with the reference screening phases. It takes a minimalist approach, placing more of the logistical/workflow burden on the users themselves. Many of the peripheral features (e.g., highlighting, tagging, etc.) are comparable.

Conclusions: Covidence works well and is worth its cost for more rigorous Cochrane-type reviews where methodology must be adhered to and documented at each stage. In spite of some limited functionality, Rayyan is a good free alternative for article screening, and is well suited for background/scoping reviews that do not require the rigor of a full systematic review. Because all review instances are free in Rayyan, it could also be a good choice for practice guidelines or other reviews that have multiple PICO questions. Finally, for those using EndNote for initial screening passes, Rayyan can work well as a replacement or compliment to this workflow.

Keywords: systematic reviews, citation management, web applications, screening tools

Poster Number: 24

Time: Monday, May 21, 2:30 PM – 3:25 PM

Adapting Research Impact Assessment to Laboratory Practice Guideline Development Projects

Carol F. Colasacco, AHIP, Medical Librarian Specialist, Pathology and Laboratory Quality Center, College of American Pathologists, Pleasant Prairie, WI

Objectives: We hypothesized that an existing research impact framework could be adapted for laboratory practice guideline impact assessment in a non-academic environment, that this framework would improve efficiency and consistency when gathering and reporting impact indicators, and that indicator data could inform both strategic planning and dissemination processes.

Methods: A literature search helped identify existing research impact frameworks. Laboratory-specific indicators were incorporated into the selected framework design, a data input form was developed, and data was collected for each guideline based on its initial publication date through December 31, 2017. Quantitative and qualitative assessment was completed on the body of guidelines as a whole and on individual guidelines as data allowed. All data was reviewed to identify strengths and opportunities of each guideline, to evaluate the framework's design and usefulness, and to review dissemination method effectiveness.

Results: The amount of data available varied significantly based on the individual framework domain, the guideline topic, and the publication date. Publication metrics and citation numbers allowed us to appreciate the international reach of the entire body of guidelines as well as to compare individual guidelines. In several cases, a "deeper dive" into specific citations provided a clearer view of how individual guidelines were used to inform specific laboratory practices. Locating indicators in unindexed sources was time intensive but did identify examples of significant impact. An initial report was presented to the guideline development oversight committee (Center Committee) at the College of American Pathologists (CAP) in January 2018, and an analytics report is now included in the agenda book for every Center Committee meeting (three times per year). A detailed summary report will be presented at an upcoming Center Committee meeting when all data is collected and analyzed.

Conclusions: A research impact framework tailored to a non-academic, guideline development environment is an effective means to gather valuable metrics that can provide a "snapshot in time" of awareness and impact of a guideline. Indicator data can be used to keep concerned parties informed (e.g., guideline development panels, organizational oversight committees, partner organizations), help inform guideline topic selection, and help assess guideline dissemination efforts. A systematic means to gather data standardizes and quantifies guideline impact evaluation more objectively than relying upon anecdotal evidence reports.

Keywords: research impact framework
publication impact
guideline development
research impact assessment

Poster Number: 25

Time: Tuesday, May 22, 1:00 PM – 1:55 PM

An Innovative Tool for National Institutes of Health Public Access Policy Compliance

Merle Rosenzweig, Informationist; Tyler Nix, Informationist; Chase Masters, Informationist; Taubman Health Sciences Library, Ann Arbor, MI

Objectives: On April 7, 2008, the National Institutes of Health instituted the NIH Public Access Policy (NIHPAP). In order to apply for and to continue receiving NIH funding, a researcher needs to comply with the NIHPAP. This poster shows the innovative and interactive graphic tool that we have created to help facilitate and improve the process for those who must comply.

Methods: There are many resources that address compliance with the National Institutes of Health Public Access Policy but most are text heavy. Our goal was to create a graphic that would simplify this complex process through an interactive visual. We broke down the myriad tasks involved and represented them in an interactive flow chart. By accomplishing each individual task, the NIH Grantee can achieve compliance with the National Institutes of Health Public Access Policy.

Results: By representing the complex task of compliance with the NIHPAP as a flow chart, we have been able to create an interactive tool that, when adhered to, achieves compliance. We have incorporated this tool into our LibGuide in the form of self-directive instructions that can be freely downloaded and saved for future referral as needed. We use this resource as a starting point in instructional workshops in presentations to faculty and administrators

Conclusions: Since the National Institutes of Health Health's Public Access Policy was launched, librarians and informationists have provided support and instruction to those needing to comply with it, including guides, lectures, and videos. Complying with the Policy can be a daunting task that can be time consuming and frustrating. The interactive graphic that we have developed, we believe, has helped our researchers with attaining that compliance

Keywords: NIH Public Access Policy, Compliance, Instruction

Poster Number: 26

Time: Tuesday, May 22, 2:00 PM – 2:55 PM

Introducing High School Students to the Library Profession

Sharon A. Purtee, Catalog Librarian, University of Cincinnati, Health Sciences Library/Technical Services, Cincinnati, OH; **Lori E. Harris**, Associate Director for the Donald C. Harrison Health Sciences Library and the Henry R. Winkler Center for the History of the Health Professions, University of Cincinnati, University of Cincinnati, Cincinnati, OH

Objectives: The Health Sciences Library, as a corporate work partner, has accepted a student for the past three school years from DePaul Christo Rey (DPCR), a local private high school for low-income students, to expose the student to the workings of an academic medical library, the benefits of higher education, and to diversify their cultural universe.

Methods: Each week, students work in Technical Services, Circulation and the Archives Department performing a variety of tasks equivalent to entry-level student assistants. Weekly mentoring plus interactions with staff and library patrons enhance their work experiences. Students are evaluated weekly on their tasks completed and the quality of the work. Those are submitted to the school and shared with the student. Library staff submit quarterly grades to the school that become part of the student's permanent record. Staff from DPCR also visit twice per academic year to meet with the student and on-site library supervisor to discuss their progress and address any challenges. The HSL is working to change outcomes for DPCR students while simultaneously introducing a diverse pool of young candidates at the high school level to the information and library science profession.

Results: The students build their computer skills and database knowledge as well as develop customer service, time management, critical thinking, and overall communication skills. They are introduced to a deeper level of diversity and inclusion due to the broad demographics of a university population. The library staff invests time to mentor each student. Direct supervisors developed an outcomes' template that has been adopted by the school for other corporate partners.

Conclusions: The academic/corporate work partner format at DPCR has been quite successful. Student achievement is high and 100% of the students in each of the four graduating classes has been accepted at a two or four year college. The students who have spent time at the HSL were in either their first or second year of high school and, while we cannot predict their future scholastic pursuit, we are confident that we exposed them to a variety of options within Information Sciences to consider it as a viable career path.

Keywords: high school, students, student assistants, career planning, recruiting

Poster Number: 27

Time: Sunday, May 20, 2:00 PM – 2:55 PM

Creating Quality LibGuides Using Best Clinical Resources

Jie Li, Assistance Director, Collection Management, Biomedical Library, University of South Alabama, Mobile, AL

Objectives: Even though the library subscribes to many useful clinical resources, many books, journals and other resources, especially ebooks are not being used by clinicians, residents and students. Because these resources are included in various packages, making them discoverable is important. Creating clinical resource LibGuides may make these resources more visible and easy to find. This poster discusses how to include the best sources in clinical resource LibGuides.

Methods: In order to include the best sources in clinical resource LibGuides, the author uses Journal Citation Reports, Eigenfactor, Journal Metrics, and Scimago to identify the best journals for clinical subjects and Doody's Core Titles, Brandon/Hill Selected List of Print Books and Journals for The Small Medical Library and MLA Master Guide to Authoritative Information Resources in the Health Sciences to choose the best books for the clinical disciplines. The author also solicits input from clinical clerkship directors and resident directors.

Results: Most users find their journal articles by browsing a database, such as PubMed or CINAHL. However, ebooks are not as easy to find as journal articles. Clinical resource LibGuides stress ebooks more than ejournals. After creating LibGuides, the usage of library resources, especially ebooks have greatly increased. They have been welcomed by clerkship and resident directors, and appreciated by residents and medical students. Journals included in the clinical resource LibGuides are convenient for users to browse regularly.

Conclusion: Including quality resources in LibGuides is one way to make library resources more visible and, therefore, used.

Keywords: Libguides, Clinical Resources

Poster Number: 28

Time: Monday, May 21, 2:30 PM – 3:25 PM

Transformations in Research Support for Public Health Scholars

Catherine (Kay) H. Smith, AHIP, Professor/Senior Research Librarian, University of Alabama at Birmingham, Lister Hill Library of the Health Sciences, Birmingham, AL; **Sarah Safranek**, Public Health & Primary Care Librarian, Health Sciences Library, University of Washington Health Sciences Library, Seattle, WA

Objectives: To report results of a project assessing research support needs of public health scholars, with the aim of expanding and developing library programs and services for this clientele. Our presentation reports study results from two institutions, located in the northwestern US, and southeastern US. The local studies were conducted as part of the Ithaka Research Support Services: Public Health project.

Methods: Librarians at each institution conducted one-on-one, semi-structured, recorded interviews, with a total of 27 public health researchers from a variety of disciplines and at various career points. Interview questions explored research methods, data sources, research management software and tools, challenges encountered conducting research, and storing and sharing research output, and researcher outlook on developments, trends and future challenges in the field of public health. Common patterns and themes were distilled from interview transcripts using grounded theory methodology. Key findings related to potential library services were identified and described.
SETTING: Schools of public health at sample academic institutions within the U.S.
POPULATION: Faculty public health researchers.

Results: Each library identified 3-5 themes reflecting the specific focus of the institution's public health researchers and their particular library support needs. These were sampled by the Ithaka S+R group along with those of the other 5 participating libraries to produce a synthesis report of 4 key challenges and 11 recommendations for libraries serving public health scholars. While the 2 libraries presenting here identified some common concerns, especially related to open access journal quality, and training and assistance needs in a numbers of areas such as grey literature and management of research results, there were also notable differences. The differences may be reflective of the types of research performed in each institution.

Conclusions: The following were identified as important common support concerns by the public health researchers at the two libraries participating in this study.

*Because of the collaborative nature of public health research, universal access to full text literature is needed by all collaborators anywhere at any time.
*Training and assistance is needed with searching, management of research results, data management and sharing, and grey literature, as well as how to identify good quality open access journals.
*Libraries need to promote their services more effectively, as many researchers are not aware of existing support services.
These concerns were highlighted among others in the synthesis created by Ithaka S+R for their report.

Keywords: Public health, researchers, research support, research methods

Poster Number: 29

Time: Tuesday, May 22, 1:00 PM – 1:55 PM

Ask the Research Experts: Everything in One Convenient Location @ the Lamar Soutter Library

Tess Grynoch, Research Data & Scholarly Communication Librarian, University of Massachusetts Medical School, Lamar Soutter Library, UMass Medical School, Worcester, MA; **Mary Piorun, AHIP**, Director, Library Services and Director, NNLM NER, Lamar Soutter Library, Lamar Soutter Library, Worcester, MA; **Regina Fisher. Raboin**, Associate Director for Library Education, Research, and Technology Initiatives, University of Massachusetts Medical School, Lamar Soutter Library, Worcester, MA

Objectives: Many departments provide various research support services for researchers but they are spread out across campus and are not easy to visit. Could the Library provide a central location for research support services to provide easy and convenient access for patrons and increased visibility for the services?

Methods: The Library and the Institutional Review Board (IRB) initially proposed the service and recruited other research support departments to be available for drop-in sessions on the second and fourth Thursday of every month from 1-2:30pm. The Library provided the space in the form of booking time in the Library's dedicated classroom and two consult rooms; and helped provide promotional materials. The pilot run of Ask an Expert drop-in sessions launched in August 2017.

Results: The initial group of five support services, the Library, IRB, Institutional Animal Care and Use Committee, Quantitative Methods Core, and Sponsored Programs grew to seven groups with the addition of Capstone Advisors and the Center for Clinical and Translational Science. Out of the four drop-in sessions in the first two months of the pilot, there have been 21 attendees. The majority of attendees (19) had questions for the Institutional Review Board experts. (Updated results will be added to the presentation).

Conclusions: The pilot successfully gathered a number of departments which are usually disparate from each other into a central location, and the Library has been recognized as a central welcoming space for support services by colleagues from the other departments. One of the challenges facing the program is finding a way to ensure that a member from each service attends each session, and promoting the service to gain questions for departments other than IRB. Overall, the pilot has succeeded in providing a proof of concept that the library is an ideal centralized location for these types of academic support services.

Keywords: Service, Collaboration, Research Support

Poster Number: 30

Time: Tuesday, May 22, 2:00 PM – 2:55 PM

Exploring Racial and Ethnic Health Disparities Research Citation Patterns

Rachel J. Hinrichs, AHIP, Health Sciences Librarian; **Caitlin Ann. Pike, AHIP**, Nursing Librarian; University Library, Indianapolis, IN

Objective: The study identifies the most heavily cited journal titles, publication types, and subject disciplines in racial and ethnic health disparities research. The overall goal is to assist librarians with collection assessment for diversity and disparity-related research, and to provide a resource to assist faculty with identifying potential sources for publication. Methods: Using a modified version of the literature mapping protocol developed by the Nursing and Allied Health Resources Section, this study analyzed the references from research articles published in 2016 in racial and ethnic health disparities journals. Four journals were selected based on coverage of racial and ethnic disparities, and input from health disparities researchers. For each reference, publication type, publication date, and journal title, if it is an article, was recorded. To identify the core journals, cited journals were divided into three zones using Bradford's Law of Scattering. A single subject discipline was assigned to each core journal based on Library of Congress classification, as determined by Ulrich's Web. Results: 332 articles from four journals yielded 13,023 references. Journal articles were the most heavily cited publication type(n=10,596, 81%), followed by government reports (n=1005, 8%). Age of citations ranged 163 years, with 41% (n=5339) of citations occurring within the previous 8 years. The peak age of citations for articles was 6 years. Forty-two core journals accounted for 33.8% of all citations. The most common subject disciplines of these core journals were medicine (n=15), and public health and population health (n=15), followed by ethnic interests (n=4). Conclusion: Similar to other public health-related fields, racial and ethnic health disparity research draws from a very diverse pool of subject disciplines from medicine to public health to the social sciences, and relies on older articles and reports published within the last twenty years. However, racial and ethnic disparity research does not rely as heavily on government reports or miscellaneous items as other public health and social services disciplines.

Poster Number: 31

Time: Sunday, May 20, 2:00 PM – 2:55 PM

Design and Validation of Search Filters for LGBTQ+ Populations

Robin M. N. Parker, Evidence Synthesis and Information Services Librarian, WK Kellogg Health Sciences Library, WK Kellogg Health Sciences Library, Halifax, NS, Canada; **Amanda Wanner, AHIP**, Information Specialist and Research Fellow, University of Plymouth, Primary and Community Care Research Group, Devon, England, United Kingdom; **Margaret Foster, AHIP**, Associate Professor, Medical Sciences Library, Medical Sciences Library, College Station, TX; **Mellanye Lackey**, Associate Director for Education and Research, University of Utah, Spencer S. Eccles Health Sciences Library, Salt Lake City, UT; **Joseph G. L. Lee**, Assistant Professor, East Carolina University, East Carolina University, Greenville, NC

Objectives: The health of LGBTQ+ people is a subject area that is challenging to search due to variant and changing terminology. This presents a challenge when developing comprehensive searches for systematic reviews. The objective for this project is to develop and validate a PubMed search filter to identify research concerning LGBTQ+ populations for systematic reviews.

Methods: We used relative recall to test sets of search terms based on the included studies from recent systematic reviews relating to LGBTQ+ topics. We searched for systematic reviews on LGBTQ+ topics in six databases and extracted the included studies from a selection of recent reviews. The resulting citations were split into a development set and a validation set. We tested combinations of index and text word terms in PubMed and used the development set to create and calculate the precision and recall of three search filters: sensitive, specific, and a balance of the two. The final search filters were then tested against the validation set to confirm the performance measures.

Results: We created a test set and a validation set of gold standard citations using the relative recall method. The search filters and their performance metrics will be presented.

Conclusions: A validated search filter is an easier and more reliable approach to retrieve relevant literature for reviews on topics related to this minority population.

Keywords: systematic review; search filter; LGBTQ+

Poster Number: 32

Time: Monday, May 21, 2:30 PM – 3:25 PM

Librarians Teaching Evidence-Based Practice to New Grad Nursing Residency Program

Catisha R. Benjamin, Manager of Library Services, Clinical and Research Library, Aurora, CO; **Marie St. Pierre, AHIP**, Librarian II, Children's Hospital Colorado, Clinical and Research Library, Aurora, CO; **Donnya Mogensen**, Nurse Residency Manager, Children's Hospital Colorado, Children's Hospital Colorado, Aurora, CO

Objectives: The librarians have presented for several years now to the New Graduate Nurse Residency Program, teaching library resources, literature searching, and evidence based practice. It benefits them directly in their evidence based projects that are a part of the program, leading to a poster presentation of their work.

Methods: The librarians present classes to the program participants, showing the library's resources, discussing how to form a PICO/T question and translate that into an effective literature search, and are on hand to assist during mentored work time as the participants are gathering their data for their projects. Reference management systems are taught also.

Results: Participants demonstrate knowledge of PICO/T questions and translating them into effective literature searches, and then use this to successfully search library resources for the appropriate evidence-based articles or systematic reviews to support their projects. These projects are then carried out in their clinical settings, and a poster session is presented to show their work. Librarians are on hand at all steps of the way and often field questions during any part of the process. The nurses are prepared for EBP in their careers and establish a solid working relationship with their librarians.

Conclusions: Librarians with a knowledge of evidence-based practice are ideally suited to teach nursing staff of all degrees of experience, The librarians at Children's Hospital Colorado collaborate with the New Graduate Nurse Residency program to reinforce or refresh their knowledge and skills in EBP in the hospital setting.

Keywords: nursing education, evidence-based practice, library instruction, hospital librarianship, clinical librarianship

Poster Number: 33

Time: Tuesday, May 22, 1:00 PM – 1:55 PM

Assessment of Pharmacy Students Drug Information Skills and Self-Efficacy Perceptions through a Virtual Poster Session

James Wheeler, Assistant Professor, PharmD, BCPS, University of Tennessee Health Science Center, Clinical Pharmacy, Nashville, TN; **Lin Wu, AHIP**, Assistant Director/Associate Professor, Research & Learning Services, University of Tennessee Health Science Center, Health Sciences Library, Memphis, TN; **Martha Earl, AHIP**, Director, Preston Medical Library, University of Tennessee Preston Medical Library, Knoxville, TN; **Eric Robert. Heidel**, Associate Professor, University of Tennessee Graduate School of Medicine, Department of Surgery, Division of Biostatistics, Knoxville, TN

Objectives: Health professions students often struggle with evidenced based medicine (EBM) concepts including formulating clinical questions, retrieving relevant information, evaluating literature for relevance, and applying evidence to practice. This study aims to assess team performance and student perceptions of self-efficacy regarding literature search and drug information skills via an assigned team project in a required drug information course.

Methods: In collaboration with pharmacy faculty, two health sciences librarians delivered foundational lectures on using library resources, conducting literature searches, using the PICO (Population, Intervention, Comparison, and Outcome) format to construct drug information questions, and reviewing medical writing and citing guidelines. Teams (n=26) composed of 6 to 7 second year student pharmacists were assigned clinical drug information requests. Utilizing the PICO format, groups conducted literature searches, authored formal responses to a drug information question, prepared counseling pearls for the lay patient, and presented their projects via a virtual poster session hosted across three campuses in within Blackboard, the University's LMS. The EBM C-PET, a validated rubric, was used to evaluate each team’s project. As a secondary measure, students completed pre-and post-class self-efficacy assessments describing their confidence in drug information processes.

Results: The mean project score was 43/50 points. One hundred one students completed the pre and post self-efficacy survey. Perceived gains in confidence occured in all ten categories surveyed including: medical writing skills, interpretation and combination of information into concise responses, knowledge of library resources, and Medline searching skills (all $p<0.001$).

Conclusions: Students performed well in the team-based drug information and displayed significant gains in confidence. Faculty-librarian collaboration could be an effective way to prepare students with EBM skills needed for pharmacy practice.

Keywords: Assessment, Information literacy, Drug information responses, Evidence-based pharmacy, Self-efficacy

Poster Number: 34

Time: Tuesday, May 22, 2:00 PM – 2:55 PM

Immigrants in the Sciences

Nisha Mody, Health & Life Sciences Librarian, UCLA, Biomedical Library, Los Angeles, CA; **Courtney Hoffner**, Librarian, UCLA, Science and Engineering Library, Los Angeles, CA; **Mary Zide**, Sciences Data Informationist, UCLA, Biomedical Library & Science and Engineering Library, Los Angeles, CA

Objectives: In response to statements made by the XX President, XXXX Chancellor, and Vice Chancellor of Equity, Diversity, and Inclusion regarding polarizing sociopolitical events, the Science Libraries will create outreach programs to promote campus inclusivity. These initiatives will raise awareness, create safe library spaces, and dismantle library neutrality in the university community.

Methods: The Science Libraries will engage the community in dialogue through the library about current sociopolitical issues to encourage the larger mission of the university and ethical duty of librarianship because "[w]e cannot pretend that by sitting still -- by claiming to be neutral -- we can avoid accountability for our roles" (Jensen, 2008). The Vice Chancellor of Equity, Diversity, and Inclusion stated that "even if [institution]-as-sovereign remains neutral, [institution]-as-speaker can take a stand" (2017). We are taking action in order to oppose discrimination and violence against our patrons from marginalized communities. The science library spaces include the Biomedical Library and the Science and Engineering Library. Outreach initiatives will include interactive exhibits, events, and tools to engage patrons. These initiatives will specifically respond to the DACA reversal, discriminatory demonstrations (e.g. Charlottesville, VA), and other divisive policies in the current political climate.

Results: A total of 1071 pins were counted between the two libraries. We used Tableau to create two data visualizations. The first included the number of pins in each country and identified which of these countries are active DACA recipients. The second visualization are the top 20 non-US countries"pinned" by our patrons. We received very positive feedback about this exhibit.

Conclusions: This was a positive exhibit that demonstrated how the sciences can take a stand and support our patrons. Some of the printing and setup of this exhibit took longer than expected. In the future, we plan to utilize student workers to support our exhibits which will also expand our perspectives. We deemed this a success and look forward to future opportunities to demonstrate library solidarity.

Keywords: immigration, health sciences, sciences, diversity, outreach, exhibits, community, library spaces

Poster Number: 35

Time: Sunday, May 20, 2:00 PM – 2:55 PM

Developing a Protocol for Tracking Publications by Grant Numbers

Megan von Isenburg, AHIP, Associate Dean, Medical Center Library & Archives, Medical Center Library, Durham, NC; **Virginia Carden, AHIP**, Administrative Research Librarian, Duke University, Medical Center Library & Archives, Durham, NC; **Emily S. Mazure, AHIP**, Biomedical Research Liaison Librarian, Medical Center Library & Archives, Duke University Medical Center Library & Archives, Durham, NC; **Jesse Akman**, Intern, Duke University Medical Center, Library - Research & Education, Burlington, NC

Objectives: At this institution, the Clinical & Translational Science Institute (CTSI) tracked publications that cited NIH Clinical & Translational Science Awards (CTSA) using only PubMed. As part of a new partnership with the library, librarians sought to discover and document additional resources and best practices for tracking CTSA-funded publications.

Methods: Upon receiving an NIH Informationist Administrative Supplement award, librarians worked with the CTSI evaluation core to develop new methods of demonstrating the impact of CTSA funding. Initially, it was necessary to ensure comprehensive tracking of publications that cite the CTSA grants. Librarians developed searches using variants of the CTSA grant numbers in PubMed, Web of Science, Scopus, and Embase databases. Additionally, PMC, PACM and NIH Reporter were searched. Retrieved citations were added to EndNote for analysis. Results from all sources were analyzed to determine which databases resulted in the highest retrieval, and to assess the overlap between sources. The final search results were compared against publication lists historically gathered by CTSI. From those findings a standardized search protocol was developed for tracking publications in the future.

Results:
The new process found all of the citations that had been on the original CTSI list and 138 new citations that had not been found earlier. 97.5% of articles were found in PubMed. Web of Science offered the most additional citations compared to Scopus and Embase. Scopus found no unique results. No additional citations were found in the grey literature sources that were not found in the next periodic search of databases.

Conclusions:
To find all citations in the most efficient way, search the databases in the following order: 1. PubMed, 2. Web of Science, 3. Embase, 4. Scopus. Future steps include bibliographic analysis of the identified citations.
Keywords: publications, grants, CTSA, searching

Poster Number: 37

Time: Tuesday, May 22, 1:00 PM – 1:55 PM

Barriers to Students' Access to Clinical Information Resources

Nicole Mitchell, Information Technology Librarian and Associate Professor of Library & Information Sciences; **Michelle Miller**, Senior Library Associate; Alabama College of Osteopathic Medicine, Library/Learning Resource Center, Dothan, AL

Objectives: To assess student physicians' barriers to access clinical information resources during their 3rd and 4th year clerkships/clinical rotations.

Methods: Students submit an end-of-clerkship survey at the end of each core clerkship. In addition to questions about their specific preceptor and clerkship, students are asked to rate and comment on the ease of use in accessing library resources, indicate the usefulness of the acquired information in meeting patient care objectives, identify the device used most frequently for accessing clinical information, and highlight the most frequent barriers to accessing clinical information at the bedside, on rounds, with preceptors, and after hours. Students also provide suggestions for ways to improve access to library information during clerkships. Library staff collect and compile the survey responses each month. Responses will be analyzed in efforts to ascertain the types of barriers students encountered when accessing clinical information resources.

Results: Results will be presented during the poster session as data collection and analysis is ongoing.

Conclusions: Conclusion will be presented during the poster session.

Keywords: students, clerkships, access

Poster Number: 38

Time: Tuesday, May 22, 2:00 PM – 2:55 PM

Building and Maintaining a Literature Search Service

Lilian Hoffecker, Research librarian, Health Sciences Library, Health Sciences Library, Aurora, CO; **Ben D. Harnke**, Education & Reference Librarian, University of Colorado Anschutz Medical Campus, Education and Reference, Aurora, CO; **Kristen DeSanto, AHIP**, Clinical Librarian, Health Sciences Library, Health Sciences Library, Aurora, CO

Objectives: As systematic reviews increase in prominence for the practice of evidence based health care, librarians have seen a rise in requests for expert searching. The demand has led to an evolving literature search service with a team of searchers at an academic health sciences campus. The authors describe the successes and challenges of the service.

Methods: The searching team, consisting of three members, utilizes an online form to receive search requests. The searchers take similar approaches to search strategy development, and organize citations using Endnote while managing systematic reviews projects with Covidence. These and other steps that they practice in common assure consistency and reproducibility of results. Recently, working with the IT department, the search team developed a web form that gathers data to track the service, which in turn can help with its evaluation and analysis. The team meets regularly to discuss issues such as fees to charge, publication authorship, training, staff capacity, and major trends in the field. The service has experienced difficulties meeting the requests for specialized searching but at the same time, it has observed growing recognition by researchers of the value we bring to their projects.

Results:

Conclusions:

Keywords: library services, searching, systematic reviews, project management, librarian authorship

Poster Number: 39

Time: Sunday, May 20, 2:00 PM – 2:55 PM

Creating a Consortium Task Force to Assess E-Resource Accessibility

JJ Pionke, Assistant Professor and Applied Health Sciences Librarian, Social Science, Health, and Education Library at the University of Illinois Urbana Champaign, Social Sciences, Health, and Education Library at UIUC, Champaign, IL; **Elizabeth Sosnowska**, Head of Health Sciences Collection Development & Collection Assessment Librarian, Rutgers University, Rutgers University - Health Sciences Libraries, Newark, NJ; **Heidi M. Schroeder**, Accessibility Coordinator, Michigan State University, Michigan State University Libraries, East Lansing, MI

Objectives: This poster showcases the creation and goals of the Big Ten Academic Alliance (BTAA) Library E-Resource Accessibility Group, which formed to assist libraries in addressing the problem of vendor-supplied electronic resources, many of which do not comply with accessibility standards or are not usable for patrons with disabilities. It contains examples e-resource accessibility testing and sample accessibility license language.

Methods: Due to a growing concern about the inaccessibility of many library electronic resources, the BTAA Libraries formed an E-Resource Accessibility Group to assess the accessibility of major resources, influence vendors, and ultimately advance the field of electronic resource accessibility by sharing information. The Accessibility Group has two subgroups, each of which focus on a different part of the larger charge. The testing subgroup has focused on the evaluation and hiring of companies to do the actual accessibility testing as well as acting as a liaison between the vendor and the testing company as testing takes place. The license language subgroup has focused on not only the creation of boilerplate accessibility language for our contracts with vendors, but also in surveying the field to see what language Big Ten Libraries have been able to successfully negotiate in regards to accessibility.

Results: To increase awareness of e-resource accessibility concerns thereby drawing attention to the need for accessibility in both the e-resources that so many libraries use as well as in the contract language that we agree to.

Conclusions:

Keywords: e-resources, Big Ten Academic Alliance, accessibility, disability, license language

Poster Number: 40

Time: Monday, May 21, 2:30 PM – 3:25 PM

Transforming the Library's Education Services One Systematic Review at a Time

Darlene Parker. Kelly, Director, Charles R. Drew University, Charles R. Drew University, Los Angeles, CA; **Andre N. Kharabi**, Graduate, Jagiellonian University, Medical College, Humble, TX; **Shahrzad Bazargan-Hejazi**, Professor, Charles R. Drew University, College of Medicine, Los Angeles, CA

Objectives: At the University, the College of Medicine requires each fourth year student to participate in the medical student research thesis program (MSRTP). Over the course of the past few years, we are being asked to assist students in the systematic review process. Initially, it was the students contacting the librarian, however this process is being modified due to creation of partners who are working more closely with the librarian. The purpose of this study is describe the exploratory methods used by a small health sciences library to become an active partner in the systematic review methodology.

Methods: The librarian met with the student and the team to discuss the research question and the systematic review process. The librarian provided an overview of the library resources and when applicable provided mediated searching. Students were instructed to conduct a literature review using PubMed, Web of Science, Psych-Info, EMBASE and Cochrane Review using the MESH terms relevant to their subject. . Articles were screened using specific eligibility criteria and the selected articles were submitted to the librarian to be independently screened. The transparency of the selected articles were further evaluated using the TREND statement checklist.

Results: Students and faculty team members reported that they were more confident with the process of searching the medical literature and using standards that are adopted when conducting systematic reviews. This initial activity has created additional opportunities for the librarians to support other students who are pursuing systematic reviews.

Conclusions: Librarians can start small and begin to have an impact by contributing to students' success in curating the literature. The team approach provided invaluable knowledge on the process and also on the outcome of the review.

Keywords: Systematic Reviews, medical students, thesis, libraries, librarians

Poster Number: 41

Time: Tuesday, May 22, 1:00 PM – 1:55 PM

Book Discussion Groups for Health Sciences Interprofessional Education: An Interprofessional Education Pilot Project

Rebecca C. McCall, AHIP, Clinical Librarian, University of North Carolina-Chapel Hill, University of North Carolina-Chapel Hill, Chapel Hill, NC; **Lee Richardson**, Information Discovery and Metadata Librarian, University of North Carolina at Chapel Hill, Health Sciences Library, Chapel Hill, NC; **Meg Zomorodi**, Clinical Associate Professor, University of North Carolina-Chapel Hill, School of Nursing, Chapel Hill, NC; **Elizabeth Moreton**, Nursing Librarian, University of North Carolina-Chapel Hill, Health Sciences Library, Chapel Hill, NC

Objectives: Academic health sciences librarians worked with the Interprofessional Education (IPE) Steering Committee to organize interprofessional book discussion groups for incoming health sciences students in allied health, dentistry, medicine, nursing, pharmacy, public health, and social work. These inaugural book discussion brought together students and faculty of different disciplines in order to engage students to ‘learn from, with, and about’ other professions.

Methods: When Breath Becomes Air, by Paul Kalanithi, was chosen to read, as the group felt that it would be relevant to all the health professions, and would allow for involved discussion on important health sciences issues. The timeline of this pilot project, its successes, and challenges, and results of participant surveys will be presented. The project included outreach to invite faculty and staff to facilitate the discussions, designing pre and post surveys, scheduling students and facilitators into interprofessional groups for the discussions, and communicating with all participants before, during, and after the event. Book discussions in small groups were held in the health sciences library and in various health schools on campus.

Results: A total of 79 students and 36 faculty, representing all health sciences schools, participated in the interprofessional education book discussions over two weeks in August 2017. Students completed pre-discussion surveys on their IPE knowledge and attitudes and both students and facilitators completed a post-discussion evaluation survey. Initial feedback has been very positive. Many participants requested more interprofessional book discussions and more opportunities to interact with students in other disciplines. The qualitative and quantitative data collected from these surveys shows the value attendees and facilitators find in IPE and the desire for more collaborative opportunities.

Conclusions: The committee learned from this pilot program, which will ensure a smoother process for future IPE book discussions. The results of the participant surveys were very positive and the IPE Steering Committee also found value in the inclusion of health sciences librarians throughout the process. Other lessons learned from the pilot project include the need for an efficient scheduling system, strong communication at all stages of the project, and beginning the planning process months ahead of time. The committee plans to conduct similar book discussions every fall semester moving forward and also explore options for other, varied interprofessional education events.

Keywords: IPE, interprofessional education, interprofessional collaboration, health sciences education, academic libraries

Poster Number: 42

Time: Tuesday, May 22, 2:00 PM – 2:55 PM

Development of a Writing and Publishing Series for National Institutes of Health and US Department of Health and Human Services Analysts, Clinicians, Fellows, and Researchers

Holly Thompson, Informationist, NIH Library, Bethesda, MD; **Alicia Livinski**, Biomedical Librarian/Informationist, NIH Library, National Institutes of Health Library, Annandale, VA; , university; , Institution

Objectives: To build and strengthen existing skills of HHS and NIH staff, a series of writing and publishing classes was developed. Topics covered the “how tos” of writing an abstract and research paper to creating a poster presentation. The series will prepare learners at all levels of their education and careers to communicate their research in a variety of professional settings.

Methods: A scan of NIH and NIHL resources was conducted to identify gaps in available training opportunities and topics covered. Informal conversations were held with HHS and NIH stakeholders to identify training needs. Additionally, current NIHL class schedules were reviewed to better coordinate with important NIH deadlines (e.g., abstract submission deadline for the annual NIH Research Festival) and/or seasonal onboarding of new fellows/trainees (e.g., the arrival of summer interns). Nine new classes on the “how tos” of writing a research paper, abstract writing, poster development, journal publishing, clinical research study design, hypothesis testing, statistics for manuscripts, and common statistical tests were piloted in 2017 to gauge interest in library-offered classes related to writing, scholarly communication, and statistics.

Results: Results: To be reported in Spring 2018.

Conclusions: Conclusions: To be reported in Spring 2018.

Keywords: NIH

Poster Number: 43

Time: Sunday, May 20, 2:00 PM – 2:55 PM

Development of Bioinformatics Services in the Library: Understanding Researcher's Bioinformatics Support Needs

Joe Wu, Bioinformatics research support specialist, University of Florida, Health Science Center Library, Gainesville, FL; **Michele R. Tennant, AHIP**, Associate Director, Health Science Center Library, Biomedical and Health Information Services, Health Science Center Libraries, Gainesville, FL; **Hannah F. Norton, AHIP**, Reference & Liaison Librarian, University of Florida, Health Science Center Libraries, Gainesville, FL; **Mary E. Edwards**, Reference & Liaison Librarian, University of Florida, Health Science Center Libraries, Gainesville, FL

Objectives: Bioinformatics tasks include finding gene sequences at the basic level to analyzing complex genomic datasets (newly collected or from public repositories) to identify disease causing mutations. This discipline helps advance our understanding of biology and medicine. Proficiency in bioinformatics is critical for those working in life sciences and health. In these studies, we seek to obtain insight on the bioinformatics training needs at the University of Florida (UF). These insights will guide the Health Science Center Library's (HSCL) efforts to develop and implement innovative support services in bioinformatics.

Methods: We developed a survey in Qualtrics and distributed to biosciences researchers in the academic health center as well as relevant interdisciplinary institutes, departments in liberal arts and sciences, and the agriculture school. Researchers in these departments represent those where bioinformatics could enhance their research efforts. We designed this survey to gauge the researchers' skill level and training interests in bioinformatics tasks such as gene sequence retrieval and genomic data analysis. We also inquired about preferred mode of training.

Results: Our results reflect a demand for bioinformatics training. There is strong desire for training in complex bioinformatics tasks such as genomic data analysis, development of data analysis pipelines, and molecular biology network analysis. We observed that graduate students were less likely to rate their proficiency at the expert level as compared to faculty for basic bioinformatics tasks such as finding gene/protein sequences and gene expression information. Further, graduate students tended to be extremely interested in training for these bioinformatics tasks as compared to faculty. Finally, classes, individual in-person consultations, and online video tutorials ranked among the top three in preferred modes of training.

Conclusions:A need for bioinformatics training has been demonstrated. To address the interest in advanced bioinformatics training (i.e. genomic data analysis), the HSCL's bioinformatics support specialist has taken the initiative to gain expertise in these areas as well as connect with bioinformatics experts on campus and vendors who may be invited to conduct advanced bioinformatics training for our clients. The HSCL will continue to address graduate student's training needs in basic bioinformatics tasks (i.e. searching for molecular biology data) through a credit-bearing course that is offered during the fall semester. Last, a recent grant award will allow the HSCL to hire an intern who will begin to evaluate existing and create new resources addressing the use of bioinformatics tools to meet the demand for online video tutorials.

Keywords:bioinformatics, developing bioinformatics support services, bioinformatics training needs, survey

Poster Number: 44

Time: Monday, May 21, 2:30 PM – 3:25 PM

Availability of Key Resources in Libraries Supporting Physician Assistant Programs: Equal Access in a Time of Rapid Growth?

David Petersen, AHIP, Assistant Librarian, Health & Human Services, Florida Gulf Coast University Library, Fort Myers, FL

Objectives: This study will examine libraries that support Physician Assistant (PA) programs (n=214) to analyze subscriptions for eight key resources. What resources in this list are purchased the most? Do libraries that support newer PA programs have as many resources as their older counterparts? Are libraries that also support medical programs (M.D./D.O.) providing greater access to the selected resources compared to libraries that do not support a medical program?

Methods: The author consulted the Physician Assistant Education Association's (PAEA) directory of PA programs (n=256) for institutions, accreditation status, and initial accreditation dates. The directory includes extended sites that are served by the library at the school's primary location. For the purposes of this study, school with extension or multi-campus sites that are served by a central library were only counted once. After eliminating these results, 214 libraries remained; data was compiled for 211 of 214 possible libraries (98.6%) in a spreadsheet. The data collection procedure featured a multi-stage process. Each institution's website was consulted to see if a medical program existed. Next, each library's holdings were checked for subscriptions to any of the following eight resources: Access Medicine, CINAHL, Clinical Key, Cochrane Library, DynaMed, Scopus, UpToDate, and Web of Science. In some cases, verification was done by email.

Results: Data shows that CINAHL (93%), Cochrane Library (85%), and Access Medicine (71%) enjoy the highest subscription rates among these libraries while Scopus (29%) was the least subscribed product. Libraries that support long standing PA programs have access to more resources from the selected list than libraries that support newly accredited programs. Additionally, libraries that also support a medical program have significantly higher access to resources from the selected list compared to libraries that do not support a medical program.

Conclusions: Libraries serving long established PA programs and medical schools have access to greater resources compared to libraries with newly accredited programs or those not supporting a medical program. Still, over two-thirds of libraries had access to 3 - 6 resources from the select list. Further research is needed to assess whether this resource discrepancy influences educational outcomes. Librarians at universities/colleges that are in the process of developing a PA program should exercise caution and advocate for additional funding, particularly if the institution does not already support a medical program.

Keywords: Physician Assistant programs
Library subscriptions
Resource access
Equal access

Poster Number: 45

Time: Tuesday, May 22, 1:00 PM – 1:55 PM

Building Resilience through Research: National Institutes of Health Disaster Research Response Project

Siobhan Champ-Blackwell, Health Sciences Librarian, Disaster Information Management Research Center, National Library of Medicine Disaster Information Management Research Center, Kansas City, MO; **Stacey Arnesen**, Chief, Disaster Information Management Research Center, National Institues of Health, Specialized Information Services, Bethesda, MD; **Aubrey Miller**, Senior Medical Advisor, National Institutes of Health, National Instititute of Enviornmental Health Sciences, Bethesda, MD; **Robin Taylor**, Librarian, ICF (supporting NLM) , Bethesda, MD; **Steven Ramsey**, Project Manager, Social & Scientific Systems, Inc., Public Health Sciences Group, Durham, NC; **Richard Rosselli**, Study Manager, Social & Scientific Systems, Inc., Public Health Sciences Group, Durham, NC; **April Bennet**, Program Manager, National Institutes of Health, National Instititute of Enviornmental Health Sciences, Bethesda, MD

Objectives: The Disaster Research Response Program is a system that provides data collection tools and research protocols. Objectives: identifying research priorities; improving access to data collection tools; improving the ability to quickly collect data after a disaster; training and integration of DR2 into planning and response systems; and creating a disaster research process that includes public health, academia, and communities.

Methods: In response to a number of disasters, including the World Trade Center attack, Hurricanes Katrina and Sandy, the Gulf Oil Spill, and Ebola response, and the research conducted in their wake, a pilot program, developed by a federal environmental health agency and a federal library was developed. A variety of methods were used to develop the program: an evaluation of a long-standing need for clinical research in disasters (e.g., H1N1 experience); a review of data collection tools used in disasters; work on the development of a Public Health Emergency Research Review Board (with other agencies); a report with 10 recommendations to improve disaster research; a working group meeting of partners in conjunction with the Office of the Assistant Secretary for Preparedness and Response (ASPR); and a review of research tools as part of public health emergency response efforts.

Results:

Conclusions:

Keywords: research, protocols, data-collection,

Poster Number: 46

Time: Tuesday, May 22, 2:00 PM – 2:55 PM

Transforming the Website Redesign Process: An Intelligent Knowledge Management Approach of Inclusion

Elizabeth Frakes, Information Scientist, Center for Knowledge Management, Center for Knowledge Management, Nashville, TN; **Spencer J. DesAutels**, Information Scientist, Vanderbilt University Medical Center, Center for Knowledge Management, Nashville, TN; **Annette Williams**, Senior Information Scientist, Knowledge Management and Eskind Biomedical Library, Center for Knowledge Management (CKM), Nashville, TN; **Zachary E. Fox**, Associate Director for Information Services, Knowledge Management , Center for Knowledge Management (CKM), Nashville, TN; **Nunzia B. Giuse, FMLA**, Professor of Biomedical Informatics and Medicine; Vice President for Knowledge Management; Director for CKM, Eskind Biomedical Library/Knowledge Management, Center for Knowledge Management (CKM), Nashville, TN

Objectives: In an age of ever-increasing information, an organization's web presence is its foremost marketing tool. To conceive a new web presence representative of the entire team's shared vision, functions, and skills, this large academic medical center department's leadership designed a three-day retreat using intelligent information management processes to elicit total participation, buy-in, collaboration, ownership, and consensus of the entire staff.**Methods:** To ensure that the composite result would be reflective of all staff input, staff was stratified by professional experience, personality type, expertise, and work focus to form two well-balanced teams. Each team was intentionally assigned a captain not usually in a leadership position to facilitate that all voices be heard. Retreat sessions alternated between 1) team sessions to devise unique solutions to key problems; 2) full staff sessions to generate consensus from team sessions, formulate website blueprints, wireframes, look and feel, and define content organization; and 3) small groups of 4-5 individuals to compose content. Key to this iterative process was involving all individuals best situated to offer input in their areas of expertise and creating a dynamic and engaging environment. Leadership opened the retreat, energized the group, and gave it leeway to devise a creative solution.**Results:** As a result of the three-day retreat, a new website was designed, reflecting a consensus-driven representation of the team and embodying leadership's vision for the department's future direction. Positive outcomes include: a cohesive overall look and feel; design of wireframes for 82 pages; content outlined and described for each page; agreed-upon quarterly updates by team members with the appropriate content knowledge, and meaningful input from all team members. Additionally, the process allowed the team to brainstorm ways to increase communication of the department's mission via a dynamic and effective web presence that directly maps CKM projects into VUMC-established yearly priorities. **Conclusions:** An in-house retreat is a viable option to produce a consensus-driven vision and website. This collaborative process contributed to increased staff buy-in and input, and successfully produced publishable content for the website. A key takeaway from the experience is that having sessions moderated by people not normally in a leadership role garnered more participation, direct involvement, and enthusiastic buy-in as all comments were viewed and weighed equally, thus giving everyone the opportunity to reflect on a shared common vision of the website.

Poster Number: 47

Time: Sunday, May 20, 2:00 PM – 2:55 PM

Piloting a Training Program to Increase Medical Information Literacy Skills

Catherine Naeger, Lead Extramural Support Assistant; Lia Fleming, Pathways Intern; Center for Scientific Review, Bethesda, MD

Objectives: This proposal will explore the feasibility of training non-scientific support staff to utilize Elsevier Expert Lookup and Dimensions to identify experts to participate as panel members to review grant applications based off the content applications slated for review.

Methods: Our organization reviews thousands of health science grant applications per year. Finding and selecting panels of experts to review remains a challenge, and many review officers rely on their own networks to recruit review members. We propose training support staff on pre-existing software to assist with expert identification, even those with little to no scientific backgrounds. Staff will be trained to extract key concepts from the abstracts and specific aims of grants, and then match these against expertise found in publication databases. This process requires an understanding of natural language processing and controlled vocabularies as each system uses different methods to match expertise. A developmental training program is currently underway in one section of the organization. Implementation of this training program for support staff is expected to expand their medical literacy capabilities and provide skills-based growth in their careers.

Keywords: training development, information literacy

Poster Number: 48

Time: Monday, May 21, 2:30 PM – 3:25 PM

Transforming Clinicians to Published Authors: How Librarians Lead Students in Research and Scholarly Communication

Dede Rios, AHIP, Director of Optometric & Clinical Library Services, Optometry Library, Rosenberg School of Optometry, San Antonio, TX; **Laura Bredahl**, Librarian for Optometry, Vision Sciences and Biology, University of Waterloo, Witer Learning Resources Centre, Waterloo, ON, Canada

Objectives: This paper examines the effects of collaborative instruction practices by librarians at two Schools of Optometry on the research and scholarly communication skills and output of students at several stages of their clinical and academic training.

Methods: Librarians at two small optometry libraries instructed first to third year Doctor of Optometry students, and vision science graduate students on research and scholarly communication skills in several formats including, stand-alone orientations, curriculum-driven workshops, and in-person, post-assignment consultations. This paper describes the development of the instruction, its uptake and the post-event surveys used to evaluate the success of the instruction.

Results: Scholarly communication improved and the number of conference posters, presentations, and scholarly publications increased among the first to third year optometry students. Awareness and use of resources supporting scholarly communication increased by the vision science graduate students. Additionally, there was a greater understanding among faculty and staff at the schools of the benefits of collaborating with librarians for students success.

Conclusions: With strong initiatives through the Association of Research Libraries and Association of College and Research Libraries to promote the library-led outreach on scholarly communication, these vision science librarians represent diverse populations, interventions, and outcomes with the same purpose of improving their students' research and writing output. Collaborations allow librarians to broaden the services they can offer students, build relationships that support student scholarship and engagement, and potentially provide a means of dissemination. This paper shows that librarians are powerful advocates who facilitate positive change in scholarly communication, increase student engagement, and lead transformation.

Keywords: optometry, clinicians, collaboration, scholarly communications, research skills, resource awareness

Poster Number: 49

Time: Tuesday, May 22, 1:00 PM – 1:55 PM

Building an Assessment Model for Instruction

Tyler Moses, Visiting Librarian; **Mia S. White, AHIP**, Medical Education & Technologies Informationist; **Sandra G. Franklin, AHIP, FMLA**, Director; **Amy Allison, AHIP**, Associate Director; Woodruff Health Sciences Center Library, Atlanta, GA

Objectives: To develop an assessment model that will measure the impact of face to face and online instruction in an academic health sciences library.

Methods: Researchers will employ mixed methods strategies. Using the logic model as a basis, the researchers will examine current course offerings for academic and healthcare users, and outline the intended stakeholders, purposes, and goals. Analyzing the data collected prior to the study, researchers will identify current metrics used for assessing courses and determine whether other metrics need to be included.

Results: After analyzing the collected data and reviewing interviews with key stakeholders, the data will be used to determine if the goals and outcomes of the instructional services have been met.

Conclusions: Logic models serve as excellent tools to establish the foundations of an assessment plan as well serving as a framework to launch an instruction pilot.

Keywords: Assessment Model, Logic Model, Instruction Services, Mixed Methods, Learning Goals

Poster Number: 50

Time: Tuesday, May 22, 2:00 PM – 2:55 PM

Adapting to the Challenges of a Distributed Learning Library System

Electra Enslow, Head of Library Research and Instruction, Spokane Academic Library/Washington State University, Spokane, WA; **Suzanne Fricke, AHIP**, Animal Health Sciences Librarian, Animal Health Library, Pullman, WA; **Kathryn Vela, AHIP**, Health Sciences Outreach Librarian, Washington State University, Spokane Academic Library, Spokane, WA

Objectives: Health sciences librarians face increasing complexity when serving innovative health science programs with multiple campuses and distributed community-based and academic partners. This paper examines the challenges faced by these librarians as they work to coordinate services and meet user needs.

Methods: Librarians based on multiple campuses took a self-directed approach to better understand the needs of evolving distributed health science programs at their institution, using methods based on organizational information theory and team sensemaking. During six in-person and virtual meetings over three months, the librarians engaged in comparative discourse, examining the unique aspects of their library settings, collection practices and the specific health sciences program populations they serve. The librarians identified where systems are consistent, where workflows have been adjusted to meet local needs, and where further studies are needed.

Results: Analysis of discussions identified seven challenge areas: 1) reference services, 2) instruction, 3) management, 4) communication, 5) collections, 6) community outreach, and 7) library systems. Using these challenge areas, the librarians identified seven potential strategies to provide a more helpful and seamless experience for library users: 1) customize reference services, 2) engage in intentional communication, 3) focus limited budgets on resources that support multiple groups, 4) understand how library materials are currently being used, 5) understand how library materials will be used in the future, 6) work with library systems, and 7) network with librarians both on and off campus.

Conclusions: As a result of this comparative exploration of library services in a distributed learning library system, the health science librarians learned that differing technology systems, collection philosophies, service policies, outreach needs, and budgets contribute to the complexity of coordinating services. The librarians will continue to take a team-based approach to addressing issues and implementing new programs and services, with emphasis on research, scholarship, and evidence-based solutions to address the libraries' growing needs.

Keywords: Distributed learning, academic partnerships, organizational study, multi-campus institution, team-based librarianship

Poster Number: 51

Time: Sunday, May 20, 2:00 PM – 2:55 PM

Different Research: Different Searches

Wichor M. Bramer, Biomedical Information Specialist, Medical Library, Medical Library, Rotterdam, Zuid-Holland, Netherlands; **Patricia F. Anderson**, Emerging Technologies Informationist, Taubman Health Sciences Library, Taubman Health Sciences Library, Ann Arbor, MI; **Tracy C. Shields, AHIP**, Reference Medical Librarian, Naval Medical Center Portsmouth, Naval Medical Center Portsmouth (Navy), Portsmouth, VA

Objectives: The daily practice of medical information specialists sees a wide variety of requests for librarian-mediated searches. The focus in current research on search methods focuses on the systematic review, a small fraction of the requests. What different types of requests can we identify and what are the most effective ways to fullfill these information needs.

Methods: We sent out a structured online questionnaire to various mailing lists to identify different types of information needs that medical information specialists observe in their daily pratice. We also investigate the challenges they experience and the solutions they found when serving those customer needs. The outcomes are combined with the expertise of the authors and evidence from the literature to create solutions for the different information needs.

Results: So far we have identified
- Clinical care: Direct questions from patients or health care providers, which often require a fast answer
- Educational assignments: the focus is often on the educational achievement of the student, not the quality of the search
- Research support: for grants the question often is whether a certain research has already been performed
- Publication support: Systematic reviews, narrative reviews, guideline searches
- Outreach: Health literacy and patient information aimed at the general public

Conclusions: Important differences are the time invested, and the optimization parameters. For clinical questions the number of hits should be low, therefore precision is optimized, which means a loss of recall. For systematic review searches sensitivity is optimized which means a low precision. There are differences in the level of evidence used and the number and type of databases required. However, all searches, including those for systematic reviews, though they are different in nature and purpose, can be seen as part of a continuum.

Keywords: expertsearching, databases, systematic reviews

Poster Number: 52

Time: Monday, May 21, 2:30 PM – 3:25 PM

Librarians Codevelop and Team Teach Evidence-Based Practice (EBP) Workshops with Hospital EBP Council

Don P. Jason, III, Health Informationist, Donald C. Harrison Health Sciences Library, Cincinnati, OH; **Emily B. Kean**, Research and Education Librarian, University of Cincinnati, Donald C. Harrison Health Sciences Library, Cincinnati, OH; **Katie Lang**, Physical Therapy Program Coordinator, Evidence Based Practice Council Chair, University of Cincinnati Medical Center, Cincinnati, OH; **Joyce J. Zehler**, Staff RN at the Center for Emergency Care, University of Cincinnati Medical Center, Cincinnati, Ohio, Blanchester, OH; **Kathy Moller, RN, BSN , CGRN** , University of Cincinnati Medical Center , Cincinnati , OH

Objectives: Librarians joined the Evidence Based Practice (EBP) Council of an academic medical center and collaboratively developed an EBP workshop. The aim of the workshop was to increase nurses' comfort level with EBP principles so that they would choose to serve as EBP liaisons. These liaisons mentor and evaluate peers that are conducting outcomes based projects for the clinical ladder program.

Methods: The two-hour course was developed in early summer of 2017 and pilot tested twice in August of 2017. 13 nurses signed up for (and attended) the initial workshops. Before the class began a pretest survey was distributed to attendees to assess their current comfort level with EBP principles. The survey featured a Likert scale with choices 1 (Not at all) through 10 (Always). Then librarians taught modules on: developing a clinical PICO question, conducting a literature review, and distinguishing between the different types of research. Afterwards nurses taught modules on the forms that are used to document outcomes based projects. They also taught a module on the rubric used to evaluate outcomes based projects. A posttest survey was distributed to all workshop participants to assess their knowledge and comfort with EBP principles.

Results: Comparing the attendees' responses from the pretest and posttest surveys demonstrated considerable positive impact.

Question 1 asserts "I am familiar with resources available at the hospital for PICO question development." The average pretest score was 5.08 and the posttest score was 8.92. A 76% improvement.

Question 2 asserts "I am comfortable reviewing literature and assisting others with literature searches." The average pretest score was 4.31 and the posttest score was 8.15. An 89% improvement.

Question 3 asserts "I can distinguish between research and non-research sources." The average pretest score was 5.69 and the posttest score was 8.46. A 49% improvement.

Conclusions: The short term goal of the workshop was to recruit EBP liaisons. In the long term, it is hoped that the workshop will improve the quality of the outcomes based projects being submitted by nurses. It will also create a hospital culture that is more comfortable with EBP principles and concepts. The EBP workshop is now offered on a monthly basis in the Health Sciences Library. The pretest and posttest survey assessment is ongoing. The progress towards the short term and long term goals will be measured as more data is collected and analyzed.

Keywords: Evidence Based Practice, Pedagogy, Teaching, Survey, Instruction, Collaboration, Assessment, Evaluation

Poster Number: 53

Time: Tuesday, May 22, 1:00 PM – 1:55 PM

Building an Assistive Technology Workspace

JJ Pionke, Assistant Professor and Applied Health Sciences Librarian, Social Science, Health, and Education Library at the University of Illinois Urbana Champaign, Social Sciences, Health, and Education Library at UIUC, Champaign, IL

Objectives: This presentation focuses on the creation of an assistive technology lab from conception through the development of the lab. Included is a discussion of the arguments for building a lab in a time of cash strapped budgets as well as what basic items to include.

Methods: Due to growing awareness of accessibility concerns and the need to be more inclusive for all people, the decision was made to create an assistive technology lab. The project has a variety of phases include conceptualization, development, and implementation. The conceptualization phase involved research into what other institutions have developed in terms of assistive technology labs. The development phase focused on research into what was needed, including interviews with students with disabilities, as well as creating an education program for library employees. The implementation phase involved the actual creation of the lab and it’s promotion and marketing.

Results: To increase accessibility to the resources that the library has by creating an assistive technology lab that promotes accessibility and inclusion. This project also involved educating library employees about the needs of people with disabilities.

Keywords: disability, assistive technology, user services, accessibility, inclusion

Poster Number: 54

Time: Tuesday, May 22, 2:00 PM – 2:55 PM

Getting Attention: How We Used an Anniversary to Promote the Library to Our Hospital System and Beyond

Dawn Melberg, Manager, Library Services, North Bay/South Bay, Health Sciences Library, Health Sciences Library, Santa Rosa, CA; **Marina Aiello**, Mgr of Library Services, Tech and Instr Design, NCAL Libraries, Kaiser Permanente Libraries, Stockton, CA; **Beverly McLeod**, Manager of Library Services, Health Sciences Library, Santa Clara, CA; **Margaret (Peggy) Makie, AHIP**, Manager of Library Services, Kaiser Permanente, Health Sciences Library, Roseville, CA; **Eve Melton, AHIP**, Regional Director Library Services, Northern California, Northen California Region, Kaiser Permanente Libraries, Stockton, CA

Objectives: To raise awareness of our librarians and library services across four organizational regions and eight US states. Our library system comprises three dozen health sciences librarians at hospitals and medical centers in three states. Our continual objective as a network is to inform clinicians, administrators, and other employees about the existence of the library and its potential benefit to them.

Methods: We took advantage of a significant anniversary - 70+ years of librarian services - to reach a large and geographically dispersed employee base. Celebrating our anniversary gave us a “stage” from which to speak with a cohesive voice to various parts of our organization. It also provided an opportunity to elevate the status of the library and expand our role as librarians.

Our multi-pronged approach:
• Get on Stage: Make our librarians more visible online and in person
• Strut our Stuff: Create special celebration logos and use them widely
• Showcase our Talent: Share our annual report infographic
• Lean In: Develop a 70-year timeline of company and librarianship history
• Sing our own Praises: Publicize our anniversary to internal and external audiences
• Make New Friends: Forge alliances with internal departments
• Keep the Old: Recognize loyal customers

Results: During the celebration, we conveyed the dual messages that the library has been integral to the organization since its founding, and that librarians are essential players in providing high-quality patient care. Even after the close of our anniversary year, we expect long-lasting benefits: The library is more “findable” because dozens of company intranet sites now feature “Library” as a department and a search term; our professionals now “look like” clinical and administrative personnel by wearing “Librarian” badges; goodwill has been generated by our acknowledging “super patrons” with personal letters of appreciation; and our timeline makes us part of company history.

Conclusions: The usual purpose of publicity is to attract new customers. Our library system already enjoys a healthy volume of business; we handle more than 1,500 requests each month. But usage is not the same as advocacy. We may be “heroes” to our users but unfamiliar to large parts of the organization. While the immediate goal of the anniversary project was to promote the library, its long-term objective was to raise awareness among employees - even those who have never used library services - that having a medical library is a valuable asset and point of pride for our entire company**.**

Keywords: marketing, publicity, promotion, advocacy, timeline, history, hospital, anniversary, celebration

Poster Number: 55

Time: Sunday, May 20, 2:00 PM – 2:55 PM

Digital Tools for Managing Different Steps of the Systematic Review Process

Wendy Wu, Librarian IV, Wayne State University, Shiffman Medical Library, Detroit, MI; **Katherine G. Akers**, Biomedical Research and Data Specialist, Shiffman Medical Library, Detroit, MI; **Ella Hu**, Biomedical Sciences Reference & Research Informationist, Wayne State University, Shiffman Medical Library, Detroit, MI; **Alexandra Sarkozy**, Science Librarian, Purdy Kresge Library, Purdy-Kresge Library, Detroit, MI; **Patricia Vinson**, PT Librarian 1, Wayne State University, Shiffman Medical Library, Detroit, MI

Objectives: The systematic review process can be facilitated by the use of digital tools and software to perform article screening, data extraction and analysis, risk of bias assessment, and other tasks. Here, we describe and categorize currently available digital tools for managing different steps of the systematic review process to help reviewers identify the tool(s) that best fits their needs.

Methods: We will first compile a comprehensive list of all currently available software and digital tools for managing steps of systematic review and meta-analysis processes (e.g., Abstrackr, Covidence, DistillerSR, Rayyan, Systematic Review Data Repository, RevMan, OpenMeta[Analyst], EPPI-Reviewer, DRAGON). We will then map the functionality of each tool onto the steps of the systematic review and meta-analysis processes with the aim of distinguishing categories of tools with similar functionalities. We will also prepare up-to-date summaries of the different tools, including their cost, extent of documentation and technical support, and limitations.

Conclusions: With the ever-increasing number of digital tools and software available for managing different steps of the systematic review process, it can be difficult for systematic reviewers to make fully informed decisions about which tool(s) to use. The results of our analysis are expected to update health information professionals' knowledge of the currently available systematic review tools and provide a framework for helping systematic reviewers select the most appropriate tool(s) for enabling knowledge synthesis.

Keywords: systematic reviews

Poster Number: 56

Time: Tuesday, May 22, 1:00 PM – 1:55 PM

Risky Business: Medical Humanities Programming Pays Off at a New Medical School

Kathryn Houk, AHIP, Health Sciences Librarian, University of Nevada, Las Vegas, UNLV Health Sciences Library, Las Vegas, NV; **Bredny Rodriguez, AHIP**, Health Sciences Librarian, Health Sciences Library, Health Sciences Library, Las Vegas, NV; **Johan Bester**, Director of Bioethics & Assistant Professor, Family Medicine, University of Las Vegas Nevada School of Medicine; **Anne Weisman**, Director of Wellness & Integrative Medicine, University of Las Vegas Nevada School of Medicine

Objectives: This poster describes the first steps in creating collections and providing programming around medical humanities; within and outside of the undergraduate medical curriculum at a new medical school and health sciences library.

Methods: Setting/Population: Library and teaching faculty at a newly developed medical school and health sciences library in the western United States have collaborated to pilot various initiatives on a health campus for medical students, faculty, staff and allied health professionals.

Description: A small group of faculty on the health campus with interest in integrating the arts and humanities into medical education and healthcare practice formed in the medical school’s inaugural year. First projects envisioned by the group include building local collections, hosting lunch discussions, providing instruction on creating medical comics, and hosting workshops or speakers on social and cultural factors that impact the current state of health and healthcare in the state. The group has received support from administration in the school of medicine and health sciences library to pursue these activities.

Conclusions: Evaluation Methods: Participation counts at events, tracking the use of comics for reflective assignments, evaluate circulation and use statistics for collection materials, and brief surveys after events have been and will continue to be utilized to determine the popularity and effectiveness of medical humanities programming.

Keywords: medical humanities, medical education, outreach, collection development, programming, community engagement

Poster Number: 57

Time: Tuesday, May 22, 1:00 PM – 1:55 PM

Capacity, Experience, and Recruitment: Hiring Library Sciences Graduate Students in Health Sciences Libraries

Ruby L. Nugent, LIS Graduate Student Assistant, University of Arizona Health Sciences Library, Tucson, AZ

OBJECTIVE:
To describe how employing graduate level library science students in paid staff positions can be a valuable practice that a) gives health science libraries the ability to build staff capacity and assist in completing projects, b) provides graduate students paid employment with the opportunity to gain valuable professional-level experience, and c) is beneficial in the recruitment of students who eventually pursue professional positions in health sciences libraries.

METHODS:
I will provide a comparison of these two academic health sciences libraries and their models for hiring graduate level library students based on personal experience at each institution. First, as the former graduate student supervisor at the University of Colorado (CU) Health Sciences Library and second, as a library student currently enrolled as a Knowledge River scholar and employed as a graduate assistant at the University of Arizona (UA) Health Sciences Library. I will share how these positions were created, recruitment and onboarding procedures, describe student job duties and expectations, and indicate the number of students who obtained employment in a health sciences library after graduation.

RESULTS:
Based on my observations and personal experience, I believe hiring library sciences students for these positions at both institutions is beneficial for the libraries and the students. Students participate in instruction, contribute meaningful project work, and cover service points. Many have presented this work at professional conference. Fifty percent of graduate students at CU and twenty-seven percent at UA have gone on to pursue health sciences in some capacity. Challenges to both models include: time invested in the hiring and onboarding process, obtaining sustainable funding, and frequent turnover. Because students can regard the experience as not generalizable outside medical librarianship, do not have a medical background, or are intimidated by applying for a position in a special library, these positions can be difficult to recruit for.

CONCLUSION:
The health sciences libraries at CU and UA have both been successful at filling graduate assistant positions with library science students. Their unique approaches exhibit practices and procedures which are appropriate for each respective library. Both support opportunities for mentorship and networking through collaboration and project work, providing professional-level experience for the students while increasing staff capacity. Interviewing graduate students about their interests and experiences with health sciences, surveying other health sciences libraries regarding the value of these models, and creating handbooks for employing graduate students could be helpful in developing more formalized models in the future.

Poster Number: 58

Time: Tuesday, May 22, 2:00 PM – 2:55 PM

A Pilot Partnership to Provide Health Information to Cancer Patients and Families

Christian Minter, Community Engagement and Health Literacy Librarian, McGoogan Library of Medicine, Omaha, NE; **Roxanne Cox**, Head, Education & Research Services, University of Nebraska Medical Center, McGoogan Library of Medicine, Omaha, NE; **Rebecca Jackson**, Manager, Patient and Community Engagement, Nebraska Medical Center, Patient Experience, Omaha, NE; **Benjamin Simon**, Library Assistant II, University of Nebraska Medical Center, McGoogan Library of Medicine, Omaha, NE; **Mary Winter**, Library Technician II, University of Nebraska Medical Center, McGoogan Library of Medicine, Omaha, NE

Objectives: McGoogan Library of Medicine at University of Nebraska Medical Center launched a pilot partnership with the new Resource and Wellness Center at Fred and Pamela Buffett Cancer Center in Omaha, NE. The goals of this pilot were to provide patients and families with easy access to quality health information, assess the health information needs of cancer patients and their families, and explore the demand for library services in the resource center.

Methods: From June 2017 through March 2018, library faculty and staff worked daily shifts at the Resource and Wellness Center and answered consumer health questions. Statistics were compiled on the number and type of health information questions received. Librarians also helped select consumer health books and brochures. The library gathered additional feedback through meetings with the Oncology Patient and Family Advisory Council, clinical staff, and health system administrators.

Results: Library faculty and staff spent 656 hours at the Resource and Wellness Center and received thirty-two requests for consumer health information. Most questions were related to a specific type of cancer, resources for coping, and practical issues such as financial assistance for treatments. The resource center now has a small collection of print materials, and an index was created to assist staff with navigating the information materials when a librarian is not present. Meetings with stakeholder groups provided insight on the organizational culture of the cancer center, and recommendations for improving engagement with patients and families. Lessons learned during this pilot will be shared during the poster presentation.

Conclusions: McGoogan Library adds value to the Resource and Wellness Center by providing patients, families, and staff with expertise in identifying and accessing quality health information to support cancer care. Feedback from stakeholders has affirmed the importance of the library's continued involvement in this space. The pilot has helped make librarians more accessible to cancer patients and families and increased the number of consumer health information requests the library receives. Future activities will include creating new library marketing materials specific to cancer patients and health providers, continuing to develop the collection of print materials, implementing an evaluation form to assess patron satisfaction, and launching a pilot tablet-lending program.

Keywords: consumer health information, partnerships, cancer, patients, caregivers

Poster Number: 59

Time: Sunday, May 20, 2:00 PM – 2:55 PM

Does a Discovery Search Product Increase Use of E-Books and E-Journals

Sara Pimental, AHIP, Senior Consultant, Care Management Institute, Care Management Institute, PETALUMA, CA; **Eve Melton, AHIP**, Regional Director Library Services, Northern California, Northern California Region, Kaiser Permanente Libraries, Stockton, CA

Objectives: Prior to the introduction of a discovery tool, low usage of ebooks and ejournals from various disparate platforms was of concern to leadership at our large hospital library system. A decision was made to implement a discovery tool in 2015. Evaluation of increased usage of ebook and e-journal resources is of significant interest to our leadership and librarians.

Methods: We will look at usage reports from our ebooks and ejournals vendors from before and after the purchase of the discovery product to see if usage has increased. We also looked at cost of the Discovery product compared with its use to determine ROI. A comparison of number of searches leading to retrieval of full-text will suggest the value and success of searches performed in the new discovery system**.**

Results: We felt we could only compare numbers within the Discovery product, not total usage for all full text. From 2016 - 2017 use of full text dropped 26% and sessions dropped 19% with the Discovery product. However in 2015 interlibrary loans generated from searches performed on the product went up 1,878%.

Conclusions: Even though use of full text didn't go up and the rise in interlibrary loans was potentially costing us money, sessions were only costing about 25 cents per session. Discovery search appears to be attracting patrons that are traditionally difficult to attract to library services such as nurses, allied health staff and, the biggest surprise, leadership, we did consider that the Discovery tool did return a satisfactory ROI.

Keywords: search, discovery, ebooks, ejournals, hospital

Poster Number: 60

Time: Monday, May 21, 2:30 PM – 3:25 PM

Assessment on the Usage of E-Books in Academic Health Sciences Libraries in Taiwan

Estelle Hu, AHIP, Librarian V, John H Stroger Hospital of Cook County, John H Stroger Hospital of Cook County, Chicago, IL; **Shih Nuan Lin**, University Librarian, China Medical University, China Medical University Library, Taichung, Taichung, Taiwan (Republic of China); **Chien Ju Chou, Head**, National Yang-Ming University, Library/Reference Services, Taipei, Taipei, Taiwan (Republic of China); **Jui-Wen Chen**, Head, Taipei Medical University, Library/Technical Services, Taipei, Taipei, Taiwan (Republic of China)

Objectives: E-book usage in academic health sciences libraries in Taiwan has received little attention in published literature. This research project will analyze the e-book usage data from three academic health sciences libraries in Taiwan. This study will show the importance of analysis on usage of e-books and expect to bring more research on this topic.

Methods: Taiwan has 12 academic health sciences libraries located within four regions. Eight libraries from four regions were approached. Three libraries from two regions agreed to participate in the study. Usage data from 19 e-book packages was collected from January 2014 to December 2016. Four packages were licensed as rental models among all three libraries. The other packages were purchased with perpetual access by one or more of the libraries. The total number of students from each university was collected during the study period. Each e-book package was analyzed by individual titles' usage. Usage of e-books from different editions were counted as one title. The top 20 most highly used titles were identified on the same current subscription packages in each library and among all three libraries. The analysis will also highlight popular subject areas and the months with the highest usage during the academic year.

Results: Three of the subscribed e-book packages' average usage was a few thousand except one package with less than 100 titles had 4-digit annual usage. Two libraries that subscribed to the same package had the same top 2 used titles. Within this package, the same 10 titles were included within the 20 highly used titles for both libraries. Another package subscribed by all three libraries had the same top 2 used titles, and 7 of the 20 most highly used titles were included from all three libraries. Two purchased packages had much higher usage than the others. The overall usage of one library with the most students' body was comparably lower than the other two libraries. Months with the highest usage occurred before or during midterms, or finals for all three libraries. Medical subject is highly used in all 3 libraries.

Conclusions: The usage of the rental packages is much better than that of the purchased packages. Two libraries had decreasing annual usage on the packages with current subscription. Library staff might want to promote library collection more to have higher usage of library resources.

Poster Number: 61

Time: Tuesday, May 22, 1:00 PM – 1:55 PM

Leading Students to the Library with Search Services

Katie A. Prentice, AHIP, Associate Director for User Experience and Assessment, Schusterman Library, Schusterman Library, Tulsa, OK

Objectives: This paper will examine multi-year trends showing a large increase in use of the library's mediated search service to measure utilization by campus population and subject area and show how the service connects users to other library services.

Methods: At a small academic library, the librarians complete mediated search requests for students, faculty, residents, and staff submitted through an online form. This project will examine three fiscal years of submissions and completion reports through exploratory analysis to determine trends and information about who is most often using the search service and analyze the themes of the requests.

Results: Analysis of the nearly 600 submissions from three years reveals an increase in use from each year to the next. Annual data show that the highest utilization of this service comes from medical residents. The second highest use is from social work students. On a campus that offers 35 degree programs, 66% of degree programs show use of this service and when counting only on-campus programs 92% of programs show use. The most common purpose for requesting a search was research followed by assignments.

Conclusions: For a small library staff, an active and robust literature search service is possible and can be popular with students, faculty, residents, and staff. On a campus that offers 30 degree programs the requests come from all areas of study. Through this process we can refer to other library services and support campus scholarship.

Keywords: mediated searching, library services, connections

Poster Number: 62

Time: Tuesday, May 22, 2:00 PM – 2:55 PM

Identifying Information Needs to Facilitate Assessment and Development of Opioid Epidemic Treatments and Prevention Programs in New Hampshire

Eugenia Liu, Health and Human Services Librarian, University of New Hampshire, Dimond Library, Durham, NH

Objectives: Prescription opioid misuse in New Hampshire is among the highest in the nation. While many publications exist describing opioid prevention programs and treatments, there is a lack of research examining treatment assessment and development. This pilot project aims to identify the information resources that healthcare and mental health professionals currently use for opioid research, what information resources are needed to asses and develop prevention programs and treatments for opioid dependency, and what barriers exist to accessing those resources.

Methods: The principal investigator identified and created lists of healthcare, substance use disorder (SUD) treatment facilities, mental health, and counseling organizations in Strafford County, NH and reached out to Strafford County Service Link, a NH Department of Health and Human Services program that connects individuals with local health and human services agencies.

A 13-item online questionnaire was created through the Qualtrics software and submitted to the University of New Hampshire's Survey Center to enhance clarity and improve the logical progression of the questionnaire. Questionnaire items collected demographic information, and identified information resources that are currently used for opioid-related research. The questionnaire also identified needed information resources and barriers to accessing those resources.

The questionnaire was distributed as a short bit.ly link via emails, phone calls, and through Service Link to the identified healthcare, SUD treatment facilities, mental health, and counseling organizations in Strafford County, NH.

Results: Participant responses (n=5) indicated their roles in healthcare, SUD prevention coordination, and counseling services, as well as the type of opioid-related research that they conduct including SUD treatment assessment, prevention program assessment, prevention program development, and patient care. Some participants have access to the CINAHL and UpToDate databases, but use the CDC's website and PubMed for opioid-related research. Barriers to accessing resources for opioid-related research include lack of database training, lack of funding to purchase database access, lack of familiarity with databases, and the perceived complexity of the research tools.

Conclusions: This pilot project identified a small sample of healthcare and mental health professionals who conduct opioid-related research using information resources that are freely available online. Research using databases like CINAHL and PsycInfo appear to be limited due to lack of financial resources as well as lack of familiarity with the tools. This project should be expanded to obtain responses from a larger population in order to paint a fuller picture of information resources used for opioid-related research.

Keywords: Opioid, substance use disorder, treatment, assessment, prevention programs, questionnaire, survey

Poster Number: 63

Time: Sunday, May 20, 2:00 PM – 2:55 PM

Doing It Right: Assessing the Impact of a Multi-Day Systematic Review Retreat for Researchers

Lynn Kysh, Clinical & Research Librarian, Norris Medical Library, Norris Medical Library, Los Angeles, CA; Robert Johnson, Clinical & Research Librarian, Norris Medical Library, Los Angeles, CA; **Rikke Sarah. Ogawa, AHIP**, Director, Louise M. Darling Biomedical Library and Science and Engineering Library, Louise M. Darling Biomedical Library, UCLA, Los Angeles, CA; **Melissa L. Rethlefsen, AHIP**, Interim Executive Director & Librarian, University of Utah, Spencer S. Eccles Health Sciences Library, Salt Lake City, UT

Objectives: Systematic review interventions often focus on training librarians to engage with a systematic review team. Instead, a course was designed to assess the impact of an Institute for Museum and Library Services (IMLS) grant-funded four day systematic review hands-on retreat on a group of multidisciplinary researchers in conducting systematic reviews in accordance to the PRISMA statement.

Methods: A team of librarians and epidemiology faculty received an IMLS grant to design and implement a four-day systematic review retreat. Ten researchers were selected through an application process to participate in the curriculum which mixed lecture and software training with breakout sessions allowing participants to work on their project and benefit from peer-review. Participants arrived with an identified research question and left the retreat with a protocol draft, a preliminary PubMed search strategy, and a decision of whether or not to include meta-analysis methods in their project. With IRB approval, assessment of the retreat has been and will continue to be capture through pre-survey followed by post surveys completed on the last day of the retreat and at the three, six, and nine month mark following. An hour long interview will be conducted at the six-month mark among willing participants.

Results: Preliminary results demonstrate a shift of intention in a majority of the participants to conduct a scoping review rather than a systematic review which indicates a broader need for education in selecting the review methodology most appropriate for a research question. Initial pre/post-surveys demonstrate a trend of self-reported learning which may potentially impact participants' future research efforts in systematic reviews, how they mentor young researchers in the process, and finally in how they appraise and apply systematic reviews in their practical work.

Conclusions: From an analysis from the fully collected data we hope to determine whether or not a four day workshop generates high quality scoping and systematic reviews from participants. We also intend to use participant feedback to adjust the curriculum and logistics of the workshop for future offerings.

Keywords: systematic reviews, curriculum, instruction, researchers

Poster Number: 64

Time: Monday, May 21, 2:30 PM – 3:25 PM

New Psychoactive Substances (NPS): An Exploratory Study on the Forum Psychoactif to Characterize New Drugs, New Consumption Patterns and User Profiles

Fossey Solenn, Student in information science, UMR912-SESSTIM-INSERM-IRD-AMU, santerCom team-UMR 912- SESSTIM, Marseille, Provence-Alpes-Cote d'Azur, France; **Briand-Madrid Laelia**, Engineer, UMR912-SESSTIM-INSERM-IRD-AMU, ESSEM Team, Marseille, Provence-Alpes-Cote d'Azur, France; **Spire Bruno**, Researcher in public health, UMR912-SESSTIM-INSERM-IRD-AMU, ESSEM team, Marseille, Provence-Alpes-Cote d'Azur, France; **Patrizia Carrieri**, Researcher in public health, UMR912-ISESSTIM-INSERM-IRD-AMU, ESSEM Team, Marseille, Provence-Alpes-Cote d'Azur, France; Roux Perrine, Researcher in public health, UMR912-SESSTIM-INSERM-IRD-AMU, ESSEM Team, Marseille, Provence-Alpes-Cote d'Azur, France; **Tanti Marc**, Head of research projects in information sciences , CESPA-UMR912-SESSTIM-INSERM-IRD-AMU, SanterCom team-CESPA, Aix Marseille Univ, INSERM, IRD, SESSTIM, Sciences Economiques & Sociales de la Santé & Traitement de l’Information Médicale, France, Marseille, Provence-Alpes-Cote d'Azur, France

Objectives: New psychoactive substances (NPS) are substances that mimic the effects of known illicit drugs but also have their own psychoactive effects. They are a real public health problem because they are easily produced. The objective is to determine NPS consumption patterns, the profile of users and their health implications by analyzing forum conversations on a community-based website called Psychoactif.

Methods: Conversations on NPS were extracted from the forum of PsychoActif website (www.psychoactif.org). We selected the conversations published from May 2016 to June 2017. Only the exchanges under the headings "Research Chemical" and "Risk Reduction Council" were analyzed.The "Risk reduction consulting" section consists of 3 sub-forums: Consulting on deep web, Consumer equipment and Shooting room, with a total of 931 conversations studied during the analysis period. The "Research Chemical" section also offers 3 sub-forums: Synthetic Cannabinoids, Cathinones and Psychedelic Research Chemicals. A total of 910 conversations were analyzed in this section. The analysis of all the data collected was qualitative (analysis of the discourse and analysis of the contents) and statistical (under Excel®).

Results: The study found a high percentage of drug users publishing on the forum (81.47%), particularly males (74.56%). They are active young people mainly between 22 and 35 years old, employed for half of them. There are different places for consumption: home, concerts, festivals. At 66.22%, the use is for fun, curiosity or transgression. Consumers appear to be polydrug users. Of the 67 NPS identified, the main NPS used were ethylphenidate, méphédrone, 5F-AKB28, 2C-B and 1P-LSD. Ethylphenidate is a stimulant drug consumed by nasal route/injection reported causing irreversible cardiac effects. 2C-B is a psychedelic drug that causes significant hallucinations and isolation.

Conclusions: Our study demonstrated the multitude of NPS currently consumed. We have noted on the forum 67 new drugs. Our analysis also revealed a poly-consumption, especially in young males in search of playful experiences or transgressions. For instance, the monitoring of ethylphenidate seems to be important due to its health and social impacts. Further studies should be conducted on other forums and to monitor the Dark Net, a source of hidden information about NPS in order to provide harm reduction messages to drug users.

Keywords: Discussion forum, New Psychoactive Substance, Psychoactif, harm reduction, risk practices

Poster Number: 65

Time: Tuesday, May 22, 1:00 PM – 1:55 PM

Leading the Way: Evaluating Health Information Professionals' Satisfaction with Discovery Systems

Marilyn G. Teolis, AHIP, Clinical Medical Librarian, James A. Haley Veterans' Hospital, Library Service (142D), Tampa, FL; **Priscilla L. Stephenson, AHIP**, Chief, Library Service, Library Service, Library Service, Tampa, FL; **Mary Virginia Taylor**, Retired Librarian, Overton Brooks VA Medical Center, Library, Shreveport, LA; **Edward J. Poletti, AHIP**, Chief, Learning Resources, Health Sciences Library, Learning Resources, Little Rock, AR

Objectives: Libraries are using discovery services to provide users with a one-box search engine to consolidate library content using a centralized index. The literature discusses use in academic libraries, but there is little data on usage in hospital libraries. We evaluated the experiences and satisfaction of hospital library staff concerning the purchase, implementation, and maintenance of a discovery service.

Methods: This study is based on a previous survey from 2015. An online questionnaire was sent to various health library mail groups, requesting participating librarians' level of satisfaction regarding discovery products. Participants were queried regarding which discovery products they had evaluated and whether a discovery product was purchased. Respondents rated specific features, their satisfaction, and whether the tool met their expectations as a single search box resource.
We inquired about the factors which influenced their purchasing decisions. Individuals who purchased a discovery service were asked if the time to implement and maintain the product was what they had anticipated. Participants were given the opportunity to share lessons learned during the process.
Finally, we invited health information professionals to share their experiences and questioned respondents regarding their perceptions of user acceptance and usage.

Results: Over 87% of the responding librarians were satisfied with discovery services. In the current study, 92% of respondents said they planned to purchase or had purchased a discovery product, in comparison with only 41% of health information professionals who reported purchases or purchase plans in the 2015 study. Many participants said discovery services not only increased utilization of the libraries' owned resources, but also enabled patrons to find underutilized resources they might not have located without a discovery service. Academic librarians said discovery services were essential to their libraries, because they integrated resources with link resolvers, and they revealed doctoral dissertations and other information in their collections which led to increased usage.

Conclusions: We anticipated librarians' satisfaction would increase as discovery services evolved, but we didn't expect the results would show a such a large, positive shift in librarians' attitudes regarding the purchase and utilization of discovery tools.
Future research efforts should focus on several barriers librarians mentioned, such as enabling discovery tools for mobile devices, improving the platform to work seamlessly with different vendors products; refining search filters to find the most specific, relevant, and high-quality resources; and simplifying maintenance and updating procedures.

Keywords: Discovery service, Hospital librarians, Survey, Research

Poster Number: 67

Time: Sunday, May 20, 2:00 PM – 2:55 PM

DynaMed Plus and UpToDate: Which Resource Do Medical School Libraries Subscribe To?

Carolann L. Curry, Reference & Outreach Librarian; Alisha Howard, Interlibrary Loan Assistant; Skelton Medical Library, Macon, GA

Objectives: DynaMed Plus® and UpToDate® are both evidence-based point-of-care clinical decision support tools available for purchase to institutions and individuals. The goal of this research was to examine which (or both) of these resources LCME-supporting health sciences libraries subscribe to.

Methods: Only libraries who provide services to Liaison Committee on Medical Education (LCME) accredited programs in the U.S. were included in this study. For a list of these schools, the authors consulted the directory posted to the LCME website (current as of August 30, 2017). Full, provisional, preliminary, and probationary accreditation statuses were included. Authors then performed a comparative analysis of each library website that supported an LCME curriculum. During website examination, authors paid particular attention to "Databases" and "Popular Resources" sections of library websites to determine current subscriptions to DynaMed Plus or UpToDate resources. For libraries that didn't clearly list their electronic resource holdings, the authors emailed those libraries directly to inquire about DynaMed Plus and UpToDate subscriptions. Data collection spanned from September 2017 to April 2018.

Results: As of August 30, 2017, there are 147 LCME programs within the U.S. Of these institutions, there are a total of 151 libraries supporting LCME schools. One library did not list their resources on their website and did not respond to authors' emails so they were omitted from this study. The majority of libraries (n=144) subscribe to at least one resource with only 6 libraries reporting that they subscribe to neither DynaMed Plus or UpToDate. Over a third (n=56) of libraries reported subscribing to both resources. Overall, UpToDate was more commonly subscribed to (n=112) while DynaMed Plus was subscribed to at 44% (n=88). Of those libraries that only subscribe to one resource (n=88), UpToDate was the most commonly subscribed to (n=56) with DynaMed Plus being subscribed to by 36% (n=32).

Conclusions: Libraries overwhelmingly subscribe to at least one resource, reinforcing the importance of evidence-based, point-of-care resources to the curriculum of LCME schools. UpToDate remains the most commonly subscribed resource when compared to DynaMed Plus. Surprisingly, over a third of LCME libraries reported subscribing to both resources.

Keywords: Point-of-Care Resources, DynaMed Plus, UpToDate, Academic Medical Library, Medical School

Poster Number: 68

Time: Monday, May 21, 2:30 PM – 3:25 PM

Transforming Anatomy Education Using Virtual Anatomy Table Services at the Library

Alexandria C. Quesenberry, Assistant Professor/Research & Learning Services Librarian, University of Tennessee Health Science Center, Health Sciences Library, MEMPHIS, TN; **Caroline Fan**, Assistant Professor/Web Services Librarian, University of Tennessee Health Science Center, The University of Tennessee Health Science Center/Health Sciences Library, Memphis, TN; **Rick L. Fought, AHIP**, Director, University of Tennessee Health Science Center, Health Sciences Library, Memphis, TN; **Gwendolyn D. Jackson**, Associate Professor and Sr. RLS Librarian, University of Tennessee Health Science Center, Health Sciences Library, Memphis, TN; **Jennifer M. Langford**, Assistant Professor/Archivist and Special Collections Librarian, University of Tennessee Health Science Center, Health Sciences Library, Memphis, TN; **Randall Watts, AHIP**, Associate Director/Associate Professor, Health Sciences Library, Memphis, TN; **Lin Wu, AHIP**, Assistant Director/Associate Professor, Research & Learning Services, University of Tennessee Health Science Center, Health Sciences Library, Memphis, TN; **Wesley Holloway**, Assistant Professor, Research & Learning Services Librarian, University of Tennessee Health Science Center, Health Sciences Library, Memphis, TN

Objectives: In order to incorporate the Anatomage virtual anatomy dissection table into library services at a health sciences academic library, a library taskforce built relationships to secure funding, developed usage policies, created an online booking system, and curated an exhibit to feature in the Anatomage table room. To promote and monitor utilization, a training class was offered and usage statistics recorded.

Methods: The addition of the Anatomage table and table services to the library took place over several stages. To secure funding, the library partnered with the student government to purchase the table. Once administration identified a space for the table, the library archivist curated a small exhibit of images from the library's most significant historical anatomy texts to complement the room. After reviewing policies and guidelines for similar technologies at other institutions, the Research and Learning Services department developed usage policies and a training program unique to the Anatomage table.

The library staff encouraged use of the table by inviting stakeholders from the campus to attend a demonstration and designing a customized online booking system using LibCal for ease of access. Data was collected through the online booking system and training schedule to illustrate the user reach of the table.

Results: The partnership between the library and student government to fund the table led to further collaborative efforts to promote the use of the resource. The exhibit of images created an inviting and attractive study space while the table is being utilized. Usage policies and booking guidelines helped to make the implementation of the new resource more organized and accessible to all users. Usage statistics will help the library to devise future plans for the table.

Table training and usage statistics are in the recording process and will be completed by the time of the meeting.

Conclusions: Collaboration was the most important factor for all aspects of this project. The positive relationship between the library and the student government was significant in the ability to bring the Anatomage table to the library. All of the library departments played a role in bringing the table to the library and promoting its services to users. The library will continue to analyze usage data and make future plans on how to collaborate with campus stakeholders to continue to transform anatomy education with the Anatomage virtual anatomy table.

Keywords: Anatomage Virtual Anatomy transform education collaboration planning implementation training policy

Poster Number: 69

Time: Monday, May 21, 2:30 PM – 3:25 PM

Librarians as Methodological Peer Reviewers for Journal Systematic Review Submissions

Holly K. Grossetta Nardini, Associate Director, Yale University, Cushing/Whitney Medical Library, New Haven, CT; **Fanny Duprilot**, Assistant to the Head, Bibliothèque des Grands Moulins, Collection Development and Management, Paris, Ile-de-France, France; **Kate Nyhan**, Research and Education Librarian, Yale University, Cushing/Whitney Medical Library, New Haven, CT; **Rolando Garcia Milian**, Biomedical Research Support Librarian, Yale University, Cushing/Whitney Medical Library, New Haven, CT; **Lei Wang**, Assistant Director, Technology and Innovation Services, Yale University, Cushing/Whitney Medical Library, New Haven, CT; **Judy M. Spak**, Assistant Director, Research and Education Services, Yale School of Medicine, Cushing/Whitney Medical Library, New Haven, CT; **Melissa Funaro**, Clinical Librarian, Cushing/Whitney Medical Library, Cushing/Whitney Medical Library, New Haven, CT; **Janene Batten**, Nursing Librarian, Yale University, Cushing/Whitney Medical Library, New Haven, CT; **Janis Glover**, Research and Education Librarian, Cushing/Whitney Medical Library, Cushing/Whitney Medical Library, New Haven, CT

Objectives: This study investigated librarian involvement in the peer review process of biomedical journals. Specifically, how frequently are librarians asked to review the methodologies of systematic review manuscripts? Librarians and information professionals have significant skills to bring to peer reviewing the search methodologies of systematic reviews (SRs) and meta-analyses (MAs) submitted to journals. We hypothesize that they could play a more significant role in peer review. Increased librarian involvement in peer review could lead to improved searches and greater compliance with recommended standards.

Methods: A Qualtrics survey was developed to capture current experiences of medical librarians involved in the peer review process of SR submissions to journals. The 16-question survey was pilot tested with a group of medical librarians and reviewed by a methodological expert. The survey was distributed through a variety of listservs in March 2018.

Results: There were 300 acceptable surveys.

Preliminary results from the survey indicated that 22% (n=63) of the respondents had been asked by a journal editor to peer review SR or MA manuscripts. Of those who replied that they had not already been asked, 42% (n=122) would peer review and 32% (n=93) might peer review a manuscript. Only 3% (n=9) would not review a manuscript if asked. The median number of SR or MA manuscripts that respondents had reviewed was 4 and the range was from 1 to 40.
Respondents peer reviewed manuscripts for 38 unique journals and the journal title most frequently mentioned was PLoS One.

Most respondents (n=31) knew why they were asked to peer review. The most frequent reason given was because of their professional expertise. Other reasons included referral by a colleague and expertise in the topic area.

Respondents who had ‘rejected or recommended a revision of a manuscript’ based their decision on the ‘search methodology’ (n=36), ‘search write-up’ (n=29), and ‘entire article’ (n=24). Those who selected ‘other’ (n=12) provided a variety of reasons for rejection: the PRISMA flow diagram; tables of included, excluded, and ongoing studies; data abstraction; inconsistent/incomplete reporting; pooling methods; and bias.

Of those respondents who had declined a request to peer review a SR or MA (n=24), the most frequently given reason was ‘not enough time’ (n=12) followed by ‘lack of expertise’ (n=10). Other reasons for declining the request were the journal’s impact factor and a lack of interest.

Keywords: systematic reviews,
peer review,
journal editorial policies,
manuscript submission

Poster Number: 70

Time: Tuesday, May 22, 2:00 PM – 2:55 PM

C2 Pipeline: Librarians Serving High School Students to Promote Health Literacy and Science, Technology, Engineering, Medicine (STEM) Career Path Equity

Alexandra Sarkozy, Science Librarian, Purdy Kresge Library, Purdy-Kresge Library, Detroit, MI

Objectives: Wayne State University health sciences, education, and business librarians worked on an instruction intervention to help Detroit high school students understand health literacy and to help navigate internet and library resources about diabetes. For the program assignments, students worked in groups to create an intervention to raise quality of life for diabetes patients- librarians supported this task.

Methods: Based on instruction during previous years for the same program, librarians revamped a diabetes education instruction session to more closely follow the tasks assigned to student groups during the week-long STEM education summer campus immersion program. Librarians with different areas of expertise collaborated to create an hour-long instruction session for several hundred students that showed them how to search CINAHL, business resources, and internet patient portals to find evidence to support various intervention ideas, as well as listen to patient voices online that expressed diabetes-related needs that could be addressed by an intervention. Both instruction and group work were incorporated into the session, and a feedback form was filled out by each student at the end of the session.

Results: This is the fourth year librarians have provided library instruction for C2 Pipeline summer immersion students. Each year we learn more about student expectations, and how to better meet student information needs. This year we received positive feedback from students about the instruction session, and saw students actively engaged in their group work and using the resources demonstrated. Additionally, librarians developed stronger relationships with C2 Pipeline program administrators and student coaches, and even volunteer mentored a group through the week during the evening. Librarians from different subject areas strengthened working relationships and enjoyed collaborating.

Conclusions: Librarians at Wayne State University will continue to participate in the C2 Pipeline program, and actively seek ways to broaden and deepen our role in this important program.

Keywords: high school students, health equity, diabetes, instruction, collaboration, health literacy

Poster Number: 71

Time: Sunday, May 20, 2:00 PM – 2:55 PM

Embedded with Problem-Based Learner Medical Students

Marita B. Malone, AHIP, Public Services Librarian, SOM Library, Rowan School of Osteopathic Medicine, Stratford, NJ

Objectives: The Rowan School of Osteopathic Medicine librarians have been trying to establish a personal connection to support the Problem-Based Learning Medical Students. This unique population of 32 students in each class are independent learners who are the hardest population for the library to reach as they are less likely to be on campus and use library resources.

Methods: On the first day of school, the Problem-Based Learning (PBL) students were given a thorough library orientation with a visual demonstration of resources. A LibGuide for PBL was created. Two weeks later, after students had worked through their first case, a mandatory library training was held which provided a thorough explanation of resources and students were given a clinical question to answer using these resources. Students were invited to the library to peruse the normally restricted reserve book area of the library. With administrative approval, a medical librarian visited each of the four classes several times during the semester. Notes were taken of students learning issues as they worked through a case. The librarian found reliable resources for these problems and posted them on the PBL learning management system. The faculty provided suggestions for upcoming cases.

Results: The results of this program were immediate. The librarians created personal contacts for the PBL students. PBL students were recognized in the library and around the university. They started using library resources in their presentations and citing their work. Many positive comments were made regarding the vastness and quality of library resources. There was an increased use in the library. When the faculty heard of the mandatory student training, they requested their own training which developed into personalized training sessions for the interested PBL faculty. These sessions will be expanded next year to include the traditional medical student track faculty.

Conclusions: Several factors were key in the success of this program. First, was the positive attitude of the faculty who welcomed the librarians and the education for themselves and their students into PBL. Second was interaction between the librarians and students. A personal connection was created which resulted in more interaction, not only in the classroom but in the library and even the hallways. Third was the resource education available to the students. Being embedded in the classroom allowed the librarians to anticipate needed resources and have them posted and available on the learning management system.

Keywords: Problem-based learners
medical students
librarians
resources
faculty

Poster Number: 72

Time: Tuesday, May 22, 1:00 PM – 1:55 PM

Pieces of the Professional Puzzle: Systematic Review Competence as Professional Competence

Whitney A. Townsend, Informationist; Patricia F. Anderson, Emerging Technologies Informationist; **Emily C. Ginier**, Informationist; Mark P. MacEachern, Informationist; **Kate M. Saylor**, Informationist; Barbara Lowther. Shipman, Informationist; **Judith E. Smith**, Informationist; Taubman Health Sciences Library, Ann Arbor, MI

Objectives: To map the indicators in the Systematic Review Competencies Framework to the MLA Competencies for Lifelong Learning and Professional Success to identify how competence in systematic review integration contributes to professional competence for health sciences librarians.

Methods: As health sciences librarians continue to engage in activities and projects that require developing new and often project-specific skills, it is important to identify the ways in which these skills may serve as indicators for professional development. Using the Systematic Review Competencies Framework (SRCF) (https://www.ncbi.nlm.nih.gov/pmc/articles/PMC5490706/) published in 2016, a team of health sciences Informationists experienced in systematic reviews mapped the individual SRCF indicators to the basic and expert indicators that make up the MLA Competencies for Lifelong Learning and Professional Success. The team used a mini-Delphi method to come to consensus on the appropriate mapping lines between the two sets of competencies. Once the competencies were mapped, it was possible to identify which areas of the MLA Competencies were most fully developed through systematic review skill-building.

Keywords: competence, competencies, systematic reviews

Poster Number: 73

Time: Sunday, May 20, 2:00 PM – 2:55 PM

Implementing a Scholarly Repository for Faculty Publications

Jeremy Gunnoe, Medical Librarian, Louis Stokes Health Sciences Library - Howard University, Washington, DC; **Fatima M. Mncube-Barnes**, Executive Director, Howard University, Howard University, Arlington, VA; **Celia Maxwell**, Associate Dean for Research, Howard University, College of Medicine, Washington, DC

Objectives: There is a need to centralize faculty publications in the health sciences at our university. Most scholarly publications are assembled and updated in preparation of accreditation or tenure applications. Establishing a publication repository on an on-going basis is more efficient and will celebrate faculty's accomplishments while motivating their effort. Maintaining this repository will expedite scholarly engagement processes.

Methods: Setting: An academic health sciences library serving a college of medicine, a college of pharmacy and a college of nursing and allied health.
Population: Approximately 355 full-time health sciences faculty members.
Methodology: The library implemented a new web-based software repository for faculty publications in the health sciences at our university. Lists of recent publications were provided to the librarian to create faculty profiles as a foundation for a centralized repository. Profiles may then be managed by individual faculty members, with assistance from the librarian. Alerts were created to facilitate the process of continuous updates.

Results: This repository was used as a conduit of improving communication and collaboration between the library and the health sciences colleges regarding scholarly publications. To date, 39% of faculty profiles have been created. The next target is to complete all faculty entries for 2017-2018.

Conclusions: Direct relationships were established with the research departments of each college to address departmental needs. Marketing efforts will be established to inform faculty of this service and engage them in management of their profiles.

Keywords: scholarly communication faculty publication repository publish faculty academic medical library

Poster Number: 74

Time: Tuesday, May 22, 2:00 PM – 2:55 PM

Campaigning for Health: Adapting a Social Media Campaign Process to Reach a Broader Audience

Joelle A. Mornini, Contractor, ICF, National Institute of Health, National Library of Medicine/Outreach and Special Populations Branch, Silver Spring, MD

Objectives: Social media campaigns can be used to share information on a health topic or resource over multiple digital communication platforms and evaluate the impact of the campaign using Google Analytics. This project describes the development of a social media campaign process to share health information with the public, with adaptions made monthly based on lessons from the previous month's campaign.

Methods: Four social media campaigns were conducted to promote online health information resources during May-August 2017, and impact from the campaigns were tracked through Google Analytics. UTM codes created through Google Campaign URL Builder were added to the URLs of the resources shared during each campaign. The Campaigns section of Google Analytics could then be checked to view the number of sessions and new users directed to the online health resource through different social media sources (email bulletin, Facebook, Twitter, and Pinterest). The following elements of the social media campaign process were updated each month, depending on results from the previous campaigns: types of online health resources shared through the campaign, content of the campaign promotional material text and images, partners with whom the campaign promotional material was shared, and social media platforms where the material was shared.

Results: The number of sessions and new users directed to the online health resources through campaign links increased steadily for each campaign, from 141 sessions and 75 new users for the May 2017 campaign to 514 sessions and 401 new users for the August 2017 campaign. Changes made to the campaign process based on Google Analytics data include promotion of targeted online health resources based on monthly health observances, broader use of email bulletins to promote campaign material, creation of multiple image options for promotional materials, and sending campaign promotional materials to partner organizations for broader dissemination of the campaign links.

Conclusions: During this project, the criteria for success was number of sessions and new users directed to online health resources, and changes were made to the campaign process based on the hypothesis that they would bring more sessions and users during the next month's campaign. If the process change did bring more sessions and users in the next month's campaign, then the change was permanently implemented. The continual adaption of to the campaign process based on evaluation using Google Analytics data leads to steady improvement in the number of sessions and new users directed through social media to online health resources.

Keywords: Social media, consumer health, user engagement

Poster Number: 76

Time: Monday, May 21, 2:30 PM – 3:25 PM

Using Data Interviews to Inform Improvements in Research Data Management Infrastructure and Services

Wladimir Labeikovsky, Bioinformationist, Health Sciences Library, University of Colorado Anschutz Medical Campus, Aurora, CO

Objectives: A central challenge for librarians providing Research Data Management Services is the varied set of requirements and practices that each research group follows. Information Technology officers face a similar dilemma in designing their offerings and ensuring they are used effectively. We will conduct detailed one-on-one and group interviews with individual research groups to assess needs and design new services.

Methods: A detailed questionnaire will be prepared in collaboration with the IT office and with other librarians to guide interviews with individual researchers. We will probe different steps in the researcher's workflow and in the research data lifecycle. Interviews will be organized by field and type of research performed to ensure a wide case distribution. Responses will be categorized by urgency and scale of proposed services and solutions and will be used for a) choosing points of contact for librarian RDM consults and b) policy proposals for related campus infrastructure (e.g. storage and computing solutions).

Keywords: data management storage organization best practices outreach education

Poster Number: 77

Time: Tuesday, May 22, 1:00 PM – 1:55 PM

Meeting the Emerging Information Needs of Precision Medicine: Enhancing Health Librarians' Knowledge and Search Skills to Support a New Clinical Service

Rolf Schafer, Manager, Library Services; Julie Williams, Research Services Librarian; **Elle Matthews**, Senior Librarian eServices; St Vincent's Hospital Sydney, Walter McGrath Library, Darlinghurst, New South Wales, Australia

Objectives: Becoming a world leader in the research and clinical applications of precision medicine is a key strategic commitment for St Vincent's Health Network Sydney. This paper describes how we responded to this challenge by building our knowledge of information resources, enhancing our database searching skills and developing a Library Guide to support a new clinical service in precision medicine.

Methods: Firstly we undertook to enhance our knowledge and skills in the field of precision medicine. This was achieved by: undertaking literature reviews on various precision medicine topics; attending several genetics and genomics lectures organised by the newly established Clinical Genomics Unit; consulting with the head of the CGU Unit and clinical geneticists; and finally undertaking e-learning sessions through MLA webinars, NCBI e-tutorials and viewing YouTube videos. Once we had acquired a better understanding of the concepts associated with precision medicine, we focused on enhancing our database searching skills. From identifying relevant MeSH and EmTree terms to genetic filters and searching specialised genomic databases. Finally, we developed a Precision Medicine Library Guide with input from our clinical geneticists. Together we identified genomic databases and resources available from reputable sites.

Results: ***By undertaking various e-learning modules and webinars, reading widely, attending presentations as well as consulting with clinicians, Library staff have increased their knowledge and understanding of the concepts associated with precision and genomic medicine. We are building our collection with precision medicine resources. Our analysis and evaluation of the resources, in consultation with clinicians laid the ground work for our Precision Medicine Library Guide. Evaluation of the usefulness and format of the guide will be undertaken. The ongoing use of specialised genomic databases and search filters has increased librarians' searching skills and confidence in using these resources.***

Conclusions: The Library team decided in 2017 that we needed to enhance our knowledge and search skills to support a new clinical service in precision medicine. The team implemented a plan resulting in the following outcomes:

• increased knowledge of genomic and bioinformatics resources
• increased confidence in searching the specialised databases
• increased awareness and accessibility to key genomic resources via a Precision Medicine Library Guide

In realigning service delivery, the Library's presence at St Vincent's Hospital has been enhanced by introducing new information resources and services to meet the changing needs of the organisation.

Keywords: Precision Medicine, Genomics, Genetics, Information Resources, Database searching, Library Guide.

Poster Number: 78

Time: Tuesday, May 22, 2:00 PM – 2:55 PM

Five Star Health Library in a Four Star Hotel

Adela V. Justice, AHIP, Senior Librarian; **Sarah S. Stone**, Health Education Specialist; The Learning Center, Houston, TX

Objectives: The authors will describe a unique consumer health library in a hotel setting that provides health information to cancer patients and their families.

Methods: This consumer health library is a partnership of 20-plus years between a cancer hospital and a hotel that is attached to the hospital’s main inpatient building. The library is one of three patient education libraries located in the hospital and utilizes the same staff and resources. The library is a self-serve model and is monitored by the hotel’s Patient Guest Relations (PGR) office. PGR staff provides front-line assistance and refers patrons with complex information requests to the two other libraries, which are staffed full-time by librarians and health education specialists. The health education specialist in charge of maintaining this library provides ongoing collection development services and teaches and facilitates classes on behalf of the library in the hotel.

Results: The library's quantitative and qualitative usage data will be compiled and included by the time of publication.

Conclusions: This library partnership effectively meets the information needs of cancer patients and their families while they are guests in a unique hotel/hospital setting.

Keywords: Consumer health, cancer hospital libraries, health education specialists, hotel setting

Poster Number: 79

Time: Sunday, May 20, 2:00 PM – 2:55 PM

Engaging Students during Grand Rounds through Live Chat

Lori Fitterling, Medical Librarian/Instructor of Medical Informatics, D'Angelo Library, D'Angelo Library, Kansas City, MO; **Bonnie Turner**, Medical Librarian/Instructor of Medical Informatics, Kansas City University of Medicine & Biosciences, Library, Joplin, MO

Objectives: To involve the student audience in an active learning exercise during first year grand rounds case presentations.

Methods: The Medical Informatics and Information Literacy course, with a librarian as course director, emphasizes the development of information literacy and critical thinking skills through a collaborative learning assignment in which student groups present clinical cases in a grand rounds format. These 20-minute presentations to the student body and faculty are followed by a 10-minute Q&A session where groups field questions about their case. Initially, questions were written on previously distributed cards and brought to the panel during the presentation. Librarians observed that the audience was less engaged and lost focus by the end of the case presentations, and participation in the Q&A session was minimal. To address this, an online chat room was introduced and transcripts and student comments were analyzed to determine student participation and interest during the Q&A session.

Results: The addition of the online chat increased the number of questions/comments, encouraged higher quality questions based on the asynchronous delivery of information, and provided a venue for faculty comments on questions that would otherwise not be considered due to time constraints. The online chat also revealed weaknesses in presentation information and format that could be addressed during debriefing sessions with the groups.

Conclusions: Engagement of the student audience has increased as seen by the number of questions and comments, and has strengthened mentoring rolls of faculty contributors. This addition of an active learning feature to first year grand rounds has been a positive focus for this course exercise.

Keywords: Online chat, grand rounds, case presentation, active learning

Poster Number: 80

Time: Monday, May 21, 2:30 PM – 3:25 PM

Transforming the eScience Thesaurus to the Data Thesaurus

Tess Grynoch, Research Data & Scholarly Communication Librarian, University of Massachusetts Medical School, Lamar Soutter Library, UMass Medical School, Worcester, MA

Objectives: As of June 2017, the eScience Portal for Librarians was no longer maintained by the NNLM/ NER and most of the content from the site was moved over to the new nation-wide NNLM RD3: Resources for Data-Driven Discovery site. As part of the move, the eScience Thesaurus, created in 2013, needed to be updated and transformed into the Data Thesaurus.

Methods: The update of the Thesaurus was threefold, update definitions and resources for current terms, identify new terms that needed to be added, and identify how the Thesaurus terms could be better integrated on other site pages. To identify new terms and update current terms, the methodology used by Kevin et al. (2013) to create the Thesaurus was revised to account for the circumstances of the update. The proposed terms were evaluated by the eScience Portal Editorial Board for inter-coder reliability. The records for the new terms were then created and the old records updated. The updated and new records were uploaded and formatted for the new website.

Results: Of the 55 terms from the eScience Thesaurus, 10 were identified for merging. After reviewing the literature, 47 terms were suggested for the Editorial Board to review and members of the Editorial Board added 12 terms to the list which were reviewed by the whole group as well. Of the 59 total terms suggested, 23 were chosen as new terms to be added to the Thesaurus. The updated and new terms can be viewed at https://nnlm.gov/data/data-thesaurus.

Conclusions: A similar revision based off Read et al.'s methodology is recommended every five years with revision of new term suggestions and updates to articles and resources on term pages occurring on an annual basis. The next steps for the Data Thesaurus will be to add more functionality and incorporate links to Thesaurus terms within the rest of the RD3 site.

Keywords: Data, eScience, Thesauri, Research Data Management, Technology

Poster Number: 81

Time: Tuesday, May 22, 1:00 PM – 1:55 PM

Not All Library Analytics Are Created Equal: LibAnswers to the Rescue!

Mariana Lapidus, Reference coordinator, Boston campus, Henrietta DeBenedictis Library, Henrietta DeBenedictis Library, Library & Learning resources, Boston, MA

Objectives:

- To state the reasons for implementing LibAnswers Analytics as a single resource for reference statistics at a three-campus university library.
- To present the advantages of using this software in comparison to previously used reference statistics databases.

To describe the positive impact of utilizing LibAnswers Analytics tools on the overall reference services, library instruction, and reference desk scheduling.

Methods: Springshare products, such as LibGuides and LibChat, have been used at the MCPHS University for several years prior to 2017, when the decision was made to start utilizing LibAnswerss, another product by the same company, in order to keep reference transcript records and a variety of statistical data. LibAnswers Reference Analytics software has replaced two separate reference statistical databases that have been previously used for a long time: in-house designed Access database, which was utilized for in-person, email and phone reference questions, and the LivePerson chat reports used to keep the online chat statistics.
Advantages of switching to the single analytics system are highlighted based on back-to-back comparison with the interfaces and main features of the previously utilized statistical tools, as well as the last year' statistical data and transcripts.

Results: Switching to the single reference analytics system made the process of keeping library statistics seamless, easy and consistent on all three campuses for all types of reference questions.
Among other great advantages, using the new interface allowed to graphically display the busiest days/hours and the number of transactions during certain time slots. In our experience, this had a positive impact on providing effective reference services and ensuring that the most productive reference desk schedule is offered for all types of patrons in order to better meet their teaching, learning and research needs.

Conclusions: LibAnswers Reference Analytics offers librarians a wide variety of excellent capabilities, utilization of which has a positive impact on the overall quality of providing reference services. It allows to seamlessly track all means of reference interactions at the desk, via telephone, chat and email and efficiently gather and process essential statistical information. Administrative interface of this software is easy to use, and a variety of statistical reports can be successfully used to better coordinate reference and circulation desks' schedules.

Keywords: Reference statistics
LibAnswers Analytics
Reference services
Reference desk schedule

Poster Number: 82

Time: Sunday, May 20, 2:00 PM – 2:55 PM

Formalizing an Institution-Wide Journal Club and Research Seminar Elective

Fatima M. Mncube-Barnes, Executive Director, Howard University, Howard University, Arlington, VA; **Lynwood McAllister**, Manager, Special Projects, Howard University Hospital, Graduate Medical Education, Washington, DC; **Peter L. Sealey**, Associate Dean for Graduate Medical Education, Howard University, Howard University, Washington, DC

Objectives: The Office of Graduate Medical Education (GME) and the Health Sciences Library are collaborating to develop a formal institutional wide Journal Club and a Research Seminar Elective.

Methods: To gain the necessary skills of conducting clinical research effectively, all of our residents are encouraged to take the Introduction to Principles and Practice of Clinical Research (IPPCR) course offered by the NIH Clinical Center.Responses from a needs assessment survey identified knowledge gaps in research methods currently utilized across the GME enterprise.

Results: Out of 269 enrollees, 60 percent participants showed great interest in research training and an establishment of formal Journal Clubs within residency programs where a dearth in scholarly activity has been identified.

Conclusions: A formal request has been made to have this program designed to be offered fully online and open throughout the year. In the meantime, a task force will be identified to design a Research Seminar Elective. Journal Clubs will also be encouraged to be offered in all the residency programs. To support research initiatives, the health sciences library will plan a series of monthly topics/workshops to provide knowledge and skills necessary to incorporate best evidence-based practice in the care of individual patients.

Keywords: Journal Club, Research Seminar Elective, IPPCR

Poster Number: 83

Time: Sunday, May 20, 2:00 PM – 2:55 PM

Enhancing Student Writing Skills: Developing a Writing Instruction Workshop

Joshua Shulman, Emerging Technology Librarian, Health Sciences Library, Los Angeles, CA

Objectives: The Charles R. Drew University Health Sciences Library developed a workshop to assist students with formatting papers according to American Psychological Association (APA) Publication Manual guidelines. The workshop was designed to increase student writing competencies by providing participants with an overview of APA requirements as well as access to instructional resources.

Methods: Three APA workshops were conducted for a total of 42 students from the School of Nursing (SON) and the College of Science and Health (COSH) students. The workshop demonstrated how to format manuscript elements, organize papers with various levels of heading, reduce bias in language, cite references in-text, and create reference lists. A post assessment questionnaire was administered to evaluate learning outcomes and effectiveness of the curriculum. Students assessed their perceived proficiencies in using APA guidelines to format and organize papers, avoid biased language, create in-text citations, and create reference lists.

Results: Students assessed their perceived proficiencies in using APA guidelines to format and organize papers, avoid biased language, create in-text citations, and create reference lists using a five-point Likert scale. One signified very low proficiency 5 indicated a very high proficiency. The mean self-assessment score for formatting manuscript elements was 3.83, while organizing the manuscript scored 3.67, reducing bias by topic scored 3.31, citing references in text scored 3.69, and creating reference lists scored 3.90.

Conclusions: Student response to the workshop was overall positive, with some students commenting on the clarity and helpfulness of the presentation and materials. School of Nursing students rated themselves as having a higher proficiency in regards to all tasks, with scores reflecting a higher mean than the other session. Scores were lowest in response to reducing bias. This portion of the workshop contained significant information presented in a short amount of time. Given the low proficiency evaluations, the section might be omitted or presented as a separate workshop. The Health Sciences Library will schedule future workshops given the success of this pilot program.

Keywords: Workshops; Writing Skills; Scientific Writing; Nursing Education; APA style

Poster Number: 84

Time: Monday, May 21, 2:30 PM – 3:25 PM

Power of Nurse-Librarian Collaboration: Aligning Research, Evidence-Based Practice, and Innovation to Sustain a Magnet Culture

Tami A. Hartzell, Senior Librarian, Werner Medical Library, Werner Medical Library, Rochester, NY

Objectives: This presentation outlines the evolving role and contributions of a hospital librarian as a member of the hospital's Evidence-Based Practice Advisory and Magnet Steering Committees.

Methods: Magnet designation is the most prestigious credential a hospital can achieve for nursing excellence and quality patient care. The librarian became involved with the hospital's first Magnet designation in 2003. Now on the epic journey for 4th time re-designation, this presentation outlines the librarian's evolving role to support the ever changing educational and competency requirements of staff nurses. Magnet requires raising the bar for nursing care delivery. Therefore, the goals set for staff nurses change each year. While primarily focused on evidence-based practice (EBP), opportunities have arisen for the librarian to become involved with other hospital-wide initiatives that seek to improve nursing knowledge and clinical quality. Participation in Nursing Grand Rounds, membership in the Magnet Steering Committee and working with the Professional Practice Council are examples of how the librarian role has extended to support Magnet standards of excellence.

Results: Ongoing librarian participation within the Magnet Steering Committee has helped the hospital to achieve three Magnet re-designations. As librarian involvement increased within the Evidence-Based Practice Advisory Committee, it was only natural to include the librarian when the Magnet Steering Committee was chartered. Through dual membership the librarian championed innovative use of OneNote to support the EBP process for the creation or revision of nursing standards. To support nurses' use of EBP across the hospital, the EBP Advisory Committee contracted with Ohio State University for a one week immersion that trained 30 advance practice nurses to become EBP mentors.

Conclusions: There are many ways hospital librarians can impact their hospital's achievement of Magnet designation. To be successful, the librarian needs to establish a base camp close to the front lines. Becoming immersed in Magnet culture, understanding how empirical outcomes are influenced by innovation, patient partnerships, evidence-based practice and evolving technologies within the Magnet Model will help the librarian identify Sources of Evidence that tell the institution's Magnet story. And, while staff nurses at the bedside value the librarian's contribution, it is imperative to establish the same connection with nursing and hospital leadership.

Keywords: Magnet; Librarian value; Hospital librarians; Nurses; Collaboration; Leadership; Evidence-based practice

Poster Number: 85

Time: Tuesday, May 22, 1:00 PM – 1:55 PM

Opportunities to Grow: Adapting Services, Transforming Relationships, and Leading the Informational Way on the Magnet Journey

Katherine Stemmer Frumento, AHIP, Director of Library Services, Greenwich Hospital, Greenwich Hospital Medical Library, East Haven, CT; **Donna Belcinski**, Content Management Librarian, Greenwich Hospital Medical Library, Greenwich Hospital Medical Library, Greenwich, CT

Objectives: To describe the authors' outreach to nurses during their hospital's two year Magnet journey.

Methods: Medical library staff supported the nurses with: participation on committees, such as diabetes education, falls prevention, and nursing research; participation in monthly nursing competency days; new nurses' orientation; evidence-based practice instruction; development of Magnet Journey and Nursing Research libguides; support of nurses pursuing advanced degrees or certifications; and development of a special collection of Magnet resources. Library staff were also present at committee meetings with Magnet surveyors.

Results: Library statistics increased 19% for literature searches, 25% for document delivery, and 50% for tutorials/orientation. Library services were praised during the Magnet exit interview. The hospital received Magnet status within six weeks of the onsite survey. The nurses' gratitude and respect for the library's services and staff increased exponentially.

Conclusions: The library's outreach efforts during the hospital's Magnet journey had a positive effect not only on the outcome of the Magnet survey, but also on the librarians' relationship with the nursing staff. We continue to see an increase in library usage by nurses, as the hospital works to maintain its Magnet status and prepare for the next Magnet survey.

Keywords: Magnet journey; outreach; nursing research; nurse education; transforming relationships; adapting services; information leadership; evidence-based practice.

Poster Number: 86

Time: Tuesday, May 22, 2:00 PM – 2:55 PM

The Role of Libraries in Community Wellness Programs

Margaret A. Hoogland, AHIP, Clinical Medical Librarian, Mulford Health Sciences Library, The University of Toledo, Temperance, MI; **Lucy Duhon**, Collection Sharing Coordinator & Scholarly Communications Librarian, The University of Toledo, Toledo, OH

Objectives: Employee wellness and community wellness are an important component of health literacy. A survey published in 2011 discusses the engagement of consumers in health care and touches on everything from fitness trackers to patient portals. The Patient Protection and Affordable Care Act provided substantial funds for employers to encourage employee participation in wellness programs. Yet library involvement receives scant attention.

Methods: Coordinate with the Toledo-Lucas County Health Department and the Toledo Lucas County Public LIbraries to provide information on nutrition, hydration, fitness opportunities, and healthy local eating options available to the University of Toledo Community. The target population is students, but anyone is welcome to participate. We will create a Bingo card with activities for the "target" areas and give students the opportunity to participate between March 12-16, 2018. Prizes will be donations from the community or provided by the Principal Investigator. This is the first time the library has put together something on community wellness and we are excited to see how the community responds.

Results: We had 8 participants (4 staff, 4 students). We had equal participation from both the Main Campus and the Health Science Campus.

Conclusions: Everyone enjoyed the program and we are excited to do this program again in Spring 2019. We will be planning earlier and do more with social media. This program led to greater collaboration between the University of Toledo (UT) and the Toledo-Lucas County Health Department, which gives UT students more opportunities to gain hands on experience in multiple different settings in the community. We are excited to build this program and continue to forge new relationships in the community and to strengthen our existing relationships.

Keywords: health literacy, employee wellness, student wellness, partnerships, academic libraries, public libraries

Poster Number: 87

Time: Sunday, May 20, 2:00 PM – 2:55 PM

Evaluation of Tools Used in Systematic Review Study Selection and Evaluation

Rachael Posey, Health & Life Sciences Research Librarian and Associate Director, Veterinary Medicine, William Rand Kenan, Jr. Library of Veterinary Medicine, Raleigh, NC

Objectives: To examine the functionality, ease-of-use, and control features for a number of commonly-used software programs used in the creation and preparation of systematic reviews, and to explore the features unique to each tool.

Methods: We used a seven-point likert scale to evaluate the following tools during use for a small systematic literature review: Covidence, Distiller, Rayyan, Parsifal, EndNote, SRDR, and CREBP-SRA. Each tool was scored by two separate reviewers for functionality, ease-of-use, and level of administrator control during the following steps of the systematic review process: uploading, title/abstract screening, full-text screening, data extraction, decision tracking, and exporting. Reviewers were blinded to each other's scores until data collection was complete. We also gathered free-text information related to specific issues and features of each tool and created a list of all of the functions that each tool performs. We then compiled all data and compared the overall average and total scores for each tool individually. We also compared the scores for each tool head-to-head with the scores for each other tool.

Keywords: systematic reveiws

Poster Number: 88

Time: Monday, May 21, 2:30 PM – 3:25 PM

Utilization of Client Survey Results to Enhance Workflows in a Changing Document Delivery Landscape

Sylvie C. Larsen, Supervisor, Document Delivery Services, Memorial Sloan Kettering Cancer Center, Memorial Sloan Kettering Cancer Center Medical Library, New York, NY; **Christine Beardsley**, Librarian, Document Delivery Services, Memorial Sloan Kettering Cancer Center, MSK Library, New York, NY; **Alethea Brisco**, Document Delivery Assistant, Memorial Sloan Kettering Cancer Center, MSK Library, New York, NY; **Donna S. Gibson**, Director of Library Services, Library, Memorial Sloan Kettering Library, New York, NY

Objectives: The purpose of this study was to assess the effectiveness of Document Delivery Services based on client feedback. Library Staff were interested in understanding users' perspective of their use of document delivery, and finding opportunities to augment the level of service.

Methods: A 15-question online survey (2/14/2017 through 3/31/2017) was sent to library users via email to solicit feedback regarding Document Delivery Services. Questions were based on issues identified by library staff. In addition, select questions were adapted from a previous survey distributed to users in 2009 before the launch of ILLiad. In preparation for the 2017 survey, five "power users" were contacted and asked to take the survey and report back any confusion regarding the questions and their experience using the tool. Some questions were modified based on their feedback.

The link to the survey was sent to ILLiad users using various communication channels - emails, social media, and an announcement on the library website. The survey link was added to automated emails sent from ILLiad, and library staff was encouraged to send the survey link directly to their clientele.

Results: Received 592 responses which represented 22% of document delivery users. Selected questions from past survey showed similar results.

We learned that simple changes could have a big impact on service while others required more planning. Some survey respondents provided their contact information and the team took advantage to offer one-on-one teaching moments.

In both surveys, the majority of users provided positive feedback. Insight was received into how best deliver information about our services - FAQs were updated accordingly, webpage revised, customized subject lines developed for automated ILLiad emails, and content was determined for a formal workshop and future interactive tutorials.

Conclusions: Document Delivery continues to be a key service for users in support of their medical and research activities. Client feedback can enhance service improvements and provide direction on the best course of action to pursue. Client engagement surveys sent to users at regular intervals offer an opportunity for them to voice their opinions on what we do right and where we might look to improve our current workflows. Feedback can also be leveraged to craft strong promotional and marketing messages.

Keywords: client engagement survey, document delivery services, workflow

Poster Number: 89

Time: Tuesday, May 22, 1:00 PM – 1:55 PM

Opening Up: Addressing Researchers' Open Source Software Needs

Patricia F. Anderson, Emerging Technologies Informationist; **Marisa L. Conte**, Assistant Director, Research and Informatics; Taubman Health Sciences Library, Taubman Health Sciences Library, Ann Arbor, MI

Objectives: To develop and implement a comprehensive solution to address researchers' needs for open source software for research, including training, information, discovery and licensing.

Methods: Many researcher needs can be addressed by open source software, including scheduling or automating tasks with scripts, cleaning and analyzing data, or employing version control. Few resources exist to help researchers identify relevant open source software tools, and even fewer teach researchers to streamline research workflows with basic programming, or to develop their own software and license it for reuse.

To address these needs, we developed and published an informational resource to help librarians and researchers keep abreast of open source software programs and development. This resource includes an annotated list of relevant open source software programs, a search strategy to identify common or emerging open source programs in general fields or by specific discipline, and commonly-used licenses for open source software distribution.

Keywords: software carpentry, software development, open source, licenses, programming, workflows

Poster Number: 90

Time: Tuesday, May 22, 2:00 PM – 2:55 PM

The Youth Health Literacy Challenge: A Partnership between University of Texas Health-San Antonio Libraries, North East Independent School District, and the San Antonio Public Library

Karen D. Barton, Liaison to the School of Health Professions and Community Engagement Librarian, Briscoe Library, UT Health San Antonio Libraries, San Antonio, TX

Objectives: This poster will describe how UT Health San Antonio Libraries partnered with the North East Independent School District (NEISD) Summer Food Service Program and the San Antonio Public Library (SAPL) on a project, the Youth Health Literacy Challenge, to promote NLM resources that could encourage youth to practice healthy behaviors. This project filled a gap in providing health information resources to the public during feeding times at library branches.

Methods: This project ran in conjunction with the SAPL's Summer Reading Program and was carried out in a similar fashion with youth logging their activities. The logs prompted youth to complete health literacy activities based on challenges written in both English and Spanish. The food service staff handed out the logs during feeding times at four library branches and the public library staff collected completed logs. Additionally, staff from UT Health San Antonio Libraries and SAPL distributed supplemental calendars that included kid-friendly recipes, word games, and information on the NLM's websites for youth. The target audience was underrepresented and underserved youth in grades 3-12, in part due to statistics that 45% of children aged 8-18 in Bexar County are overweight or obese. Teen cooking programs were provided in two library branches to promote the program.

Results: Program partners distributed 1,838 out of 2,253 calendars during the project period. NEISD staff distributed 1,269 out of 1,900 logs, yet no completed logs were returned. The youth seemed to show more interest in taking home a calendar or participating in a cooking program than completing and returning the logs.

Conclusions: Since the school district does not typically hand out health information during its summer feeding program, it would be beneficial to continue to promote health information resources to participants through vehicles such as calendars. Rather than distribute logs, the challenges could possibly be incorporated into a cooking or other library program in unique ways to further engage youth.

Keywords: Health literacy, consumer health, community engagement, outreach, youth, community partnerships

Poster Number: 91

Time: Sunday, May 20, 2:00 PM – 2:55 PM

Evidence-Based Practice Skill Retention and Use by Dietetic Interns: Did Library Instruction Have an Impact?

Rachel J. Hinrichs, AHIP, Health Sciences Librarian, Indiana University-Purdue University Indianapolis, University Library, Indianapolis, IN

Objective: To determine if dietetic interns retain the evidence based practice (EBP) knowledge and skills that they were taught in three library instruction sessions in the fall by the end of the 10-month internship, and whether there is a change in their EBP clinical behaviors.

Methods: This non-randomized before and after study will use a validated survey to measure EBP knowledge, and EBP clinical behaviors. Dietetic interns (n=16) from a large, Midwestern university will be given the survey after EBP library instruction in the fall, and at the end of their internship in the spring. Library instruction sessions will cover PICO questions, database searching, filtered and unfiltered resources, and critical appraisal. A paired t-test will be used to compare interns' scores in the fall and spring.

Results: Fourteen interns (n=14) completed both surveys. On the EBP knowledge assessment, interns scored an average of 18/24 (75%) in the fall after library instruction, and 13/24 (54%) in the spring, a difference that is considered statistically significant ($t(13)=7.0$, $p<.0001$). The decrease was primarily due to missing questions on statistics and advanced Boolean searching. Interns retained and even improved their scores on PICO, MeSH, and the evidence pyramid. A slightly statistically significant change in evidence-based practice behaviors was found between the fall and spring ($t(26)=2.1$, $p=.046$). In particular, interns reported that they searched PubMed ($t(13)=2.8$, $p=.016$), and critically appraised articles more frequently ($p(13)=2.2$, $p=.045$).

Conclusion: Despite the three library sessions occurring early in the internship, these results suggest that interns retain information on PICO, MeSH, and the evidence pyramid, but not on statistics or complex Boolean searching. It is possible that these skills were not used frequently in the internship, so they did not retain the information. Interns did report, however, that they more frequently performed all evidence-based behaviors including searching PubMed, accessing systematic reviews, and critically appraising articles, while decreasing their use of textbooks. While the sample size is small and not necessarily generalizable to other populations, this study suggests that dietetic interns retain some information from library EBP instruction, and do make use of the EBP resources and skills demonstrated by the librarian during their internship. Future studies could examine different health professional students, and test whether spreading library sessions over the course of the year would increase retention and evidence-based behaviors further.

Poster Number: 92

Time: Monday, May 21, 2:30 PM – 3:25 PM

The 21st Century Circuit Hospital Librarian: Transforming Hospital Library Services through the Innovative Use of Technology and Old-Fashioned Teamwork

Stacy Posillico, Health Sciences Librarian, Northwell Health, Eastern Region Hospitals at Northwell Health, Hempstead, NY; **Debra Rand, AHIP**, Associate Dean for Library Services and Corporate Director of Libraries, Zucker School of Medicine at Hofstra Northwell, Hempstead, NY

Objectives: At Northwell Health, a multi-hospital healthcare system, a new position of Regional Hospital Librarian was created in 2016. Using modern e-technology and perennial team building skills, the librarian established a presence in six community hospitals, which resulted in increased awareness and invigorated use of the health system's library resources. We present a successful example for expanding library services across a broad network.

Methods: Northwell Health continuously maintains a robust e-medical library collection and an active librarian staff at its tertiary hospitals. However, due to retirements and attrition, several of its community hospitals had been without an assigned librarian on-site for some time. In fall 2016, system leadership agreed to create the position of Regional Hospital Librarian serving six community hospitals. When off-site, the librarian uses a dedicated cell phone for calls and texts, a laptop for accessing the library e-collection, and screen-sharing technology to provide assistance. The librarian joined long-established committees and councils within each hospital, becoming a known and valuable part of each team. We describe the Corporate Library Director and system administration's hiring process for the new position, creation of a customer survey, the evolution of the role thus far, and challenges encountered and successes enjoyed in the first year.

Results: In the first year of the program, the number of caregivers, educators, and staff at each of the six hospitals who requested and received literature searches or educational sessions from the librarian has increased substantially. Awareness of the existence of the librarian and the e-medical information library is growing. Survey results indicate that caregivers at all of the region's hospitals are benefitting from the presence of a librarian, by saving time; enhancing their research, EBM/EBP, and quality improvement projects; improving their information literacy skills; and gaining knowledge about EBM/EBP resources available via the e-medical information library. Presentations at each hospital by the new librarian have received positive feedback from attendees.

Conclusions: With the advent of new technology and the utilization of enduring teambuilding principles, health sciences librarians can develop a visible presence within a hospital, even without being physically present at the hospital on a daily basis. All caregivers and staff at a hospital benefit from the attention and service of a dedicated hospital librarian, regardless of whether they are served in-person or remotely.

Keywords:
hospital libraries
healthcare systems
e-resources
regional library services
teamwork

Poster Number: 93

Time: Tuesday, May 22, 1:00 PM – 1:55 PM

Patient/Parent Access to Health Sources (PATHS) Leading to Improved Health Outcomes

Rachel F. Fenske, Information Services/Outreach Librarian; Assistant Librarian, University of South Alabama, University of South Alabama Biomedical Library, Mobile, AL

Objectives: This project provides patients and family members of those newly diagnosed with pediatric cancer and other chronic illnesses at the University of South Alabama (USA) Children's & Women's Hospital a means of acquiring and using reputable, easy to understand health resources during the educational processes upon diagnosis and treatment; thus, improving their understanding of the diagnosis and assist in making better health decisions.

Methods: Parents and patients within the hematology/oncology, pediatric, NICU and infusion units of the USA Children's & Women's Hospital receive education in the use of interactive videos, MedlinePlus and NIH resources from a librarian and nurse educators. Android tablets, funded by a NNLM subaward, are used to provide access to these resources during the patient’s hospital stay. Surveys were developed to determine the efficacy of various educational interventions to increase understanding of their child’s diagnosis, treatment plan and care. The expected outcome is that engaging parents/patients in an interdisciplinary team approach will achieve high patient satisfaction in the educational process and positively influence comprehension of vast information disseminated upon diagnosis, assist in continual care of home based procedures, and ultimately improve the patient’s health.

Results: From March - December 2017, there were 96 instructional sessions serving 133 people. Of those, 43 included viewing X-Plain ® interactive videos. Responses from 38 surveys indicated that 45% knew little to nothing about their or their child's condition before viewing a health related video where 55% knew a considerable amount. After viewing the video, there was 95% agreement that it gave patients/parents a better understanding of the diagnosis, procedure or care. This is reassuring given the large percentage who already had a good understanding of the condition. Data from 39 NICU and Pediatric unit surveys indicated 88% agreement that patients/parents liked using computer tablets to locate health information and 85% agreement that searching MedlinePlus gave them more confidence locating quality information about their child's condition. Moreover, 87% agreed they will continue using the resources.

Conclusions: The implementation of Android tablets in patient education has been successful and is now an essential part of the educational process. Integrating technology to address learning styles while providing quality health information from NLM and other patient education resources will lead to improved health outcomes for these patients. Data collection is ongoing to establish evidence-based guidelines for best practices in patient/family education.

Keywords: patient/parent education, new diagnosis, pediatric oncology, educational interventions, computer tablets, Android tablets, pediatrics

Poster Number: 94

Time: Tuesday, May 22, 2:00 PM – 2:55 PM

Does the Availability of Medical Marijuana Decrease Opiate Prescribing? Surveying the Landscape

Patricia Devine, Outreach & Communications Coordinator, National Network of Libraries of Medicine, Pacific NW Region, Seattle, WA

Objectives: To discover the impact of state regulation of medical marijuana on opiate use by asking:

- Do hospitalization rates for substance misuse and overdose drop in states permitting marijuana use for medical purposes?
- Do opiate prescription rates for pain decrease when medical marijuana is permitted?
- How can librarians become knowledgeable and identify resources on this topic for clinicians?

Methods: Marijuana for medical use is now legal in 29 states, but studying the medical effects of marijuana is challenging. Because marijuana is categorized as a DEA Schedule I drug, researchers have encountered difficulty in conducting Randomized Clinical Trials. But research is expanding in this important field. Librarians have a role in raising awareness among healthcare professionals and researchers.

To better understand the landscape of medical marijuana and opiate use, and discover ways for librarians to act as advocates and share high quality information, a literature review will be conducted and results summarized. Key researchers will be interviewed, including: Chris Bundy, MD, MPH, Medical Director of the Washington Physicians Health; David Bradford, PhD, Department of Public Administration and Policy at the University of Georgia; and Ashley C. Bradford; master of public administration student at the University of Georgia.

Keywords: Medical marijuana
Drug policy
Opiates
Pain Management
Chronic Pain

Poster Number: 95

Time: Sunday, May 20, 2:00 PM – 2:55 PM

Clinical Medical Librarian Licensure: Pros and Cons

John Bramble, Librarian, Spencer S. Eccles Health Sciences Library, University of Utah, Eccles Health Sciences Library, NN/LM MCR, Salt Lake City, UT; **Claire Hamasu**, Associate Director, Retired, Spencer S. Eccles Health Sciences Library, Spencer S. Eccles Health Sciences Library, Salt Lake City, UT; **Shawn Steidinger, AHIP**, Medical Librarian, Medical Library, Primary Children's Medical Library, Salt Lake City, UT

Objectives: In hospitals and clinics, anyone who "touches" a patient has a license authorizing them to do so...from the phlebotomist to the cardiologist, from the genetic counselor to the social worker and so on. Everyone, except the clinical medical librarian, that is. The question of requiring a license to practice clinical medical librarianship is a topic worth discussing.

Methods: This presentation will explore the first step in this discussion, understanding the positives and negatives of occupational licensure. The main purpose of licensure is ensuring health and safety protections to consumers. Licensure has benefits, especially for workers, one being protection from competition by limiting fields to those who demonstrate they have the essential knowledge and skills to practice their profession. Licensure, however, comes with substantial costs. Evidence suggests many of the requirements for licensure do not match the actual skills needed to practice. Further evidence shows licensure may increase the costs for goods and services, restrict employment opportunities, and cause difficulty taking skills to another state. Should licensure be required for clinical medical librarians? This poster will inform your point of view.

Results: There is no shortage on the pros and cons of occupational licensure. The results of the Pros and Cons occupational licensure most relevant to Clinical Medical librarianship will be discussed during the presentation.

Conclusions: The conclusions of the Pros and Cons of Clinical Medical librarianship will be discussed during the presentation.

Keywords: clinical medical librarianship occupational licensure health safety protections

Poster Number: 96

Time: Monday, May 21, 2:30 PM – 3:25 PM

Art, Technology, Medicine: Creativity and Innovation in the Health Sciences

Skye Bickett, AHIP, Assistant Director, Library, GA-PCOM, Suwanee, GA; **Erica V. Rosalle**, Library Specialist, Georgia Campus - Philadelphia College of Osteopathic Medicine, Library, Suwanee, GA; **Meghan T. Di Rito**, Education and Outreach Librarian, Georgia Campus of the Philadelphia College of Osteopathic Medicine, Library, Suwanee, GA

Objectives: Our goal was to introduce our community to ways in which creative arts (e.g. painting and music), or technological arts (e.g. video games), intersect with the healing arts. We held an event that brought our community together for fun and stress relief, while teaching about different aspects of medical education, patient education, and therapy.

Methods: TThis program was the cornerstone of a series of events celebrating National Medical Librarians Month. Our audience was the students, faculty, and staff of a graduate-level health sciences school. We included staff in our programming, drawing on the expertise and talent of other entities outside of the Library. Thus, the program expanded from an introduction to graphic medicine to include other aspects of artistic and technological expression. The event had four components: Graphic Medicine, Art and Music Therapy, Gaming Technologies and Virtual Reality, and Stress Relief. Each station included a demonstration or example of the component, description of the therapeutic or educational modality, and a digital literature list for the community to learn more about how health care professionals use these methods in enrichment of personal health or patient education and treatment.

Results: Based on feedback from the campus community, this event was a success. We asked attendees at each station to fill out an informal satisfaction survey, indicating whether they liked, were indifferent to, or disliked the station. Survey completion at each station ranged from 14-55; however, not all attendees filled out the survey. We received verbal feedback from station facilitators, attendees, and passers-by that will help improve future events. Responses to each station were overwhelmingly positive, and many attendees expressed their excitement for next year’s event.

Conclusions: Our analysis of the event revealed areas for improvement. We noticed that stations that had interactive elements resulted in more engagement. Stations without interactive components received more traffic, but the audience spent less time at the station and were less engaged. Because of these results, we plan to incorporate an activity into each station next year. Data collection was an issue because not every participant filled out our survey. Many potential attendees told us that they could not attend because of testing or other class-related conflicts. We will take these factors into account when planning next year’s event.

Keywords: art, technology, interprofessional

Poster Number: 97

Time: Tuesday, May 22, 1:00 PM – 1:55 PM

Patient-Centered Rounding in an Inpatient Pediatric Setting

Judy C. Stribling, AHIP, Assistant Director, Clinical Services; **Keith C. Mages, AHIP**, Clinical Medical Librarian; **Diana Delgado, AHIP**, Associate Director, Information, Education and Clinical Services ; Samuel J. Wood Library & C.V. Starr Biomedical Information Center, New York, NY

Objectives: Clinical Medical Librarians provide unique health literacy services for patients, families, and caregivers on the Phyllis and David Komansky Center for Children's Health pediatric floors of NewYork-Presbyterian Hospital by conducting twice weekly patient rounds and bringing consumer health information and services to bedsides. The Pediatric Consumer Librarian Service generates statements of appreciation from patients and parents, enthusiasm among stakeholders, and reports of increased job satisfaction by librarians.

Methods: Development of the PCLS began with the formation of a team who held held a brainstorming session where it defined the program's mission and goals, identified key clinical stakeholders - physicians, nurses, social workers, and the hospital's Child Life staff - and drafted an initial service overview. The primary mission of the PCLS is to provide pediatric patients and their family members with access to reliable, targeted, and timely health information resources that encourage and enable informed decision making. Secondarily, the team seeks to improve patient satisfaction and comfort during their hospital stay. Goals of the program included returning health information on the same day requested, marketing PRC and medical library services.

Results: Between August 9, 2016 and October 27, 2016, the PCLS team conducted 24 rounds on the general Peds and PICU floors and answered 94 patient, family,or health-provider questions. Then, 139 health information items were delivered in a variety of formats: printed material delivered at bedside 127 (13 in Spanish Language); printed material sent via U.S. mail 2; electronic material sent via e-mail 7 ; 1 videos displayed at bedside; and 2 books loaned to patients.

Conclusions: The program's success is partially demonstrated by usage statistics and observed anecdotal comments, however, a formalized evaluation tool is not currently utilized. The PCLS team plans to develop survey tools to measure the program's impact upon patient satisfaction and knowledge retention. A separate tool to measure satisfaction among clinicians is also being considered. Formal program evaluation will be undertaken at 6 and 12 months, and the program adjusted accordingly. Connecting with preteen pediatric patients has been one of the most difficult tasks faced by the team. The Assistant Director purchased children's coloring books related to hospitalization

Keywords: Consumer health; hospital patients; inpatients; pediatrics; rounds; librarian rounds

Poster Number: 98

Time: Tuesday, May 22, 2:00 PM – 2:55 PM

Graphic Novels and the Humanity of Mental Illness: An American Library Association Carnegie-Whitney Funded Annotated Bibliography Project

Tina L. Hefty, Assistant Director of Library Services, Rocky Vista University, Frank Ritchel Ames Memorial Library / Rocky Vista University, Ivins, UT; **Jenifer A. Fisher**, Research Librarian, Instructor of Medical Humanities, Rocky Vista University, Frank Ritchel Ames Memorial Library, Parker, CO

Objectives: We were awarded a Carnegie-Whitney Grant to prepare and disseminate an annotated bibliography that organizes and describes graphic novels written about mental illness. We anticipate the product will be especially beneficial to patients/caregivers, medical professionals, and librarians. The objective of our presentation is to introduce this project to the medical library community.

Methods: Given the fact that approximately one in five adults experiences mental illness in any given year, it is safe to say that its impact is both deep and wide. Reading graphic novels about mental illness can be an especially beneficial endeavor for people experiencing emotional challenges, as those individuals can find reassurance in knowing they are not alone. Additionally, medical professionals can benefit greatly from these works, as the visual aspect of graphic novels skillfully reveals the deeply personal, and often hidden, side of mental illness.

It is our goal to help patients and medical professionals connect to these resources. One of the ways we plan to meet this goal is by heightening awareness among medical librarians. Sharing information about our annotated bibliography with the medical library community would be hugely beneficial in ensuring the success of this project.

Results: The online guide can be accessed at https://library.rvu.edu/graphicmedicine. It was officially launched in early May of 2018. That said, many details pertaining to our assessment of this product, including its impact and overall reception, are not yet available. We hope MLA attendees will provide us with feedback or utilize the feedback form on the online guide.

Conclusions: Although we hope everyone will enjoy this website, we anticipate it will have particular benefit for three user groups listed under Objectives. We invite you to view our poster for further details and conclusions.

Keywords: Annotated Bibliography, Mental Health, Narrative Medicine, Graphic Novels, Graphic Medicine

Poster Number: 99

Time: Sunday, May 20, 2:00 PM – 2:55 PM

Facilitating Literature Searching on Patient, Caregiver, and Allied Health Education: A Comparison of Bibliographic Databases

Jane Morgan-Daniel, AHIP, Community Engagement and Health Literacy Librarian, University of Florida, Health Science Center Libraries, University of Florida, Gainesville, FL; **Nancy Schaefer, AHIP**, Reference and Instruction Librarian, Biomedical & Health Information Services, Health Science Center Libraries, Gainesville, FL

Objectives: Within allied health, the absence of a comprehensive, interdisciplinary database significantly challenges practitioners seeking literature on patient, caregiver, and student education strategies. This study examines topic-related coverage in five bibliographic databases. It identifies preferred databases and search techniques on the basis of unique features and effectiveness, thereby reducing the barriers associated with finding evidence on pedagogy.

Methods: Sample research questions on educational methods and instructional models for patient, caregiver, and student education were solicited from a purposive sample of allied health faculty in Occupational Therapy, Physical Therapy, and Speech, Language, and Hearing Sciences. An evaluation rubric was developed based on the themes discerned in these questions. Two health sciences and three education bibliographic databases were searched for pedagogical literature. Using the rubric, the authors compared database coverage, controlled vocabulary, limits, and content relevance of the retrieved results. Other considerations included capacity for proximity searching and truncation within phrases, and overlap between the search results.

Results: CINAHL and PubMed retrieved the largest percentages of relevant results for the student and patient education questions. For unique relevant results, PubMed yielded the greatest percentage for the student and patient education questions. ERIC yielded the highest percentage for caregiver education, while Education Source netted the second highest percentage for student education.

Conclusions: These results demonstrate the value of searching both education and health sciences databases for allied health educational research questions, with the most useful specific database varying according to the targeted educational intervention group.

Keywords: Allied Health,
Literature searching,
Bibliographic databases,
Patient and caregiver education

Poster Number: 99

Time: Tuesday, May 22, 1:00 PM – 1:55 PM

Validating the MEDLINE®/PubMed® Health Disparities and Minority Health Search Strategy-Spotlight: Race and Ethnicity

Q. Eileen Wafford, Research Librarian, Northwestern University, Galter Health Sciences Library, Chicago, IL; **Corinne H. Miller**, Clinical Informationist, Northwestern University, Galter Health Sciences Library, Chicago, IL; **Linda O'Dwyer**, Interim Head, Research and Information Services, Galter Health Sciences Library, Galter Health Sciences Library and Learning Center, Chicago, IL

Objectives: We will validate the MEDLINE®/PubMed® Health Disparities and Minority Health Search Strategy. Given the complexity of health disparities, we elected to validate the filter in stages based on health disparities domains, starting with race and ethnicity. We will use our analysis to provide recommendations that optimize the sensitivity and specificity in retrieving health disparities citations that cover race and ethnicity.

Methods: We will develop an inclusion criteria that incorporates established definitions and information gathered from a survey of experts. Using smoking cessation to focus our search, we will apply the inclusion criteria and find all relevant Cochrane reviews and cited clinical trials that are also indexed in PubMed to develop a validation or “gold standard” set of citations. The reference set will be citations on diabetes mellitus from the MEDLINE®/PubMed® Health Disparities and Minority Health Search Strategy subset. We will calculate the sensitivity, precision, specificity, and accuracy of the validation set using the reference set. From our findings, we hope to identify candidate search terms from the validation set by hand searching titles and abstracts with Rayyan and frequency analysis. We will retest the sensitivity, precision, specificity, and accuracy of the candidate terms using the reference set and make recommendations accordingly.

Keywords: Health disparities, filter validation, hedges, search strategies

Poster Number: 100

Time: Monday, May 21, 2:30 PM – 3:25 PM

The Rewarding System of Library Resources Promotion Program of Taipei Medical University Library

Shu-Yuan Siao, Head, Division of Knowledge Services, Main Library, Taipei Medical University Library, Taipei, Taipei, Taiwan (Republic of China); **Tzu-Heng Chiu**, Professor, Center of General Education/International Cooperation Division, National Central Library, Center for General Education, Taipei, Taipei, Taiwan (Republic of China); **Chi-Ju Chiu**, Reference librarian, Knowledge Services Sec.,, Taipei Medical University, Taipei Medical University Library, Taipei, Taipei, Taiwan (Republic of China); **Hsiao-Fen Yu**, Reference librarian, Knowledge Services Sec., Main Library, Taipei Medical University Library, Taipei, Taipei, Taiwan (Republic of China)

Objectives: In order to attract the faculty and students to the library and to use the library resources, the TMU library integrates all its promotion programs, designs a rewarding system of sticker collecting and rafflering to encourage them to know and utilize the services and resources of TMUL. This poster presents the results of those promotion activities.

Methods: The promotion activities of TMUL services and resources included books borrowing, electronic resources training sessions, database learning sheets, book recommendation, lectures, …etc. The rewarding system is designed for users to get one sticker for borrowing one book, get two stickers for completing a learning sheet, get one sticker for attending other promotion activities…etc. And theses stickers then can raffle a prize (stationery, mugs, USB, mobile power supply, iPad mini…) or exchange scholarship (US$17, US$33, US$67, and US$100).

Results:
The TMUL have been held this semester-long promotion program for 9 semesters, with the cumulative participants of 2,034. According to the feedback from the questionnaires, the overall satisfaction is 4.1 (in a 5-point scale) and users' abilities of using, evaluating, and gathering information had been enhanced. In addition, we also achieved the purpose of raising the usage of library resources in a big scale.
The qualitative feedbacks are as follows…
"Via answering questions of the learning sheet, I knew how to use electronic resources more effectively."
"The virtual medical humanities exhibition enabled me to learn very rich content at home."
" I feel I am pretty good! "
" Very interesting. "
" This program not only made me know more about the electronic resources of the library, but also made me aware of many websites related to research articles. "

Conclusions:
After years, we found that a semester-long promote program is too long for student users, as a result, the participant numbers show the trend of declining. In the future, the TMUL will try to launch more diverse resources promotion activities, and held them with festivals and events such as back to school, graduation, library week, international OA week … etc.

Keywords: Promotions of library services and resources, TMU, rewarding system design

Poster Number: 101

Time: Tuesday, May 22, 2:00 PM – 2:55 PM

Painting Away the Stress: Use of Art Workshops to Effect Stress Reduction in an Accelerated Pharmacy Program

Mickel Paris, Health Sciences Librarian, University of Pacific, Rite Aid Information Commons, Stockton, CA; **Rachel Keiko. Stark, AHIP**, Health Sciences Librarian, Library, California State University, Sacramento University Library, Sacramento, CA; **Ed Rogan**, Assistant Professor, University of the Pacific, Thomas J. Long School of Pharmacy and Health Sciences, Stockton, CA; **Mary-Kate Finnegan Dopkins**, Interim Health Sciences Librarian, University of the Pacific, Rite Aid Information Commons, University of the Pacific, Stockton, CA; **Cassie A. Etter**, Access Services Specialist, University of the Pacific, University of the Pacific Libraries, Stockton, CA

Objectives: The objective of the research is to assess the effect of art workshops on student self-reporting of stress in a University of the Pacific accelerated Pharmacy program. The health sciences library, in collaboration with pharmacy faculty, hosted three art workshops with the goal of positively effecting the reduction of stress levels for students creating art work following an introductory lesson.

Methods: Students were invited to participate in up to three 2-hour art classes, with each class focused on a different medium in the Rite Aid Information Commons. The classes were taught by a school of pharmacy professor and the classes were advertised through email, classroom announcements, social media announcements, and physical posters available throughout the School of Pharmacy and Health Sciences. Students were asked to complete the self-evaluation questionnaire (STAIP-AD Test Form Y), a validated measurement to assess stress, before and after participating in the art class. The Health Sciences Librarian provided the measurement to the students and the students were informed that the School of Pharmacy professor did not have access to data until all identifying information has been removed.

Results: Results are not complete. Once all data has been collected, it will be processed using statistical software and analyzed. Results will be made available during the poster session at MLA ’18.

Conclusions: Conclusions are not complete and will be given following interpretation of results. Conclusions will be made available during the poster session at MLA ’18.

Keywords: art workshops
pharmacy
stress testing
STAIP-AD Test Form Y

Poster Number: 102

Time: Tuesday, May 22, 2:00 PM – 2:55 PM

The Art of Conversation: A Comparative Analysis of Best Practices and Guidelines for Reference Interviews and Medical Interviews

Caitlin Plovnick, Coordinator for Teaching & Learning, Seattle University Library, Seattle, WA; **Rachel Keiko. Stark, AHIP**, Health Sciences Librarian, Library, California State University, Sacramento University Library, Sacramento, CA

Objectives: The purpose of this study is to examine the similarities and differences between reference interviews and medical interviews. The reason for this is to identify overlaps between the practice of librarians and health professionals, and find common ground for communication, as well as to seek to improve the reference interview by incorporating best practices from other fields.

Methods: This project consists of a comparative content analysis of interview guidelines identified from professional associations and scholarly literature.

Results: Findings uncovered structural similarity between different guidelines, with key differences identified in terms of nuance and engagement with specific populations and sensitive situations.

Conclusions: There is much that librarians can learn from health professionals in terms of overlapping skills and interview techniques, that has not yet been fully explored.

Keywords: Reference, Reference Interview, Medical Interview, Professional Practices,

Poster Number: 103

Time: Sunday, May 20, 2:00 PM – 2:55 PM

Faculty's Behaviors and Attitudes toward Open Access Journals in Taipei Medical University

Tzu-Heng Chiu, Professor, Center of General Education/International Cooperation Division, National Central Library, Center for General Education, Taipei, Taipei, Taiwan (Republic of China); **Shu-Yuan Siao**, Head, Division of Knowledge Services, Main Library, Taipei Medical University Library, Taipei, Taipei, Taiwan (Republic of China); **Hui-Chun Lai**, Scholarly communication librarian, Taipei Medical University, Taipei Medical University Library, Taipei, Taipei, Taiwan (Republic of China); **Hsiao-Fen Yu**, Reference librarian, Knowledge Services Sec., Main Library, Taipei Medical University Library, Taipei, Taipei, Taiwan (Republic of China)

Academic libraries have been the advocate of Open Access for years. This study investigates knowledge, behaviors and attitudes towards OA journals (OAJ), and behaviors of paper submission and knowledge sharing of all fulltime faculties in TMU. Research findings will serve as reference for the TMUL to design the future OA services and promotion policy.

In response to the 2017 International OA week, the TMUL holds series promotion activities, including this survey. The research team design an online questionnaire, and then sent it to all fulltime faculty (n=624) of the University. The questionnaire consists of four parts: 1.Backgound information (current college, current position, full time faculty position year, experience to OAJs… etc.); 2. Behaviors of submitting papers(importance of each journal factor and reader group when submitting papers.) 3. Opinions toward OA (OA opinions, APC opinions, scenarios, APC fund, expected library role…etc.), and 4. Knowledge sharing & fulltext download (reasons, times, sources, etc.). Via the survey, we hope to explore behaviors and attitudes of our faculty and also make them have a deeper understanding about OA.

129 questionnaires were received with the return ratio of 20.67%. Major findings are as follows: (1) 72.9 % of respondents have ever submitted papers to open access journals, 92.2% ever read OAJs, and 56.6% did deposited publications in TMU's Institutional Repository system. (2). When choose a journal for submission, the most important factors are scope (98.4%) and reputation (96.9%) of that journal. (3). 79.1 % of respondents are willing to submit papers to high quality academic OAJs, and 42% disagreed that the amount of the article processing charge (APC) reflects the quality of journals. (4). 69% say they have ever used research fund to pay APC and 46.5% ever paid by their own money. In average, the respondents think 517 USD is reasonable for a OAJ's APC.

General speaking, the fulltime faculty of TMU have positive attitudes toward OA. However, they think the reasonable price for APC is 517USD and this amount is far away from the reality. Based on our data, among the top 10 academic journals that TMU faculty published their papers in 2017, seven of them are OAJs. Therefore, the TMUL joined the membership of BioMed Central (BMC) in November 2017 to support the OA movement in our campus. Since then, faculty can get 15% off for ACP of BMC journals.

Poster Number: 104

Time: Monday, May 21, 2:30 PM – 3:25 PM

Clinical Librarians and Their Essential Services in Academic and Health Care Settings: A Comparative Survey

Sarah T. Wright, Clinical Librarian; **Rebecca C. McCall, AHIP**, Clinical Librarian; University of North Carolina-Chapel Hill, Chapel Hill, NC

Objectives: While there are many indicators and models of individual successful clinical librarian services and programs in health sciences libraries in the literature, there is no recent, comprehensive summary of services provided by clinical librarians. This poster, based on a current survey of clinical librarians, provides summary data of clinical librarian service models in the US and internationally.

Methods: In February, 2018, we distributed an online survey to librarians who currently provide clinical services in academic and/or hospital settings. Requests to complete the survey went to multiple library listservs frequented by clinical librarians. The survey collected statistics in the following areas: clinical team rounding; participation in departmental activities; instruction for students, residents, or professional staff; expert searching and research involvement; identification of clinical teams served; the ratio of clinical librarians to all health sciences librarians at institution, the average number of clinical departments served; and their clinical environments and geographic locations. The authors asked survey questions regarding clinical librarians' tasks and their perception of service impact on their constituents.

Results: Three hundred and thirty-eight participants responded to the 14 question survey. Responses to the ranked questions regarding services were coded by the authors. Overall, 180 respondents answered all questions. The remaining participants answered some of the survey questions. 27% survey responses were from individuals at academic libraries, and 63% of responses were from hospital libraries. 52% of respondents were from the United States, while 48% were from international respondents, including Canada, UK, and Europe. The majority of clinical librarians serve physicians, residents, allied health professionals, and nurses. From this survey, the authors can make the following statements: Clinical librarians serve multiple specialties working with a variety of clientele. In ranking their services, clinical librarians feel that they provide these top impact services to their clientele: 89% save clinicians time, 87% advance evidence-based practice, and 76% increase teaching skills of clinicians.

Conclusions: Our analysis of current trends in clinical librarian services provides an overview of service models and the areas of focus clinical librarians have in their current positions. These results can be used to: 1) give creative ideas for services to current clinical librarians, 2) help justify a clinical librarian service to management, and 3) assist a new clinical librarian in providing creative and meaningful services to health care professionals.

Keywords: Clinical Librarians, Library Services, Mixed Methods Research, Librarian Roles

Poster Number: 105

Time: Tuesday, May 22, 1:00 PM – 1:55 PM

Phone a Friend (or a Librarian): Learning and Communication Preferences among Occupational Therapy Students at Three Universities

Lisa A. Adriani, AHIP, Research and Instruction Librarian, Edward and Barbara Netter Library, Quinnipiac University, Hamden, CT; **Daniel G. Kipnis**, Life Sciences LIbrarian, Campbell Library, Rowan University, Glassboro, NJ; **Daniel Verbit**, Liaison to Occupational Therapy, Jefferson University, Gutman Library, Phila, PA; **Ronda Kolbin**, Public Services Librarian, Quinnipiac University, Arnold Bernhard Library, Hamden, CT

Objectives:
Medical librarians from three universities surveyed occupational therapy (OT) students to understand their learning preferences and to examine their tendencies for seeking research assistance. Questions included: Whom do OT students turn to for reference help and where do librarians rank? How much time do OT students spend on their research before seeking assistance? What are OT students preferred method for learning content? The librarians also aimed to compare the results with a 2006 survey to see if learning preferences from OT students have changed over the past decade.

Methods:
Librarians distributed an anonymous questionnaire to enrolled students in occupational therapy programs using a web-based survey tool during the fall of 2016. The questionnaire included 29 multiple choice and modified Likert scale questions. OT students were asked about their preferences for specific communication methods with the librarians, learning modules, timing of database instruction, style of written instructions, and social media.

Results:
The survey collected responses from 136 students. The majority of the responses came from first year OT students, but the survey included undergraduate, masters and doctorate students. Most students turned to their classmates for help followed by the librarians and Google. Students preferred in person communication above email, live chat, telephone. There was a strong preference for database instruction at the beginning of the semester. There were other incidental findings with distance education students. Distance education students preferred to contact a librarian first before a classmate and also preferred to use email first while in-person contact was last.

Conclusions:
The results validate the work the three libraries have in place. The majority of instruction sessions are offered toward the beginning of the semester. A comparison of the data with a similar study from 2006 was performed. In both studies, the students first turned to their classmates for research help, while the librarian ranked second in 2016 and third in 2006. In 2006, librarians ranked behind a friend (second place) although the difference was minimal. In 2016, the majority of students indicated they would seek research help after 30 minutes to 1 hour of work and also preferred to communicate with the library in person. In 2006, the majority of students said they sought research help prior to 30 minutes and preferred to communicate with the library in person.

Keywords: Occupational therapy, Information literacy, Library instruction, Rehabilitation sciences, Communication, Teaching

Poster Number: 106

Time: Tuesday, May 22, 2:00 PM – 2:55 PM

The Center for Data to Health (CD2H): Leveraging Library Expertise

Kristi L. Holmes, Library Director, and Associate Professor of Preventive Medicine, Health and Biomedical Informatics Division, Galter Health Sciences Library, Galter Health Sciences Library & Learning Center, Chicago, IL; **Karen Gutzman**, Impact and Evaluation Librarian, Galter Health Sciences Library, Galter Health Sciences Library, Chicago, IL

Objectives: The new National Center for Data to Health (CD2H) was recently funded by the National Center for Advancing Translational Sciences for the CTSA Program as a data and informatics coordinating center to provide collaborative research infrastructure. Here we describe the CD2H, the areas of cultural and technological importance for focus, CD2H goals and how researchers and librarians can actively participate in the community.

Methods: Here we will address several key objectives: describe the mission of the CD2H, including opportunities for collaborative innovation and the barriers that the CD2H center aims to overcome; describe the governance structure and communication channels of the CD2H; describe a wide range of collaborations and activities for community engagement - especially ways that libraries and librarians can get involved and leverage resources from this project for their own campuses; and discuss opportunities for the CD2H to catalyze the work of the clinical and translational sciences community.

Results: There are a number of ways that library and information community members can intersect and partner with the CD2H. Working groups for the CD2H present a great opportunity for collaboration and communication about projects, initiatives, and new ideas. The working groups are topical and cover a wide range of thematic areas: data; software, tools, & algorithms; ontologies & standards; people, expertise, & attribution; engagement; education; and evaluation & analytics. Two additional working groups address the CD2H's major demonstration areas of rare disease and the lifespan. Join us! We're looking for individuals and teams to help us advance biomedical research informatics and data science to support interoperability, collaborative innovation, and training, and more.

Conclusions: By attending this poster, attendees will learn about the CD2H, understand ways to get involved and benefit, as well as have an opportunity to discuss the CD2H to Health directly in person with librarians actively involved on the project. The CTSA Program National Center for Data to Health (CD2H) is supported by the National Center for Advancing Translational Sciences (NCATS) at the National Institutes of Health (Grant U24TR002306).

Keywords: informatics, data, collaboration, team science, education, evaluation, engagement

Poster Number: 107

Time: Sunday, May 20, 2:00 PM – 2:55 PM

Flipping the Classroom for Graduate Medical Education: Library/Residency Program Collaboration

Gale Oren, AHIP, Librarian, Kellogg Eye Center, Kellogg Eye Center, University of Michigan, Ann Arbor, MI

Objectives: To demonstrate a successful collaboration between a medical librarian and an Ophthalmology Graduate Medical Education (GME) program in flipping the residency lecture series. The flipped classroom model entails presenting the lecture and materials in advance, followed by shortened interactive in-class sessions.

Methods: In July 2017 the GME Program of the Kellogg Eye Center, began implementation of a flipped classroom model for the residency lecture series. The team consisted of the Residency Program Director and Coordinator, the Chief Resident, the departmental Librarian, and faculty advisors. Prior to July, the residency lecture series consisted of traditional teacher-based lectures. First steps included decisions about the platform, the model, and the timeline. The University's Learning Management System, "Canvas", was to be the platform upon which the modules would be built. As the success of this project was dependent on faculty and resident participation, much effort was put into educating all parties of the goals, processes, benefits, and anticipated outcomes of the project. Each lecture module includes advance study materials such as podcasts, questions, case-study flashcards for in-class discussion, and recommended reading materials.

Results: The Kellogg Eye Center Education Resources site went live on Canvas on July, and after a bumpy start, by the end of August was going strong. Over the past two months there has been 100% faculty participation, and good resident compliance, as measured by Canvas analytics. A unit quiz has been administered as part of the ongoing assessment. An initial satisfaction survey is in process to see how the flipped classroom can be improved upon.

Conclusions: This poster will discuss the process of flipping the graduate medical education lecture series, challenges faced, lessons learned, early outcomes of the project, and the role of the librarian.

Keywords: Medical education
Flipped classroom
Librarian roles
Learning Management Systems

Poster Number: 108

Time: Monday, May 21, 2:30 PM – 3:25 PM

Active Learning in Library Instruction for Students in Problem-Based Learning

Rebecca O. Davis, Senior Lecturer, Simmons College, Simmons College / School of Library and Information Science, Boston, MA

Objectives: Discuss active learning in library instruction sessions for students in Problem Based Learning. Discuss activities used by librarians in the instruction sessions for students and how they connect with the goal of Problem Based Learning.
Discuss other possibilities for active learning activities for students.

Methods: Reviewing the literature to learn what has been done in the past and currently combined with my experience and evaluation of the activities that I used in library instruction. The review of literature will focus on Health Sciences/Medical Libraries because Problem Based Learning is used in the medical/health programs. I have experience working with dental students in PBL and providing library instruction sessions with active learning activities.

Keywords: active learning, library instruction, Problem Based Learning

Poster Number: 109

Time: Tuesday, May 22, 1:00 PM – 1:55 PM

Postcards from the Archives: A Glimpse of Osteopathic History

Lori Fitterling, Medical Librarian/Instructor of Medical Informatics; **Robyn Oro**, Access Services/Special Collections Coordinator; D'Angelo Library, Kansas City, MO

Objectives: To research, catalog, preserve and digitize postcards from the library archives. Each postcard is associated with some aspect of osteopathic medicine or the university.

Methods: Until recently, 26 postcards were stored in various folders in the general archival collection at the library. Librarians determined that these postcards had historical significance and should be a part of the collection. Dates of publication and condition about each item, were noted in the research and cataloging process. Items were digitized in-house using a Bookeye 4 scanner with OPUS software, and made accessible using ContentDM through the library's digital archives collection. The physical copies were placed in sleeved dividers and stored in a temperature controlled room for preservation purposes.

Results: Twenty-six postcards are now available in digital format and can be accessed with the library archives collection from the library website. An image of the address side of the postcard is linked to each record and some postcards contain messages and stamps. The postcard collection features several hospitals and campus buildings associated with osteopathic medicine from the years 1908 to 2010. There are also postcards depicting A.T. Still associated locations and artifacts. Postcards of note: B.B. Springs, Bowling Green, MO - was a bath house run by an osteopathic physician; Central College of Osteopathy Faculty (Kansas City, MO) 1908-09.

Conclusions: The printing of postcards began in the United States in the 1800's providing a way to send a small note or card for less postage. This collection of postcards spans several years and displays images of hospitals and university buildings from the 100-year history of the campus. The focus on osteopathic medicine as seen with the A. T. Still cabin, statue, and portrait postcards, helps to establish the osteopathic role in our university history. By preserving these types of artifacts, and making them accessible, the library is meeting the mission to preserve the history of the university.

Keywords: postcards osteopathic medicine archives

Poster Number: 110

Time: Tuesday, May 22, 2:00 PM – 2:55 PM

Transforming Library Space: Promoting Healthy Babies with a Nursing Mother's Room

Sarah C. Adcock, Instruction & Research Librarian, University of Mississippi Medical Center, Rowland Medical Library, Jackson, MS; **Elizabeth G. Hinton, AHIP**, Instruction & Research Librarian, Rowland Medical Library, Rowland Medical Library, Jackson, MS; **Susan B. Clark**, Director, Rowland Medical Library, Rowland Medical Library, University of Mississippi Medical Center, Jackson, MS; **Chameka C. Robinson, AHIP**, Head, Access Services, University of Mississippi Medical Center, Rowland Medical Library, Jackson, MS

Objectives: The library's institution currently has two nursing mother's rooms in the hospital, neither is conveniently located for students and non-clinical employees. In an effort to support the institution's recently achieved "Baby-Friendly" status and the active promotion of breastfeeding among new mothers, the library seeks to provide space for nursing in a private, unused room within the library.

Methods: The CDC's "Healthy People 2020" breastfeeding objective is for 81.9% of infants to be breastfed for any length of time, or "ever breastfed." Working with the Nursing Mother's Room Task Force and following guidelines set by the Patient Protection and Affordable Care Act of 2010, an unused room in the library will be repurposed into a place suitable for new mothers to pump breast milk. The room will remain locked for safety and privacy (as determined by the lactation specialist nurse), so the key will be checked out to each user at the library's circulation desk. Since usage of the room will be tracked, first-time users will receive a short survey. The survey will include questions about satisfaction with the nursing room, and success will be measured by overall user satisfaction and frequency of use.

Results: An unused room within the library was remodeled to accommodate two women pumping breast milk. After collaborating with numerous departments within the institution (a large academic medical center), the six-week construction project began late fall of 2017. The room features a sink, refrigerator, computers, counter with counter-height electrical outlets, privacy wall, new flooring, paint, adjustable chairs, and artwork. Room usage policies were developed and posted in the room and on the library's website. Marketing efforts included: a feature article in the institution's online bi-weekly publication, screensaver notifications on in-network computers, advertisements on the library's BrightSign, and communications with student and staff leaders. In the first three months since opening, nine breastfeeding mothers have used the room with a total of 153 room checkouts. Survey responses and comments were overwhelmingly positive. 100% of respondents replied that the location of the room is convenient and they would use room again.

Conclusions: Rowland Medical Library supports breastfeeding mothers by providing a convenient, functional, and attractive space to pump breast milk. Moving forward, Rowland Medical Library will continue to market the nursing mother's room, monitor and refine room policies, and investigate utilizing the new LMS system to schedule and indicate room privacy preferences.

Keywords: library space, nursing mothers, breastfeeding, outreach

Poster Number: 111

Time: Tuesday, May 22, 2:00 PM – 2:55 PM

Promoting National Library of Medicine Resources through Digital Signage

Elizabeth G. Hinton, AHIP, Instruction & Research Librarian, Rowland Medical Library, Rowland Medical Library, Jackson, MS; **Sarah C. Adcock**, Instruction & Research Librarian, University of Mississippi Medical Center, Rowland Medical Library, Jackson, MS; **Chameka C. Robinson, AHIP**, Head, Access Services, University of Mississippi Medical Center, Rowland Medical Library, Jackson, MS; **Susan B. Clark**, Director, Rowland Medical Library, Rowland Medical Library, University of Mississippi Medical Center, Jackson, MS

Objectives: Rowland Medical Library at the University of Mississippi Medical Center sought to inform its public about freely available NLM resources. Due to recent construction, foot traffic outside the library increased. The library promoted NLM resources to passersby using digital signage in the hallway outside the library. Additionally, users of the library's newly renovated nursing mothers room had access to LactMed through two new computers.

Methods: Funding from a NNLM Technology Improvement Award enabled the library to purchase equipment to publicize NLM resources. The computers were positioned in the nursing mothers room, and LactMed was available on the computers to promote breast milk health and to support the institution's "Baby-Friendly" status. First-time users of the nursing mothers room received a short survey including questions about increased knowledge of LactMed.

Two additional screens were connected to BrightSign software and installed in the heavily traveled hallway outside the library. To cultivate public awareness, NLM resources (LactMed, TOXLINE, Hazardous Substances Data Bank, and Dietary Supplement Label Database) were highlighted on a rotating basis on the screen. The library also promoted the same resources by hosting a NLM database "circuit training" session.

Results: Results from the nursing mothers room surveys indicate satisfaction with the addition of computers to the room. Although 25% of the respondents already knew about LactMed, 67% used the database while in the room and found the information helpful. Of the database training participants, 100% learned something new and plan to use what was learned in their practice or research. 60% of the participants said they would attend future sessions on NLM databases: either to explore different databases, or for a more in-depth demonstration of the same databases. The most popular databases suggested for additional training were TOXLINE and the Dietary Supplement Label Database.

Conclusions: Attendance and interest in the database training session and increased awareness of NLM resources for nursing mothers indicate success of the initiative. Use of LactMed in the nursing mothers room will continue to be assessed and future database training sessions with different resources will be offered.

Keywords: NNLM resources, technology, library space, nursing mothers, assessment, outreach

Poster Number: 112

Time: Monday, May 21, 2:30 PM – 3:25 PM

What's All The Buzz? A Library-Sponsored Spelling Bee

Rachel C. Lerner, AHIP, Research & Instruction Librarian, Quinnipiac University, Edward & Barbara Netter Library, Hamden, CT; **Jessica Kilham**, Research and Instruction Librarian, Edward and Barbara Netter Library, Edward and Barbara Netter Library, Hamden, CT; **Matthew Wilcox**, Director, Edward and Barbara Netter Library, Edward and Barbara Netter Library, Hamden, CT; **Gina M. Addona**, Access Services and Document Delivery Supervisor, Quinnipiac University, Edward and Barbara Netter Library, Hamden, CT

Objectives: To further integrate the library into campus life, staff held a spelling bee for the medical, nursing, and health sciences disciplines. The bee was intended to support literacy, contribute to student wellness and recreation, and align the library with campus programming. Ultimately, the goal was to create a fun, light-hearted competition for students and faculty, while sneaking in vocabulary development.

Methods: Vocabulary words were collected two ways: staff combed through medical texts and crowdsourced the collection on personal social media. A final list of 175 terms was separated into three tiers (easy, intermediate, & challenging). A dictionary of record was chosen for definitions and sentences were written for each term. A moderator was selected and given both phonetic keys and time to practice. An invitation was sent to students in early January 2017. Posters throughout the building and a slide on the digital display also advertised the event.

Results: 24 participants registered in advance; 4 people withdrew due to scheduling conflicts. The three major schools were represented, and faculty, staff, and students participated. Approximately 15 people attended the event as spectators. The winner and runner-up were both second-year medical students.

Conclusions: This program was a success; students requested that it be offered annually. Plans are underway for the next event.

Keywords: Programming, students, vocabulary, innovation, events, university, recreation

Poster Number: 113

Time: Tuesday, May 22, 1:00 PM – 1:55 PM

Prevalence of Publication in Predatory Journals at Emory University

Kimberly R. Powell, Research Impact Informationist; **Jeremy Kupsco**, Research Informationist; Woodruff Health Sciences Center Library, Atlanta, GA

Objectives: In 2017 the journal Nature published challenges to the assumption that research intensive U.S. institutions are immune to the hazards of predatory publishing. Sample articles from hundreds of potentially predatory journals were analyzed: the NIH was the most frequent funder and Harvard was among the most frequent institutions. Our study was designed to identify the publication prevalence at our institution.

Methods: Predatory publishers were defined using an archived version of Beall's list, a now defunct website that was widely recognized as the only comprehensive black list for potential predators. The archive was collected January 15, 2017 and reflects updates made 1 - 2 weeks prior. To identify our NIH publications, records were collected from PubMed Central using an institution search and limiting to 2011 - 2016 to reflect a five - year period covered by Beall's last update. PMC was selected under the assumption that direct journal inclusion in PubMed/MedLine serves as a proxy for quality. Journal and ISSN data were reference d against Ulrich's Periodical Directory to determine publishers. Data were then compared against the Beall's listing of potentially predatory publishers and standalone journals.
The publication costs for the predatory journals were used to determine the total amount of NIH funding used to pay for publications in predatory journals.

Results: The review of the University's Publications submitted to PubMed Central from 2011 to 2016 revealed 15090 publications. Of those 15090 articles 218 publications (1.4%) were from publishers that fell in Beall's list of predatory publishers. A review of publication fees for the publishers that University faculty published in revealed that approximately $300,000 dollars of Federal grant money was spent over the 5 year period publishing in predatory publications.

Conclusions: Previously, it was thought that publishing predatory journals was primarily a problem in developing countries. However, like the 2017 Nature study, we found that researchers publishing at Emory are publishing in journals that are considered predatory. While the rate of publication in predatory journals is low (1.4%) it did cost approximately $300,000 of Federal tax payer money, which amounts to approximately 70% of the funds of one year of the average NIH R01 grant.

Keywords: Scholarly Communication;
Predatory Publishing;
PubMed Central; Beall's List

Poster Number: 115

Time: Sunday, May 20, 2:00 PM – 2:55 PM

From Fee to Free: Making Interlibrary Loan No Charge (for Patrons)

Paul C. Gahn, Assistant Director/Assistant Professor, Electronic and Collection Services, University of Tennessee Health Science Center, University of Tennessee Health Science Center Health Sciences Library, Memphis, TN; **Caroline Fan**, Assistant Professor/Web Services Librarian, University of Tennessee Health Science Center, The University of Tennessee Health Science Center/Health Sciences Library, Memphis, TN; **Rick L. Fought, AHIP**, Director, University of Tennessee Health Science Center, Health Sciences Library, Memphis, TN; **Gwendolyn D. Jackson**, Associate Professor and Sr. RLS Librarian, University of Tennessee Health Science Center, Health Sciences Library, Memphis, TN; **Jennifer M. Langford**, Assistant Professor/Archivist and Special Collections Librarian, University of Tennessee Health Science Center, Health Sciences Library, Memphis, TN; **Zeb Mathews**, Assistant Professor/ Research and Learning Services Librarian, University of Tennessee Health Science Center, Health Sciences Library, Memphis, TN; **Alexandria C. Quesenberry**, Assistant Professor/Research & Learning Services Librarian, University of Tennessee Health Science Center, Health Sciences Library, MEMPHIS, TN; **Randall Watts, AHIP**, Associate Director/Associate Professor, Health Sciences Library, Memphis, TN; **Lin Wu, AHIP**, Assistant Director/Associate Professor, Research & Learning Services, University of Tennessee Health Science Center, Health Sciences Library, Memphis, TN

Objectives: An academic health sciences library decided to make interlibrary loan requests free effective Feb. 1, 2016. Previously it charged patrons $6.00 per request. This poster will show the resulting increase in requests, methods for coping with the increase and added value provided to the library and its patrons.

Methods: Statistics from the full year (2014-2015) prior to fee elimination will be compared to the full year after (2016-2017).
Copyright and ILL borrowing cost increases for 2016 and 2017 will be shown.
Functionality of technology implemented will be reviewed.

Results: Borrowing requests:
2013-2014: 1,383
2014-2015: 1,269
2015-2016: 2,396
2016-2017: 3,772
2017-2018: (through March):

Conclusions: Removing the barrier of an ILL fee has increased costs for the library. However, the benefit to removing the cost is that the library is better informed as to what campus constituents truly need to complete research and make clinical decisions. Understanding the content needs allows the library to make better collection management decisions and ultimately be more efficient with allocated funding.

Keywords: interlibrary loan

Poster Number: 116

Time: Monday, May 21, 2:30 PM – 3:25 PM

Leading the Way: A Study of the Value of Federal Medical Library Services

Priscilla L. Stephenson, AHIP, Chief, Library Service, Library Service, Library Service, Tampa, FL; **Edward J. Poletti, AHIP**, Chief, Learning Resources, Health Sciences Library, Learning Resources, Little Rock, AR; **Nancy A. Clark**, Director, Library Network Office, Library Network Office, Veterans Health Administration, Dallas, TX; **Mary Virginia Taylor**, Retired Librarian, Overton Brooks VA Medical Center, Library, Shreveport, LA; **Beatrice F. Nichols**, Chief Librarian, Medical Library, Library Program Office, Fort Sam Houston, TX; **Lisa R. Eblen, AHIP**, Associate Head Librarian, Naval Medical Center, Portsmouth, Library Services, Portsmouth, VA

Objectives: Health sciences library staff in federal agencies provide knowledge-based information to support their agencies' missions. They provide research support to help improve health outcomes, garner research support, and reduce costs. This study examined whether information seekers considered the information provided by their federal library staff to have positively affected patient care, research project development, or healthcare decision making.

Methods: There is little research on the value of information services in federal libraries. This study is based on earlier research of hospital library value, including 2 unpublished federal library studies conducted in 2010 and 2014. This project provided an opportunity for those receiving research assistance from federal health sciences librarians to evaluate the impact of that information on their work. Medical library staff from 3 federal agencies provided an online survey to library customers receiving reference assistance during a 3-month study period, July through September 2017. The combined responses of 1,110 clinical patient care providers, researchers, and health administrators represented more than 76 facilities. The survey tool was designed to capture immediate evaluations of the value of material provided in response to the requestors' specific reference questions. Results were reported in the aggregate as well as by agency and facility.

Results: Information requests were in support of patient care (33.09%), research (29.81%), education (25.71%), and administrative work (11.39%). Of the patient care requests, respondents said library research had improved patient management (60.11%), avoided adverse events (11.08%), limited the need for additional tests or procedures (13.57%), and could result in reduced costs (45.71%). Clinical staff estimated that library support for their research saved them 3,445 hours. Research information requests had the potential to result in requestors obtaining more than $500,000 in funding (17.55%). Overall, 98.87% of respondents received new or updated information.

Conclusions: The survey documented the value of medical libraries to federal agencies in terms of improvements for patient care, costs avoided or reduced, staff time saved, and funding application support. Reference support provided by federal health science libraries was judged to be pertinent to the respondents' specific requests, answered their questions, and provided new or updated information

Keywords: Hospital libraries, Value, Critical incident, Survey

Poster Number: 117

Time: Tuesday, May 22, 1:00 PM – 1:55 PM

Provision of Research Assessment Services in Academic Health Sciences Libraries

Yingting Zhang, AHIP, Research Services Librarian, Rutgers University, Rutgers University Libraries, RWJ Library of the Health Sciences, New Brunswick, NJ

Objectives: Academic health sciences libraries have been providing research support services. However, most did not include research assessment services until recently when such services become demanded. This study aims to investigate if, what, and how research assessment services are provided by academic health sciences libraries. The findings will help librarians develop effective research assessment services to meet researchers' needs.

Methods: A list of health sciences libraries (N=150) was downloaded from AAHSL Membership Directory at http://www.aahsl.org/index.php?option=com_community&view=search&searchId=117383 in January 2018. Data were extracted from their websites mainly in the areas of research services, classes, workshops, special programs and library guides aiming to answer these questions: 1) Are research assessment services being provided? 2) If yes, what levels of assessment services are provided? 3) How are the research assessment services provided? 4) What other research support services are provided? When a website did not post enough or clear information, contacting listed point persons was pursued to acquire additional information and clarification about the service. Data collected were recorded in an excel file and analyzed accordingly.

Results: The study revealed that 60 out of 150 libraries (n=150, 40%) provide research assessment services at various levels. Among them, 21 libraries provided basic level with a brief guide on research metrics; 22 offered intermediary level with detailed guides/workshops to measure research impact; 17 provided formal services by designated professionals and special programs. 18 (n=150, 12%) have access to the services provided by other libraries in their systems. 67 libraries (n=150, 45%) did not have such services; 5 (n=150, 3%) were excluded due to various reasons. The study also showed other research services offered in the studied libraries.

Conclusions: More academic health sciences libraries than expected provided information on research assessment in addition to many other types of research support services. However, most libraries provided the research assessment services at a very limited level except a small number of libraries that offer very formal bibliometric services to their users. It would be beneficial for these providers to share what lessons they learned and what programs proved to be effective. Probably the subsequent study is to survey these libraries and service providers to make recommendations on best practices.

Keywords: Academic Health Sciences Libraries, Bibliometric Services, Bibliometrics, Research Assessment Services, Research Evaluation Services, Research Metrics, Research Support.

Poster Number: 118

Time: Tuesday, May 22, 2:00 PM – 2:55 PM

Engaging the University Community Utilizing a National Library of Medicine Traveling Exhibit

Rachel C. Lerner, AHIP, Research & Instruction Librarian, Quinnipiac University, Edward & Barbara Netter Library, Hamden, CT; **Gina M. Addona**, Access Services and Document Delivery Supervisor, Quinnipiac University, Edward and Barbara Netter Library, Hamden, CT**; Lynn Sawyer**, Technical Services Assistant, Quinnipiac University, Arnold Bernhard Library, Hamden, CT

Objectives: To contribute to the wellness and intellectual life on campus, the Library booked the NLM Harry Potter's World exhibit. Campus Libraries created 14 events/exhibits to compliment the panels. The project goals were to provide historical context to renaissance science and medicine through the lens of Harry Potter, and to use Harry Potter as a touchstone to discuss current medical topics.

Methods: Two campus libraries shared the exhibit, which spent three weeks at each location. All students were invited to participate regardless of their library affiliation. The 15 events/exhibits included two opening receptions with the campus mascot, three lectures (two from campus faculty, and a keynote speaker from an outside organization), a Harry Potter medical bibliography, a movie night, six educational curated displays, a streaming video display, and a therapy horse demonstration. A complimentary online exhibit mirrored the physical displays and included video of the lectures. The libraries partnered with student groups, student affairs, facilities, public safety, and public affairs to help organize, fund, facilitate, and promote the programs. Marketing efforts included a marauder's map (take-away program flier), posters, table tents for the cafeteria, digital display slides, announcements via the student intranet, advertisements in the library newsletter, and targeted departmental advertising.

Results: By far, the four most attended and well-received events were the therapy horse meet-and-greet (care of magical creatures), the Harry Potter movie night, the opening receptions, and the Poisons lecture (defense against the dark arts). The library-curated exhibits were all well received, with anecdotal commentary to the coordinators such as, "very educational," "super interesting," and "Oh! I love this!" The National Library of Medicine exhibits were well attended, with student, faculty, and staff interest (the library gate counts reflect the increased foot traffic). University members brought their families to events and exhibits as well.

Conclusions: Students, staff, and faculty of all ages were excited, engaged, and able to relate to the content. One professor allowed students to go to a lecture in lieu of class. Other allowed students to use the events as part of a course requirement. The two library directors appreciated database advertisement and the opportunity to redirect students to library resources for further information. This was a worthwhile project that added to the university community. We will likely do something like this again in the future, now that we have best practices for our institution.

Keywords: NLM, exhibit, university, community, engagement, interprofessional, students

Poster Number: 119

Time: Sunday, May 20, 2:00 PM – 2:55 PM

From Passive to Active: A New Model for Library Orientation

Nancy A. Bianchi, Health Sciences Librarian, Dana Medical Library, University of Vermont–Burlington; Gary S. Atwood, Research and Instruction Librarian, University of Vermont, Dana Medical Library, Burlington, VT

Objectives: This poster shares the experience of academic librarians turning a traditional, passive library orientation at a Graduate Medical Education Fair for new residents into an active learning activity.

Methods: Every summer, new residents enter postgraduate medical training programs at the University of Vermont (UVM) Medical Center. In an effort to introduce the library early in their clinical careers, liaison librarians have participated at a Graduate Medical Education Fair since 2012. In the past, the library's orientation activity consisted of a table full of paper handouts, staffed by overzealous librarians. Feedback from the residents, however, revealed that they politely collected the paperwork but frequently used or understood little of its content. In 2016, the library orientation for new residents was completely redesigned and updated. The traditional library handouts were replaced by an active learning exercise centered around a poster that highlighted the essential resources and services provided by the library. Residents were asked to spend just 1 minute reading through the library poster, and then invited to complete a brief poster survey.

Results: Results of this new orientation format were quite revealing. Completed poster surveys were returned by more than 85% of the residents, showing that they liked the poster format and its effectiveness in introducing the library. The poster also prompted many questions and interesting conversations among residents and librarians right on the spot. The simple design of the survey questions encouraged residents to identify what they liked and wanted to learn more about the library. In addition, the survey fostered a perfect opportunity to ask questions about anything important that was included or missing from the poster.

Conclusions: Librarians plan to continue to use this poster driven learning activity, to study its results, to modify its content when appropriate, and to build on its success in other library presentations.

Keywords: Library Orientation; Residents and Fellows; Graduate Medical Education; Poster; Survey

Poster Number: 120

Time: Monday, May 21, 2:30 PM – 3:25 PM

Provision of a Library Bioinformatics Service to Support Medical Research

Peter R. Oxley, Associate Director of Research Services; **Terrie Wheeler**, Director; Weill Cornell Medicine, Samuel J. Wood Library and C.V. Starr Biomedical Information Center, New York, NY

Objectives: Genomics research and precision medicine continue to become more prevalent in medical research and clinical practice. Yet not all departments are able to support bioinformatics staff. Thus, to provide support for medical and research bioinformatics in a medical college setting, our library hired a research scientist with genomics and bioinformatics experience, to establish teaching and consultation services in bioinformatics.

Methods: In 2017, we established a weekly demonstration hour in the library, showcasing databases and analytical techniques, and hosted specialty workshops for bioinformatics software and data visualization. Concurrently, we offered a consultation service for one-to-one assistance with data analysis, project preparation, data management and visualization. We also became members of the Data Carpentry organization, to equip librarians and research support staff to teach data science workshops to students and staff.
To supplement this service, we have also created a scientific software hub, providing centralized access to tutorials, documentation, and registration for scientific software licensed or supported by the college.

Results: In six months, we have offered 17 demonstrations and six workshops, with up to 27 people in attendance at each session, including medical and graduate students, research staff, and faculty. We have provided 28 consultations (including 9 ongoing projects), which required 58 hours of data analysis.
The scientific software hub has received 284 license requests in its first seven months, and has enabled the consolidation of two software products and one database widely used at the college, but previously individually licensed. An additional three software licenses are undergoing recruitment of interest and budgeting for provision as a site-wide license.

Conclusions: Although still in its infancy, the library bioinformatics program has shown consistent growth, and we anticipate the Data Carpentry courses will be well received (based on a previous pilot program). Positive feedback indicates the service is successfully meeting a real need within the college, and is perceived to provide real value to our clients. Future developments include the expansion of the service to include expertise for collaboration in precision medicine initiatives and more programs that will be of direct benefit to clinicians.

Keywords: bioinformatics, research, precision medicine, genomics, data management, analysis, data visualization

Poster Number: 121

Time: Tuesday, May 22, 1:00 PM – 1:55 PM

Reading Today, Leading Tomorrow: An Interprofessional Education Book Club

Rachel Keiko. Stark, AHIP, Health Sciences Librarian, Library, California State University, Sacramento University Library, Sacramento, CA; **Donna Jensen**, Assistant Professor, CSU, Sacramento, Department of Gerontology, Sacramento, CA

Objectives: The Objective of the Interprofessional Education Book (IPE) Club was to provide students with an opportunity to participate in an IPE activity with a focus on aging and older adults outside of the classroom and to work with faculty from various departments. This project utilized two validated measurements to assess participants' attitudes towards aging and older adults, and IPE.

Methods: Working together, a gerontology professor and health sciences librarian developed a time line for the book club, choose the book to be read, and chose the measurements that were used to assess whether the book club improved participants understanding of IPE and if participant attitudes toward aging and older adults changed during their participation in the book club. This project was approved by the university IRB, and permission to use and modify the Interprofessional Attitudes Scale (IPAS) and the Older Adult and Visual Analog Scale (At-O-A) was obtained. Advertising included branded posters, email outreach to students and faculty, and direct appeal to students enrolled in Gerontology classes and student workers at the University Library. Participants were asked to complete a pre/post modified version of the IPAS and At-O-A scales. After completion of the program, the pre/post measurements were analyzed using statistical software.

Results: Results are not currently available as our research is not yet complete.

Conclusions: Our research is not yet complete.

Keywords: Book Club, Interprofessional Education, IPE, Gerontology, Aging, Collaboration, Library Space

Poster Number: 122

Time: Tuesday, May 22, 2:00 PM – 2:55 PM

Librarians as Health Advocates in Health Fairs

Jahala Simuel, Medical Librarian, College of Nursing and Allied Health Liaison, Louis Stokes Health Sciences Library, Clayton, NC; **Fatima M. Mncube-Barnes**, Executive Director, Howard University, Howard University, Arlington, VA

Objectives: Health fairs are convenient avenues for health professional students to recruit patients for class requirements. Inter-professional collaboration engages and enriches the experience of the students and also foster relations with neighboring communities. This effort is a good health preventative tool for communities. Analyzed data will help improve our health fairs and identify the most vulnerable individuals who need hospital referrals.

Methods: As a member of the National Network of the Library of Medicine, our library is a resource for medical, dental, pharmacy, nursing and allied health sciences students and faculty. It is also a health information resource for individual patients, healthcare providers and researchers globally. Promoting health literacy in our neighboring public housing apartments and city's Housing Authority engages inter-professional health students to improve health literacy on patient's well-being. The Health Sciences librarians partner with student health fair coordinators, health sciences faculty and students to promote health literacy. Given the low socio-economic status of our neighboring communities, communication strategies are tailored to the participant's needs and abilities.

Results: Analyzed data will help librarians improve health information pamphlets. Negative health screenings of the most vulnerable individuals are referred to respective health professional. The health fairs are the best platform for students to learn from each other and also recruit patients to fulfill their discipline requirements.

Conclusions: Through this study, health sciences librarians use health fairs to foster collaboration with students and faculty in the health sciences. The librarians partner with health fair coordinators to address health needs of the Public Housing for the Senior and Disabled, located across the street from the University. Communication strategies will be tailored to the needs and abilities of the participants.

Keywords: health fairs, literacy, communities, students, clinicians, collaboration, inter-professional

Poster Number: 123

Time: Sunday, May 20, 2:00 PM – 2:55 PM

Growing ORCID

Merle Rosenzweig, Informationist; **Chase Masters**, Informationist; **Tyler Nix**, Informationist; Taubman Health Sciences Library, Ann Arbor, MI

Objectives: As an open, non-profit, community-based effort, ORCID (Open Researcher and Contributor ID) creates and maintains a registry of unique research identifiers and a transparent method of linking research activities and outputs to these identifiers. The objective of this poster is to trace ORCID from its launch on October 16, 2012 to a globally accepted author ID tool.

Methods: Data collected includes: number of ORCID IDs created; adoption by universities, publishers, and funding agencies; and use by international communities. Researchers and scholars face the ongoing challenge of distinguishing their research activities from those of others with similar names. The ORCID is indicated as a persistent URL with a 16-digit machine-readable identifier that distinguishes an individual scientist and author in much the same way that a Digital Object Identifier (DOI) uniquely identifies a paper, book, or other scholarly publication. If a researcher publishes under a professional maiden name rather than a married name, has grants under various forms of their name, or has a common name such as Smith or Brown, an ORCID assures that all of their scholarly output is assigned to them. The ORCID also enables a researcher to have a digital CV that can be update regularly.

Results: ORCID is unique in its ability to reach across disciplines, research sectors and national boundaries. Publishers have incorporated ORCID into their manuscript submission system; funding agencies are integrating it into the grant application process, reporting workflows, and implementing a single sign on; it is being assigned to data sets; and inserted into citations in online databases, among others uses. The information in an ORCID record is controlled by the individual; they are able to determine its content and control access to information displayed.

Conclusions: The acceptance and impact of ORCID can be seen by the increase in the number of IDs created from its launch in 2012. In October 25, 2012 there were 6,083 and as of January, 2018 the number reached 4,335,567 and is growing. The various languages that the interface has been translated into demonstrate the acceptance by the international community. Among the languages are Portuguese, Korean, Italian, French, Czech, Chinese (simplified and traditional), Russian, Spanish, Arabic, and German.

Keywords: Author identification, Author disambiguation, Open Researcher and Contributor ID, ORCID

Poster Number: 124

Time: Monday, May 21, 2:30 PM – 3:25 PM

2017 Inaugural Celebration of Yale School of Nursing Authors

Melanie J. Norton, Head of Access and Delivery Services, Cushing/Whitney Medical Library, Cushing/Whitney Medical Library, New Haven, CT; **Janene Batten**, Nursing Librarian, Yale University, Cushing/Whitney Medical Library, New Haven, CT; **Alyssa Grimshaw**, Access Services/Clinical Librarian, Yale University, Access and Delivery Services/Clinical Information Services, New Haven, CT

The Yale School of Nursing (YSN) Librarian and the Dean of the Nursing School conceptualized an event to celebrate YSN book authors and editors. Book authors have been under-celebrated in the YSN community. The objective for the event was to showcase and celebrate authors of books published in the past 5 years. Methods: A call to authors went out to faculty, students, and alumni of Yale University's School of Nursing . 9 authors responded with titles of their books and an additional 4 authors were discovered through a search of current faculty. A save the date was sent to YSN community. Books not owned by the library were purchased to be included in the library's collection. A display of books was exhibited with printed labels identifying author names, book title, and year of graduation if they were Alumni. An Invitation to the event was sent to authors and the School of Nursing community.

Poster Number: 125

Time: Tuesday, May 22, 1:00 PM – 1:55 PM

Recovery-Oriented Collection Development: Results of a Qualitative Study on Mental Health Information Needs in a Patient Library

Riley Saikaly, Research Analyst, Centre for Addiction and Mental Health, Office of Education, Toronto, ON, Canada**; Alexxa Abi-Jaoude**, Research Coordinator, Centre for Addiction and Mental Health, Office of Education, Toronto, ON, Canada; **Genevieve Ferguson**, Research Analyst, Centre for Addiction and Mental Health, Office of Education, Toronto, ON, Canada**; Sharon Bailey, AHIP**, Manager, Centre for Addiction and Mental Health, Library Services and Archives, Toronto, ON, Canada; **Andrew Johnson**, Manager, Centre for Addiction and Mental Health, Office of Education, Toronto, ON, Canada

Objectives: To understand the health information needs of inpatient and outpatient mental health clients and their families at a mental health and addictions hospital and to determine how a hospital-based consumer health library for inpatients and outpatients can best facilitate the uptake of consumer health information to support recovery.

Methods: Our qualitative approach included one-on-one semi-structured interviews with clients (n= 11), families (n= 8), health care providers (n= 7), as well as two focus groups with library volunteers (n= 7) at the hospital. A total of 33 individuals participated. A pilot collection of books was presented in these sessions to encourage discussion about the types of resources that are most relevant for recovery.

Results: Participants expressed a need for multiple formats (e.g., online, print, face-to-face), holistic perspectives to recovery, and accessible resources (e.g., language, cognitive ability). Though informational needs were well articulated, it was evident that the inpatient library, how it currently operates, may not be the preferred venue for accessing health information. A majority of participants expressed using the library solely for recreation and relaxation. Despite its availability, participants addressed barriers to utilizing the space in general. These included a need to prioritize marketing and promotion of the space and available programming; consistent hours; connection to community; and development of an online presence.

Conclusions: Our findings uncovered intriguing revelations around the meaning of the inpatient library for clients and families in a mental health setting and the need to meet both their informational and recreational needs. Participants' perspectives will help to inform both the quality improvement of the current inpatient library space, as well as to think ahead to the development of a future patient and family learning centre.

Keywords: consumer health information
co-creation
patient libraries

Poster Number: 126

Time: Tuesday, May 22, 2:00 PM – 2:55 PM

Two Years of Library Participation in a Health Sciences Summer Camp

Lauren Wojcik, AHIP, Medical Librarian, Austin, TX, Austin, TX; **Darlene Ennis, AHIP**, Medical Librarian, Dell Children's, FRC/Medical Library, Austin, TX

Objectives: A newly-developed hospital-based summer camp for young people interested in the health professions provided an outreach opportunity for the hospital's medical librarians. Program participants spent four days interacting with healthcare professionals in a clinical simulation lab setting. The medical library utilized its time in the camp schedule to develop skills related to locating and evaluating health information using the internet.

Methods: The library participated in this summer camp in 2016 and 2017. In preparation for the first summer, hospital librarians customized an existing health literacy curriculum to meet the learning objectives of their institution's program. They developed teaching materials and handouts for both middle school and high school groups. As a result of student feedback and further research, librarians revised the course in 2017 to promote active learning and enhanced student engagement.

Results: In 2016, librarians presented a 45-minute course to three groups of middle school-aged youth and two groups of high school-aged youth, with a total of 130 participants. Participants were highly satisfied with the camp in general, but had mixed feelings about the library's course. In 2017, librarians facilitated five, 45-minute active learning sessions. A total of 75 students participated in these sessions. Student engagement and satisfaction improved with utilization of this revised curriculum. Students also reported improved confidence in their abilities to use the internet to find accurate, reliable health information.

Conclusions: The camp has been well-received within the community and reached maximum enrollment both summers. This project provided an opportunity for the library to become involved with a revenue-generating project while also demonstrating a commitment to working with other hospital departments in support of the organization's mission and goals.

Keywords: summer camp, hospitals, consumer health, active learning, outreach, revenue

Poster Number: 127

Time: Sunday, May 20, 2:00 PM – 2:55 PM

Hospital Library Benchmarking Study

Angela Spencer, AHIP, Manager, Medical Library, C. Alan McAfee, MD Medical Library, Chesterfield, MO; **Elizabeth Mamo**, Library Director, Rochester Regional Health, Rochester Regional Health, Rochester, NY**;** **Brooke Billman, AHIP**, Medical Librarian, College of American Pathologists, Northfield, IL

Objectives: To assess the current landscape of hospital libraries by collecting benchmarking data from hospital librarians in the U.S. and other countries. Since the last MLA benchmarking survey published in 2006 hospital libraries have faced significant changes including downsizing, position and library elimination, and hospital mergers. This survey provides information to inform the development and implementation of effective advocacy for hospital libraries.

Methods: A web-based, anonymous survey was designed to collect information from hospital librarians representing stand-alone hospitals and hospital systems. Hospital library benchmarking surveys, including the previous MLA surveys, were reviewed and applicable questions were included.
The 57-question survey was distributed via select list servs, targeting the US and Canada but open to any country. The topic areas covered hospital/health system, library, and library staff demographics; library characteristics and scope of service; interlibrary loan and document delivery; library funding; and library budget.

Results: There were a total of 180 respondents but the total number of responses for each question varied. Analysis shows that, of the responding libraries, 67% are part of a hospital system; 24% having merged with or were bought by another hospital or health system and 37% have acquired 1-5 hospitals in the last 10 years; 78% are not for profits; 47% have 1,000-5,000 FTE in the organization; 57% have one library; 48% have 1 FTE librarian, 35% have 2-5; 82% do not or are not able to use social media; 61% don't have strategic plans; 66% belong to a consortium; 48% provide up to 250 search requests a year; 66% do not receive funding outside of their organization; 33% have budgets for print books totaling less than $1,000; 30% have budgets less than $100,000 and 10% have budgets over $1M.

Conclusions: These findings contribute to the field's knowledge of current hospital library demographics and services. The results suggest implications regarding staffing and the depth of services within each unique setting, especially rapidly expanding health systems. Librarians can use this information for strategic planning and with hospital administrators to justify library budget and staffing decisions.

Poster Number: 128

Time: Monday, May 21, 2:30 PM – 3:25 PM

On the Road Again: Improving Health Education by Logging 3,300 Miles of Windshield Time to Meet with Nursing Students and Faculty at Remote Locations across Montana

Mary Anne Hansen, Professor/Health Sciences Librarian, Montana State University, Bozeman, MT

Objectives: Two land grant university librarians recognized that a divide remains between on-campus users and distance users; thus they are working to blur the lines of this divide by providing virtual support as well as taking services on the road across thousands of miles and meeting F2F with librarians and health sciences students and faculty located across Montana.

Methods: This university has a growing online Nursing program with remote sites in four locations; additionally, this library collaborates with tribal college librarians distributed across Montana. After several years of implementing virtual solutions to meet users' research needs and to build relationships through such means as email, web conferencing and telephone, those virtual interactions surfaced a desire among distributed students and faculty for face to face interactions with librarians. To that end, these librarians developed a successfully-funded Community Health Outreach proposal to travel to all four branch campuses and to also extend their reach to the tribal colleges in the state - while continuing to provide virtual reference services and research consultations. The librarians are measuring how merging educational technology with more traditional interactions in the provision of library instruction and services impacts health sciences programs statewide.

Results: Two land grant university librarians amassed 3500 miles during five road trips to visit 13 libraries and health sciences departments. They consulted with librarians and faculty about the health information needs at their colleges and in their communities. They also provided programming to numerous health sciences students and faculty at the remote Nursing campuses and tribal college libraries they visited, while also learning about initiatives and programming happening at each of those locations. They were able to extend the reach of the Regional Medical Library and the National Library of Medicine in their Liaison roles in their state.

Conclusions: These librarians strengthened relationships with health sciences faculty at remote Nursing campus locations and tribal colleges libraries across their state. Additionally, they provided health outreach to traditionally underserved tribal communities. On-site visits helped greatly to solidify their liaison role with remote Nursing faculty and others. Communication with remote constituencies. was greatly improved as a result of these in-person visits. Relationships were enhanced with medical librarians at the remote Nursing campuses. Finally, evidence was found that merging educational technology with face to face interactions in the provision of library instruction and services can positively impact health sciences programs statewide.

Keywords: tribal communities, virtual services, on-site interactions, outreach, distributed nursing campuses

Poster Number: 129

Time: Sunday, May 20, 2:00 PM – 2:55 PM

Mapping the Competencies: Association of College and Research Libraries to Core Entrustable Professional Activities and Accreditation Council for Graduate Medical Education

Emily Brennan, Research and Education Informationist, Medical University of South Carolina, Library, Charleston, SC; **Heather Collins, AHIP**, Assistant Director, Research and Learning Department, Archie R. Dykes Health Sciences Library , A.R. Dykes Health Sciences Library, Kansas City, KS; **Kelly Thormodson**, Director, Library Resources, University of North Dakota, School of Medicine and Health Sciences University of North Dakota, Grand Forks, ND; **Nancy E. Adams**, Director of Foundational Medical Sciences, George T. Harrell Health Sciences Library, Penn State College of Medicine, Hershey, PA; **Iris Kovar-Gough, AHIP**, Health Sciences Librarian, Michigan State University, Michigan State University Libraries, East Lansing, MI; **Joey Nicholson**, Education and Curriculum Librarian, NYU Langone Medical Center, NYU Health Sciences Library, New York, NY; **Rikke Sarah. Ogawa, AHIP**, Director, Louise M. Darling Biomedical Library and Science and Engineering Library, Louise M. Darling Biomedical Libary, UCLA, Los Angeles, CA; **Neil Rambo**, Director, NYU Health Sciences Libraries, NYU Health Sciences Library, New York, NY; **Judy M. Spak**, Assistant Director, Research and Education Services, Yale School of Medicine, Cushing/Whitney Medical Library, New Haven, CT; **Megan von Isenburg, AHIP**, Associate Dean, Medical Center Library & Archives, Medical Center Library, Durham, NC; **Elizabeth R. Lorbeer, AHIP**, Chair, Department of the Medical Library, Western Michigan University Homer Stryker M.D. School of Medicine, Kalamazoo, MI

Objectives: To aid librarians in advocacy and instruction, the Association of Academic Health Sciences Libraries (AAHSL) task force on competency-based medical education was asked to map medical education competencies to the ACRL Framework for Information Literacy.

Methods: Task force members individually mapped relevant EPAs (7, 9, 13) to the ACRL Framework and compared mapping results. For EPA items, where inter-rater agreement was low, task force members discussed the EPA item in question and came to a consensus on corresponding item(s). After establishing standards, task force members were assigned in dyads to map two additional standards documents (2017-18 LCME Functions and Structures of A Medical School and ACGME Milestones) to the ACRL Framework. Any areas of disagreements between raters within a dyad were resolved through discussion with a third member. Applying the Framework structure to undergraduate and graduate medical education standards reveals areas for improvement in information literacy in medical education. Reviewing and mapping education standards to the Framework also can aid the library community in framing information literacy education within the larger picture of medical education competencies.

Keywords: medical education, Core EPAs, ACRL Framework, instruction, mapping

Poster Number: 130

Time: Tuesday, May 22, 2:00 PM – 2:55 PM

Veteran Voices: Library Impact on Veterans

JJ Pionke, Assistant Professor and Applied Health Sciences Librarian, Social Science, Health, and Education Library at the University of Illinois Urbana Champaign, Social Sciences, Health, and Education Library at UIUC, Champaign, IL

Objectives: This poster discusses the impact that the library has had on veterans through a series of interviews with veterans from many different time periods that included peace and conflict. Highlighted are responses to questions that revolved around veteran views of libraries and reading in general.

Methods: Veterans were interviewed over the course of four months in 2017. The interview questions were added to a larger question set around veteran experiences and the interviews themselves were often deposited in the Library of Congress as part of their Veterans History Project. Interviews lasted anywhere from 30-90 minutes and were audio recorded. Veteran responses to the library and reading questions were transcribed by a graduate assistant. After the interviews were complete, the transcriptions were then analyzed for common themes.

Results: Surprisingly, veterans often stated that they didn't want any special programming or focus on military history. Veterans that had children often used the library because they were parents. Veteran views towards libraries were largely positive, even if the library was not used often.

Conclusions: Libraries have been very concerned with reaching out to the veteran population but for the most part, veterans want to be considered as just another user of the library and not as a special population. Veterans are generally proud of their service but typically do not make their service the main focal point of their lives and they do not want libraries making it the key factor in outreach to them.

Keywords: veterans, oral history, interviews, library use, reading

Poster Number: 131

Time: Tuesday, May 22, 2:00 PM – 2:55 PM

Developing Library User Personas for Strategic Planning and Advocacy

Emily J. Glenn, AHIP, Education and Research Services Librarian; **Christian Minter**, Community Engagement and Health Literacy Librarian; McGoogan Library of Medicine, Omaha, NE

Objectives: An academic medical center library employed the design thinking approach to better understand the roles of people in the library community and their current needs. With a future building renovation and shifting library services, it was time to refresh our impressions about what users are thinking, feeling, and doing concerning collections, education and research support, space, special collections, and outreach.

Methods: Two librarians conducted semi-structured interviews with 21 people representing six groups: faculty, students, post-docs, clinicians, support staff, and patient representatives. The questions elicited storytelling about the participants' motivation, purpose, priorities, approaches to collaboration, information-seeking behaviors and barriers, perceptions of space and environment, technology interests, perceptions of libraries, and lifestyle. Librarians analyzed the interview transcripts, then coded the interview text to twelve major themes.

A free software product, Xtensio, was used to design a visual representation of each persona. The following sections were included: a headshot, demographics, a “who am I?” quote, up to five personality keywords, a list of goals and frustrations, a short biography, a scale of motivating factors, lifestyle brands, library products and services used, and a statement that describing what the library should do or be.

Results: Based on the interviews and feedback from the library strategic planning teams, six individual personas were developed. These personas helped the library's strategic planning teams to think broadly about future services, collections, outreach approaches, and spaces for the communities the library serves. The results were presented to library staff, followed by discussions about potential bias, requests for expansion of anecdotes, and suggestions for improving the product.

Conclusions: This "shoestring budget" project helped our library answer questions about users and non-users that had not been asked in recent memory and also provided some valuable secondary information. The content of the interviews was used to help develop focused questions for the stakeholder interview phase of renovation planning. The most vocal storytellers were those who had familiarity with the library and perceived us as an ally; those who wanted to lodge a complaint; and novice users were curious about a product or service. This project revealed immediate opportunities to improve advocacy for the library and to promote services.

Keywords: Audience awareness
Strategic planning
Design thinking
Library users

Poster Number: 132

Time: Monday, May 21, 2:30 PM – 3:25 PM

Communicating the Concept and Value of Open Science

Kendra Godwin, Research and Data Librarian / NLM Associate Fellow, NYU Langone Health, NYU Health Sciences Library, New York, NY

Objectives: Open science is often discussed, but not well-understood. Future library planning surrounding policy development, scholarly communications, and education efforts involves being able to effectively communicate what open science is. The goal of creating an introductory open science framework is to inform discussion on how institutions could conceptualize, discuss, and promote open science.

Methods: Creating this framework required an exploration of the open science ecosystem. Methods included 1) conducting a literature review; 2) gathering examples of its benefit; and 3) organizing informal conversations with thought leaders. The literature review produced an example set of open science definitions that is representative of different groups, and shows how different institutions that are committed to furthering open science formally define the term and find meaning. The examples show how open science advances science and benefits healthcare in ways that would not be possible if processes were closed. The thought leaders are individuals within the field of open science, many dedicating their recent careers to its exploration and promotion, as well as open policy, data science, data, and librarianship. These conversations helped guide how open science could be understood and furthered in different areas.

Results: What emerged from reading and conversation was variation, that open science is a creation of promotion and purpose for whomever is using the phrase. It is a dynamic concept with no universal understanding or singular definition. Open science is, but is also far more than, just the sharing of research data. Key characteristics: involvement with any or all parts of the research life cycle; a process rooted in and relying on digital technologies; collaboration with people at different levels and in differing fields; and conducted in a way that will allow for sharing and reuse. Many organizations are in the information gathering stage surrounding open science, and there is increased interest from agencies that shape and fund science.

Conclusions: Open science is a concept worth exploring and promoting, but audience communication is important. Individual researchers react best to a simple approach that utilizes examples, visuals, and key points, and focuses on the personal connection. Takeaways are on funding, resources and tools, and how it relates to careers. Within an institution, the focus is on how open science complements its mission. Any conceptualization of open science, including the building of new connections or initiatives for its advancement, must align with and strengthen current work.

Keywords: open science, open access, open data, policy, publishing, research

Poster Number: 134

Time: Tuesday, May 22, 2:00 PM – 2:55 PM

A Paradigm Shift: Transforming Collection Development to Meet the Needs of Users

Megan Inman, Collection Development Librarian, East Carolina University, William E. Laupus Health Sciences Library, Greenville, NC; **Marlena Barber**, Assistant Director of Collections & Historical Services, Laupus Health Sciences Library, Greenville, NC

Objectives: For the past few years, this health sciences library has been ordering eBooks in addition to ordering books in print. Over time, the library has experienced a shift in increased eBook acquisitions versus print. The purpose of this study is to analyze expenditures and trends in usage by format and acquisition model to better inform collection development decisions.

Methods: This study will examine the expenditure data in combination with eBook and print usage for the past six years. Expenditure data will include approval plan purchases, standing orders, firm orders, and Patron Driven Acquisition (PDA) purchases. Usage data will include print circulation, eBook usage of perpetually-owned titles, and PDA usage. All raw data will be exported and analyzed using Microsoft Excel. Tables will be created to examine financial data, print circulation, and eBook reports by usage type for each fiscal year. Following this, trends will be identified and reported accordingly.

Results: Print book usage at Laupus Library has seen a decline since 2012, whereas eBook usage has shown a large increase. Total book expenditures indicate a higher overall spend in print materials since 2012. Year to year book expenditures have decreased overall for print and eBook materials with more of the budget emphasis placed on print formats, but recent years have shown an increase in eBook purchasing over print books.

Conclusions: This study has reinforced our current collection development policy and supported our trends in purchasing decisions. The use of eBooks provides many advantages including increased accessibility and the ability to provide more comprehensive statistical data. Limitations of this study are that subscription eBooks platforms were not included. Usage of this type of product when reviewed for assessment purposes has typically been substantial.

Keywords: Collection Development, Usage Statistics, Budget, Expenditures

Poster Number: 135

Time: Sunday, May 20, 2:00 PM – 2:55 PM

Impact of a Collaborative Evidence-Based Practice Nursing Education Program on Clinical Operations

David E. Coleman, Medical Librarian/Informationist, Medical Library Services / Hawaii Pacific Health, Honolulu, HI

Objectives: Describe the collaborative program, the definition, structure, process of Evidenced Based Practice and examples of the integration into and impact of successful EBP projects on the clinical operations of Hawaiʻi health facilities.

Methods: Provide workshops to educate practicing nurses in the concepts, strategies, development and implementation of evidence based programs, policies and procedures.

Results: Program delivery to over 300 nurses has led to the development and implementation of several nurse-driven projects that have demonstrated a positive impact on clinical operations within Hawaiʻi health facilities and resulted in several peer-reviewed publications.

Conclusions: Application of collaborative Evidence Based Practice focused education and information literacy programs for professional nurses can result in improved care quality, patient outcomes and nurses perception their own knowledge and skills.

Keywords: Evidence Based Practice, Information Literacy, Clinical Nursing, Professional Development

Poster Number: 136

Time: Monday, May 21, 2:30 PM – 3:25 PM

Video Productions at Saint Joseph Hospital: A Teaching Hospital in Denver, CO

Karen Wells, Manager, Libraries and Knowledge Services, Libraries and Knowledge Services, Libraries and Knowledge Services-Clinical Research Library, Denver, CO**; Tim Rockwood**, Multi-Media Service Coordinator and Data Analytics, Saint Joseph Hospital, Libraries and Knowledge Services-Clinical Research Library/Multi-Media Services, Denver, CO; **Eric Neail**, Multi-Media Technician and Video Productions Specialist, Saint Joseph Hospital, Libraries and Knowledge Services-Clinical Research Library/Multi-Media Services, Denver, CO

Objectives: The purpose was to assess a new video production service, through the Library's Multi-Media (MMS) Department, compiling 2 years of data. Assessment included impact of internal video productions on educational/training programs (GME and Nursing Education), on other hospital departments, and on the reduction of cost of outsourced video productions, through our limited/low budget program.

Methods: Data was collected through automated statistical-gathering tools utilizing Vimeo and HealthStream, or via manual record keeping on MP4s for laptops. Information covered:

• Video Production Requests

-----Total annual number; by hospital department and by video subject content

• Video Viewing Data

----- Analyses of and rationale for heaviest viewed videos

----- Analyses of videos usage on multiple devices (PCs, laptops, smartphones, via iOS or Androids)

----- Analyses of formal video usage in Education (Medical or Nursing) vs. informal videos (for Administration, general news announcements, etc.)

-----Analyses/discussion of student tracking and class completion requirements, including data and rationale for partial viewing vs. viewing to completion

Results: Positive outcomes occurred in the following areas:

• High production services and viewing services usage in education programs and all hospital departments

• Increase in demand for video productions annually

• Significant savings to the hospital for in-house productions vs. outsourcing costs for similar products

Challenges included copyright, HIPAA regulations, data gathering standardization, budget restraints (equipment purchases and MMS staffing), and project management in a busy, active hospital.
Future directions include collection assessment for video retention or disposition, and investigations of a) adding OCLC/WMS records for increased discoverability, and b) increasing access possibly to more mobile-responsive platforms**.**

Conclusions: Video productions are useful tools for educational, formal communications, as well as informal communications within our hospital. Video use and production requests were heaviest in GME and Nursing Education programs. However, other departments (administration, individual units, quality, marketing, etc.) also requested creating videos to disseminate information. Videos were viewed by every department, via many venues, including Manager Meetings, All-Hands meetings, and mandatory training assigned to staff. Learners benefited from multiple learning modalities and could view video on demand. Significant cost savings were realized due to reduced outsourcing of video productions. Subsequently, MMS popularity increased requests for video productions each year.

Keywords: Video Productions, Multi-Media Services, Medical Education, Nursing Education, Hospital Communications

Poster Number: 137

Time: Sunday, May 20, 2:00 PM – 2:55 PM

An International Collaboration: Team-Based Informationist and Physician Instruction in Ghana

Emily C. Ginier, Informationist, University of Michigan, Taubman Health Sciences Library, Ann Arbor, MI**;** **Gurpreet K. Rana**, Global Health Coordinator, Taubman Health Sciences Library, Taubman Health Sciences Library, Ann Arbor, MI

Objectives: In October 2016, Informationists from the University of Michigan Taubman Health Sciences Library (THL) traveled to Ghana to provide instruction to residents and medical trainees. The informationists provided formal information skills training which is not as prevalent in West Africa. In collaboration with two attending physicians at Korle Bu Teaching Hospital (KBTH), the largest medical center in Ghana, the informationists developed an interactive workshop for obstetrics and gynecology residents, fellows, and selected faculty.

Methods: Keeping up with information resources and strategies is key to success in an ever-changing world. With the increasing volume of medical literature, clinicians and researchers need to continuously develop their skills in the process of identifying, appraising, utilizing and managing information in the clinical and research environments effectively and efficiently. The workshop that was developed in collaboration with the two Ghanaian physicians built awareness of health information resources and data sources; taught strategies to find high-quality, critically-appraised evidence; and provided an introduction to Mendeley as an information management tool to improve research workflow. The workshop was made up of two parts: a didactic lecture followed by small group interactive consultations on specific research or clinical questions.

Results: The workshop was extremely well-received with requests for additional instruction sessions. Subsequent activities include surveying the residents on their use of information resources introduced during the workshop and identifying perceived impact on their clinical and research activities.

Conclusions: The informationists became more familiar with teaching in a comparably low resource setting. Challenges included internet speed and availability and addressing trainees' varied levels of information literacy proficiency. Additionally, some trainees were less computer literate than their colleagues. As instructors, the informationists had to be cognizant of the varied skill levels while teaching. The team of THL informationists and KBTH physicians plan on assessing trainees' confidence and perception of information seeking skills and will consider opportunities for asynchronous digital learning.

Keywords: global health, instruction, collaboration, medical education, international, obstetrics, gynecology, developing

Poster Number: 138

Time: Tuesday, May 22, 2:00 PM – 2:55 PM

3D Printing Pilot: Testing a Potential 3D Printing Service in a Health Sciences Library

Edith Starbuck, Associate Information Services Librarian, Donald C. Harrison Health Sciences Library, University of Cincinnati, Cincinnati, OH; **Tiffany J. Grant**, Research Informationist, University of Cincinnati Science and Engineering Libraries, Health Sciences Library, University of Cincinnati, Cincinnati, OH

Objectives: The Health Sciences Library purchased a MakerBot Replicator 3D Printer in the fall of 2015 to explore providing a 3D printing service. A 3D Printing Pilot was developed to determine potential usage, associated costs, staffing requirements, and the feasibility of a self-sustaining (i.e. cost recovery) service based in the library.

Methods: The pilot was available at no cost to faculty, staff, and students on the medical campus. Pilot participants were able to submit files of their choosing, though the library reserved the right to refuse any print job. The pilot submission process was done through Research Electronic Data Capture (REDCap) which collected all information from the original submission file to a 3D printer pilot evaluation survey which was sent to participants after the print job was picked up. The REDCap project included two surveys that were filled out by the participant and two data entry forms filled out by library faculty and staff. Staff from multiple areas of the library were essential to the 3D printing pilot process. The pilot was to be done over a 4 week period but printer malfunctions extended the pilot another month.

Results: There were a total of 54 3D print requests, many of which were repeat participants. The majority of print jobs were for personal use although a few were scientific in nature. Print job duration ranged from ten minutes to thirty hours and were between 2 and 371 grams in weight. The pilot process management was challenging. Of the 54 print requests submitted, only 30 print jobs were completed during the pilot. During the pilot period, the MakerBot 3D printer required replacement parts and ultimately a replacement printer. These requirements caused delays, extended the pilot, and left pilot participants dissatisfied.

Conclusions: Pilot participants who received their print jobs were primarily satisfied with their 3D printed item, and most indicated they would use a 3D printing service if offered, although fewer were certain they would be willing to pay for the service. Although the MakerBot Replicator was able to print some relatively detailed 3D print jobs, it was not clear this model would be able to print more intricate designs. Nor is this model robust enough to endure continuous use as a library sponsored service. Dedicated staffing and a different 3D printer make would also be required.

Keywords: 3D printing pilot, MakerBot Replicator, REDCap data collection

Poster Number: 139

Time: Sunday, May 20, 2:00 PM – 2:55 PM

Implementation of a Cloud-Based Self-Checkout System at a Health Sciences Library

Lori Snyder, Collection Development and Digital Resource Management Librarian; **Xiaoyu Sun**, IT Generalist 4; **Cynthia Robinson, AHIP**, Association Dean for Library & Information Services and Library Director; The Pennsylvania State University, Harrell Health Sciences Library: Research and Learning Commons, Hershey, PA

Objectives: The library wanted to pilot a self-checkout system at the main library in preparation for a satellite location staffed by a single librarian, focused on outreach and teaching. The self-check system had to work with the library's existing integrated library system and information technology infrastructure, require minimum technical support and maintenance, and be easy to use.

Methods: The library implemented a cloud-based self-checkout system at the main library location that uses an app on the borrower's mobile device for check-out. Implementation was seamless, requiring only an open SIP connection in the library's ILS for set-up. The library chose to purchase an iPad to run a version of the app for library users without a smartphone or tablet.

Results: The usage for the self-checkout system has been low despite an advertising campaign by the library. This may be a result of a number of factors such as the library's move to a primarily electronic collection, a general decrease in the circulation of non-reserve materials (reserve items cannot be checked out with the system), or the extended hours the main library is staffed with circulation personnel.

Conclusions: The self-checkout system moved to the satellite location in April 2018. Both the initial implementation and the move to the new location were straight-forward with only a few challenges. The library will evaluate the effectiveness of the self-checkout system in its new location over the next year.

Keywords: Access services, Circulation, Self-Checkout, Cloud-based systems

Poster Number: 140

Time: Monday, May 21, 2:30 PM – 3:25 PM

Meeting Students Where They Learn: Out of the Lecture Hall and into the Simulation Lab

Jill Deaver, Reference Librarian/ Assistant Professor, Lister Hill Library of the Health Sciences/ Reference Department, Birmingham, AL; **Fenil Patel**, Simulation Coordinator, University of Alabama at Birmingham, Office of Interprofessional Simulation for Innovative Clinical Practice, Birmingham, AL; **Shilpa Register**, Director of Research, UAB Office of Interprofessional Simulation for Innovative Clinical Practice, Birmingham, AL

Objectives: To investigate the model of simulation as a framework for a.) observing existing knowledge of information seeking and b.) discovering effective methods to improve medical students' searching skills in their clinical years.

Methods: In this pilot, medical students will participate in simulation training designed to investigate skills in seeking and collating information related to a specific patient case. Students will participate in a prebrief session, a twenty-minute search activity using resources of their choosing, a debrief session, and an exit survey. The survey consists of Likert scale and open-ended questions assessing comfort levels, confidence levels, and information seeking behavior satisfaction.

Results: We had 2 medical students in our pilot study. Both participants arrived at the correct diagnosis (thrombocitopenic purpura). One participant utilized Google to narrow potential differentials, followed by a more specific search using PubMed. The other participant searched normal values and utilized Up to Date to find validation of his/her clinical decision making.

Conclusions: The pilot study verified the importance of having an effective search strategy to validate and support clinical decision making in novice medical students. Future directions include more pilots for additional data.

Keywords: Information seeking behavior; Librarians; Simulation Training; Medical Education; **Teaching**

Poster Number: 141

Time: Tuesday, May 22, 1:00 PM – 1:55 PM

Searching by Author Affiliation: Challenges in Tracking Faculty Research Output

Mary Shultz, Director, University of Nevada, Reno School of Medicine, Savitt Medical Library, Reno, NV**;**
Sandra L. De Groote, AHIP, Scholarly Communications Librarian, University Library, Chicago, IL

Objectives: Literature searching by author affiliation is one method to track faculty scholarly productivity. This is challenging when a single institution has multiple locations or where large state systems have separately accredited universities. Results may also be impacted by inconsistencies in authors' affiliations. This study will identify specific challenges and propose best practices for affiliation searches to track faculty output.

Methods: Author affiliation searches were run in PubMed for medical faculty at both a medium-size state university and a large-size state university. Variations in search strategies were used to determine the most effective strategy to achieve the highest intersection of precision and recall.

Data was collected on numbers of false hits per search strategy. Results of each affiliation strategy were compared to a small sample of known author name searches from each university to determine the likelihood of missed citations. Results were analyzed to determine the best techniques to track scholarly output by author affiliation.

Results:
Multiple search strategies for author affiliation were conducted for both a medium-size and large size medical school. The results retrieved varied across all search strategies for both institutions. Further analysis of results is currently underway to determine the numbers of false hits. The initial results show inconsistencies in how authors designate their affiliation and the impact of this on the accuracy of PubMed author affiliation searching. These inconsistencies also affect the ability to track scholarly output through automated PubMed searches.

Conclusions:
There is a need for an institution protocol for author affiliation which provides faculty with guidelines for submitting their affiliation when publishing. Greater consistency in published affiliations will lead to better tracking of research productivity over time.

Keywords: Author Affiliation Searching
Scholarly output

Poster Number: 142

Time: Tuesday, May 22, 2:00 PM – 2:55 PM

From Class Project to Published Work: A Librarian's Role in Publishing

Michele R. Tennant, AHIP, Associate Director, Health Science Center Library, Biomedical and Health Information Services, Health Science Center Libraries, Gainesville, FL; **Grace L. Gibson**, Student, University of Florida, Environmental Science, Gainesville, FL; **Soleil T. Nguyen**, Student, University of Florida, Architecture, Gainesville, FL; **Michel Eric Andriamihajanirina**, MS, 0, Marketing and International Commerce, Antananarivo, Antananarivo, Madagascar

Objectives: University librarians support institutional missions beyond the walls of the academic health center, with efforts including instruction to undergraduates and international outreach. A librarian at the Health Science Center Library at the University of Florida worked with numerous partners to edit and publish a conservation-themed children's book. Community health can be tied to the health of that community's environment; education can be a first step in improving both.

Methods: The librarian taught a one-credit Honors course on the book "Thank You, Madagascar: the Conservation Diaries of Alison Jolly". The course required students to develop an original class project. Two students wrote and illustrated an engaging children's alphabet book, concentrating on Malagasy animals and plants. Based on the exceptional quality of the work, the librarian was inspired to have the book published, with the goal of distribution to NGOs and other educational/conservation entities within Madagascar. As editor, the librarian consulted with educators in Madagascar to examine cultural nuances, and with on-campus experts for publishing and copyright issues. Based on feedback, she worked with the student authors to identify culturally-appropriate content, and partnered with a Malagasy colleague to reorganize the layout for clarity, simplify the text for age-appropriateness and translate the final product into Malagasy.
Results: The illustrated children's book "Madagascar From A to Z" is the end product of these collaborations. This book is expected to be distributed to non-governmental agencies, schools, and research centers in Madagascar during UF's annual study abroad course in July of 2018.
Conclusions: Collaborations external to the library can grow in unexpected and meaningful ways, while still supporting the missions of the institution; in this case the missions of instruction, service, and internationalization. Working closely with students and the Malagasy colleague has enriched the librarian's work, and resulted in a collaborative tangible product that promises to make a difference.

Poster Number: 143

Time: Sunday, May 20, 2:00 PM – 2:55 PM

Implementing a High-Performance Computing Service

Alexa Mayo, AHIP, Associate Director for Services; **Jean-Paul Courneya**, Bioinformationist; Health Sciences and Human Services Library, Baltimore, MD

Objectives: Despite having an ideal setup in their labs for wet work, researchers often lack the tools to analyze the magnitude of data that result from experiments. Here we describe the Library's support for analysis of high-throughput data for global molecular profiling by offering a high performance computer with open source software along with expert Bioinformationist support.

Methods: To meet the research needs of users requiring high-throughput data analysis and computation when dealing with small or mid-size datasets, the Library offered a high performance computer (HPC) complemented by educational programming. The Library's Bioinformationist identified a stack of open source bioinformatics software to provide analysis options for experimental data generated in University labs such as: flow cytometry, scientific images, DNA, RNA, and Protein. To encourage its use, the HPC was promoted to targeted groups at the University. The Bioinformationist developed self-guided learning materials and offered one-on-one consultations and workshops on topics such as NCBI BLAST, Bioinformatics on the Cloud, and ImageJ. Researchers are able to apply the data analysis techniques learned in the classroom in an ideal computing environment, closing the loop between learning and practice. The HPC was funded by the University through a competitive process.

Results: An online reservation system was put in place to allow researchers to schedule HPC use. The Bioinformationist and IT Systems Engineer developed procedures to ensure data security. Selected use cases: a student analyzed high resolution CT data and produced images for an upcoming scientific conference. Faculty members used the HPC's licensed software, Pathway Studio, to evaluate the interconnectedness of targets of interest found in their high-throughput data. It was also used to put together a visualization of primary data for a grant application. Using a local instance of Galaxy, an open-source bioinformatics software framework, a researcher downloaded publicly available sequence data from NCBI Sequence Read Archive and reproduced the data analysis to validate its rigor and reproducibility.

Conclusions: The Library has successfully implemented an HPC with state-of-the-art open source and licensed bioinformatics software. This allows faculty, staff, and students to learn in a sandbox environment and analyze high-throughput data that they can't on their own computers.

Keywords: high performance computer
Bioinformatics
Bioinformatics open source software

Poster Number: 144

Time: Monday, May 21, 2:30 PM – 3:25 PM

The Background of Health Sciences Librarians: How They Are Adapting, Transforming, and Leading

Jessica A. Koos, AHIP, Health Sciences Librarian, Stony Brook University Health Sciences Library, Ronkonkoma, NY; **Laurel P. Scheinfeld**, Health Sciences Librarian, Stony Brook University Health Sciences Library, Hauppauge, NY

Objectives: To gain insight into what factors may contribute to choosing health sciences librarianship as a career.

Methods: A 15-question survey was developed in order to gather information regarding respondents' educational backgrounds, professional experience, current employment situation, professional development preferences and how they discovered the field of health sciences librarianship. Both quantitative and qualitative information was gathered. The survey was distributed via the MEDLIB-L listserv and the CANMEDLIB listserv, and remained open for approximately one month. It was open to any individual self-identifying as a health sciences or medical librarian currently residing in the United States or Canada.

Results: A total of 397 respondents participated in the survey. The results indicated that health science librarians have highly varied educational and career backgrounds. Less than one-third of respondents reported having a science background and approximately half worked in other types of libraries prior to a health science library. Multiple reasons for choosing to work in this field were provided with “interest in health sciences” being the most popular response. Knowledge of medical terminology was most often gained through professional development opportunities and independent study.

Conclusions: The multifaceted characteristics of health sciences librarians needs to be taken into account when designing educational programming and recruiting for the field.

Keywords: career development, education, professional development

Poster Number: 145

Time: Tuesday, May 22, 1:00 PM – 1:55 PM

Taking Action for Open Access: Upload-a-Thon at the Faculty of Dentistry

Natalie Clairoux, Librarian, Bibliothèque de la santé, Montréal, PQ, Canada

Objectives: In November 2016, a dedicated collection for Faculty of Dentistry (FMD) publications was created in Université de Montréal's institutional repository. Several solicitation emails were sent to faculty members afterwards; yet, only 5 post-prints were uploaded in the open archive by October 2017. As FMD researchers contribute to about 50 articles every year, a more personalized approach was clearly required to encourage deposits.

Methods: The dental librarian organized an "Upload-a-thon" as suggested by SPARC's Open Action Kit for Open Access Week 2017. First, she identified potential authors using data from an alerts-based EndNote database of FMD publications. Fifty-three articles were published between October 2016 and September 2017. To ensure that article post-prints were readily available for deposit, the librarian focused on a subset of 21 papers where 9 individuals had the corresponding author status. Next, these researchers were invited via email to send their post-print(s) directly to the librarian, who had already verified the journals' self-archiving policies. Upon a positive response, the paper was forwarded to the repository's cataloguing team, thus bypassing the submission form. The message also included links to register for the dental librarian's Open Access workshop and to the libguide on this topic. Finally, impromptu visits were made to corresponding authors’ offices to discuss Open Access benefits and share color-themed cupcakes.

Results: Six corresponding authors responded favorably to the librarian's request by sending 19 potential articles to the librarian. Moreover, one author asked a colleague to forward an article too. Seven participants attended the workshop, including one FMD faculty member who later submitted 6 articles. After verification, there was a total of 23 uploads to the institutional repository (10 post-prints and 13 publisher's PDFs). By April 1, 2018, these records had generated 430 downloads. The Cupcake Tour allowed the librarian to engage with targeted authors and further disseminate open access information to 10 other faculty, students or staff members.

Conclusions:
Successful uploading results were achieved using a combination of 1) targeting potential authors; 2) favoring a personal interaction via a tailored email or an open discussion after the workshop. Offering comfort food captured the attention of additional members at FMD. Consequently, the "Upload-a-thon" will be planned again next year. In addition, now that a first contact has been established and champions identified, the librarian plans to solicit them directly when their name turns up in a bibliographic database alert.

Keywords: Open Access, Institutional repositories, Dentistry, Scholarly Communication, Marketing, Outreach

Poster Number: 146

Time: Tuesday, May 22, 1:00 PM – 1:55 PM

Paying Up Front: The Cost of Complete Open Access for Three Academic Units

Jason Burton, Lead STEM Librarian, Indiana University Bloomington, University of Mississippi–Oxford

Objectives: If every article published were published open access, what would the cost be? This study examines the cost for three academic units.

Methods: A bibliography was created for faculty journal publications across Biology, Neuroscience, and Optometry covering 2014-2016. The cost per article was calculated based on the current (2017) article processing charge for each journal title assuming no institutional or personal discount. An average cost per paper, faculty member, and unit was calculated for the three academic units.

Results: Results will be presented at MLA'18

Conclusions: Conclusions will be presented at MLA'18

Keywords: open access, publishing

Poster Number: 147

Time: Sunday, May 20, 2:00 PM – 2:55 PM

Leading the Way: Student Research in the Institutional Repository

Lee Richardson, Information Discovery and Metadata Librarian, University of North Carolina at Chapel Hill, Health Sciences Library, Chapel Hill, NC; **Barbara Rochen. Renner**, Library Services Evaluation Specialist and Liaison, Allied Health Sciences and Professor, Department of Allied Health Sciences, Health Sciences Library, Health Sciences Library and Department of Allied Health Sciences, School of Medicine, Chapel Hill, NC; **Francesca Allegri**, Assistant Director for Clinical, Academic, and Research Engagement, Health Sciences Library, University of North Carolina at Chapel Hill, Chapel Hill, NC

Objectives: This poster reports the process developed and challenges encountered by academic health sciences librarians while creating and piloting a workflow to solicit, describe and deposit student research posters from two Allied Health Sciences programs into an institutional repository (IR). This project, the first of its kind on campus, provides leadership and a framework for others to preserve student research.

Methods: Allied Health students conduct significant research and share results in poster sessions during student research events. This research is not always continued or disseminated in other ways. Therefore, capturing and preserving this gray literature in the IR ensures it will always be available. While each research poster is valuable on its own, collecting them together also provides researchers, faculty, prospective students, and alumni the opportunity to examine student research output as a collection and in the context of other scholarly work from the university. Challenges include exploring various requirements with IR staff; communicating the value of depositing materials into the IR to faculty and students; discovering the best timing, methods, and formats to get the posters and deposit agreements from each student.

Results: Almost 100 Allied Health student posters are available in the IR due to this project. Capturing this part of the intellectual output from the university makes it freely available for anyone, anywhere in the world with internet access (a.k.a. open access). Students now have a stable URL to use for linking and know their content is available whenever needed. This project helped raise awareness of the purpose and benefits of the IR, which may lead to more deposits. The process developed to deposit these posters can be adapted to deposit any health sciences student posters and scholarly work.

Conclusions: Due to the success of this project, library staff will continue depositing Allied Health student research posters into the IR and are considering expansion to other student works such as capstone projects. Library staff will refine and adapt the process as they present it to new students and faculty. Library staff will also use this process to work with colleagues in other health sciences disciplines to deposit student posters. Information will be shared across the libraries with the goal of encouraging deposits from non-health science disciplines.

Keywords: institutional repositories, scholarly communications, student research, Allied Health Sciences, posters

Poster Number: 148

Time: Sunday, May 20, 2:00 PM – 2:55 PM

Perceptions of Health Sciences Librarians in Liaison Roles on the Acquisition of Discipline-Specific Knowledge

Irene M. Lubker, AHIP, Research and Education Informationist, Medical University of South Carolina, Medical University of South Carolina Libraries, Charleston, SC

Objectives: To explore the perceptions of health sciences liaison librarians on how they acquire knowledge in their content areas. The acquisition of discipline specific knowledge and skills can greatly improve a librarians' ability to interact with and understand the research needs of the students and faculty in the different disciplines.

Methods: A preliminary phenomenological qualitative study of health sciences librarians at one public academic research university was conducted. Based on findings from the first study and feedback from colleagues, the study was expanded to other academic health sciences universities. The sample consists of twenty-one diverse population of health science librarians who work in liaison roles at a variety of university health sciences campuses in the United States of America and Canada. Focus groups and semi-structured interviews with flexibility for probing were conducted in person and online to collect data. Field notes and memos were used to enhance data collection. Content analysis of the collected data was used to interpret the findings.

Results: Different factors such as the personal attributes of librarians, the environment of the different health sciences professional schools/programs, librarians' previous education and training are conducive to librarians' acquisition of discipline specific knowledge. Professional development activities from library organizations and non-library entities also play a role in enhancing librarians' knowledge.

Conclusions: The findings of this study could be used to improve on boarding activities for new health sciences librarians and professional development of all other librarians. Conversations around the learning of health sciences liaison librarians in different professional schools should be ongoing.

Keywords: liaisons, qualitative research, research, adult learning, training, perceptions, knowledge acquisition

Poster Number: 149

Time: Tuesday, May 22, 1:00 PM – 1:55 PM

Adapting to New Needs in a Complex Research Environment

Barbara Rochen. Renner, Library Services Evaluation Specialist and Liaison, Allied Health Sciences and Professor, Department of Allied Health Sciences, Health Sciences Library, Health Sciences Library and Department of Allied Health Sciences, School of Medicine, Chapel Hill, NC; **Lee Richardson**, Information Discovery and Metadata Librarian, University of North Carolina at Chapel Hill, Health Sciences Library, Chapel Hill, NC; **Jennifer S. Walker**, Clinical Librarian & Liaison, School of Dentistry, University of North Carolina at Chapel Hill, Health Sciences Library, Chapel Hill, NC

Objectives: Based on input from deans of the university's health sciences schools, library leadership would like to increase librarian support across the research lifecycle. Often, librarians who are early career, new to the university, or new to research intensive settings do not have the background to provide optimal support and need information about research processes, terminology, and research entities on and off campus.

Methods: To provide better research support, informationists and embedded liaison librarians indicated a need to be more conversant with researchers and to understand better how their processes work. Specifically, they need to know how the research works; the training and career lives of researchers, including stressors; major funding mechanisms; terminology; local research supports; overview of rules and regulations; the fuller context of health-related research conducted at a research-intensive university. A senior, PhD-trained librarian collected key information and shared this with others for input. In addition, literature was surveyed to find out more about research training and knowledge needs of liaison librarians participating in research. Other sources consulted included resources and guides from other academic institutions and research agencies. An Information Discovery and Metadata Librarian was added to the project to assist in making the resources discovered more easily findable and usable.

Results: A thorough guide was created to help librarians and others better understand health-related research in order to provide better support to health sciences researchers. An internal LibGuide was created to organize and share content. Anticipated uses include presentations to a variety of library groups, individual or group exploration, focused study of specific content, selection of training programs for in-depth study, review of terminology in preparation for meetings or discussions with researchers, and a repository of information to use in consultation with researchers. The guide is online and editable. An index and table of contents will facilitate ease of use.

Conclusions: The library has a resource to meet a variety of needs, including those of early career liaison librarians and librarians new to the university or to research intensive settings. Librarians are developing learning sessions to review the guide, which will continue to be refined based on feedback. Other possible goals include sharing with librarians across campus who work with researchers outside of the health sciences and exploring how to adapt the content for early career researchers in specific health disciplines.

Keywords: research, researchers, research careers, early career researchers, liaison librarians, informationists, embedded librarians, instruction, research lifecycle

Poster Number: 150

Time: Tuesday, May 22, 2:00 PM – 2:55 PM

In Their Own Words: Physicians and Staff Share How They Use and Value the Library

Hovey Lee, AHIP, Clinical Information Specialist / Manager of Library Services, Kaiser Permanente Panorama City Medical Library, Panorama City, CA

Objectives: This poster describes the planning process of a storytelling campaign in a hospital library and illustrates why storytelling is so important to all libraries marketing strategy. (Name blinded) Medical Library has devised a plan to collect stories and share them with the community with the goal of simultaneously engaging the users while advocating for the library.

Methods: 1) Strategize - Think about the library users, mainly physicians and staff nurses, their wants and needs. How to tell a story that will resonate with them. What will change their perception of the library. Focus on people and benefits, not features or programs. Build a culture of storytelling where librarians reach out to users and ask them to share their library stories.
2) Collect stories - a. Logistics: identify interviewees (go where the users are), thank you notes, create a shared story folder, equipment, formats. b. Design questions to ask: See what they are passionate about and why they think the library is a vital resource.
3) Edit stories - Most importantly adopt an enthusiastic tone; showcase the impact and difference the library's making
4) Distribute stories - as widely as possible by all accessible print and electronic formats

Results: At the time of submitting this poster proposal, stories are being collected and shared with new enthusiasm within the local medical center. Therefore our results are incomplete and evaluation is yet to take place. If successful, this storytelling campaign model can be adopted by all the (Name blinded) libraries and beyond. What storytelling will not fix is ineffective management, strategies or programs, but if done well, it will greatly enhance all other library marketing and communication efforts.

Conclusions: People don't remember talking points, they respond to emotion and feelings, not analysis or numbers. All libraries need to start thinking about telling their stories and engaging users to share their personal experience. When storytelling is done right, it is the most powerful marketing tool for all libraries.

Keywords: storytelling, marketing, hospital library, customer testimonies, communication

Poster Number: 151

Time: Sunday, May 20, 2:00 PM – 2:55 PM

Information Professionals' Role in Building a Genetic Test Knowledgebase

Jennifer Lege Matsuura, Senior Information Specialist, ECRI Institute, Health Technology Assessment Information Service, Ambler, PA; **Michele A. Datko**, Senior Information Specialist, ECRI Institute, Health Technology Assessment Information Service, Plymouth Meeting, PA; **Amy Stone**, Library Director, ECRI Institute, Library, Plymouth Meeting, PA; **Jennifer L. De Lurio**, Knowledge Manager, ECRI Institute, Plymouth Meeting, PA; **Eileen G. Erinoff**, Director, HTA/EPC Information Center, ECRI Institute, Plymouth Meeting, PA

Objectives: Genomics and genetic testing are transforming the medical community. The National Library of Medicine, professional societies, and nonprofit and commercial organizations have developed resources on this topic, but it is difficult to find vetted, multifactorial information. The objective was to develop an evidence-based repository of "tests that matter." We describe information professionals' role in developing a curated genetic testing resource.

Methods: We conducted a needs assessment with potential users, defined the parameters of "tests that matter," determed that the information landscape will need to be continually redefined because of the fast-changing nature of the field, identified the unique data elements that define each test, designed the database structure based on these defining data points, identified and reviewed existing terminology for genetic testing, created scanning processes and work flows to identify new leads and maintain currency of database contents, and implemented a workflow that integrates information professionals and subject specialists to create, populate, review, index, and publish genetic test profiles.

Results: We created a dynamic repository of genetic tests with a rich vocabulary of descriptive test characteristics and a growing user base. Our structured workflow involves a collaborative team of dedicated information and subject specialists, an informed approach to content development, and a robust quality control process.

Conclusions: Information specialists play a critical role in all steps of database development, population, and expansion.

Keywords: Databases as Topic; Genetic Testing; Molecular Diagnostic Techniques; Systems Analysis

Poster Number: 152

Time: Tuesday, May 22, 1:00 PM – 1:55 PM

Research Impact Core: A Research Impact Initiative at the University of Michigan

Tyler Nix, Informationist; **Judith E. Smith**, Informationist**; Marisa L. Conte**, Assistant Director, Research and Informatics; Taubman Health Sciences Library, Ann Arbor, MI

Objectives: The Taubman Health Sciences Library is developing a "Research Impact Core" to provide increased programming and expertise in evaluation metrics, tools, and best practices for the University of Michigan health sciences community.

Methods: The initial focus of the Research Impact Core is to provide information sessions and consultations promoting a greater understanding of the strengths and limitations of impact metrics (e.g, the H-Index) and use of metrics tools. Starting in late 2016, the authors built on existing relationships with similar campus offices offering faculty development programming, pursued skill-building through conferences and training, and talked with librarians who have established services in their institutions. To date, the authors have, 1) offered several drop-in and classroom training sessions and aimed at health sciences faculty, research administrators, and students 2) developed a research guide highlighting metrics tools and resources, and 3) created promotional materials.

Results: The authors will share results including: instructional and promotional content developed to date, challenges encountered, and feedback from initial training sessions and consultations.

Conclusions: The authors will share strategies for next steps in the development of the Research Impact Core.

Keywords: Research impact, research assessment, research evaluation, research metrics, alternative metrics

Poster Number: 153

Time: Tuesday, May 22, 2:00 PM – 2:55 PM

Warm Fuzzies: Boosting Staff Engagement with a Traveling Gopher

Katherine Chew, Research/Outreach Services, Health Sciences Libraries, University of Minnesota–Minneapolis; **Andre Nault**, Veterinary Librarian, Veterinary Medical Library, Veterinary Medical Library, St. Paul, MN

Objectives: An institution-wide employee engagement survey was conducted fall of 2014. One survey metric centered on gauging employee perception on how they were valued and acknowledged for their contributions to the organization. While the results were generally favorable, management decided this could be higher as staff recognition is core to employee engagement, satisfaction, retention, and ultimately patron satisfaction with the organization.

Methods: A staff recognition committee was put together to brain-storm ideas of how to recognize employee contributions that didn't involve the more standard recognition types like "employee of the month". One of the ideas that rose to the top was that of a "traveling award". The idea is for staff to present the traveling award to an individual from whom they received great service (to either patrons or a fellow staff member) or who made their job easier in one form or another. The traveling award is meant to be a peer-to-peer and the award nominator is encouraged to submit a brief description to the internal bi-weekly newsletter of who received the award and why. In addition, all persons receiving the traveling award have their names entered into a quarterly drawing to enjoy coffee or lunch with the library director.

Results: To make the traveling award more fun, a pair of stuffed institutional mascots was purchased (in case of one traveler going astray). The designated traveling award and its cousin were ready to start visiting staff in May of 2015 and proved to be a huge success. Since its roll-out, the traveling award has visited staff 28 times with accompanying write-ups posted in the library's bi-weekly emailed newsletter. Staffs has embraced the traveling awards and have used them to call out colleagues for a wide variety of reasons such as project leadership, administrative support, great customer service, or just "general awesomeness."

Conclusions: Staff wants to be respected and valued for their contributions and respond to appreciation through recognition of their good work because it sends an extremely powerful message that their work is valued and that they are an important part of the organization. Staff that feels that their contributions are valued by their peers and the organization is more likely to have greater job satisfaction, work better together as teams and feel a sense of pride in the organization's goals and values.

Keywords: engagement, recognition, respect, appreciation, job satisfaction,

Poster Number: 154

Time: Tuesday, May 22, 2:00 PM – 2:55 PM

Adapting-Transforming-Leading: A Year in the Life of a National Network of Libraries of Medicine-Sponsored Outreach Librarian

Katherine Chew, Research/Outreach Services, Health Sciences Libraries, University of Minnesota–Minneapolis

Objectives: The National Network of Libraries of Medicine (NNLM) establishes partnerships with health sciences libraries that share the NNLM's mission of outreach. Outreach librarians sponsored by NNLM regional offices conduct a wide range of activities on the behalf of the NNLM to public libraries, health professionals and to the general public with emphasis on instruction on National Library of Medicine resources.

Methods: As a designated Partner Outreach Library for the Greater Midwest Region (GMR) NNLM, the Health Sciences Libraries Outreach Program supports the outreach mission of the National Network of Libraries of Medicine and aids the GMR in its mission to improve awareness of and access to reliable, freely available, online health information resources by delivering programming within the local communities on such topics as health literacy, healthy aging, information resources for immigrants, NLM science resources or outreach librarianship. This is done through exhibiting at strategically chosen health or community events or conferences throughout the year, presenting informational sessions at these conferences, conducting training or workshops at public libraries for library staff or patrons, providing PubMed instructions for local high schools, and participation in GMR grant funded outreach projects.

Keywords: outreach, NNLM

Poster Number: 155

Time: Sunday, May 20, 2:00 PM – 2:55 PM

Innovative Outreach: Creative Approaches to Providing Clinical Librarian Services

Samuel B. King, Assistant Professor, Campus Library Manager, Manchester Campus Library, Manchester Campus Library/Library and learning Resources, Manchester, NH**; Mariana Lapidus**, Reference coordinator, Boston campus, Henrietta DeBenedictis Library, Henrietta DeBenedictis Library, Library & Learning resources, Boston, MA

Objectives: Objective: Determine the extent and nature of clinical librarian programs within a variety of healthcare libraries (including academic institutions). Highlight innovative approaches toward clinical librarianship, especially the application of this service in the virtual world and nontraditional environments such as classrooms and work experience sites. Recent developments at the academic institution where the authors work will be shared.

Methods: An electronic survey will be sent to a broad selection of healthcare and academic libraries through the Medlib-L listserv as well as the MLA Informatics SIG, the Canadian CHLA listserv, the ACRL ITIG/NER, and other library lists. This survey will determine the nature and success of clinical library services as well as identify creative examples which can serve as inspirational benchmarks for other institutions. In addition, respondents will be asked to speculate of future adaptations of clinical librarian services within their institutions.

Results: The results of this survey (when received) will provide a broad picture of the current state of clinical librarian services within the libraries surveyed. As the survey has not yet been conducted, we cannot provide additional information as to the results of this study. However, it is our expectation that valuable statistical data will be collected and valuable examples of the creative application of this service will become available. It will be equally important to identify some service gaps and areas where responses expect the service to evolve.

Conclusions: Clinical librarian programs are active in all regions of the United States and Canada. The majority of these programs are managed by libraries. Most libraries offer clinical librarian services physically AND virtually. In addition to traditional clinical areas, most programs provide extended services such as classrooms, meetings and practitioner/student projects. Most respondes believe enhanced technology will result in the expansion of clinical librarianship. We believe the results, based on different libraries' experiences, will assist librarians as they pursue clinical services within their own institutions, and hopefully inspire more libraries to consider such a service.

Keywords: "clinical librarians", "outreach", "professional role", "computer-assisted instruction",

Poster Number: 156

Time: Monday, May 21, 2:30 PM – 3:25 PM

A Transformative Ghana-US Collaboration: Developing an Innovative Health Sciences Librarian Exchange

Gurpreet K. Rana, Global Health Coordinator, Taubman Health Sciences Library, Taubman Health Sciences Library, Ann Arbor, MI**; Emily C. Ginier**, Informationist, University of Michigan, Taubman Health Sciences Library, Ann Arbor, MI

Objectives: In July 2017, University of Michigan's Taubman Health Sciences Library collaborated with Library Administration at Kwame Nkrumah University of Science and Technology (KNUST) to build an exchange visit for a mid-career health sciences librarian from Ghana. The exchange visit proposed to enhance sustainability of information skills and research capacity in a low resource setting and expand a new collaboration between the institutions.

Methods: After on-site meetings with Ghanaian library leadership to discuss institutional and individual priorities, an agreement of understanding was developed. The visiting librarian was integrated into the U.S. library's existing workflow for a three week period. The visiting librarian was given an opportunity to observe and work with health sciences informationists with diverse specializations and responsibilities, including user populations within the health system and health sciences schools. He also had exposure to librarians, units and initiatives in the broader library system. Learning and working experiences were provided within the academic library’s infrastructure of clinical and classroom-integrated instruction, research activities, data services, expert searching, virtual reference and collection development. The visiting librarian’s itinerary provided opportunities for a bilateral and international exchange of skills, expertise, and innovations in health sciences librarianship.

Keywords: international, collaboration, exchange, capacity building, diversity

Poster Number: 157

Time: Tuesday, May 22, 1:00 PM – 1:55 PM

Transforming Print to Electronic Theses and Dissertations

Sandra L. Bandy, AHIP, Chair, Content Management, Augusta University, **Robert B. Greenblatt**, M.D. Library/ Augusta University, Augusta, GA

Objectives: In response to a changing environment, the library collaborated with The Graduate School (TGS) to transition from print to electronic theses and dissertations (ETD). Since graduate students are writing their thesis and dissertations on a computer, the library initiated the electronic submission to provide long-term archiving of ETDs. This paper discusses new submission processes, including successful strategies and lessons learned.

Methods: The University's existing institutional repository is the new host for ETDs allowing students to upload their final thesis or dissertation into the repository. TGS worked with ProQuest to create an online ETD administrator for students. An ETD Microsoft Word template was designed and programmed by the library according to Graduate School specifications and approved by TGS administrators. TGS dissertation and thesis preparation manual was updated to reflect new formatting and template requirements and a second alternative template was developed for student use. To introduce the new process and workflow to PhD program directors, librarians attended TGS Council meeting. A Graduate School LibGuide for TGS was amended to provide instruction on the new ETD standards and process including the need for additional face-to-face instruction on ETDs. The Library developed an ETD bootcamp for the new process.

Results: After reviewing the initial submission process, the library partnered with ProQuest to simplify their procedures. The library worked with ProQuest to create a Sword protocol for automatic deposits of metadata and PDF files to the repository. Students no longer deposit their final thesis or dissertation into the repository but only to ProQuest eliminating a step in the submission process.

Conclusions: Collaborating with our university's Graduate School created a unique partnership that resulted in new library ETD services for graduate students. Future work with ETDs will focus on retrospectively digitizing the library's print dissertations.

Keywords: ETD, The Graduate School, Dissertation, Thesis

Poster Number: 158

Time: Monday, May 21, 2:30 PM – 3:25 PM

Transforming Library Communications with a Tap: Using iBooks to Familiarize Students, Faculty, and Staff with a New Health Sciences Library

Bredny Rodriguez, AHIP, Health Sciences Librarian, Health Sciences Library, Health Sciences Library, Las Vegas, NV; **Dana Thimons, AHIP**, Health Sciences Librarian, Health Sciences Library, Las Vegas, NV; **Kathryn Houk, AHIP**, Health Sciences Librarian, University of Nevada, Las Vegas, UNLV Health Sciences Library, Las Vegas, NV

Objectives: Develop an innovative tool to effectively communicate essential information about the new library to students, faculty, and staff.

Methods: Librarians serving an inaugural medical school class faced three challenges in sharing new information about library spaces and services:

Due to time restraints, the library was not formally scheduled in the students' orientation. There is no formal orientation for faculty.
The startup environment resulted in frequently evolving policies and procedures.
Usage statistics showed that electronic library guides were not being used as frequently as expected.

The library needed an innovative tool to push new services and policies to users.

Students are provided iPads, and iBooks are being developed to supplement anatomy courses. Apple iBooks can be designed using a variety of formats, including narrative. Multimedia content can be embedded into iBooks. Additionally, content can be pushed through automatic updates.

The librarians saw an opportunity to use iBooks to bring the library's content to life while addressing the aforementioned challenges.

Keywords: iBooks
Communications
Orientation
Outreach
Multimedia
Library guides
Technology
Content Management

Poster Number: 159

Time: Sunday, May 20, 2:00 PM – 2:55 PM

Inquiring Minds Want to Know: How Medical History Mishaps Build Passion for Research

Natalie Logue, Access Services Librarian, Augusta University, Robert B. Greenblatt, M.D. Library, Augusta, GA

Objectives: To explore how flexible pedagogy and active learning was used in an undergraduate Inquiry course to teach information literacy and research practices to students. Inquiry is a required course for undergraduate students taught by faculty throughout the university including medical illustration, history, and physical therapy departments.

Methods: In fall 2016, the Inquiry course “Heroes of Patent Medicine: How Coca-Cola Cured the South and other Strange Remedies” was designed and taught by a librarian using a combined case-study and flipped classroom curriculum. Medical history mishaps was used as the topic of this course to attract students with an interest in health sciences. Students used case studies and in-class discussion to determine their research interests. Out of class research drove the course direction rather than pre-set content. The course culminated in group research projects that included developing research questions, writing an annotated bibliography, and delivering a visual presentation. Student progress was assessed using the Information Competency Assessment (Marshall, 2006), which was distributed at the beginning and end of the course.

Keywords: Information Literacy; course design; active learning

Poster Number: 160

Time: Monday, May 21, 2:30 PM – 3:25 PM

Mapping Student Learning Outcomes to the Association of College and Research Libraries Framework for Information Literacy: Research Skill Integration in an Undergraduate Occupational Science Curriculum

Laura Kuo, AHIP, Health Scienes Librarian, Ithaca College, Ithaca College Library, Ithaca, NY**; Lisabeth Chabot**, College Librarian, Ithaca College, Ithaca College Library, Ithaca, NY**; Julie Dorsey**, Associate Professor, Ithaca College, Department of Occupational Therapy, Ithaca, NY

Objectives: This collaborative assessment study examines a four-year undergraduate Occupational Science (OS) program. A health sciences librarian performed a curricular review by analyzing all required course syllabi which included assignments and course readings. For each year, research-related student learning outcomes (SLOs) were created and mapped to at least one or more of the six ACRL threshold concepts.

Methods: Nineteen OS syllabi and corresponding assignments and readings were closely examined. The four-year program requires students to take one OS course in the first year, two in the second year, five in the third year, one in the summer before their fourth year, and ten courses during their final year. In reviewing the syllabi, the librarian considered the following questions: what content is being taught, when is it being taught, and how is it being taught. Next, the librarian developed research skill-based SLOs. For each SLO, at least one the following ACRL threshold concepts was assigned: Authority is Constructed and Contextual, Information Creation as a Process, Information Has Value, Research as Inquiry, Scholarship as Conversation, Searching as Strategic Exploration. Throughout the process, the librarian consulted with two OS faculty members to ensure agreement with mapping.

Results: 39 SLOs were created and categorized into at least one of the following broad themes: search strategies, resources, and assessment. Search strategy development and resource knowledge emerged as the foci for the first year, decreased during the second and third year, and increased during the final year. Critical assessment of information emerged during the third and fourth year. Searching as Strategic Exploration was the most common threshold concept during the first and second years. During the third year, Research as Inquiry was the most common threshold concept. During the fourth year, Information Has Value occurred more than other threshold concepts.

Conclusions: Creating a curriculum map based on analysis of syllabi, assignments, and readings informed the identification of library research skills that support the OS program. Developing SLOs and grouping them within at least one broad library theme and one or more threshold concept painted a cohesive picture for communicating how and when to provide library instructional support. Earlier in the academic program students are introduced to search strategies, whereas later in the program more complex threshold concepts such as Research as Inquiry and Information Has Value are emphasized. An infographic was developed to illustrate the patterns and results of the analysis.

Keywords: Information Literacy; Curriculum Mapping

Poster Number: 161

Time: Tuesday, May 22, 1:00 PM – 1:55 PM

Transforming Systematic Review Instruction to Inform Global Nutrition Policy Making

Kate Ghezzi-Kopel, Applied Health Sciences Librarian, Albert R. Mann Library, Ithaca, NY; **Sarah Young**, Liaison Librarian, Carnegie Mellon University, Research and Academic Services, Hunt Library, Pittsburgh, PA

Objectives: We present the curriculum, learning outcomes and an informal assessment of impact of intensive librarian involvement in a two-week systematic review (SR) training course. The institute is a partnership between The World Health Organization (WHO), the Cochrane Collaboration and our institution's Division of Nutritional Sciences (DNS). Participants produce SRs to inform WHO guideline development and policy in developing countries.

Methods: We developed curriculum to train researchers to construct SR searches, select appropriate databases, translate search syntax, search the grey literature, use Covidence, manage the SR process and report search strategies for reviews in nutrition and related topics. We serve as active consultants for reviews produced by institute participants, holding office hours in conjunction with the content sessions presented by The WHO, Cochrane and DNS faculty. With each iteration of the institute, which began in 2014, the library curriculum evolves and methods of support are adjusted to best meet participant needs. An assessment of learning outcomes was carried out in the most recent year, and impact in terms of number of participants reached and reviews produced was assessed.

Results: In the first year of the institute in 2014, the librarian taught 2 sessions. In 2017 we taught 5 sessions, consulted extensively with project teams, and received multiple invitations to serve as co-author. 96 researchers from around the world have completed the training and have produced 5 Cochrane reviews, 11 protocols and 15 registered/in progress titles. A pre-assessment in 2017 showed most participants arrived with no skills or basic skills on all SR-related learning outcomes. Those who completed a post-assessment left with intermediate skills or higher on all outcomes, and basic skills on some of the more advanced concepts.

Conclusions: Growth of librarian involvement in this institute shows a need for increased systematic review instruction to inform nutrition policy-making in developing countries. Participants indicated in the post-assessment that they need support and practice developing complex search strategies, translating search syntax, identifying gray literature, and using Covidence. Future directions for the institute could include development of tools to support search strategy development, translation, and documentation to further the impact and effectiveness of these SRs. More broadly, the need for capacity-building among information specialists supporting the growing numbers of researchers doing SRs in developing countries is evident and should be investigated further.

Keywords: Systematic Reviews, Evidence Synthesis, Policy Making, Nutrition, Instruction, Developing Countries

Poster Number: 162

Time: Tuesday, May 22, 2:00 PM – 2:55 PM

Advertising The Library (ATL): Increasing Awareness and Usage of the Library Space and Resources through a Focused Marketing Effort

Sola Whitehead, Medical Librarian, VA Portland Health Care System, Portland, OR

OBJECTIVE: The objective of this library marketing effort was to increase the visibility and awareness of the Library, and to increase usage of the library space and resources by staff, patients, and family members.

METHODS: Several methods were used to increase patient/family member awareness of the library space and resources. The librarian worked with the medical media service to create a trifold brochure for distribution in patient waiting areas. Collaboration with Public Affairs occurred for regular social media posts about the library. In addition, health topic handouts were made available in several hospital areas, and included basic library information.

To increase staff awareness, a consistent and repeating section was added to the weekly facility newsletter. Each announcement has the same header and "look," and features a different library resource. Newsletter articles for the library are included twice monthly. Outreach to service chiefs, resident coordinators, and other management to offer short presentations at staff meetings or other gatherings, is an ongoing effort.

RESULTS: Baseline data were collected for a three-month period beginning August 1st, 2017. Data included number of visitors to the physical library space, circulation statistics, number of staff Athens accounts, and number of sessions on one library-purchased resource (BrowZine). The same statistics were gathered for the three-month period running November 2017 - January 2018.

The total number of visitors to the library increased from 952 in August-October, to 1201 in November-January, an increase of 26%. Circulation showed a slight decrease, from 137 total items circulated August - October to 126 items circulated November - January. Staff Athens accounts increased 13%, from 321 to 363. Finally, the number of total sessions accessing electronic journal content via BrowZine increased 91% from 308 in August-October to 590 in November-January.

CONCLUSION: Marketing efforts are having the desired effect, resulting in increased usage of library resources and increased visits to the library space. However, work is still needed to reach additional patients and family members. Marketing efforts need to be sustainable and ongoing. Marketing efforts will continue, with the intention of assuring that patients, family members, and staff think of the Medical Library when considering their information needs**.**

Poster Number: 164

Time: Monday, May 21, 2:30 PM – 3:25 PM

Teaching Medical School Faculty and Staff to Create Videos in Support of a New Third-Year Curriculum

Carol Gordon, AHIP, Head, Research and Education Services/Assistant Research Professor, Southern Illinois University School of Medicine, SIU Medical Library / Research and Education Services, Springfield, IL; **Lydia Howes**, Research and Education Librarian, Southern Illinois University School of Medicine, Southern Illinois University Medical Library, Springfield, IL

Objectives: Plans for a new innovative approach to the School of Medicine's third year curriculum called for creation of resource videos to supplement the curriculum. Library faculty aimed to increase use of the Library's two-room multimedia recording and editing suite, support the new curriculum and to promote production of information that is eye-catching and more easily consumed.

Methods: Library faculty presented a workshop for curriculum committee members on how to create an educational video. Separate stations in the Library were set up for recording a video, story-boarding, editing, and final production/posting. The next month a similar workshop was held as a pop-up session for school faculty. Research and education librarians provide support for using audio recording and editing and for video editing. The Library offers classes on converting PowerPoint to video using Camtasia software and on creating infographics for educational use. A podcast series produced by one of our librarians has also helped to promote use and awareness of the equipment available in the multimedia suite.

Results: As of March 2018, 47 videos have been created and made available for 3rd year students on a media server. The multimedia suite is now booked regularly by faculty from clinical and basic sciences departments.

Discussion: Most of the faculty did not have experience with educational videos or with multimedia projects. Their busy schedules left them limited time to spend learning to use new software and hardware. The pop-up workshops broke down the process into manageable steps and highlighted resources available in our multimedia suite. We included information in the handout packet regarding the type of projects that could be made using the suite's hardware and software. We also included a list of free video creation and editing resources for those who preferred to work in their offices rather than in the Library.

Keywords: multimedia, educational videos, infographics, curriculum support

Poster Number: 165

Time: Tuesday, May 22, 1:00 PM – 1:55 PM

Transforming a Literature Search Service by Adapting a Reference Management System

Peggy E. Cruse, Librarian; **Susan L. Groshong**, Librarian; **Jacquelyn R. Morton**, Librarian; Library & Information Commons, Seattle, WA

Objectives: Librarians at Seattle Children's Hospital and Research Institute, providing a robust mediated literature search service, lacked a collaborative platform for archiving and sharing search strategies and documentation. Available information request management systems traditionally track a library's reference service workflow. Librarians modified Altarama's RefTracker Express to improve our search quality, increase efficiency, leverage search expertise, and demonstrate alignment with institutional goals.

Methods: Librarians customized RefTracker system forms to capture client demographics, question refinement, resources consulted, and database strategies. In addition to traditional metric categories, librarians adapted records to show the relationship between searches and institutional strategic goals, generating data for service value. All new incoming requests are entered into the system. To jumpstart the internal knowledge base, past searches were selected for inclusion based upon their topic significance and strategy complexity. Before initiating searches, librarians check for duplicative requests and reoccurring search topics. When matches are found, librarians build upon past strategies incorporating peer expertise. Final search result files are uploaded to the request record, allowing for future retrieval and use. Service improvement will be measured by the percentage of searches that use previous RefTracker records.

Results: To date, librarians closed 354 records in RefTracker. Each record captures full requestor information, client-librarian communication, databases searched, search strategies, and the search summary with results. There is an increasing trend in the number of searches completed per month that reuse existing strategies. In the first six months, an average of 5% of requests reused a previous strategy. In the subsequent six months, an average of 27% of requests reused previous strategies. The time saved by using search strategy components from previous requests allows for potential refinement of search concepts, synonyms, and syntax. The archived strategies serve as a knowledge base of the team's areas of expertise.

Conclusions: RefTracker forms are flexible, easy to customize, and easy to incorporate into daily work. Staff training, record audits, and form revisions improve entry compliance and reporting accuracy. Since implementation, data shows improved search efficiency and greater sharing of search expertise. Demonstrating connections to hospital strategic goals is more challenging; the current method is inconsistent and requires ongoing surveillance of hospital communications. Next, the team will implement a RefTracker-automated literature search survey, deployed when records are closed, and use system communication for librarian-client interactions, further streamlining workflow and data entry.

Keywords: literature search service, quality improvement, software implementation, RefTracker Express

Poster Number: 166

Time: Tuesday, May 22, 2:00 PM – 2:55 PM

Library Appy Hour: Effective Collaboration Between Library and Resource Vendors

Rie Goto, Medical Librarian, Hospital for Special Surgery, Kim Barrett Memorial Library / Education and Academic Affairs, New York, NY; **Bridget Jivanelli**, Medical Library Coordinator, Hospital for Special Surgery, Kim Barrett Memorial Library, New York, NY

Objectives: This project describes organizing a successful library event by collaborating with vendors while overcoming barriers such as staff shortage and limited funding. The primary objective for this event was to promote app usage by providing instruction on how to access and use apps. Additionally, instruction on how to configure mobile devices according to hospital's IT specifications was provided.

Methods: After conducting a library user survey in 2016, the library identified areas for opportunities. Improving remote access to library resources topped the list. After reevaluating online resources and usage, it was determined that various apps were underutilized. To promote app usage and to address remote access issues, the library organized an event open to all hospital staff focusing on mobile apps called "Appy Hour". Due to staffing and funding restraints, the library decided to partner with sponsors to share the task of teaching and to support refreshments/catering services. Six information vendors agreed to participate - four in person, two via WebEx. Each vendor had a table and 15 minutes to demonstrate their apps at a podium. The event was advertised via e-mail lists targeted to Medical Staff Attendings, Residents, Fellows, Research Division and the Nursing, Physician Assistants, Physical Therapy Departments.

Results: Appy Hour was held on August 9th, from 5pm to 6:30pm in the hospital's main conference center. We had 28 attendees, including MDs, Residents, Fellows, Nurse Practitioners, Physician Assistants, and Researchers. 4 vendors (7 people) provided onsite demonstrations, and 2 vendors (2 people) provided online demonstration. Evaluation surveys were send out to both attendees and participating vendors.

Conclusions: We are analyzing results from both Appy Hour attendees and participating vendors. Key findings will be presented at the MLA in May 2018. Overall preliminary feedback is positive, with strong possibilities to make it an annual event. Tips to consider when organizing an event like this: Provide a catchy name, select a good catering company and be mindful with your budget, consider credit card payment options, check your institution's donations and gift policy as well as conflict of interest policy, and plan an activity you can enjoy taking part in.

Keywords: Instruction, Orientation, Event, Vendor, Marketing, Library technology, Mobile apps

Poster Number: 167

Time: Sunday, May 20, 2:00 PM – 2:55 PM

Integrating into the Doctor of Nursing Practice Program: A Case Study in Librarian Immersion

Janene Batten, Nursing Librarian, Yale University, Cushing/Whitney Medical Library, New Haven, CT

Objectives: As mid-career nurses, YSN DNP students have a very strong clinical careers, but often lack the advanced skills for information seeking required in a doctoral program. The YSN the DNP Project starts when the student enters the program. It begins with developing a solid background review of existing evidence on a practice change topic that will become their DNP Project.

Methods: The YSN Librarian co-teaches with DNP faculty in the first semester course, Evidence Seminar. This seminar is taught entirely asynchronously online using Canvas. The Librarian developed curriculum to orient students to databases, the skills of solid question building, effective search methods, and literature synthesis techniques. This curriculum was delivered through a series of four online video tutorials, labelled “episodes”, explaining each of these concepts. The Librarian learned how to create voice over PowerPoint to introduce important concepts, and to allow the scaffolding of learning across the four episodes. Each video contained unique objectives, and covered unique content. In this seminar, the Librarian also developed and scored two assignments with skill assessment that reflected the work done in the modules. These assignments account for 70% of the grade for the seminar.

Results: Librarian is seen as an integral teaching colleague in the DNP Program. Students very quickly mature in their techniques for good literature searching across a variety of databases in a number of disciplines. Faculty have heightened expectations for the student work throughout the program knowing that information seeking skills have been addressed. The School is graduating students who continue the good habits of ensuring that they have found the evidence to support their practice.

Conclusions: The DNP is a fast growing terminal nursing degree in the United States. Unlike a PhD program where new research generates new evidence, the focus for students in this degree program is to translate existing evidence into practical situations. The DNP project immersion journey starts when the student enters the program. It begins with developing a solid background review of existing evidence on a practice change topic. The librarian is well placed to assist DNP students as they enter the program to quickly gain the skills to find evidence in support of their DNP project.

Keywords: Doctor of Nursing Practice
DNP
librarian immersion
school of nursing

Poster Number: 168

Time: Monday, May 21, 2:30 PM – 3:25 PM

Leadership in the Interim or: How We Learned to Stop Worrying and Embrace the Uncertainty

Hannah F. Norton, AHIP, Reference & Liaison Librarian; **Michele R. Tennant, AHIP**, Associate Director; **Mary E. Edwards**, Reference & Liaison Librarian; Health Science Center Libraries, Gainesville, FL

Objectives: Library administration provides organizational leadership, support, and direction; functioning well without a permanent director is a particular challenge. We not only maintained stability in the library after the sudden retirement of the director and during a lengthy search for her replacement, but continued to progress including implementing new services and spaces, filling positions, and developing new campus partnerships.

Methods: Following the unexpected retirement of the library's director, the associate director and a newly-tenured librarian were charged with running the library until a new director was found. The associate director's 8-month long sabbatical, five open library faculty positions, and the administrative inexperience of the librarian were additional challenges. Through extensive planning and team-work, playing tag-team interim, and reassigning a third librarian to lead the reference team during the associate director's sabbatical, the team set upon and completed an ambitious plan to move the library forward, rather than be satisfied with the status quo. Before the associate director's sabbatical, the team met frequently to ensure shared priorities and plans for implementing them, including time spent crafting position descriptions for the faculty openings. Throughout, the team communicated consistently, bouncing ideas off one another and making joint decisions whenever possible.

Results: The team accomplished a number of their goals:
• hiring, training, and mentoring five library faculty across two locations;
• implementing 24/7 access to the library's second floor for quiet study;
• creating new teams to focus on marketing and wellness in the library;
• renovating two conference rooms and developing a temporary office space for the forthcoming director;
• revising the library's professional development travel funding system;
• upgrading computers in the library's public spaces through partnership with campus IT;
• mentoring two librarians through the tenure/promotion;
• supervising and evaluating library faculty and staff with minimal upheaval.

Conclusions: Success in a new or enhanced leadership role requires flexibility, open communication, strategic thinking, and confidence. While interim roles inherently involve uncertainty on the part of library personnel, it is possible to maintain stability and move forward in achieving shared library goals through extensive planning and collaboration.

Keywords: leadership, collaboration, interim, management, strategic thinking

Poster Number: 169

Time: Tuesday, May 22, 1:00 PM – 1:55 PM

Transforming the Systematic Review Service

Stephanie C. Roth, AHIP, Biomedical & Research Services Librarian; **Natalie Tagge**, Education Services Librarian; Temple University Ginsburg Health Sciences Library, Philadelphia, PA

Objective:
To transform the systematic review service by moving to a team-based model and to provide quality education and educational services to our researchers (faculty & students).

Methods:
In early 2017, the Health Science Library began plans to transition the current systematic review service to a team-based service model. The systematic review librarian did a scan of other systematic review services and checked the literature for a team-based model. Due to the paucity of open access literature in this area, one librarian at the health sciences library decided to draft and design a unique team based model in an openly accessible format. The education librarian assisted in the development of a unique classification of librarian skills, an intake form for systematic review requests, and a new set of learning outcomes for researchers. Evaluation of the program is continuous and an implementation team is appointed to regularly assess the program.

Results:
Since the new SR model implementation we have:
*Added more librarians from other disciplines (Medical & Science librarians) plus added locations throughout the University (Health Sciences Library, Podiatry Library & Main Campus Library)
*Added more review types to our systematic review library guide and expanded our services
*Created formal marketing for both the literature search service and the educational services
*Provided instruction to main campus Reference & Instruction Librarians & offered mentoring opportunities
*Expanded services and created a new workshop on Systematic Review Tools
*The service was featured in a Temple blog as an example of expanding roles of liaisons
https://sites.temple.edu/assessment/2018/03/05/improving-temple-libraries-system-for-systematic-reviews/
*The Biomedical & Research Services Librarian was asked to participate on a dissertation committee for a Network Meta-analysis
*The number of educational & literature search service requests has increased overall and also in terms of the variety of review types.

Conclusion:
The feedback for the new systematic review service model from librarians at our institutional has been positive. This model is practical and is applied in many ways to support systematic review education.

Poster Number: 170

Time: Tuesday, May 22, 2:00 PM – 2:55 PM

Lessons Learned: Creating User-Focused Library Workshops

Elizabeth Q. Huggins, Lessons learned: transforming library workshops, Loyola University Chicago, Loyola University Chicago Health Sciences Library, Maywood, IL; **Gail Y. Hendler**, Associate Provost and Director, Health Sciences Library, Loyola University Chicago, Maywood, IL; **Tiffany Tawzer**, Access Services and Emerging Technology Librarian, Loyola University Chicago Health Sciences Library, Maywood, IL; **Jeanne Sadlik**, Associate Director, Health Sciences Library, Loyola University Chicago, Maywood, IL

Objectives: Low and inconsistent attendance has plagued the library's information skills workshops since 2014. An evaluation of the workshop series identified new approaches to provide user-focused workshops and increase engagement.

Methods: Feedback was solicited from workshop evaluations and student and faculty representatives of the library advisory committees. Responses revealed insights into scheduling and a general preference for an online format, but few workshop topics were suggested. Current library literature was reviewed for best practice. Trends in workshop scheduling, topic and format from the past two years were identified from academic and health sciences libraries. In June 2016, an assessment of the existing workshop series identified its strengths and weaknesses. Feedback from stakeholders was incorporated into planning the new workshops. Library faculty met to review the new series.

Results: The new workshops had higher attendance and more attendees per workshop than previous iterations. More than 50 people attended the four online and six on-campus workshops during the 2016-2017 academic year. Introduction to Refworks Online, one of the first online workshops, had the largest attendance of the year with more than 20 participants. Hospital staff, residents, and clinical faculty, previously absent, registered for more than half of the new workshops.

Conclusions: Assessing the library workshops, incorporating stakeholder feedback, and collaborating with library faculty lead to user-focused workshops and more engagement with the campus community. Library faculty will continue to evaluate the workshops, review their evaluations and attendance, and propose new topics for future semesters.

Keywords: library workshops

Poster Number: 171

Time: Sunday, May 20, 2:00 PM – 2:55 PM

Intergenerational Communication among Medical Librarians

Jenessa M. McElfresh, Health Sciences Librarian, R. M. Cooper Library, Clemson University, Easley, SC; **Rachel Keiko. Stark, AHIP**, Health Sciences Librarian, Library, California State University, Sacramento University Library, Sacramento, CA

Objectives: The purpose of this study is to review the literature on intergenerational workplace communication among librarians and other front facing service employees. Our hypothesis is that there is no current literature on intergenerational communication among health information professionals, demonstrating a need for further study in this area.

Methods: Relevant publications were identified from multiple academic and professional databases, utilizing various search strategies to perform a comprehensive review of the literature on the topic of intergenerational communication. In addition to searching for scholarly publications that investigate this topic in libraries, we identified and searched for publications that focused on generational studies in occupations and industries with similar service models in order to better understand the broader literature on the topic of intergenerational communication. In order to be as comprehensive as possible, no language restrictions were imposed and the search terms used were broad. The inclusion criteria for articles selected for this study was based upon the most relevant research available from the academic literature, determined through publication topic, validity of research, and date of publication.

Results: In surveying the literature on intergenerational communication among health information professionals, we identified no articles with a specific focus on the health sciences. We then broadened the search to include publications on librarians, and found a select number of both research studies and opinion pieces published that focused on aspects of generation and librarianship. Due to the few recent articles published on the topic, however, many of these publications reflected a pre-Millennial workplace. In expanding our search to include front facing, service industries with similar customer service needs, we found significantly more recent academic publications.

Conclusions: This survey of the literature reveals that there is a demonstrated gap in the academic research conducted on the many aspects of generational diversity in libraries. For health- and medicine-focused specialties in particular, the dearth of literature presents an opportunity for further study. We intend to utilize the research currently available to conduct research in this area, with an emphasis on intergenerational communication.

Keywords: Literature review, generations, age diversity, leadership topics, workplace interactions

Poster Number: 172

Time: Tuesday, May 22, 1:00 PM – 1:55 PM

Leading the Way to Transform Burnout among Health Sciences Librarians

Tallie Casucci, Assistant Librarian, Spencer S. Eccles Health Sciences Library, J. Willard Marriott Library, Salt Lake City, UT; **Amy Locke**, Associate Professor of Family and Preventive Medicine; Co-Director, Resiliency Center, University of Utah, Salt Lake City, UT

Objectives: Measure burnout among health sciences librarians and determine if a wellness game intervention improved personal and workplace wellness.

Methods: A burnout and satisfaction survey was administered to health sciences faculty in summer 2016 and fall 2017. A single item assessed emotional exhaustion, validated to the Maslach Burnout Inventory. The survey was a part of a Health Sciences initiative to address faculty burnout and job satisfaction. Each department selected wellness champions to develop programs to meet identified departmental wellness priorities with specific metrics.

The library wellness champion created a team-based game after participatory interviews. Players collected points for activities related to appreciation, social, mental, and physical wellness. At the game conclusion, a paper-based survey was administered to library employees. The survey included multiple choice and free-text questions. The data were analyzed with descriptive statistics and the grounded theory. Game participants celebrated with an awards lunch.

Results: Twelve library faculty completed the wellbeing and burnout survey. They scored poorly on burnout indicators in both 2016 and 2017. Emotional Exhaustion increased from 43% to 73%. Faculty felt a great deal of stress due to their job (58% in 2016 and 91% in 2017 compared to 45% among other health sciences employees). Factors predicting burnout include sense of control over workload, job satisfaction, stress because of job and finding meaning in work. Highest areas of concerns were a chaotic work environment, work flexibility, sense of control and team collegiality.

30 out of the 59 employees completed the post-game survey. 70% reported the game encouraged them to socialize with colleagues. After coding qualitative data, five categories emerge: socialize (19), motivation (6), fun (5), game play (3), and recognize habits (3). Participants found the wellness game to be a useful strategy in encouraging a more social culture with fun activities.

Conclusions: Similar to previous studies on bibliometric librarians and health professionals (mainly physicians and nurses), health sciences librarians experience burnout. Although the game intervention did not improve burnout or job satisfaction, it did build collegiality and recognition amongst employees. A wellness game can encourage team building, but may not sufficiently address the root causes for health sciences librarian burnout.

Keywords: job satisfaction, burnout, wellness, game

Poster Number: 173

Time: Tuesday, May 22, 1:00 PM – 1:55 PM

Usability Study to Evaluate a Transformed Health Sciences Library Website

Jessica Petrey, Clinical Librarian; **Lauren E. Robinson**, Emerging Technologies Librarian; Kornhauser Health Sciences Library, Louisville, KY

Objectives: The purpose of this study was to evaluate the usability of a newly designed health sciences library website in terms of accurate and efficient information retrieval, as well as overall user satisfaction, in comparison with the previous version of the site.

Methods: Population: Nine self-selected participants from the health sciences campus, including students, residents, and faculty.

Methodology: Comparative usability study of a new and old website administered in survey format. Participants were given five tasks to complete on both websites, while researchers monitored time and accuracy. The Microsoft Desirability Toolkit alongside guided questions were used to solicit additional qualitative feedback.

Results: At the time of submission nine participants had completed the usability test. The number of correctly answered question increased from 4.56 on the old website to 5.00 on the new website, a 9.76% improvement (p=0.0176, Paired, One-tailed). Additionally, the time to completion decreased from 00:04:08 to 00:02:02, a 50.51% improvement (p=0.0066, Paired, One-tailed). The final metric showed a drop from 42 to 14 instances of backtracking, a 66.67% improvement ((p=0.0116, Paired, One-tailed). The reaction card activity yielded a trend of negative card selection for the old website, and positive card selection for the new website.

Conclusions: At the time of submission the quantitative analysis indicates a marked improvement in accuracy as measured by the number of correctly answer tasks and efficiency as measured by time and backtracking. The qualitative analysis indicates a marked improvement in user satisfaction. In addition, the researchers will be implementing suggested improvements after completion of the study.

Keywords: Usability, website design, study design, website usability

Poster Number: 174

Time: Tuesday, May 22, 2:00 PM – 2:55 PM

Transforming Our Teaching: Adapting Our Instruction Program to Better Meet User Needs

Caitlin Meyer, Research & Education Librarian, Cushing/Whitney Medical Library, New Haven, CT

Objectives: To broadly explore and reconsider what educational services medical libraries should be offering their users.

Methods: Taking into consideration both in-person and online instruction, the project aims to identify unmet needs, implement standardized learning objectives in our classes, and create novel educational experiences for our users. The information gathering phase included a comprehensive audit of our existing online materials, taking inventory of workshops and curricular instruction, and a landscape review of peer institutions' library-sponsored classes.

Results: Results and conclusions to be available by the time of the conference.

Conclusions: Results and conclusions to be available by the time of the conference.

Keywords: instruction
assessment
user needs
education

Poster Number: 175

Time: Sunday, May 20, 2:00 PM – 2:55 PM

Just Enough Irons in the Fire: Using an Evidence-Based Block Rotation to Increase Internal Medicine Residents' Knowledge and Usage of Mendeley

Ene Belleh, AHIP, Clinical Information Librarian, Christiana Care Health System, Pennsylvania Hospital - Penn medicine, Philadelphia, PA

Objectives: To demonstrate how a hospital library sought to increase residents' awareness and use of the reference manager, Mendeley by incorporating its features and teaching it simultaneously with the Internal Medicine Residency Program's Evidence-Based curriculum and improving our teaching method in the process.

Methods: As part of the Internal Medicine Residency Program's EBM curriculum, block rotation occurs 3 times per year and has 4-5 residents per rotation. Each block meets with the librarian for an in-depth 2 ½ hours EBM session that includes EBM concepts, hands-on exercises, literature searching, and critical appraisal. Mendeley is introduced and taught in tandem for the managing and organization of results. Mendeley enables residents to collaborate seamlessly during their rotation. The librarian creates a group for each rotation (IM-EBM Block Rotation 1) and has all participating residents create web accounts and download the Mendeley Desktop. All materials (i.e. objectives, PDFs, worksheets, assignments etc) for the EBM sessions are posted in the group. Residents are able to share content, ideas and have discussions back and forth with themselves and the librarian within the group.

Results: Goal of introducing Mendeley and increasing its usage while enhancing the teaching method for the EBM curriculum was simultaneously achieved. As a result the teaching method for the EBM curriculum was more collaborative.

Keywords: Evidence-Based curriculum, Evidence-Based block rotation, Reference Manager, Residency Program

Poster Number: 176

Time: Tuesday, May 22, 2:00 PM – 2:55 PM

Transforming through Wellness Programming at a Health Sciences Center Library

Ariel Pomputius, Health Sciences Liaison Librarian, University of Florida, Health Science Center Libraries, Gainesville, FL; **Jane Morgan-Daniel, AHIP**, Community Engagement and Health Literacy Librarian, University of Florida, Health Science Center Libraries, University of Florida, Gainesville, FL; **Margaret Ansell, AHIP**, Nursing & Consumer Health Liaison Librarian, University of Florida, Health Science Center Libraries, Gainesville, FL; **Nina Stoyan-Rosenzweig**, Senior Associate in Libraries, Health Science Center Libraries, Health Science Center Libraries, Gainesville, FL; **Robert Lockwood**, Senior Library Technical Assistant, University of Florida, Health Science Center Libraries, Gainesville, FL; **Terry K. Selfe, AHIP**, Translational Research and Impact Librarian, University of Florida, University of Florida–Gainesville; **Michele R. Tennant, AHIP**, Associate Director, Health Science Center Library, Biomedical and Health Information Services, Health Science Center Libraries, Gainesville, FL

Objectives: In the highly demanding healthcare field, there is a push for accrediting bodies and curriculum committees to better support the health and well-being of their communities. As a university institution serving all health science colleges, the library can support wellness initiatives in other health science colleges by providing information resources and offering passive and active wellness programming in library spaces.

Methods: A Wellness Team at an academic health center library was created to bring more wellness programming for students, faculty, and staff to the library space. Early wellness pilot programs included a small Wellness Collection of monographs purchased with library funds, and a rotation of collaborative jigsaw puzzles. The team developed a Wellness Initiative Proposal of active and passive programming based on discussions with student wellness-related organizations and a wellness assessment survey of the health center community. Programming suggestions included yoga classes, therapy dogs, meditation classes, pop-up parks, art supplies, artwork, exercise equipment, and coffee break socials; the majority of programming was free or low-cost (<$1000 total). The proposal was presented to department heads of the library for approval in the late summer, and the Wellness Team was encouraged to move forward with several of the proposed programs for the fall.

Keywords: wellness, mindfulness, meditation, therapy animals, art, programs
